# Supplementary material for: Intramedullary nails versus distal locking plates for fracture of the distal femur: results from the Trial of Acute Femoral Fracture Fixation (TrAFFix) randomised feasibility study and process evaluation
Source: BMJ Open. 2019 May 5;9(5):e026810. doi: 10.1136/bmjopen-2018-026810 (PMC6502051; doi:10.1136/bmjopen-2018-026810)
Supplement: Supplementary data [file bmjopen-2018-026810supp001.pdf]

Trial of Acute Femoral Fracture Fixation (TrAFFix), a feasibility study

For patients with an acute, fragility fracture of the distal femur, is there a clinical and cost-effectiveness difference between locking plate fixation and retrograde intramedullary nail fixation?

Protocol version 3.0
05 May 2017

Ethical approval

NRES approval was obtained on the 01/08/2016 with reference number 16/WA/0225

Funding

This study is funded by the National Institute of Health Research Health Technology Assessment (15/59/22)

Sponsorship

The University of Oxford is the sponsor of this study.

Registration

The study has been registered with the ISRCTN registry under reference number ISRCTN92089567, and on the NIHR Portfolio (CPMS ID: 32536).

Dates

Study start date: 01/06/2016
Study end date: 31/01/2018

Table of contents

TABLE OF CONTENTS 2

ABBREVIATIONS 3

1. CONTACT DETAILS 4

2. SYNOPSIS 5

3. RATIONALE 6

3.1 BACKGROUND 6

3.2 GOOD CLINICAL PRACTICE 7

3.3 CONSORT 7

4. TRIAL DESIGN 8

4.1 TRIAL SUMMARY 8

4.2 OBJECTIVES 8

4.3 MEASUREMENTS 9

4.4 SAMPLE SIZE 12

4.5 METHODOLOGY 12

4.5.1 Eligibility 12

4.5.3 Recruitment and consent – process evaluation 15

4.5.4 Randomisation 15

4.5.5 Post randomisation withdrawals/exclusions 16

4.5.6 Blinding 16

4.6 TECHNOLOGIES ASSESSED 16

4.6.1 Treatment options 16

4.6.2 Rehabilitation 17

4.6.3 Follow-up 17

4.7 PROCESS EVALUATION 17

4.7.1 Implementation 18

4.7.2 Mechanisms of impact 18

4.7.3 Context 18

4.8 ADVERSE EVENT MANAGEMENT 19

4.8.2 Risks and benefits 19

4.9 END OF TRIAL 19

5. DATA MANAGEMENT 21

5.1 STATISTICAL ANALYSIS 21

5.2 ECONOMIC EVALUATION 22

5.3 PROCESS EVALUATION 22

6. TRIAL OVERSIGHT 23

6.1 TRIAL SUPERVISION 23

6.2 QUALITY CONTROL 23

6.3 FUNDING 24

6.4 INSURANCE AND INDEMNITY ARRANGEMENTS 24

6.5 DISSEMINATION 24

6.6 PROJECT TIMETABLE AND MILESTONES 25

7. PROTOCOL AMENDMENTS: 26

8. REFERENCES 27

Abbreviations

AE – Adverse Event
CI – Chief Investigator
CRF – Clinical Reporting Form
DEMQoL – Dementia Quality of Life
DSMC – Data Safety Monitoring Committee
DRI – Disability Rating Index
EQ-5D – EuroQol
GCP – Good Clinical Practice
HE – Health Economy/Economist
HTA- Health Technology Assessment
MCA – Mental Capacity Act
MOS – Medical Outcomes Survey
MRC – Medical Research Council
OCTRU – Oxford Clinical Trials Research Unit
PACS - Picture Archiving and Communications System
PI – Principal Investigator
QA – Quality Assurance
QALY – Quality Adjusted Life Year
RCT- Randomised Controlled Trial
REC – Research Ethics Committee
RF – Research Fellow
SAE – Serious Adverse Event
SOP – Standard Operating Procedure
TMG – Trial Management Group
TrAFFix – Trial of Acute Femoral Fracture Fixation
TSC – Trial Steering Committee
VAS – Visual Analogue Scale

1. Contact details

Chief Investigator

Xavier Griffin
Oxford Trauma, Kadoorie Centre
NDORMS, University of Oxford
John Radcliffe Hospital, Headley Way
Oxford, OX3 9DU
xavier.griffin@ndorms.ox.ac.uk

Research Manager

Dr Juul Achten
Oxford Trauma, Kadoorie Centre
NDORMS, University of Oxford
John Radcliffe Hospital, Headley Way
Oxford, OX3 9DU
juul.achten@ndorms.ox.ac.uk

Trial Co-ordinator

Dr Robin Lerner
Oxford Trauma, Kadoorie Centre
NDORMS, University of Oxford
John Radcliffe Hospital, Headley Way
Oxford, OX3 9DU
robin.lerner@ndorms.ox.ac.uk

Trial Management Group

Mr Xavier Griffin
Prof Matt Costa
Dr Juul Achten
Dr Nick Parsons
Dr Robin Lerner
Mr Damian Haywood
Dr Melina Dritsaki
Dr Janis Baird

Trial Steering Committee

Mr Mike Kelly	Chair
Mr Graham Smith	Independent clinician
Dr Ada Keding	Independent statistician
Dr Philip Bell	Independent lay member
Mr Xavier Griffin	Chief Investigator

Data monitoring committee

Mr Dan Perry	Chair
Mr Tim White	Independent clinician
Dr Nikki Totton	Independent statistician

2. Synopsis

Study Title	Trial of Acute Femoral Fracture Fixation.<br>For patients with an acute fragility fracture of the distal femur, is there a clinical and cost-effectiveness difference between locking plate fixation and retrograde intramedullary nail fixation?	
Internal ref. no. / short title	TrAFFix	
Study Design	Feasibility trial for a multi-centre, multi-surgeon, parallel, two-arm, randomised controlled trial	
Study Participants	Participants of 18 years and older who have sustained an fracture of the distal femur and who would benefit from internal fixation of the fracture	
Planned Sample Size	52	
Planned Study Period	01/6/16 - 31/01/18	
	Objectives	Outcome Measures
Primary	• Assess the feasibility of a future definitive trial	• Recruitment rate<br>• Completion rate of EQ-5D-5L at 4 months
Secondary	• Perform a process evaluation to understand the generalisability and likely success of a future trial<br><br>• Explore the validity of self and proxy reporting of the EQ-5D-5L in this population.	• Compliance with peri-operative and post-operative components of protocol<br>• Intervention acceptability<br>• Patient and staff experiences<br><br>• Dementia quality of life measure (DEMQoL)<br>• EQ-5D-5L (proxy and self report)

3. Rationale

3.1 Background

Fractures of the distal femur are increasingly common injuries. They account for 5% of all fractures of the femur with an estimated incidence of 10 per 100,000.[1] The optimal management of these fractures remains controversial. There is a bimodal distribution of the incidence of these fractures with age.[2] The majority, approximately 85%, are fragility fractures sustained by elderly patients after a fall from a standing height, the remainder are typically sustained by multiply injured patients after massive trauma.[2] This study will investigate the commonest fracture subtype – fragility fractures sustained in the elderly population often as a consequence of osteoporosis.

Fragility fractures of the distal femur occur in the same population as hip fractures.[3] Similar to hip fractures, these are significant injuries in a vulnerable group of patients; causing considerable morbidity and mortality as well as placing a major financial burden on the NHS.[4] Hip fracture is approximately ten times more common than fracture of the distal femur.[1] However, the overall incidence of fragility fractures associated with osteoporosis is rising steadily, promising an increasing challenge to future healthcare provision.[5]

There has been very little research exploring treatment options for distal femoral fractures in this population. A recent Cochrane review[6] found few trials in this area, most of which compared outdated implants, such as non-anatomic, non-locking plating systems or earlier generation nails. Furthermore, important limitations in the methodology of each of the trials were identified leading to substantial risks of bias. It was suggested that in order to optimise patient functional recovery following this debilitating injury "a well-designed, adequately powered, randomised controlled trial comparing modern treatments is required".[7]

It is current practice to manage displaced fractures with operative fixation if the patient is medically fit enough to undergo surgery.[3 8] Surgery reduces the substantial complications associated with non-operative treatment, such as prolonged immobilisation and bed rest, as well as the problems of non-union and mal-union.[9]

Since the operative treatment of these fractures was popularised, there has been a wide variety of implants employed to achieve fixation. Despite the significant advancements in implant design and manufacture, operative fixation is still associated with substantial complications similar to those seen with hip fractures.[8 10] This reflects the common pathology underlying these injuries, that the bone is weakened by osteoporosis, undermining the stability of the bone-implant construct.

The two interventions most commonly used in UK practice are intramedullary fixation with a locked retrograde nail (nail), and extramedullary fixation with an anatomical angular stable plate (locking plate).[3] Nails offer twin theoretical advantages; the mechanical impact of a long, intramedullary device that is close to the axis of the femur[11] and the biological advantages of minimum disruption of the fracture site and stimulation of blood supply through reaming.[12] However, nails provide only limited options for distal locking screws, as all screws must pass through the centre of the nail, so the stability of the bone-implant construct may be sub-optimal. Locking-plate fixation has been facilitated by recent advances in implant technology that allow the screws to be screwed into the bone as well as the plate itself ('locked'). This produces a 'fixed-angle' bone-plate

construct. These plates were designed specifically for use in osteoporotic bone, and have been shown to exhibit excellent biomechanical properties.[13] However, they are more expensive than nails and require larger surgical wounds to apply. There are few clinical data available to guide clinicians,[7] and it is clear that there is no current consensus concerning the best management of these injuries.[3]

We performed a multicentre retrospective study to review the current management of distal femoral fractures at four UK major trauma centres.[3] We found that only two devices are now used for fixation – retrograde nails and locking plates. Also, over 80% of these injuries were fragility fractures in a population which matched those sustaining hip fracture. Furthermore, mortality in this frail elderly group of patients was 20% at one year and the prevalence of cognitive impairment 30%. The overall annual incidence in each centre was 20 fractures per year. This retrospective study illustrates the patient demographics, the variability in treatment of these fractures in the UK and the considerable morbidity associated with the injury.

Crucially, we have found that there may be an important difference in outcomes following the choice of surgical management of these patients. The mean benefit of a nail over locking plates may be as great as 0.12 in EQ-5D-derived utility (p=0.019).**[14]** The minimum clinically important difference for EQ-5D is estimated to be 0.08.**[15]** Similar effect sizes are demonstrated in other measures of function and quality-of-life, such as the Glasgow Outcome Scale (extended) and SF-12.[14 16 17] These findings have also been reported by other groups. A small pilot study in the US comparing these technologies found some evidence of a similar benefit in quality of life in favour of nails (mean difference EQ-5D 0.1, p=0.07).[18]

We propose, therefore, to conduct a feasibility study for a later definitive randomised controlled trial comparing functional outcome after treatment with modern intramedullary nails or anatomical locking plates for fragility fractures of the distal femur.

3.2 Good Clinical Practice

The trial will be conducted in accordance with the Medical Research Council's Good Clinical Practice (MRC GCP) principles and guidelines, the Declaration of Helsinki, Oxford Clinical Trials Unit SOPs, relevant UK legislation and this Protocol. GCP-trained personnel will conduct the trial.

3.3 Consort

The trial will be reported in line with the CONSORT statement.[19]

4. Trial design

4.1 Trial summary

The research plan is broken into two phases. Phase 1 is a feasibility study and will determine the expected rate of recruitment in a large scale multicentre randomised controlled trial. Phase 2 will be a definitive multi-centre randomised controlled trial in the UK. This protocol concerns Phase 1 only.

The feasibility study will take place in 6 centres over a period of 20 months. The aim will be to determine the number of eligible and recruited patients in the trauma centres over the course of 10 months. Screening logs will be kept at each site to determine the number of patients assessed for eligibility and the reasons for any exclusions. In addition, the number of eligible and recruited patients, and the number of patients who decline consent/withdraw, will be recorded. Follow-up will be limited to 4 months to determine early completion rates for the outcome measures.

All adult patients (≥18 years old) presenting at the trial centres with an isolated, acute, fracture of the distal femur are potentially eligible to take part in the trial. The study will assess feasibility in patients with fragility fractures and non-fragility fractures. Fragility fractures will be defined using a combination of the mechanism of injury (fall from standing) and age (fractures sustained by adults ≥50 years old). [20] We hope the broad eligibility criteria will ensure that the results of the study can readily be generalised to the wider patient population. A computer generated randomisation sequence, stratified by centre and chronic cognitive impairment will be produced and administered independently by a secure web-based service. Randomisation will be on a 1:1 basis to either nail or locking plate fixation. Both of these operations are widely used within the NHS[3] and all of the surgeons in the chosen centres will be familiar with both techniques.

Demographic data, pre-injury and contemporary functional data will be collected at baseline. Radiographs will be collected at baseline and 6 weeks post-surgery. Follow-up questionnaires will be completed at 6 weeks and 4 months post-surgery. The primary outcome (EQ-5D-5L[21]) will be determined at 4 months, when the majority of recovery has been attained by this population of patients.[3,22]

Patients without cognitive impairment will be asked to complete the EuroQol 5 Dimensions (EQ-5D-5L) health-related quality of life questionnaire[21] and the Disability Rating Index (DRI).[23] Patients with cognitive impairment, or an appropriate proxy, will complete the EQ-5D-5L[21] and Dementia Quality of Life (DEMQoL)[24] questionnaires.

A process evaluation will be performed as part of the feasibility study. The main aim of this evaluation will be to identify barriers and facilitators in the delivery of the interventions and the trial, through a mixed methods approach. This will include qualitative interviews with staff and participants as well as a quantitative assessment of the characteristics of the sample, the fidelity of the interventions and the acceptability of the follow-up schedule. Results from the process evaluation will inform the development of any future definitive trial.

4.2 Objectives

1. Assess the feasibility of a future definitive trial.
2. Perform a process evaluation to understand the generalisability and likely success of a future trial.
3. Explore the validity of self and proxy-reporting of the EQ-5D-5L in this specific population.

4.3 Measurements

The primary outcome measures for this study are the participant recruitment rate and the completion rate of the EQ-5D-5L at 4 months post-surgery. Measurements that will be made during the trial are summarised in Table 1 (overleaf).

Baseline characteristics: Routine baseline characteristics will be recorded for all participants to describe the nature of the participants. Additional measurements will be made to more fully describe the groups:

Grip strength: Grip strength is a measure of muscle strength and gives an indication of sarcopaenia, a predictor of frailty, and will be measured as previously described by Roberts et al.[25]

Frailty: The degree of frailty can provide useful predictive information[26], and will be measured using the Rockwood frailty scale score. The Rockwood scale score is a quick 9-point index used to measure frailty.[26]

Social support: The Medical Outcomes Survey (MOS) social support survey is a brief multi-dimensional, self-administered social support survey.[27]

Self-efficacy: Self-efficacy is a measure of an individual's confidence in their ability to accomplish tasks and overcome problems. Low levels of self-efficacy are associated with less optimal health behaviours.[28]

EuroQol 5 Dimensions (5L) Score (EQ-5D-5L)[21]: EQ-5D-5L is a validated, generalised and standardised instrument comprising a visual analogue scale (VAS) measuring self-rated health and a health status instrument, consisting of a five-level response (no problems, some problems, moderate problems, severe problems and unable) for five domains related to daily activities;[21] (i) mobility, (ii) self-care, (iii) usual activities, (iv) pain and discomfort and (v) anxiety and depression. Responses to the health status classification system are converted into an overall score using a published utility algorithm for the UK population. [29] A respondent's EQ-VAS gives self-rated health on a scale where the endpoints are labelled 'best imaginable health state' (100) and 'worst imaginable health state' (0). EQ-5D-5L has some important advantages in this study. It has been validated for use in patients with cognitive impairment where an appropriate proxy may respond to the questions.[30] It can be administered by mail or by telephone. Our recent work has demonstrated it to have excellent measurement properties in comparison with other commonly used disease and region-specific outcome tools in the similar cohort of patients with fragility hip fracture.[20 31] EQ-5D-5L scores will be collected at baseline (for pre and post-surgery), 6-weeks post-surgery, and 4-months post-surgery.

The secondary outcome measures in this study are:

Disability Rating Index (DRI):[23] The DRI score is a validated self-reported questionnaire. It consists of 12 items specifically related to function of the lower limb. These data will be collected at baseline, 6 weeks, and 4 months post-surgery in participants who do not have cognitive impairment. The DRI[23] has been proven to be a robust, practical clinical and research instrument with good responsiveness and acceptability for assessment of disability caused by impairment in the lower limb.[32]

Dementia Quality of Life Measure (DEMQoL):[24] The DEMQoL[24] score is a validated questionnaire specifically designed to assess quality of life in patients with dementia. A large minority of the participants in this study are expected to have co-existing dementia. The score can be self or proxy-reported and consists of 28 or 31 items respectively. These data will be collected at baseline, 6 weeks, and 4 months post-surgery in participants who have cognitive impairment. Recently preference based utility scores for a UK population have also been published.[33]

Complications: All complications will be recorded. Complications will be classified as either:

- unrelated to the trial protocol,
- related systemic complications (including venous thromboembolic phenomena, death, pneumonia, urinary tract infection, blood transfusion, acute cerebrovascular incident, acute cardiac event, other),
- related local complications (superficial/deep infection, non/mal union, failure/removal/revision of metalwork, injury to adjacent structures such as nerves/tendons/blood vessels, other).

Radiographic evaluation: Standard anterior-posterior and lateral radiographs of the femur will be assessed for mal-union at 6 weeks post-injury. Radiographs are those routinely used for the investigation of patients with a suspected fracture of the distal femur and for the follow-up of such patients following any intervention, so there will be no need to request any additional or special investigations. Radiographs will be assessed by an independent researcher at each site for:

- Evidence of early loss of fixation
- Varus/valgus more than 5°
- Re/procurvatum more than 10°
- Shortening more than 1cm

Semi-structured interviews: Patients, carers and staff will be asked to participate in qualitative interviews to discuss their experience of participating in the trial and the intervention. Interviews will be semi-structured, based on the semi-structured interview guide.

	Pre-surgery	Baseline	6 Weeks	4 Months
**Pre-Randomisation**				
Fracture classification[34]	X			
Cognitive impairment	X	X		
Age	X			
**Personal Details**				
Contact details		X		
**Baseline characteristics**				
Date of birth		X		
Sex		X		
Current medications		X		
Co-morbidities		X		
Current/previous occupation		X		
Educational attainment		X		
Grip strength		X	X	X
Self-efficacy report		X		
Rockwood frailty score		X		
MOS social support		X		
Government benefits		X		
Residential status		X		
Discharge destination		X		
Mobility		X		
**Treatment**				
Additional fixation		X		
Anaesthesia		X		
Grade of surgeon		X		
Prescribed medications		X		
Rehab Assessment		X		
**Outcomes**				
EQ-5D-5L		Pre+post injury	X	X
DEMQoL[A]		Pre+post injury	X	X
DRI[B]		Pre+post injury	X	X
Radiographs		X	X	
Complications		X	X	X
Health Economics				X
Qualitative interviews[C]		X	X	X

Table 1 Follow-up measures. [A]Patients with chronic cognitive impairment, [B]Patients without chronic cognitive impairment, [C]Selection of patients & staff

4.4 Sample size

Data from the feasibility study will be used calculate estimates of the standard deviation of the primary outcome measure (EQ-5D-5L) to drive a formal power analysis and sample size calculation for the definitive trial; evidence from other relevant sources to EQ-5D-5L [20 31] will also be used to inform this process. We anticipate that each of 6 participating centres will treat approximately 1.5 eligible patients per month.[3] Taking a conservative approach, we would expect, to ensure feasibility, to be able to recruit 1.0 patients per month per centre. Given our schedule for centre opening times and recruitment length, we will have 52 centre months available to recruit for this study. Assuming that the recruitment rate is 1.0 per month per centre and monthly centre counts of patient recruitment numbers are approximately Poisson distributed and independent of one another, then this will allow us to estimate the recruitment rate with a 95% confidence interval of 0.73 - 1.28.[35] Therefore recruiting 52 patients in total should provide sufficiently precise estimates of the monthly recruitment rate to decide if a definitive trial is feasible.

For the qualitative interviews, convenience sampling will be used to identify potential participants at different time points. The sampling of patients and staff will be reviewed on an ongoing basis throughout the process evaluation, and interviews will continue until data saturation is achieved.

4.5 Methodology

4.5.1 Eligibility

Patients will be screened against the following criteria:

<u>Inclusion criteria</u>

Patients will be eligible for this study if they:

- are ≥18 years old,
- have a fracture of the femur involving the distal two "Muller" squares,[34]
- would, in the opinion of the attending surgeon, benefit from internal fixation of the fracture.

<u>Exclusion criteria</u>

Patients will be excluded from this trial if they have:

- a loose knee or hip arthroplasty requiring revision,
- pre-existing femoral deformity,
- an arthroplasty that precludes nail fixation.

Patients who sustain injuries to areas of the body other than the lower limbs, which may affect the primary outcome measure, will still be included in the analysis. For patients with bilateral fractures of the distal femur, a rare event, both fractures will be recorded but only one fracture will be included in the trial. The treating surgeon will decide which injury to include in the study prior to randomisation.

All patients who are eligible for inclusion in TrAFFix and their personal consultees, as well as all staff members involved in the research and intervention delivery, are eligible to be approached about participating in qualitative interviews as part of the process evaluation.

Many of the patients who sustain this injury will have some degree of cognitive impairment, similar to patients sustaining hip fracture. Some will have chronic cognitive impairment[3] and some will likely be suffering with acute delirium.[36] Cognitive status is an important, independent predictor of patients' baseline functional, quality-of-life status and likely outcome in this population.[22 36] This is

possibly related to patients' pre-injury independence, post-injury expectations and ability to participate fully in their rehabilitation. Inclusion of these patients is essential to determine specific effect sizes in this subgroup and so that the sample properly reflects the population sustaining this injury. Recent studies investigating hip fracture have demonstrated that contemporary trials including patients with cognitive impairment have good acceptability and are feasible.[37-39]

4.5.2 Recruitment and consenting – for randomised control trial

The clinical care team will notify the research team of any potentially eligible patients. Non-identifiable patient details will then be used for screening in the Emergency Department and Trauma Wards at the trial centres. Reasons for patients' exclusion or ineligibility will be recorded.

The nature of these injuries means that the great majority of patients will be operated on immediately or on the next available trauma operating list, depending on access to an appropriate operating theatre. Some patients may be unconscious, all will be distracted by the injury to their lower limb and its subsequent treatment and all will have had large doses of opiates for pain relief, potentially affecting their ability to process information. Similarly, patients' next-of-kin, carers and friends are often anxious at this time and may have difficulty in weighing the large amounts of information that they are given about the injury and plan for treatment. In this emergency situation the focus is on obtaining consent for surgery (where possible) and informing the patient and any next-of-kin about immediate clinical care. The consent procedure for this trial will reflect that of the surgery, with the clinical team assessing capacity before taking consent for the surgical procedure and this capacity assessment then being used to decide on the proper approach to consenting to the research. The appropriate method, as described below, will then be used to gain either prospective or retrospective consent from the patient or appropriate consultee by a GCP-trained, appropriately delegated member of the research team.

Conducting research in this 'emergency setting' is regulated by the *Mental Capacity Act 2005* (MCA). As patients may lack capacity as described above, and because of the urgent nature of the treatment limits access to and appropriate discussion with personal consultees, we propose to act in accordance with section 32, subsection 9b of the MCA following a process approved by the relevant research ethics committee; the clinical team will make an assessment of capacity as per usual procedures for obtaining consent for a surgical procedure. The clinical team will then provide guidance to the research team as to whether the patient has capacity to consent prospectively or if consultee agreement should be sought.

Where the clinical team advise that prospective patient consent is appropriate, this will be sought by the research team. If the clinical team advise that prospective patient consent is not appropriate, the research team will approach an appropriate Consultee. Where a Personal Consultee is available, they will be provided with the study information. The Personal Consultee will be given the opportunity to ask questions and discuss the study after which their written agreement will be recorded. Where a Personal Consultee is not available then a Nominated Consultee will be identified to advise the research team. The Nominated consultee will be the patient's treating surgeon. If that surgeon is a member of the research team, another independent surgeon will be identified. The Nominated Consultee will be asked to agree for the patient to be randomised, this will be prospectively recorded during the electronic randomisation process. Hereafter, at the first appropriate opportunity and when the clinical situation allows, the Nominated Consultee will provide a wet-ink signature on a copy of the electronic recorded agreement. Consent or agreement for further participation into the study after surgery will then be sought by the patient themselves or a Personal/Nominated consultee.

Those patients who are able to consent before their operation will always be approached for consent before surgery. For those patients that did not consent prior to surgery, the research associate will

provide the patients with all of the study information at the first appropriate time when the patient has regained capacity. The patients will be given the opportunity to ask questions and discuss the study with their family and friends. They will then be asked to provide written consent for continuation in the study. Patients will be asked to consent to long-term follow-up and data linkage to routine NHS datasets.

For those patients who did not prospectively consent or who had a nominated consultee give prospective agreement and still lack capacity after their surgery, a personal Consultee will be contacted to advise the research team about the patients continued participation in the study. The Personal Consultee will be provided with all the study information and be given the opportunity to ask questions and discuss the study with other relatives and friends. If the consultee is present during the consultee agreement discussion, they will be asked to sign a consultee agreement form. In those circumstances where the consultee is not present (for example when they are being contacted via telephone), verbal agreement will be recorded by the research associate on an informed agreement checklist. For all patients, a consultee decision with regards agreement for the patient to continue to be involved in the study, will be recorded in the patient's notes.

On rare occasions, participants may be discharged prior to consent or consultee agreement. If this happens the trial team will make every effort to discuss the trial with the patient at their next clinical follow-up appointment. If the patient lacks capacity at this appointment, the trial will be discussed with the patient's personal consultee.

Patients or Personal Consultees who prefer not to be actively involved in the study follow-up, will be asked if they are willing to consent to the research team using their routinely collected NHS data for the study. All original signed consent forms will be kept in the investigator site file. Three copies of the consent forms will be made; one held in the patient's medical notes, one for the patient and one copy for the study team.

Throughout the study, best efforts will be made to involve participants who, temporarily or permanently, lack capacity in the decision to be involved in the study. The clinical team will make a judgement about the amount and complexity of the information that the participant is able to understand and retain on an individual basis. Appropriate information will be communicated to the participant and updated as their understanding changes. At all times the study team will act in accordance with the participants' best interests. Any new information that arises during the trial that may affect participants' willingness to take part will be reviewed by the Trial Steering Committee; if necessary this will be communicated to all participants. A revised consent form will be completed if necessary.

Responsibility for recording and dating both oral and written informed consent or agreement will be with the investigator, or persons delegated by the investigator, who conducted the informed consent discussion. Delegated responsibility should be recorded on the site delegation log. Permission will be sought to inform the patients GP of their participation in the study.

4.5.3 Recruitment and consent – process evaluation

As part of the initial consent process patients and their personal consultees will be asked whether they may be approached about participating in interviews with regards their views on participating in this trial. Written agreement from patients or their personal consultees to be approached for an interview will be sent to the TrAFFix research office. Patients who do not consent to the main trial may agree to be approached by a member of the research team about taking part in an interview. A researcher from the TrAFFix team will identify which patients will be approached for interview, from those who agree to be approached. A researcher from the TrAFFix team will also identify which NHS staff could be approached for interview.

Participants who agree to be approached will have a separate informed consent discussion for the interviews with a member of the research team either in person, by phone, or by post. NHS staff will initially be invited to interview by phone or email, and if they agree to be approached an informed consent discussion will take place with a researcher from the TrAFFix office by phone, post or in person.

As part of the process evaluation, conversations between researchers and patients or personal consultees may be audio recorded. This may include the initial discussion about TrAFFix, before informed consent for the main study has been give. Where audio recording may be acceptable and appropriate, local research staff will explain the rationale for the process evaluation and recording of the conversation and ask patients whether the conversation can be audio recorded. Where patients agree, their verbal consent will be recorded at the start of the recording. Where patients do not agree, the informed consent conversation will continue as usual without being recorded.

4.5.4 Randomisation

Eligible participants will usually be enrolled by a trial research associate or member of the clinical team using a secure online registration and randomisation system provided by the Oxford Clinical Trials Research Unit (OCTRU). Basic information including the patient initials, age and eligibility checks will be entered. The patient will then receive a trial ID that will be used on all non-public facing trial documentation.

The treatment allocation will be generated prior to surgery, allowing randomisation outside of working hours. The allocation sequence will be stratified by recruitment centre and cognitive status.

Stratification by centre will ensure that any clustering effect related to the centre itself will be equally distributed across the trial arms. This will not eliminate centre or surgeon –specific effects, but as between 10 and 30 surgeons will be involved in the management of the patient group, it is likely that any one surgeon will treat only 2-3 patients enrolled in the trial. Patients will also be stratified by chronic cognitive impairment, which will be judged by the clinical team upon randomisation, to ensure that participants with cognitive impairment will be allocated evenly across the treatment groups.

4.5.5 Post randomisation withdrawals/exclusions

Participants may decline to continue to take part in the trial at any time without prejudice. A decision to decline consent or withdraw will not affect the standard of care the patient receives. Once withdrawn, the patient will be advised to discuss their further care plan with their surgeon. Data collected up to the point of withdrawal will be included in the final analysis. Post-randomisation withdrawals will not be replaced.

4.5.6 Blinding

As the surgical scars are clearly visible, the patients cannot be formally blinded to their treatment. Participants will only be informed of their treatment allocation at the end of the trial. In addition, the treating surgeons will also not be blind to the treatment, but will take no part in the post-operative assessment of the patients. The functional outcome data will be collected and entered onto the trial central database by a research assistant/data clerk in the trial central office. The radiographs collected will be reviewed by independent assessors.

4.6 Technologies assessed

Participants will usually be assessed in the Emergency Department. Diagnosis of a fracture of the distal femur will be confirmed from plain radiographs of the femur. Where there is doubt over the radiological pattern of the fracture, for example whether it extends into the knee or not, participants will be reviewed by the on-call orthopaedic surgeon and where clinically indicated a CT may be performed – this constitutes standard of care practice.

All participants will undergo the following investigations as a minimum: electrocardiogram, full blood count, group and save, coagulation screen, urea, creatinine and electrolytes. Routine thromboprophylaxis will be started in all participants not already receiving anticoagulant therapy. Pharmaceutical and mechanical prophylaxis measures will be used in accordance with current practice agreed at each centre. A regional or general anaesthesia technique will be used and routine analgesia provided according to local practice.

All participants will receive perioperative prophylactic antibiotics in accordance with current practice agreed at each centre. Appropriate preparation, positioning and fracture reduction will be left to the discretion of the operating surgeon, as per their normal clinical practice. Participants will be randomly allocated to fixation using either intramedullary nailing or locking plate fixation.

4.6.1 Treatment options

All the hospitals involved in this trial currently use both of the methods of fixation[3] and all of the consulting surgeons involved will be familiar with both techniques.

Intramedullary nailing: Fixation of the fracture will be achieved with a proximally and distally locked nail that spans the entire diaphysis of the femur. All nails will be introduced retrograde through the knee joint. In this pragmatic trial, the details of surgical incision and approach, fracture reduction and supplementary fixation with wires or screws will be at the surgeon's discretion as per their normal clinical practice.

Locking plate fixation: Fixation of the fracture will be achieved with anatomical distal femoral locking-plate and screws. Locking plates will be defined as those in which at least one fixed angle locking screw is placed distal to the fracture. The operating surgeon will determine the length, number and type of additional screws. Additional fixation with lag screws and cerclage wires will be

at the surgeon's discretion. In this pragmatic trial, the details of surgical incision and approach, fracture reduction, number and type of other screws and supplementary fixation with wires or screws will be at the surgeon's discretion as per their normal clinical practice.

4.6.2 Rehabilitation

Patients allocated to either of the two groups will receive the same standardised, written physiotherapy advice detailing the exercises they need to perform for rehabilitation following their injury. All of the patients in both groups will be advised to move their toes, ankle and knee joints fully within the limits of their comfort. Weight bearing status will be decided by the treating surgeon, with a preference for early weight bearing mobilisation immediately or as soon as the surgeon feels appropriate. In this pragmatic trial, any other rehabilitation input beyond the written physiotherapy advice (including a formal referral to physiotherapy) will be left to the discretion of the treating clinicians. However, a record of any additional rehabilitation input (type of input and number of additional appointments) together with a record of any other investigations/interventions will be requested as part of the 6 week and 4 month postal follow-ups and this will also form part of the trial dataset.

4.6.3 Follow-up

Baseline, 6-week and 4-month clinical report forms (CRFs) will be sent to the trial coordinating centre. The Research Associate will perform a clinical assessment and make a record of any early complications at 6 weeks. Standardised radiographs from 6-weeks post injury will be reviewed by a researcher at each site.

The majority of pre-injury function is recovered in this cohort of patients by 4 months.[20 31] The outcome measures will therefore be collected at this time point. The functional and health-related quality of life outcome data will be collected using the EQ-5D-5L[21], DEMQoL[24] and DRI[23] at baseline (post injury and retrospectively pre-injury), and 6 weeks and 4 months post-surgery.

Where possible patients will complete baseline, 6 week, and 4 month questionnaires while in hospital during their initial stay or during routine follow-up appointments, with the help of an appropriate proxy where necessary. If patients do not complete a questionnaire during a follow up visit, a copy will be sent to their address. If patients have not returned questionnaires, a copy of the outstanding questionnaires will be sent again by post and a follow-up phone call will be made by the trial central office. (The primary outcome questionnaire can be completed over the phone, even if postal copies are not returned). In the unlikely event that the follow-up data can still not be secured, then the patient's GP will be contacted to ensure correct contact details are available for the patient.

As part of routine clinical practice patients will be seen in clinic on a regular basis after this injury. Any further clinical follow-up in the first year after the injury will be at the discretion of the surgeon but will not influence the collection of the standard outcome data.

We will use techniques common in long-term cohort studies to ensure minimum loss to follow-up, such as collection of multiple contact addresses and telephone numbers, mobile telephone numbers and email addresses.

4.7 Process evaluation

We will evaluate intervention implementation, context and mechanisms of impact. This approach is consistent with recent Medical Research Guidance on process evaluation of complex interventions.[40] Process evaluation will enable us to understand how the interventions work and under what circumstances effectiveness is achieved. In this feasibility study we will be developing and testing

methods for process evaluation which might then be modified in the subsequent definitive trial. The findings of the process evaluation might also lead to modifications in the plan for outcomes evaluation and so we will not separate research team members conducting process and outcomes evaluation in the way that might be appropriate in the definitive trial. The process evaluation will be led by a post-doctoral fellow with mixed methods research experience, who will work closely with research nurses and surgeons in the participating centres.

4.7.1 Implementation

We will assess the implementation of study procedures in each of the six participating centres. Specifically, we will assess the fidelity of procedures for screening patients and applying study eligibility criteria, rates of screening and recruitment in each centre over the ten-month recruitment period, application of operative procedures for the two interventions and any deviations from the study protocol, fidelity of delivery and patient compliance with the peri-operative (thromboprophylaxis, analgesia and antibiotics) and post-operative components (rehabilitation and standardised physiotherapy advice) of the study. These data will be collected through standardised recording of the screening and eligibility assessments using an enhanced screening log, in which patients' reasons for refusal to participate can also be recorded, audio recording of a sample of the participant-researcher interactions, interviews with research nurses, surgeons, and physiotherapists and occupational therapists in each participating centre, and transcription of data from patient notes describing peri-operative, operative and post-operative care. Acceptability of the interventions will be assessed through interviews with participants during the post-operative period and will be carried out by the post-doctoral fellow or a member of the evaluation team with appropriate qualitative experience. Those patients who decline to take part in the full study to be invited for an interview to explore the reasons for declining to participate, in order to inform the design of any subsequent definitive trial.

4.7.2 *Mechanisms of impact*

We will work with stakeholders to develop a logic model for the feasibility study. Based on our previous research, the logic model will work from a hypothesis that nail fixation is associated with better outcomes than locking plate fixation. Together with stakeholders, we will identify relevant intermediate outcomes that might be associated with the effect of the interventions on the primary outcomes of interest. Such intermediate outcomes will relate to patient and surgical factors. Likely patient factors include the health of participants, as indicated by the presence of co-morbidities, their physical frailty and psychosocial factors including levels of self-efficacy and social support. Patients will also be interviewed to find out their experiences of treatment. Surgical factors include the skills and treatment preferences of the surgeons participating in the study. These will be assessed through assessment of surgical case-load and expertise collected from operative logs, and questionnaires and interviews completed by staff. .The content of interviews and questionnaires will be developed and reviewed on an ongoing basis during the trial.

4.7.3 Context

Contextual factors have the potential to influence the effectiveness of interventions and can lead to differential effects. The multi-centre nature of the feasibility study, and proposed definitive trial, means that factors within the centres need close consideration. Within the feasibility study we will work with stakeholders to identify important contextual factors and will develop methods for their assessment in the feasibility study in order that they can be refined for the definitive trial. Evaluation of context will focus on three areas: the demographic features of populations served by participating centres; care pathways and configuration of services within each centre; and centre specific issues, related to staffing for example. The approaches used to assess these components will include

interviews with research nurses, surgeons, physiotherapists and occupational therapists, documentary analysis and observation within the centres.

4.8 Adverse event management

Adverse events (AE) are defined as any untoward medical occurrence in a clinical trial subject and which do not necessarily have a causal relationship with the treatment. All AEs will be listed on the appropriate case report form for routine return to the TrAFFix central office. Serious AE (SAE) are defined as any untoward and unexpected medical occurrence that:

- Results in death,
- Is life-threatening,
- Requires hospitalisation or prolongation of existing inpatients´ hospitalisation,
- Results in persistent or significant disability or incapacity,
- Is a congenital anomaly or birth defect,
- Any other important medical condition which, although not included in the above, may require medical or surgical intervention to prevent one of the outcomes listed.

Some SAEs may be expected as part of the surgical interventions, and that do not need to be reported to the trial coordinating centre, provided that they are recorded on the 'complications' section of the CRF. These include:

- Wound infection
- Venous thromboembolic phenomena
- Blood transfusion
- Death
- Pneumonia
- Urinary tract infection
- Cerebrovascular accident
- Myocardial infarction/acute coronary syndrome
- Damage to a nerve, tendon or blood vessel

All other SAEs will be entered onto the Serious Adverse Event reporting form and sent to the TrAFFix office by fax or email within 24 hours of the PI (or delegated clinician) becoming aware of them. Once received, causality and expectedness will be confirmed by the Chief Investigator. SAEs that are deemed to be unexpected and related to the trial will be notified to the Research Ethics Committee (REC) within 15 days of being reported to the TrAFFix office. All such events will be reported to the Trial Steering Committee and Data Monitoring Committee at their next meetings.

All participants experiencing SAEs will be followed up as per protocol until the end of the trial.

4.8.2 Risks and benefits

The risks associated with this study are predominantly the risks associated with the surgery: infection, bleeding and damage to the adjacent structures such as nerves, blood vessels and tendons. Participants in both groups will undergo surgery and will potentially be at risk from any/all of these complications. There are no data to suggest that the risk is greater in one group or another. We believe that the overall risk profile is similar for the two interventions but assessment of the number of complications in each group is an objective of this trial.

4.9 End of trial

The end of the trial will be defined as the collection/receipt of the last follow-up questionnaire from the last participant.

5. Data Management

The Case Report Forms will be designed by the trial coordinator in conjunction with the trial management team. All electronic patient-identifiable information will be held on a secure, password-protected database at the University of Oxford, accessible only to the research team. Paper forms with patient-identifiable information will be held in secure, locked filing cabinets within a restricted area. On all other documentation, participants will be identified by a trial ID and the participant's initials only. Audio recordings of interview and participant-researcher interactions will be transcribed, and the anonymised transcriptions stored on secure servers at the University of Oxford, identified by a trial ID and initials only.

Direct access to source data/documents will be required for trial-related monitoring and/or audit by the Sponsor, NHS Trust or regulatory authorities as required. All paper and electronic data will be retained for at least five years after completion of the trial.

5.1 Statistical Analysis

This feasibility study is not powered to formally assess the size of the treatment effect, rather to estimate the recruitment rate. However, the totality of the data collected will be used to assess the feasibility of a definitive large RCT; recruitment rate being the driver of the feasibility study design on the basis that unless a reasonable recruitment rate can be achieved no formal trial will be possible. The recruitment rate will be estimated based on data collected and a 95% confidence interval determined for this measure.

If the estimated recruitment rate is such that a definitive trial is feasible then no formal analysis will be undertaken and data from the feasibility study will be locked and carried over into the main (definitive) trial. No formal analysis of treatment efficacy will be undertaken in this scenario. However, the study DSMC will see unblinded summary statistics, together with recruitment data, and will advise the TSC with relevant safety or ethical guidance as the study progresses. The reasons and patterns of any missing data, loss to follow-up and participant withdrawals will be carefully considered and reported, with particular emphasis on how these may impinge on the future trial.

If a definitive trial is not feasible, then outcome data will reported in the conventional manner. Baseline demographics (e.g. Age, Gender, cognitive status) will be compared between groups to ensure approximate balance has been achieved. This is a small study (n=52), so group treatment effects are unlikely to be estimated with much precision and consequently inferences will be tentative and reported as such. The main analysis will investigate differences in the primary outcome measure, EQ-5D-5L[21] score at 4 months, between the two treatment groups (nail and plate) on an intention-to-treat basis. In addition a per-protocol analysis will also be reported and early EQ-5D-5L status will also be assessed and reported at 6 weeks. Differences between groups will based on a normal approximation for EQ-5D-5L.[20 31] Tests will be two-sided and considered to provide evidence for a significant difference if p-values are less than 0.05 (5% significance level). The stratified randomisation procedure should ensure a balance in cognitive impairment and recruiting centre between test treatments. Although generally we have no reason to expect that clustering effects will be important for this study, in reality the data will be hierarchical in nature, with patients naturally clustered into groups by recruiting centre. Therefore we will account for this by generalising the conventional linear (fixed-effects) regression approach to a mixed-effects modelling approach; where participants are naturally grouped by recruiting centres (random-effects). This model will formally incorporate terms that allow for possible heterogeneity in responses for patients due to the recruiting centre, in addition to the fixed effects of the treatment groups, cognitive impairment and

other participant characteristics that may prove to be important moderators of the treatment effect (e.g. age and gender).

The main analyses will be conducted using specialist mixed-effects modelling functions available in the software package R (http://www.r-project.org/) where EQ-5D-5L[21] data will be assumed to be normally distributed; possibly after appropriate variance-stabilising transformation. The primary focus will be the comparison of the two treatment groups of patients, and this will be reflected in the analysis which will be reported together with appropriate diagnostic plots that check the underlying model assumptions. Results will be presented as mean differences between the trial groups, with 95% confidence intervals.

Secondary analyses will be undertaken using the above strategy for approximately normally distributed outcome measures such as DRI[23]. For dichotomous outcome variables, such as complications related to the trial interventions, mixed effects logistic regression analysis will be undertaken with results presented as odds ratios (and 95% confidence intervals) between the trial groups. The temporal patterns of any complications will be presented graphically and if appropriate a time-to-event analysis (Kaplan-Meier survival analysis) will be used to assess the overall risk and risk within individual classes of complications.

The reasons and patterns of any missing data, loss to follow-up and participant withdrawals will also be carefully considered and reported. A more detailed statistical analysis plan will be agreed with the Trial Steering Committee at the start of the study.

5.2 Economic evaluation

The feasibility of a future definitive economic evaluation of treatment with modern intramedullary nails or anatomical locking plates for fragility fractures of the distal femur will be investigated in this study. Unit costs for health and social care resources will be collected at 4 months via self-reported patient questionnaires and appropriate proxies (when necessary)[21 29] with the view to inform any future trial. The data collected in the participant questionnaires at each time point will also record indirect costs borne by participants and carers as a result of attending hospital visits; as well as direct non-medical costs (including travel expenses) attributable to their health state. Unit cost data will be obtained from national databases such as the BNF and PSSRU Costs of Health and Social Care.[41] EQ-5D-5L[21] and DEMQoL-U[24 33] will be collected at baseline (pre-injury status and current injury status) and 4 months following treatment allocation in order to estimate quality-adjusted life years (QALYs) in any future trial-based economic evaluation.

5.3 Process evaluation

Qualitative data collected to inform the process evaluation from interviews with patients, surgeons and other staff will be transcribed verbatim. The material will be organised into themes, using inductive coding. Using a constant comparative approach, these themes and their sub-themes will be used to produce a coding framework. The relationship between themes and sub-themes will be illustrated in a thematic map.

6. Trial Oversight

The day-to-day management of the trial will be the responsibility of the Trial Manager, supported by the CTU administrative staff. This will be overseen by the Trial Management Group, who will meet monthly to assess progress. It will also be the responsibility of the Trial Manager to undertake training of the research staff at each of the trial centres. The trial statistician and health economist will be closely involved in setting up data capture systems, design of databases and clinical reporting forms. A Trial Steering Committee (TSC) and an Independent Safety & Data Monitoring Committee (DSMC) will be set up.

6.1 Trial Supervision

Day-to-day management of the trial will be overseen by a Trial Management Group which is made up of the Investigators listed in Section 1 and staff working on the project within OCTRU. A TSC -with an independent Chairman - and DSMC will be set up.

The TSC, which includes independent members, provides overall supervision of the trial on behalf of the funder. Its terms of reference will be agreed with the HTA and will be drawn up in a TSC charter which will outline its roles and responsibilities. Meetings of the TSC will take place at least once a year during the recruitment period.

An outline of the remit of the TSC is to:

- monitor and supervise the progress of the trial towards its interim and overall objectives,
- review at regular intervals relevant information from other sources,
- consider the recommendations of the DSMC,
- inform the funding body on the progress of the trial.

The DSMC is a group of independent experts external to the trial who assess the progress, conduct, participant safety and, if required critical endpoints of a clinical trial.

The study DSMC will agree and adopt an appropriate charter which defines its terms of reference and operation in relation to oversight of the trial. They will not be asked to perform any formal interim analyses of effectiveness. They will, however, review accruing data, summaries of the data presented by treatment group, and will assess the screening algorithm against the eligibility criteria. They will also consider emerging evidence from other related trials or research and review related SAEs that have been reported. They may advise the chair of the Trial Steering Committee at any time if, in their view, the trial should be stopped for ethical reasons, including concerns about participant safety. DSMC meetings will be held at least annually during the recruitment phase of the study. Full details including names will be included in the DSMC charter.

6.2 Quality control

We will institute a rigorous programme of quality control. The trial coordinator will be responsible for ensuring adherence to the trial protocols at the trial sites. Quality assurance checks will be undertaken by the CTU to ensure integrity of randomisation, study entry procedures and data collection. The CTU has a quality assurance manager who will monitor this trial by conducting regular (at least once in the lifetime of the study, more if deemed necessary) inspections of the Trial Master File. Furthermore the processes of consenting, randomisation, registration, provision of information

and provision of treatment will be monitored. Written reports will be produced for the TSC, informing them if any corrective action is required.

6.3 Funding

This study is funded by the National Institute of Health Research Health Technology Assessment (15/59/22)

6.4 Insurance and Indemnity Arrangements

The Sponsor has a specialist insurance policy in place - Newline Underwriting Management Ltd, at Lloyd's of London - which would operate in the event of any participant suffering harm as a result of their involvement in the research. Standard NHS cover for negligent harm is in place for NHS procedures. There will be no cover for non-negligent harm.

6.5 Dissemination

The study monograph will be prepared by the trial management team at the completion of the trial. No patient identifiable information will be contained in any form of dissemination of study results.

6.6 Project Timetable and Milestones

We propose a 20 month study starting in June 2016. The trial timetable is shown below, with key milestones indicated and responsible parties identified:

Month	By date	Activity	Milestone	Responsibility
-4-0		Ethics submission	REC approval	CI/RF
0-2	June 2016	Start study	1st TSC/DSMC meeting	CI/TC
		Finalise trial protocol	Protocol final version	TMG
	July 2016	Complete CRF's	CRF final version	CI/Stat/TC
	Aug 2016	Start recruitment lead centre & feasibility centre 2	1st trial site open for recruitment	TC/CI
3-9	Oct 2016	Start recruitment at feasibility centres 3,4, 5& 6	All feasibility sites open for recruitment	TC/CI
	July 2017	End recruitment	~60 patients enrolled	
6-16	Nov 2017	Complete follow-up all sites	All patients completed follow-up	
17-18	Dec 2017	Statistical analysis		Stat
	Jan 2018	HE analysis		HE
		Reporting and full protocol development		TMG/HE
post	Feb 2018	Study close down	Final TSC/DSMC meeting<br>HTA report	CI/TC<br>TMG

CI Chief Investigator, RF Research Fellow, TMG Trial management group, TM Trial Manager, TSC trial steering committee, DMEC Data monitoring and Ethics Committee, Stat statistician, HE Health Economist, DC Data Clerk

7. Protocol Amendments:

Amendment No.	Date of Amendment	Date of Approval
AM05	Modification of inclusion criteria, extension of recruitment period.	[TBC]

8. References

1. Court-Brown CM, Caesar B. Epidemiology of adult fractures: A review. Injury 2006;**37**(8):691-7.
2. Martinet O, Cordey J, Harder Y, et al. The epidemiology of fractures of the distal femur. Injury 2000;**31 Suppl 3**:C62-3.
3. Smith JRA HR, McArthur J, Morrison R, Yip G, Aquilina A, Kelly M. Distal femoral fractures: The size of the problem in the UK. Orthopaedic Trauma Society. Edinburgh, UK, 2013.
4. Johnell O, Kanis JA. An estimate of the worldwide prevalence, mortality and disability associated with hip fracture. Osteoporos Int 2004;**15**(11):897-902.
5. Bleibler F, Konnopka A, Benzinger P, et al. The health burden and costs of incident fractures attributable to osteoporosis from 2010 to 2050 in Germany--a demographic simulation model. Osteoporos Int 2013;**24**(3):835-47.
6. Griffin XL, Parsons N, Zbaeda MM, et al. Interventions for treating fractures of the distal femur in adults. Cochrane Database Syst Rev 2015;**8**:CD010606.
7. Griffin XL PN, Zbaeda M, McArthur J. Interventions for treating fractures of the distal femur in adults. Cochrane Database Systematic Reviews 2015;***in press***.
8. Zlowodzki M, Bhandari M, Marek DJ, et al. Operative treatment of acute distal femur fractures: systematic review of 2 comparative studies and 45 case series (1989 to 2005). J Orthop Trauma 2006;**20**(5):366-71.
9. Butt MS, Krikler SJ, Ali MS. Displaced fractures of the distal femur in elderly patients. Operative versus non-operative treatment. J Bone Joint Surg Br 1996;**78**(1):110-4.
10. Lu-Yao GL, Keller RB, Littenberg B, et al. Outcomes after displaced fractures of the femoral neck. A meta-analysis of one hundred and six published reports. J Bone Joint Surg Am 1994;**76**(1):15-25.
11. Harma A, Germen B, Karakas H, et al. The comparison of femoral curves and curves of contemporary intramedullary nails. Surgical and Radiologic Anatomy 2005;**27**(6):502-6.
12. Browner BD EC. The science and practice of intramedullary nailing. Philadelphia 1987;**Lea & Febiger**.
13. Miller DL, Goswami T, Prayson MJ. Overview of the locking compression plate and its clinical applications in fracture healing. J Surg Orthop Adv 2008;**17**(4):271-81.
14. Hoskins W SR, Bucknill A. Nails or plates for fracture of the distal femur? Data from the Victoria Orthopaedic Trauma Outcomes Registry. Bone Joint Journal 2016;**in press**.
15. Walters SJ, Brazier JE. Comparison of the minimally important difference for two health state utility measures: EQ-5D and SF-6D. Quality of life research : an international journal of quality of life aspects of treatment, care and rehabilitation 2005;**14**(6):1523-32.
16. Ware J, Jr., Kosinski M, Keller SD. A 12-Item Short-Form Health Survey: construction of scales and preliminary tests of reliability and validity. Med Care 1996;**34**(3):220-33.
17. Jennett B, Snoek J, Bond MR, et al. Disability after severe head injury: observations on the use of the Glasgow Outcome Scale. Journal of Neurology, Neurosurgery, and Psychiatry 1981;**44**(4):285-93.
18. Tornetta P, Egol KA, Ertl JP, et al. Locked plating versus retrograde nailing for distal femur fractures: A multicentre randomized trial. Orthopaedic Trauma Association 2013;**Phoenix**(Arizona).
19. Schulz KF, Altman DG, Moher D, et al. CONSORT 2010 statement: updated guidelines for reporting parallel group randomised trials. BMJ 2010;**340**:c332.

20. Griffin XL, Parsons N, Achten J, et al. Recovery of health-related quality of life in a United Kingdom hip fracture population. The Warwick Hip Trauma Evaluation--a prospective cohort study. Bone Joint J 2015;**97-B**(3):372-82.
21. Brooks R. EuroQol: the current state of play. Health Policy 1996;**37**(1):53-72.
22. Griffin XL, Achten J, Parsons N, et al. Platelet-rich therapy in the treatment of patients with hip fractures: a single centre, parallel group, participant-blinded, randomised controlled trial. BMJ Open 2013;**3**(6).
23. Salen BA, Spangfort EV, Nygren AL, et al. The Disability Rating Index: an instrument for the assessment of disability in clinical settings. J Clin Epidemiol 1994;**47**(12):1423-35.
24. Smith SC, Lamping DL, Banerjee S, et al. Development of a new measure of health-related quality of life for people with dementia: DEMQOL. Psychol Med 2007;**37**(5):737-46.
25. Roberts HC, Denison HJ, Martin HJ, et al. A review of the measurement of grip strength in clinical and epidemiological studies: towards a standardised approach. Age Ageing 2011;**40**(4):423-9.
26. Rockwood K, Song X, MacKnight C, et al. A global clinical measure of fitness and frailty in elderly people. CMAJ 2005;**173**(5):489-95.
27. Sherbourne CD, Stewart AL. The MOS social support survey. Soc Sci Med 1991;**32**(6):705-14.
28. Schwarzer R, Bäßler J, Kwiatek P, et al. The Assessment of Optimistic Self-beliefs: Comparison of the German, Spanish, and Chinese Versions of the General Self-efficacy Scale. Applied Psychology 1997;**46**(1):69-88.
29. Dolan P. Modeling valuations for EuroQol health states. Med Care 1997;**35**(11):1095-108.
30. Hounsome N, Orrell M, Edwards RT. EQ-5D as a quality of life measure in people with dementia and their carers: evidence and key issues. Value in health : the journal of the International Society for Pharmacoeconomics and Outcomes Research 2011;**14**(2):390-9.
31. Parsons N, Griffin XL, Achten J, et al. Outcome assessment after hip fracture: is EQ-5D the answer? Bone & joint research 2014;**3**(3):69-75.
32. Parsons H, Bruce J, Achten J, et al. Measurement properties of the Disability Rating Index in patients undergoing hip replacement. Rheumatology (Oxford) 2015;**54**(1):64-71.
33. Mulhern B, Rowen D, Brazier J, et al. Development of DEMQOL-U and DEMQOL-PROXY-U: generation of preference-based indices from DEMQOL and DEMQOL-PROXY for use in economic evaluation. Health Technol Assess 2013;**17**(5):v-xv, 1-140.
34. Marsh JL, Slongo TF, Agel J, et al. Fracture and dislocation classification compendium - 2007: Orthopaedic Trauma Association classification, database and outcomes committee. J Orthop Trauma 2007;**21**(10 Suppl):S1-133.
35. Cocks K, Torgerson DJ. Sample size calculations for pilot randomized trials: a confidence interval approach. J Clin Epidemiol 2013;**66**(2):197-201.
36. Dolan MM, Hawkes WG, Zimmerman SI, et al. Delirium on hospital admission in aged hip fracture patients: prediction of mortality and 2-year functional outcomes. J Gerontol A Biol Sci Med Sci 2000;**55**(9):M527-34.
37. Griffin XL, McArthur J, Achten J, et al. The Warwick Hip Trauma Evaluation Two -an abridged protocol for the WHiTE Two Study: An embedded randomised trial comparing the Dual-Mobility withpolyethylene cups in hip arthroplasty for fracture. Bone Joint Res 2013;**2**(10):210-3.
38. Griffin XL, McArthur J, Achten J, et al. The Warwick Hip Trauma Evaluation One -an abridged protocol for the WHiTE One Study: An embedded randomised trial

comparing the X-bolt with slidinghip screw fixation in extracapsular hip fractures. Bone Joint Res 2013;**2**(10):206-9.
39. Sims AL, Parsons N, Achten J, et al. The World Hip Trauma Evaluation Study 3: Hemiarthroplasty Evaluation by Multicentre Investigation - WHITE 3: HEMI - An Abridged Protocol. Bone Joint Res 2016;**5**(1):18-25.
40. Moore GF, Audrey S, Barker M, et al. Process evaluation of complex interventions: Medical Research Council guidance. BMJ 2015;**350**:h1258.
41. PSSR Unit., Curtis L. Unit Costs of Health and Social Care. Personal Social Services Research Unit, University of Kent & National Schedule of Reference Costs Year 2012-2013 DoH 2013.
