# Supplementary material for: Intramedullary nails versus distal locking plates for fracture of the distal femur: results from the Trial of Acute Femoral Fracture Fixation (TrAFFix) randomised feasibility study and process evaluation
Source: BMJ Open. 2019 May 5;9(5):e026810. doi: 10.1136/bmjopen-2018-026810 (PMC6502051; doi:10.1136/bmjopen-2018-026810)
Supplement: Supplementary data [file bmjopen-2018-026810supp002.pdf]

TrAFFix

TrAFFix

Trial of Acute Femoral fracture Fixation

Statistical Analysis Plan

Version 2.0 – 2Nov2016

Based on Protocol version 2.0 – 27Jul2016

Name	Title	Role (Author/Reviewer/Approver)	Signature	Date
Nick Parsons	Trial Statistician	**Author**		02Nov2016
Robin Lerner	Trial Manager	**Reviewer**		02Nov2016
Xavier Griffin	Chief Investigator	**Reviewer**		02Nov2016

Oxford Clinical Trials Research Unit (OCTRU) and

Note: this document is version controlled and all draft versions and related communications should be stored in the Trial Master File

CONTENTS

1. INTRODUCTION 3
   1.1 KEY PERSONNEL 3
2. BACKGROUND INFORMATION 3
   2.1 RESEARCH HYPOTHESES AND STUDY OBJECTIVES 3
   2.2 STUDY DESIGN AND TRIAL EXPECTED TIME-POINTS 3
   2.3 ELIGIBILITY 4
   2.4 TREATMENT INTERVENTIONS 4
   2.5 SAMPLE SIZE 4
   2.6 RANDOMISATION 5
   2.7 HYPOTHESES AND DEFINITION OF PRIMARY AND SECONDARY OUTCOMES 5
   2.8 OUTCOMES ASSESSMENT SCHEDULE 5
   2.9 STATISTICAL ANALYSIS OUTLINE 5
3. QUALITY CONTROL AND DATA VALIDATION 7
   3.1 DEFINITION OF DERIVED DATA 7
   3.2 VALIDATION OF THE PRIMARY ANALYSIS 7
4. INTERIM ANALYSIS AND DATA REVIEW 7
5. SPECIFICATION OF STATISTICAL PACKAGES 7
6. DESCRIPTIVE ANALYSES 7
   6.1 REPRESENTATIVENESS OF STUDY SAMPLE AND PATIENT THROUGHPUT 8
   6.2 BASELINE COMPARABILITY OF RANDOMISED GROUPS 8
   6.3 COMPARISON OF LOSSES TO FOLLOW-UP 8
   6.4 DESCRIPTION OF AVAILABLE DATA 8
   6.5 DESCRIPTION OF COMPLIANCE WITH INTERVENTION 9
   6.6 UNBLINDING OF RANDOMISED TREATMENTS 9
   6.7 RELIABILITY 9
7. DEFINITION OF POPULATIONS FOR ANALYSIS 9
8. ANALYSES TO ADDRESS PRIMARY AIMS 9
   8.1 EVALUATION/DEFINITION OF PRIMARY OUTCOME (WHERE APPLICABLE) 9
   8.2 STATISTICAL METHODS USED FOR ANALYSIS OF PRIMARY OUTCOME 9
   8.3 ADJUSTMENT OF P VALUES FOR MULTIPLE TESTING 10
   8.4 MISSING DATA 10
   8.5 PRE-SPECIFIED SUBGROUP ANALYSIS 10
   8.6 TREATMENT BY CENTRE INTERACTION 10
   8.7 SENSITIVITY ANALYSIS 10
9. ANALYSIS TO ADDRESS SECONDARY AIMS 10
   9.1 EVALUATION/DEFINITION OF SECONDARY OUTCOMES (WHERE APPLICABLE) 11
   9.2 STATISTICAL METHODS USED FOR ANALYSIS OF SECONDARY OUTCOMES 11
   9.1 PRE-SPECIFIED SUBGROUPS OF KEY SECONDARY OUTCOMES (WHERE APPLICABLE) 11
   9.2 SENSITIVITY ANALYSIS OF KEY SECONDARY OUTCOMES (WHERE APPLICABLE) 11
   9.3 HEALTH ECONOMICS AND COST EFFECTIVENESS 11
10. ADDITIONAL ANALYSES 11
   10.1 EXPLORATORY ANALYSES 11
   10.2 BLINDED ANALYSIS 11
   10.3 META-ANALYSES (IF APPLICABLE) 12
11. SAFETY ANALYSIS 12

12. APPENDIX: GLOSSARY OF ABBREVIATIONS 12
13. DOCUMENT HISTORY 12
14. CHANGES FROM PREVIOUS VERSION OF SAP 12

1. INTRODUCTION

This document details the proposed presentation and analysis for the main paper(s) reporting results from the ***NIHR HTA funded Trial of Acute Femoral Fracture Fixation.*** The results reported in these papers should follow the strategy set out here. Subsequent analyses of a more exploratory nature will not be bound by this strategy, though they are expected to follow the broad principles laid down here. The principles are not intended to curtail exploratory analysis (for example, to decide cut-points for categorisation of continuous variables), nor to prohibit accepted practices (for example, data transformation prior to analysis), but they are intended to establish the rules that will be followed, as closely as possible, when analysing and reporting the trial.

The analysis strategy will be available on request when the principal papers are submitted for publication in a journal. Suggestions for subsequent analyses by journal editors or referees, will be considered carefully, and carried out as far as possible in line with the principles of this analysis strategy; if reported, the source of the suggestion will be acknowledged.

Any deviations from the statistical analysis plan will be described and justified in the final report of the trial. The analysis should be carried out by an identified, appropriately qualified and experienced statistician, who should ensure the integrity of the data during their processing. Examples of such procedures include quality control and evaluation procedures.

1.1 Key personnel

List of key people involved in the drafting and reviewing this SAP, together with their role in the trial and their contact details.

Author(s) (Trial statistician(s)):

Nick Parsons – Senior Statistician, Trial Statistician

Reviewers (Chief Investigator, Trial Manager, DSMC, TSC, Statistician as appropriate):

Xavier Griffin – Chief Investigator

Approver (Senior Statistician):

Nick Parsons – Senior Statistician, Trial Statistician

2. BACKGROUND INFORMATION

2.1 Research Hypotheses and Study Objectives

1. Assess the feasibility of a future definitive trial.
2. Perform a process evaluation to understand the generalisability and likely success of a future trial.
3. Explore the validity of self and proxy-reporting of the EQ-5D-5L in this specific population.

2.2 Study Design and trial expected time-points

The study design including the type of trial (e.g. parallel group, cluster, crossover, factorial, single arm), single/multi-centre, blinding, allocation ratio, trial framework (e.g. superiority, equivalence, non-inferiority, exploratory), primary outcome and crucial trial time-points.

TRAFFix is a randomized (1:1 allocation ratio) controlled multicentre trial with two (parallel) groups assessing fixation of the femur after fixation using either a nail or a plate.

Date of grant activation	01Jun2016
Date of start of recruitment:	XXAug2016
Date of expected end of recruitment:	31May2017
Date expected end follow-up:	30Sep2016
Date expected start of analysis:	01Oct2017
Date End of grant:	31Nov2017
Target number of subjects:	52
Participating Centres:	6

2.3 Eligibility

Inclusion criteria

Patients will be eligible for this study if they:

- are ≥50 years old as a surrogate for bone-density and therefore fragility fracture,
- have a fracture of the femur involving the distal two "Muller" squares,
- would, in the opinion of the attending surgeon, benefit from internal fixation of the fracture.

Exclusion criteria

Patients will be excluded from this trial if they have:

- a loose knee or hip arthroplasty requiring revision,
- pre-existing femoral deformity,
- an arthroplasty that precludes nail fixation.

2.4 Treatment Interventions

Intramedullary nailing: Fixation of the fracture will be achieved with a proximally and distally locked nail that spans the entire diaphysis of the femur. All nails will be introduced retrograde through the knee joint. In this pragmatic trial, the details of surgical incision and approach, fracture reduction and supplementary fixation with wires or screws will be at the surgeon's discretion as per their normal clinical practice.

Locking plate fixation: Fixation of the fracture will be achieved with anatomical distal femoral locking-plate and screws. Locking plates will be defined as those in which at least one fixed angle locking screw is placed distal to the fracture. The operating surgeon will determine the length, number and type of additional screws. Additional fixation with lag screws and cerclage wires will be at the surgeon's discretion. In this pragmatic trial, the details of surgical incision and approach, fracture reduction, number and type of other screws and supplementary fixation with wires or screws will be at the surgeon's discretion as per their normal clinical practice.

2.5 Sample Size

Details of the sample size for primary outcomes and any co-primary or important key secondary outcomes (if applicable), including treatment effect, power, levels of statistical significance (one-tailed or two-tailed), clinical relevance and justification.

Data from the TrAFFix feasibility study will be used to calculate estimates of the standard deviation of the primary outcome measure (EQ-5D-5L) to drive a formal power analysis and sample size calculation for the

future definitive trial; evidence from other relevant sources reporting the metrics properties of EQ-5D-5L in the study population will also be used to inform this process. We anticipate that each of 6 participating centres will treat approximately 1.5 eligible patients per month. Taking a conservative approach, we would expect, to ensure feasibility, to be able to recruit 1.0 patients per month per centre. Given our schedule for centre opening times and recruitment length, we will have 52 centre months available to recruit for this study. Assuming that the recruitment rate is 1.0 per month per centre and monthly centre counts of patient recruitment numbers are approximately Poisson distributed and independent of one another, then this will allow us to estimate the recruitment rate with a 95% confidence interval of 0.73 - 1.28.[35] Therefore recruiting 52 patients in total should provide sufficiently precise estimates of the monthly recruitment rate to decide if a definitive trial is feasible.

2.6 Randomisation

The method(s) of randomisation, including the randomisation allocation ratio and any stratification/minimisation factors.

The unit of randomisation will be the individual patient. Randomisation will have a 1:1 allocation to two intervention groups (Nail and Plate) and be stratified by recruiting centre (6 centres will be used) and chronic cognitive impairment (Yes or No). Within each strata a fixed block size of 4 will be used for all blocks.

Full details of the randomisation are available in TrAFFix_RBP_v1.0_16Aug2016, stored in the confidential statistical section of the TMF.

2.7 Hypotheses and Definition of Primary and Secondary Outcomes

Clear description of hypotheses and definitions of the primary and secondary outcome measures and, when applicable, any methods used to enhance the quality of measurements (e.g., multiple observations, training of assessors). It might be useful to specify types of data that will be encountered in this trial.

The principle aim of this feasibility study will be to determine the number of eligible and recruited patients in the trauma centres over the course of 10 months. The primary outcome measures for this study are the participant recruitment rate and the completion rate of the EQ-5D-5L at 4 months post-surgery.

2.8 Outcomes Assessment Schedule

**Outcomes**	Baseline	6-weeks	4-months
EQ-5D-5L	Pre + post injury	X	X
DEMQoL[A]	Pre + post injury	X	X
DRI[B]	Pre + post injury	X	X
Radiographs	X	X	
Complications	X	X	X
Health Economics			X
Qualitative interviews[C]	X	X	X

2.9 Statistical Analysis Outline (references refer to protocol)

Main statistical analysis

This feasibility study is not powered to formally assess the size of the treatment effect, rather to estimate the recruitment rate. The totality of the data collected will be used to assess the feasibility of a definitive large RCT; recruitment rate being the driver of the feasibility study design on the basis that unless a reasonable recruitment rate can be achieved no formal trial will be possible. The recruitment rate will be estimated based on data collected and a (Poisson distributed) 95% confidence interval determined for this measure.

If the estimated recruitment rate is such that a definitive trial is feasible then no formal analysis will be undertaken and data from the feasibility study will be locked and carried over into the main (definitive) trial. No formal analysis of treatment efficacy will be undertaken in this scenario. However, the study ISDMC will

see unblinded summary statistics, together with recruitment data, and will advise the TSC with relevant safety or ethical guidance as the study progresses. The reasons and patterns of any missing data, loss to follow-up and participant withdrawals will be carefully considered and reported, with particular emphasis on how these may impinge on the future trial.

If a definitive trial is not feasible, then outcome data will reported in the conventional manner. Baseline demographics (e.g. Age, Gender, cognitive status) will be compared between groups to ensure approximate balance has been achieved. This is a small study (n=52), so treatment group effects are unlikely to be estimated with much precision and consequently inferences will be tentative and reported as such. The main analysis will investigate differences in the primary outcome measure, EQ-5D-5L[21] score at 4 months, between the two treatment groups (Nail and Plate) on an intention-to-treat basis. In addition a per-protocol analysis will also be reported and early EQ-5D-5L status will also be assessed and reported at 6 weeks. Differences between groups will based on a normal approximation for EQ-5D-5L.[20 31] Tests will be two-sided and considered to provide evidence for a significant difference if p-values are less than 0.05 (5% significance level). The stratified randomisation procedure should ensure a balance in cognitive impairment and recruiting centre between test treatments. Although generally we have no reason to expect that clustering effects will be important for this study, in reality the data will be hierarchical in nature, with patients naturally clustered into groups by recruiting centre. Therefore we will account for this by generalising the conventional linear (fixed-effects) regression approach to a mixed-effects modelling approach; where participants are naturally grouped by recruiting centres (random-effects). This model will formally incorporate terms that allow for possible heterogeneity in responses for patients due to the recruiting centre, in addition to the fixed effects of the treatment groups, cognitive impairment and age and gender, as these latter participant characteristics may prove to be important moderators of the treatment effect. The mixed-effects model will be the primary analysis, and will be reported as such.

The main analyses will be conducted using specialist mixed-effects modelling functions available in the software package R (http://www.r-project.org/) where EQ-5D-5L[21] data will be assumed to be normally distributed; possibly after appropriate variance-stabilising transformation. The primary focus will be the comparison of the two treatment groups of patients, and this will be reflected in the analysis which will be reported together with appropriate diagnostic plots that check the underlying model assumptions. Results will be presented as mean differences between the trial groups, with 95% confidence intervals.

Secondary analyses will be undertaken using the above strategy for other approximately normally distributed outcome measures such as DRI[23]. For dichotomous outcome variables, such as complications related to the trial interventions, mixed effects logistic regression analysis will be undertaken with results presented as odds ratios (and 95% confidence intervals) between the trial groups. The temporal patterns of any complications will be presented graphically and if appropriate a time-to-event analysis (Kaplan-Meier survival analysis) will be used to assess the overall risk and risk within individual classes of complications. The reasons and patterns of any missing data, loss to follow-up and participant withdrawals will also be carefully considered and reported.

A proportion of study participants will be unable to self-report EQ-5D, often due to dementia, so an appropriate individual (e.g. carer, relative) will be asked to proxy-report. The Dementia Quality of Life Measure (DEMQoL) is a validated questionnaire specifically designed to assess quality of life in patients with dementia. DEMQoL can be self or proxy-reported (28 or 31 items respectively). In order to assess the relative performance and merits of EQ-5D and DEMQoL in the study population, these two measures will be compared in the subgroup of study participants who are unable to self-report EQ-5D. Given the relatively small size of this study, and minority of participants who will be unable to self-report, there will not be enough data to undertake formal statistical testing for differences between measures. However, graphical presentation of the distribution and relationship (correlation) between measures for individuals will provide will allow some

assessment to be made of the merits and metric properties (e.g. variance) of the two measures. Agreement or moderate to strong correlation between measures will indicate that they are measuring the same underlying (latent) trait (which we take to be the true QoL of the study participants). Utilities will also be calculated for the two measures and compared in a similar manner.

Economic Evaluation

The feasibility of a future definitive economic evaluation of treatment with modern intramedullary nails or anatomical locking plates for fragility fractures of the distal femur will be investigated in this study. The analysis plan for the health economics evaluation will be detailed in a separate analysis plan.

Process evaluation

Qualitative data collected to inform the process evaluation from interviews with patients, surgeons and other staff will be transcribed verbatim. The analysis plan for the process evaluation will be detailed in a separate analysis plan.

3. QUALITY CONTROL AND DATA VALIDATION

Quality control and data validation will be carried out in accordance with OCTRU SOPs and TrAFFix monitoring and data management plans.

TrAFFix Monitoring Plan V1.0 21Jul2016:

Z:\KC_TRAFFIX\TRAFFIX eTMF V3.0\Risk assessment and Monitoring\2. Monitoring Plan

TrAFFix Data Management Plan V1.0 21Jul2016

Z:\KC_TRAFFIX\TRAFFIX eTMF V3.0\Data Management\Data Management Plan

3.1 Definition of Derived Data

Definitions of any data derived for analysis outlined here with full details stored in the Data and Sharing Management Plan (see OCTRU SOP DM-008 Data and Sharing Management Plan).

3.2 Validation of the Primary analysis

The primary outcome and key secondary outcomes will be analysed, following the analyses detailed in this SAP, by a statistician independent of the trial using different statistical software (if possible). Any discrepancies will be reported in the Statistical report (See OCTRU SOP STATS-005 Statistical Report).

4. INTERIM ANALYSIS AND DATA REVIEW

A brief summary of the format, remit and time points for meetings of the Data and Safety Monitoring Committee including any planned formal interim analyses.

The Data and Safety Monitoring Committee is independent from sponsor and follows the TrAFFix_DSMC_Charter_V1.0_02Sep2016. No interim analyses are planned.

5. SPECIFICATION OF STATISTICAL PACKAGES

All analysis will be carried out using appropriate validated statistical software such as STATA, SAS, SPLUS or R statistical software. The relevant package and version number will be recorded in the Statistical report.

6. DESCRIPTIVE ANALYSES

Summary of flow of trial participants through the trial and baseline stratification, demographic and clinical characteristics of each group.

6.1 Representativeness of Study Sample and Patient Throughput

The flow of participants through each stage of the trial, including numbers of participants randomly assigned, receiving intended treatment, completing the study protocol, and analysed for the primary outcome is provided following the appropriate guideline (e.g. CONSORT). Protocol violations/deviations and information relating to the screening data including the number of ineligible patients randomised, together with reasons.

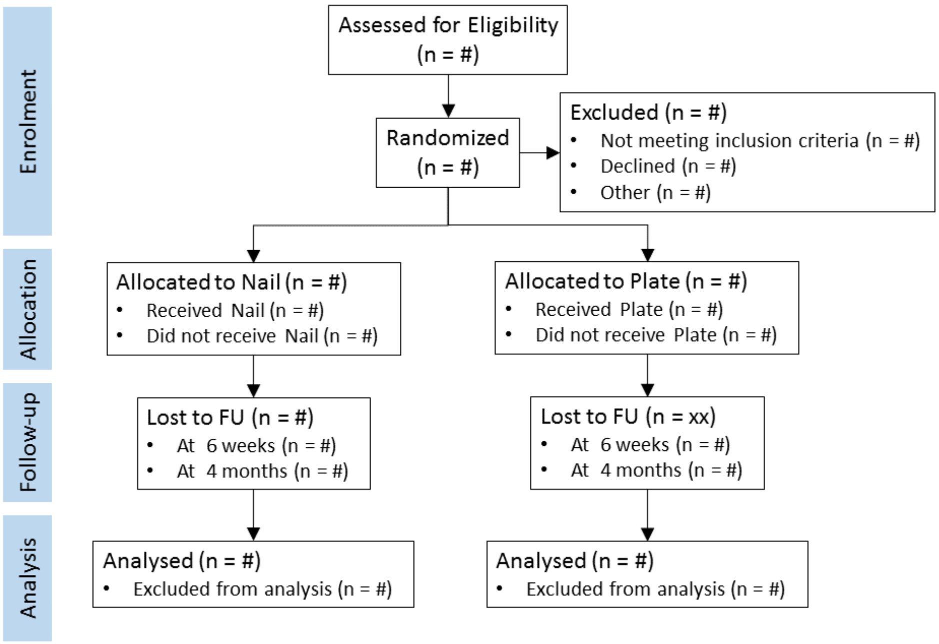

6.2 Baseline Comparability of Randomised Groups

Baseline characteristics are reported by treatment group, including the stratification/minimisation factors (if applicable) and important prognostic, demographic and clinical covariates).

Numbers (with percentages) for binary and categorical variables and means (and standard deviations), or medians (with lower and upper quartiles) for continuous variables will be presented; there will be no tests of statistical significance nor confidence intervals for differences between randomised groups on any baseline variable.

6.3 Comparison of Losses to Follow-up

Description of loss to follow-up together with reasons. Statistical tests comparing loss to follow-up to be undertaken and how these will be reported.

Loss to follow-up together with reasons will be reported by intervention arm. To assess differential losses between the groups this will be tested using absolute risk differences (95% confidence interval) and a chi-squared test. Any deaths (and their causes) will be reported separately.

6.4 Description of Available Data

The completeness of data required for primary and secondary outcomes is described, detailing methods to limit the possibility of missing data and methods for dealing with missing data when it occurs.

It seems likely that some data may not be available due to voluntary withdrawal of patients, lack of completion of individual data items or general loss to follow-up. Where possible the reasons for data missingness will be ascertained and reported. Although missing data are not expected to be a problem for this study, the nature

and pattern of the missingness will be carefully considered — including in particular whether data can be treated as missing completely at random (MCAR).

6.5 Description of Compliance with Intervention

Description of compliance with intervention – may include important covariates related to the interventional procedure or the standard treatment used in both arms.

A summary of the treatment received, as distinct from the treatment allocated, will be provided for each study participant. This is a pragmatic study, so detailed implementation of the study interventions will be left to the individual surgeons and will reflect their usual practice.

6.6 Unblinding of Randomised Treatments

Detail who is blinded in the study (participants, local site staff, outcome assessors). All cases of treatment unblinding will be listed, together with reasons and summarised (numbers, percentages)

As the surgical scars are clearly visible, the patients cannot be formally blinded to their treatment. Participants will only be informed of their treatment allocation at the end of the trial. In addition, the treating surgeons will also not be blind to the treatment, but will take no part in the post-operative assessment of the patients. The functional outcome data will be collected and entered onto the trial central database by a research assistant/data clerk in the trial central office.

6.7 Reliability

The radiographs collected will be reviewed by independent researchers at each hospital, A small sample of EQ-5D-5L utility scores will be checked by hand calculation to ensure that the computer algorithm has been implemented correctly.

7. DEFINITION OF POPULATIONS FOR ANALYSIS

Populations for analysis are defined as follows:

Intent to treat (ITT): all participants randomised in their randomised groups.

Per protocol (PP): all participants grouped by the intervention they received, rather than to which they were allocated. Participants receiving neither intervention will be excluded from the PP analysis

Safety: All participants who started the intervention or received a minimum amount of treatment as defined in the protocol.

8. ANALYSES TO ADDRESS PRIMARY AIMS

Statistical methods to be used to compare groups for primary outcome(s) and methods for point and interval estimation. Include methods for additional analyses, such as adjusted analyses and subgroup analyses, together with which populations will be analysed.

8.1 Evaluation/Definition of Primary Outcome (where applicable)

Criteria of evaluation and definition of outcomes and methods of calculation (rules for calculation of derived variables including definitions that can be programmed from the data).

Recruitment rate.

8.2 Statistical Methods Used for Analysis of Primary Outcome

Description of the primary and secondary/supplementary analyses and primary and secondary time-points (if applicable), including details of how results will be reported, both in tabular and graphical format.

The overall study recruitment rate will be estimated based on data collected and a 95% confidence interval determined for this measure. If the estimated recruitment rate is such that a definitive trial is feasible then no formal analysis will be undertaken and data from the feasibility study will be locked and carried over into the main (definitive) trial.

8.3 Adjustment of P values for Multiple Testing

Acknowledgement of the issue of multiple testing (if applicable), together with the rationale of the intention formally or informally adjust for multiplicity. If no adjustment planned this will be stated.

There is no multiple testing as only a single primary outcome is considered. Therefore significance levels used will be set at the conventional 5% level. Interim analyses of primary and secondary endpoints will not be carried out unless requested by the DSMC, who will formulate a plan for maintaining the overall study type I error rate if this is the case.

8.4 Missing Data

A description of methods utilised for dealing with missing, spurious (outliers) and unused data during statistical analysis including the type of missing data. If multiple imputation is to be used the methods will be specified. Methods for handling withdrawals and protocol deviations will be documented. State if no missing data adjustment will be used. Where multiple imputation is utilised the methods used to test the validity of the different assumptions will be described, either here or under the sensitivity analyses section.

A qualitative assessment will be made of the amount and pattern of any missing data and how this may affect the feasibility and or design of the main study.

8.5 Pre-specified Subgroup Analysis

Any pre-specified subgroup analyses will be described, together with the justification for relevance and importance and will include methods of analysis and presentation of the results. It is recommended that subgroup-treatment interaction methods are used with presentation using forest plots. This is usually included in the protocol, but additional subgroup analyses may be added before the final datalock following the blinded analysis or publication of other trials/research.

No subgroup analyses are planned

8.6 Treatment by Centre Interaction

Consistency of effect will be assessed across the 6 centres by informal examination of the within centre effects. There will be limited capacity to investigate these formally and it is noted that such centre effects are expected by chance.

8.7 Sensitivity Analysis

Describe any analysis utilised to check the robustness of the results. This may include repeating the primary analysis for different patient populations as defined in section 8.

No sensitivity analyses are planned.

Note: Sensitivity analyses for checking validity of multiple imputation assumptions should be detailed either here or in Section 9.4.

9. ANALYSIS TO ADDRESS SECONDARY AIMS

A list of the secondary outcomes will be provided with a separate section for each outcome with full definitions and analyses methods, together with details of the populations to be analysed and presentation of results will be provided. Sometimes the statistical method will be described first in full and then for each secondary outcome the method will be referred to in brief.

9.1 Evaluation/Definition of Secondary Outcomes (where applicable)

Not applicable.

9.2 Statistical Methods Used for Analysis of Secondary Outcomes

Statistical methods used to compare groups for secondary outcome(s); Methods for additional analyses, such as subgroup analyses and adjusted analyses.

The primary aim of this feasibility study is to assess the recruitment rate. Secondary outcomes will be collected;

Disability Rating Index (DRI), Dementia Quality of Life Measure (DEMQoL), EuroQol 5 Dimensions (5L) Score (EQ-5D-5L), self-efficacy, frailty, grip strength and complications. Methods for analysis of these variables are provided in Section 2.9. If a definitive trial is deemed feasible, then no analysis of these data will be undertaken and data will be carried over into the main study. However, if the main study is not deemed feasible, then outcome data will reported in the conventional manner.

9.1 Pre-specified Subgroups of Key Secondary Outcomes (where applicable)

Any pre-specified subgroup analyses of key secondary outcomes will be described, together with the justification for relevance and importance and will include methods of analysis, populations to be analysed and presentation of the results. This is usually included in the protocol, but additional subgroup analyses may be added before the final datalock following the blinded analysis or publication of other trials/research.

Not applicable.

9.2 Sensitivity Analysis of Key Secondary Outcomes (where applicable)

Describe any analysis utilised to check the robustness of the results for key secondary outcomes. See section 8.7.

Not applicable.

9.3 Health Economics and Cost Effectiveness

A summary from the protocol will be provided with reference to the relevant separate analysis plan (if applicable).

The statistician is not undertaking this analysis.

10. ADDITIONAL ANALYSES

10.1 Exploratory analyses

Pre-specified exploratory outcomes and analyses

Any outcomes and analyses pre-specified as exploratory in the protocol – detailed definition and analysis methodology will be provided or separate documents referred to together with who will perform these.

Additional Exploratory Analysis Not Specified Prior to Receiving Data

Any analyses not specified in the analysis protocol will be exploratory in nature and a significance level of 0.01 will be used to declare statistical significance. 99% confidence intervals will be presented.

10.2 Blinded analysis

Details of a blinded analysis will be undertaken prior to the final datalock will be provided, together with the rationale for undertaking this. If no blinded analysis to be undertaken, then state this.

No blinded analysis will be undertaken.

10.3 Meta-analyses (if applicable)

If meta-analysis is planned detail which trials will be included, which outcomes will be combined and whether random or fixed effects will be utilised. If this is under a separate protocol/analysis plan then reference this here.

Not applicable.

11. SAFETY ANALYSIS

Briefly outline the safety reporting from the protocol – this may be explicitly included as a secondary outcome – but include here. Describe how it will be summarised and compared (if applicable). Will adverse events/complications also be recorded, summarised and analysed – full details can be included here including dummy tables.

All complications and adverse events will be recorded. Complications will be classified as either: (a) unrelated to the trial protocol, (b) related systemic complications (including venous thromboembolic phenomena, death, pneumonia, urinary tract infection, blood transfusion, acute cerebrovascular incident, acute cardiac event, other) or (c) related local complications (superficial/deep infection, non/mal union, failure/removal/revision of metalwork, injury to adjacent structures such as nerves/tendons/blood vessels, other). The analysis will be conducted by intention to treat. Serious adverse events are defined as those that are fatal, life threatening, disabling or require hospitalisation or prolongation of hospitalisation.

12. APPENDIX: GLOSSARY OF ABBREVIATIONS

SAP	Statistical Analysis Plan
DSMC	Data and Safety Monitoring Committee
TSC	Trial Steering Committee
CI	Chief Investigator

13. DOCUMENT HISTORY

14. CHANGES FROM PREVIOUS VERSION OF SAP

A summary of key changes from earlier versions of SAP, with particular relevance to protocol changes that have an impact on the design, definition, sample size, data quality/collection and analysis of the outcomes will be provided. Include protocol version number and date.

Version number<br>Issue date	Author of this issue	Protocol Version & Issue date	Significant changes from previous version together with reasons
V1.0_19Sep2016	NP	Protocol_V2.0_27Jul2106	Not applicable as this is the 1st issue
V2.0_2Nov2016	NP	Protocol_V2.0_27Jul2106	Paragraph added to describe comparison between EQ-5D and DEMQoL
			Add to or delete as required
