# Supplementary material for: Intramedullary nails versus distal locking plates for fracture of the distal femur: results from the Trial of Acute Femoral Fracture Fixation (TrAFFix) randomised feasibility study and process evaluation
Source: BMJ Open. 2019 May 5;9(5):e026810. doi: 10.1136/bmjopen-2018-026810 (PMC6502051; doi:10.1136/bmjopen-2018-026810)
Supplement: Supplementary data [file bmjopen-2018-026810supp003.pdf]

Supplemental table 1: Reasons for non-inclusion and mechanisms of injury returned by participating hospitals. $The eligible age was changed from 50 to 18 during the study. Site names have been removed, *denotes major trauma centre.

	Site ID							
**Reason ineligible**	**A***	**B**	**C**	**D***	**E**	**F***	**G***	**Total**
Patient 50 or 18 years old$	3	0	2	6	0	4	3	**18**
Patient managed non-operatively	2	18	2	1	3	0	5	**31**
Pre-existing femoral deformity	1	0	1	0	0	0	0	**2**
Pre-existing arthroplasty	4	2	3	5	6	6	7	**33**
Other:	1	0	0	0	0	3	0	**4**
**Reason missed**								
Staffing/weekend	0	0	1	1	0	3	0	**5**
Technical (RRAMP fault)	0	0	0	0	1	0	0	**1**
Unknown (added from monitoring)	1	0	0	0	4	0	10	**15**
Surgeon preference	10	15	5	3	1	5	0	**39**
**Reason declined**								
Patient doesn't want to be part of research	0	0	1	0	0	0	0	**1**
Consultee doesn't want to be part of research	0	1	0	0	0	0	0	**1**
**Mechanism of Injury (of eligible patients)**								
Fall from <2m	15	5	6	6	9	5	9	**55**
Fall from >2m	1	0	0	0	0	2	0	**3**
Other	4	0	1	1	0	4	1	**11**
**Mechanism of Injury (of ineligible patients)**								
Fall from <2m	7	0	6	5	9	7	11	**45**
Fall from >2m	0	0	0	0	0	1	0	**1**
Other	3	0	2	7	0	5	4	**21**
Not reported	1	20	0	0	0	0	0	**21**
