# Supplementary material for: Intramedullary nails versus distal locking plates for fracture of the distal femur: results from the Trial of Acute Femoral Fracture Fixation (TrAFFix) randomised feasibility study and process evaluation
Source: BMJ Open. 2019 May 5;9(5):e026810. doi: 10.1136/bmjopen-2018-026810 (PMC6502051; doi:10.1136/bmjopen-2018-026810)
Supplement: Supplementary data [file bmjopen-2018-026810supp004.pdf]

Supplemental table 2: Overview of process evaluation findings

**Item**	**Component**	**Data Sources**	**Quantitative Findings**	**Qualitative findings**
Reach	• Number of eligible patients reached	• Screening logs<br>• Enhanced screening	• 23 of 85 eligible patients were included in the study<br>• 82 patients with distal femoral fractures were not recorded on the initial screening logs<br>• The number of distal femoral fractures missing from the screening logs varied from 0 to 34 between the centres	• Staff were confident that few patients were eligible for the study, suggesting that they were unaware that potentially eligible patients were being missed
Fidelity	• Procedure for screening patients<br>• Application of the study eligibility criteria	• Screening logs<br>• Enhanced screening<br>• Interviews with staff	• 82 patients who were admitted to the participating centres with a distal femoral fracture during the recruitment period were not included on the screening logs<br>• 39 eligible patients were excluded due to surgeon preference	• Clinical teams did not always notify the research teams of eligible patients<br>• In two centres, RAs did not attend the daily trauma meeting, where patient eligibility could be discussed<br>• Staff emphasised that there were fewer patients with distal femur fractures than they had expected and reported that many were ineligible<br>• Some staff felt that many surgeons had strong preferences for one of the two interventions and were unwilling to randomise patients<br>• These qualitative findings are linked to the themes i) making it work categories (category: research culture) and ii) knowing it's the right decision (category: making sense of the eligibility criteria)
	• Consent discussion	• Audio – recordings of patient staff interactions	• N/A	• When discussing the study with patients, RAs emphasised that both treatments are routinely used and that their surgeon was happy for them to receive either intervention<br>• They also avoided jargon and described the study in simple terms, using phrases such as '50/50', 'computer decides' and 'heads or tails' to explain randomisation

		• Interviews with RAs and participants		• These findings are linked to the theme: navigating research with patients after orthopaedic trauma (categories: making sense of the study and enabling participation)
	• Deviations from protocol/ application of operative procedures	• CRFs<br>• Interviews with staff	• Two protocol deviations were recorded. One patient allocated a plate received a nail and one patient allocated a nail received a plate	• Some staff described strategies that may help avoid cross overs in surgical trials. If the treating surgeon was uncomfortable or unwilling to perform one of the interventions then another surgeon could take that patient onto their operating list instead.<br>• These findings are linked to the themes i) making it work (category: research culture) and ii) knowing it's the right decision (category: clinical uncertainty)
	• Delivery of post-operative rehabilitation components<br>• Patient compliance with postoperative	• CRFs<br>• Interviews with participants and surgeons	• Eight patients were instructed to weight bear as tolerated, three were instructed to weight bear as required to allow mobilisation, seven partial weight bear and five non-weight bear.<br>• At six week follow-up, six of 15 participants who completed the questionnaire were weight bearing and nine were not.<br>• At four month follow-up, nine of the 14 participants who completed the questionnaire were weight bearing and five were not.	• Surgeons held different views about the most appropriate rehabilitation instructions for patients with this fracture but the majority favoured early weight bearing<br>• For participants, rehabilitation was typically slow and for some it was a frustrating process.<br>• Loss of confidence and pain hindered rehabilitation for some participants
Acceptability	• Acceptability of Interventions	• Screening logs<br>• Interviews with participants and staff	• 39 eligible patients were excluded due to surgeon preference	• Some staff felt that many surgeons had strong preferences for one of the two interventions and were unwilling to randomise patients<br>• Surgeons' unwillingness to randomise patients stemmed from their beliefs about whether both methods of fixation were appropriate for a patients' specific fracture and their own surgical skills

				• Participants tended to be less concerned about the nature of the interventions as long as their fracture was treated appropriately.<br>• The majority of participants recalled that the study involved a plate vs nail<br>• Staff understood that it would be difficult for patients to appreciate the differences between these two interventions and that that the type of metalwork used to fix their fracture was unlikely to be a priority for patients<br>• These findings are linked to the themes i) knowing it's the right decision (categories: surgeon preference and clinical equipoise), and ii) navigating research after orthopaedic trauma (category: making sense of the study)
	• Acceptability of study procedures	• Screening logs<br>• Interviews with participants and staff	• Two patients declined to participate in the study<br>• Two of seven patients who were entered in the study under nominated consultee consent declined when approached for retrospective consent to continue in the study after surgery<br>• One participant withdrew from the study after surgery	• RAs found that in the majority of cases patients were accepting of the nominated consultee consent procedure<br>• The majority of staff found randomisation to be acceptable to most patients<br>• When randomisation was described to participants during their interview, they typically seemed to be accepting of this procedure or indifferent towards it<br>• Several participants demonstrated a misunderstanding of randomisation at interview<br>• These findings link to the theme: navigating research with patients after orthopaedic trauma (categories: making sense of the study and enabling participation)
How is delivery achieved?	• Facilitators and barriers in the delivery of the study	• Interviews with staff	• N/A	• The overarching concept of facilitating trial recruitment was identified from our analysis of the interview data. This concept aids our understanding of how delivery was achieved by highlighting facilitators and barriers to delivering complex trauma trails such as TrAFFix.<br>• Themes comprising this concept are navigating research with patients after orthopaedic trauma, ii) knowing it's the right decision, and iii) making it work
