# Supplementary material for: Intramedullary nails versus distal locking plates for fracture of the distal femur: results from the Trial of Acute Femoral Fracture Fixation (TrAFFix) randomised feasibility study and process evaluation
Source: BMJ Open. 2019 May 5;9(5):e026810. doi: 10.1136/bmjopen-2018-026810 (PMC6502051; doi:10.1136/bmjopen-2018-026810)
Supplement: Supplementary data [file bmjopen-2018-026810supp005.pdf]

Supplemental table 3: Overview of process evaluation findings

Existing Factors	Injury and Treatment	Post-discharge factors
• Age<br>• Comorbidities<br>• Cognition<br>• Pre-injury mobility<br>• Pre-injury activity<br>• Nutrition<br>• Psychological factors (emotional resilience, self-efficacy, depression)	• Injury event<br>• Surgeon experience<br>• Surgeon preference<br>• Surgeon beliefs about rehabilitation<br>• Experience and care in hospital<br>• Communication – clear language<br>• Team work	• Transitions (e.g. from hospital to home)<br>• Appropriate discharge residence<br>• Follow-up<br>• Rehabilitation<br>• Pain<br>• Confidence<br>• Family (living arrangements, advocacy)<br>• Practical support<br>• Social support<br>• Changes to lifestyle after injury<br>• Loss of dignity<br>• Loss of independence<br>• Funding and access to social care<br>• Holistic approach to care<br>• Recovery pathways<br>• Balance of care between family and the system
