# Supplementary material for: Intramedullary nails versus distal locking plates for fracture of the distal femur: results from the Trial of Acute Femoral Fracture Fixation (TrAFFix) randomised feasibility study and process evaluation
Source: BMJ Open. 2019 May 5;9(5):e026810. doi: 10.1136/bmjopen-2018-026810 (PMC6502051; doi:10.1136/bmjopen-2018-026810)
Supplement: Author's manuscript [file bmjopen-2018-026810.draft_revisions.pdf]

BMJ Open

BMJ Open is committed to open peer review. As part of this commitment we make the peer review history of every article we publish publicly available.

When an article is published we post the peer reviewers' comments and the authors' responses online. We also post the versions of the paper that were used during peer review. These are the versions that the peer review comments apply to.

The versions of the paper that follow are the versions that were submitted during the peer review process. They are not the versions of record or the final published versions. They should not be cited or distributed as the published version of this manuscript.

BMJ Open is an open access journal and the full, final, typeset and author-corrected version of record of the manuscript is available on our site with no access controls, subscription charges or pay-per-view fees (http://bmjopen.bmj.com).

If you have any questions on BMJ Open's open peer review process please email editorial.bmjopen@bmj.com

BMJ Open

Intramedullary nails vs distal locking plates for fracture of the distal femur: results from the Trial of Acute Femoral Fracture Fixation (TrAFFix) randomised feasibility study and process evaluation

Journal:	*BMJ Open*
Manuscript ID	bmjopen-2018-026810
Article Type:	Research
Date Submitted by the Author:	20-Sep-2018

Complete List of Authors:	Griffin, Xavier; University of Oxford Nuffield Department of Orthopaedics Rheumatology and Musculoskeletal Sciences, Oxford Trauma; Oxford University Hospitals NHS Foundation Trust<br>Costa, Matthew; University of Oxford Nuffield Department of Orthopaedics Rheumatology and Musculoskeletal Sciences, Oxford Trauma; Oxford University Hospitals NHS Foundation Trust<br>Phelps, Emma; University of Oxford Nuffield Department of Orthopaedics Rheumatology and Musculoskeletal Sciences, Oxford Trauma<br>Parsons, Nicholas; University of Warwick, Warwick Medical School<br>Dritsaki, Melina; University of Oxford Nuffield Department of Orthopaedics Rheumatology and Musculoskeletal Sciences, Oxford Clinical Trials Research Unit<br>Achten, Juul; University of Oxford Nuffield Department of Orthopaedics Rheumatology and Musculoskeletal Sciences, Oxford Trauma<br>Tutton, Elizabeth; University of Warwick, Warwick Medical School; Oxford University Hospitals NHS Foundation Trust<br>Lerner, Robin; University of Oxford Nuffield Department of Orthopaedics Rheumatology and Musculoskeletal Sciences, Kadoorie Centre<br>McGibbon, Alwin; Patient representative<br>Baird, Janis; University of Southampton, MRC Epidemiology Resource Centre; University of Southampton and University Hospital Southampton NHS Foundation Trust, NIHR Southampton Biomedical Research Centre

Keywords:	Clinical trials < THERAPEUTICS, fractures, bone, fracture fixation, internal, randomized controlled trial, feasibility studies, femoral fractures

SCHOLARONE™
Manuscripts

TITLE

Intramedullary nails vs distal locking plates for fracture of the distal femur: results from the Trial of Acute Femoral Fracture Fixation (TrAFFix) randomised feasibility study and process evaluation

Authors

Xavier L Griffin[1,7*]

Matthew L Costa[1,7]

Emma Phelps[1]

Nicholas Parsons[5]

Melina Dritsaki[2]

Juul Achten[1]

Elizabeth Tutton[6,7]

Robin Lerner[1]

Alwin McGibbon[8]

Janis Baird[3,4]

on behalf of the TraFFix study collaborators

*corresponding author:

Email: xavier.griffin@ndorms.ox.ac.uk

Tel: +44 01865 223114

Address: Oxford Trauma, Kadoorie Centre, John Radcliffe Hospital, Oxford, OX3 9DU, UK

[1] Oxford Trauma, Nuffield Department of Orthopaedics, Rheumatology and Musculoskeletal Sciences (NDORMS), Kadoorie Centre, University of Oxford, Oxford, OX3 9DU, UK

[2] Oxford Clinical Trials Research Unit, NDORMS, University of Oxford, Windmill Road, Oxford, OX3 7LD, UK

[3] MRC Lifecourse Epidemiology Unit, Southampton General Hospital, University of Southampton, SO16 6YD, UK

[4] NIHR Southampton Biomedical Research Centre, University of Southampton and University Hospital Southampton NHS Foundation Trust, Southampton, UK

[5] Statistics and Epidemiology Unit, Warwick Medical School, University of Warwick, Coventry, CV4 7AL, UK

[6] University of Warwick, Warwick Research in Nursing, Warwick Medical School, Gibbet Hill Campus, Coventry CV4 7AL, UK

[7] Oxford University Hospitals NHS Foundation Trust, Trauma Research, Kadoorie Centre, John Radcliffe Hospital, Oxford OX3 9DU, UK

[8] Patient and public representative, Warwickshire, UK

Word count

4494/4000

For peer review only - http://bmjopen.bmj.com/site/about/guidelines.xhtml

For peer review only

ABSTRACT

Objectives: This feasibility study and process evaluation assessed the likely success of a definitive trial of intramedullary fixation with locked retrograde nails versus extramedullary fixation with fixed angle plates for fractures of the distal femur.

Design & setting: A multicentre, parallel, two-arm, randomised controlled feasibility study with an embedded process evaluation was conducted at seven NHS hospitals in England. Treatment was randomly allocated in 1:1 ratio, stratified by centre and chronic cognitive impairment. Participants, but not surgeons or research staff, were blinded to the allocation.

Participants: Patients 18 years and older with a fracture of the distal femur, who their surgeon believed would benefit from internal fixation, were eligible to take part.

Interventions: Patients were allocated to receive either a retrograde intramedullary nail or an anatomical locking plate.

Outcomes: The primary outcomes for this feasibility study were the recruitment rate and completion rate of the EQ-5D-5L at four months post-randomisation. Baseline characteristics, disability rating index, quality of life scores, measurements of social support and self-efficacy, resource use, and radiographic assessments were also collected. The views of patients and staff were collected during interviews.

Results: Recruitment and data completion were lower than expected. 23 of 82 eligible patients were recruited (nail; 11, plate; 12). The recruitment rate was estimated as 0.42 (95% CI; 0.27 to 0.62) participants per centre-month. Data completeness of the EQ-5D-5L at four-months was 61 percent (95% CI; 43 to 83%). The process evaluation demonstrated that the main barriers to recruitment were variation in treatment pathways across centres, lack of surgeon equipoise and confidence in using both interventions, and newly formed research cultures that lacked cohesion.

Conclusions: A modified trial design, with embedded recruitment support intervention, comparing functional outcome in cognitively intact adults who have sustained a fragility fracture of the distal femur is feasible.

Registration: ISRCTN92089567.

299/300 words

Strengths and limitations of this study:

- Mixed methods feasibility study including a randomised trial and process evaluation
- Conducted at seven hospitals, including several major trauma centres
- The study randomised only a small number of patients

Keywords: Femur; femoral fractures; fractures, bone; fracture fixation, internal; Randomized controlled trial; feasibility studies;

Disclaimer: This report presents independent research commissioned by the National Institute for Health Research (NIHR). The views and opinions expressed by the interviewees in this publication are those of the interviewees and do not necessarily reflect those of the authors, those of the NHS, the NIHR, MRC, CCF, NETSCC, the Health Technology Assessment programme or the Department of Health.

For peer review only - http://bmjopen.bmj.com/site/about/guidelines.xhtml

For peer review only

INTRODUCTION

Background

Distal femur fractures have an incidence of 10 per 100,000 and have a bimodal distribution with age, with approximately 85% sustained by older patients after a fall from a standing height and the remainder sustained by patients after major trauma. [1 2]

There is currently no consensus about the best way to treat these fractures. A recent Cochrane review identified few trials in this area, many using outdated implants or with important methodological limitations, and suggested that future patients' functional outcomes could be improved by a well-designed randomised controlled trial comparing modern treatments.[3]

In a multicentre retrospective study to review current practices, we found that the treatments commonly used in the UK are fixation with a locked retrograde nail (nail) and fixation with an anatomical, angular stable plate (plate).[4] Nails offer minimal disruption of the fracture site, a potential mechanical benefit from a device close to the axis of the femur, and stimulated blood supply from reaming of the intramedullary canal.[5] On the other hand fixed-angle plates are specifically designed for osteoporotic bone, and the bone-plate constructs have excellent biomechanical properties.[6] However plates require larger incisions and are more expensive than nails. Limited evidence suggests that there may be a benefit of using nails over plates in terms of patients' quality of life, with small studies identifying differences of 0.09 and 0.1 in EQ-5D-5L-derived utility scores at one year.[7 8]

This study of fracture fixation with nails compared with plates aimed to assess the feasibility of a future definitive trial, and perform a process evaluation to understand the generalisability and likely success of a future trial.

METHODS

Approval and oversight

The national research ethics service approved the study (ref: 16/WA/0225) and local approval was obtained from each of the recruiting sites. The trial protocol is available in the supplemental material and has been previously published.[9] The conduct of the study was overseen by independent data safety monitoring and trial steering committees.

Inclusion criteria

Patients aged 18 and over who were admitted to one of seven participating NHS hospitals in England with a fracture of the distal femur, which their treating surgeon believed would benefit from internal fixation were potentially eligible to take part. Patients with a loose knee or hip arthroplasty requiring revision, or an arthroplasty or pre-existing femoral deformity that precluded nail fixation, were excluded.

All potentially eligible participants for TrAFFix and their carers, as well as all staff involved with the study at participating centres, were eligible to be interviewed for the process evaluation.

Consent

Where patients had capacity to consent to be involved in the study prior to surgery, this was sought by hospital research teams. For patients lacking capacity to provide informed consent, the hospital research teams sought agreement from an appropriate consultee, either a nominated consultee (e.g. treating surgeon who was not part of the research team) or a personal consultee such as the next of kin. Where a consultee gave agreement prior to surgery and the patient regained capacity following surgery, consent for continued participation in the study was sought from the patient.

For peer review only - http://bmjopen.bmj.com/site/about/guidelines.xhtml
For peer review only

During the consent process patients and carers indicated their willingness to be approached for interviews for the process evaluation. Consent for interviews was sought at the start of each interview by a researcher from the University of Oxford.

Randomisation and blinding

Following consent from the patient or agreement from an appropriate consultee, participants were randomised prior to surgery to receive either nail or plate in a 1:1 ratio using an online randomisation system. The trial statistician generated the allocation sequences. Allocations used fixed blocks of size four and were stratified by centre to ensure any clustering effect related to centres was distributed across arms, and by the presence of chronic cognitive impairment to ensure this important effect modifier was distributed evenly across groups.

Participants were not informed of their allocation during the trial but were able to request to be informed of their allocation at the end of the study. Surgeons and research staff were not blinded. Radiographs were reviewed by independent assessors, however due to the presence of the implants they were also not blinded.

Sample size

There was no sample size calculation performed, instead it was estimated that recruiting for 52 centre-months with an average of one patient per centre-month would allow an estimation of the recruitment rate of a future definitive trial with a 95% confidence interval of 0.73-1.28.

Interventions

Potential participants were reviewed in daily trauma meetings and operated on at the next available theatre time. Patients were assessed and received anaesthesia, analgesia and prophylactic antibiotics as per local practices. Preparation, positioning and reduction of the fracture, details of incision and approach and supplementary fixation with wires or screws were left to the discretion of the treating surgeon.

For patients allocated a nail, fixation of the fracture was achieved with a proximally and distally locked nail that spanned the entire diaphysis of the femur. All nails were introduced retrograde through the knee joint. For patients allocated plates, fixation of the fracture was achieved with anatomical distal femoral locking-plate and screws, defined as those in which at least one fixed angle locking screw was placed distal to the fracture.

Outcome measures

The primary outcomes for this feasibility study were the recruitment rate and completion rate of the EQ-5D-5L[10] at four months post-surgery.

Screening

Participating hospitals were asked to record screening data for all patients admitted with a fracture of the distal femur during the study period. In addition, an orthopaedic surgeon at each participating hospital retrospectively reviewed admissions data and re-screened all admissions during the recruitment period. Similarly, we requested admissions data from the Trauma Audit Research Network (TARN)[11] in order to monitor the accuracy of screening data from research staff and orthopaedic trainees. These data included admission date, diagnosis and treatment received.

Clinical and patient-reported outcomes

Baseline recordings were made of patient characteristics, grip strength[12] and Rockwood frailty scores[13] as predictors of frailty, the level of social support available to patients using the medical outcomes survey (MOS) social support survey[14], and patient activation using a general self-efficacy questionnaire.[15]

For peer review only - http://bmjopen.bmj.com/site/about/guidelines.xhtml

EQ-5D-5L, a generic five-level health utility instrument used to measure health-related quality of life across five domains of mobility, self-care, usual activities, pain and depression was measured at baseline (pre-injury and contemporary) and 6-weeks and 4-months post-injury. Dementia quality of life scores (DEMQoL, and DEMQoL-proxy when completed by a carer) were recorded for patients with chronic cognitive impairment, along with proxy-reported EQ-5D-5L scores.[16] The disability rating index (DRI)[17], which measures disability from 0-100 across 12 items, was recorded at each time point for patients without cognitive impairment.

Radiographs taken at six-weeks post-injury were assessed independently for evidence of loss of fixation, varus or valgus deformity >5° (deformity in the coronal plane), recurvatum/procurvatum >10° (deformity in the sagittal plane), and shortening of the femur >1cm. Related medical complications occurring during the trial period were recorded.

In order to assess the feasibility of a health economic assessment in a larger trial, resource use data were measured using baseline CRFs (treatment costs) and a patient completed questionnaire at four months post-injury. Estimation of unit costs followed recent National Institute for Health and Care Excellence (NICE) guidelines on costing health and social care services[18] using the NHS reference costs[19] and NHS Supply Chain Catalogue[20] and inflated to 2017/18 prices using the NHS hospital & community health services (HCHS) index for health service resources.[21]

Analysis

Analyses were conducted in R (V3.3.0, R Foundation for Statistical Computing, Vienna, Austria. https://www.R-project.org/) and the statistical analysis plan is available as supplemental material. The recruitment rate was estimated using Poisson regression analysis, and the completeness of EQ-5D-5L was calculated as the percentage of randomised participants completing the EQ-5D-5L questionnaire. Given the small study group, treatment effects were not estimated.

Responses from the EQ-5D-5L health classifications were converted into an overall score using a published utility algorithm for the population of the United Kingdom.[22]

Process evaluation

A process evaluation was performed to assess implementation, mechanisms of impact and context of the interventions, in line with MRC guidelines.[23] In order to inform the development of a definitive trial, our process evaluation also examined the implementation of study processes. The evaluation of implementation included the reach and fidelity of screening and the acceptability of the interventions and study procedures. To understand how the interventions produced change in outcomes, we identified relevant intermediate outcomes that might be associated with the effect of the interventions on the primary outcomes of interest. The context in which the intervention is delivered will influence outcomes. A particular focus in our evaluation of context considered contextual similarities and differences between the participating centres, which might have influenced the delivery of study procedures.

We used a mixed methodology approach using a variety of data sources in the process evaluation including qualitative interviews with patients (n=9), carers (n=2) and staff (n=24), screening logs, national datasets and a one-day workshop with patients and public representatives. Interviews were based on a topic guide that included participants' experience of taking part in TrAFFix and the experiences of staff recruiting to TrAFFix. Interviews were audio recorded and transcribed verbatim; data were managed using NVivo 10 (QSR International, Warrington, UK) Data were analysed inductively using a thematic analysis. Quantitative data were summarised using standard descriptive statistical techniques. The analyses were integrated using the framework of a process evaluation and summarised in tables.

For peer review only - http://bmjopen.bmj.com/site/about/guidelines.xhtml

Patient & public involvement

A public representative was a member of the trial management committee, and was a co-applicant on the funding application. Patients and public representatives were consulted on the design of patient-facing documents via the UK Musculoskeletal Trauma PPI mailing list. Patient and public representatives were also invited to a meeting to develop a logic model.

Important changes to the study design

Patient's age (50 years and older) was initially used as an inclusion criterion as a surrogate for likely fragility fracture. However following review of the screening data, the independent steering committee requested that the minimum inclusion age be lowered to 18 and a mechanistic determination of fragility fracture, using mechanism of injury, be assessed as part of the feasibility study to explore the impact of this modification on recruitment rate.

Instead of utilising the algorithm by Herdman et al[24] to convert EQ-5D-5L to EQ-5D-3L utility as mentioned in the health economics analysis plan, the NICE-approved method of calculating utility values was performed using the algorithm provided by Van Hout et al.[22]

RESULTS

Recruitment & baseline

Recruitment opened in October 2016 and closed in August 2017, with a total of 54.8 centre-months of recruitment measured. Twenty three participants were randomised into the study, giving an estimated recruitment rate of 0.42 (95% CI: 0.27 to 0.62) participants per centre-month. The flow of participants through the trial is described in Figure 1.

Differences in surgical practices were identified across participating hospitals. For example the numbers of patients treated non-operatively varied between 0 and 18 patients (0% to 44% of patients screened at each centre), and the proportion of patients excluded due to surgeon preference varied between 40% and 70%. A breakdown of recruitment and reasons for exclusion for each site is given in supplemental Table S1.

Of the 173 of patients screened, the most common reason that patients were not recruited were surgeon preference for either a nail or plate (23%; 39/173), presence of a pre-existing arthroplasty (19%; 33/173) and a decision to treat the patient non-operatively (18%; 31/173). The number of patients treated non-operatively was higher than anticipated, but confirmed by data from retrospective screening of all admissions, and by data from TARN. The baseline characteristics of randomised participants in each group were well matched and are described in Table 1. Following the change of the minimum inclusion age to 18 years old, one patient under 50 was randomised.

Table 1 Baseline variables and treatment details

		**Nail (n=11)**	**Plate (n=12)**
Baseline variables			
Age (years); mean (sd; n)		70.1 (13.6; 10)	78.7 (14.9; 11)
Grip strength (kg); mean (sd; n)		16.8 (4.6; 4)	18.7 (1.2; 3)
Rockwood frailty score (1-9); mean (sd; n)		3.7 (2.2; 10)	4.5 (2.1; 12)
Pre-Op AMTS	< 7; n (%)	3 (27.3)	1 (8.3)
	7+; n (%)	4 (36.4)	7 (58.3)
Post-Op AMTS	< 7; n (%)	0 (0.0)	1 (8.3)
	7+; n (%)	2 (18.2)	3 (25.0)
Gender	Female; n (%)	7 (63.6)	9 (75)

For peer review only - http://bmjopen.bmj.com/site/about/guidelines.xhtml

	Male; n (%)	4 (36.4)	3 (25)
Ethnicity	Indian; n (%)	1 (9.1)	0 (0)
	White; n (%)	9 (81.8)	11 (91.7)
Diabetes	No; n (%)	5 (45.5)	9 (75)
	Yes; n (%)	5 (45.5)	2 (16.7)
Regular Smoker	No; n (%)	9 (81.8)	10 (83.3)
	Yes; n (%)	1 (9.1)	1 (8.3)
Living Arrangement	Care home; n (%)	1 (9.1)	1 (8.3)
	Live alone; n (%)	3 (27.3)	4 (33.3)
	Live with relatives; n (%)	1 (9.1)	1 (8.3)
	Live with wife/husband/ partner; n (%)	5 (45.5)	5 (41.7)
Treatment details			
Time from admission to surgery (days); median (IQR)		2 (1 to 3)	2 (1 to 3)
Mechanism of injury	Fall from <2m (%)	11 (100%)	12 (100%)
Fracture Classification*	A1; n (%)	7 (63.6)	5 (41.7)
	A2; n (%)	3 (27.3)	2 (16.7)
	A3; n (%)	0 (0)	1 (8.3)
	B1; n (%)	0 (0)	1 (8.3)
	C2; n (%)	1 (9.1)	1 (8.3)
	C3; n (%)	0 (0)	2 (16.7)
Periprosthetic Fracture	No; n (%)	8 (72.7)	9 (75)
	Yes; n (%)	3 (27.3)	3 (25)
ASA Grade	1 or 2; n (%)	3 (27.3)	4 (33.3)
	3 or 4; n (%)	8 (72.8)	6 (50)
Method Fixation	Nail; n (%)	10 (90.9)	1 (8.3)
	Plate; n (%)	0 (0)	11 (91.7)
	Other; n (%)	1 (9.1)	0 (0)
Grade Operating Surgeon	Consultant; n (%)	7 (63.6)	8 (66.7)
	SAS; n (%)	1 (9.1)	1 (8.3)
	ST3+; n (%)	3 (27.3)	3 (25)

Notes:
Where totals do not sum to column totals then it indicates that there were missing data
Reported percentages are based on the full population
* AO/OTA classification of peri-articular fractures

Clinical outcomes

Of the 23 participants randomised the EQ-5D-5L questionnaire was completed by 20 (87%, 95% CI: 65 to 97%), 15 (65%, 95% CI: 43 to 83%) and 14 (61%, 95% CI: 39 to 80%) patients at baseline, six weeks and four months respectively. There were insufficient numbers of patients to draw any meaningful conclusions about treatment effects between groups.

The mean scores across each time point for EQ-5D-5L, DRI and DEMQoL are reported in Table 2. Independent assessors at participating sites were asked to review any radiographs taken at 6-week follow-up appointments, the results of assessments are described in Table 3, along with a summary of the post-operative complications reported during the four-month follow-up period.

For peer review only - http://bmjopen.bmj.com/site/about/guidelines.xhtml

Table 2 Patient reported outcome measures

	Nail (n=11)	Plate (n=12)
Pre-injury: mean (sd; n)		
EQ-5D-5L	0.59 (0.29; 10)	0.56 (0.22; 10)
DRI	45.4 (36.7; 9)	67.5 (10.1; 10)
DEMQoL Proxy	87 (0; 1)	-
DEMQoL Self	-	78.2 (0; 1)
Post-injury: mean (sd; n)		
EQ-5D-5L	-0.05 (0.28; 10)	-0.04 (0.16; 10)
DRI	86.5 (15.5; 9)	92.5 (5.3; 10)
DEMQoL Proxy	97 (0; 1)	-
DEMQoL Self	-	84 (0, 1)
6-weeks: mean (sd; n)		
EQ-5D-5L	0.35 (0.30; 10)	0.05 (0.16; 5)
DRI	78.0 (16.9; 9)	87.3 (3.1; 5)
DEMQoL Proxy	87 (0; 1)	-
DEMQoL Self	-	-
4-months: mean (sd; n)		
EQ-5D-5L	0.38 (0.36; 9)	0.37 (0.41; 5)
DRI	60.9 (23.1; 8)	82.8 (2.9; 4)
DEMQoL Proxy	89 (0; 1)	-
DEMQoL Self	-	-

Note
- indicates no data were available

Table 3 Summary of 6-week post-operative radiograph review and reported complications reported up to 4-months

	Nail (n=11)	Plate (n=12)
6-week radiographic assessment: n (%)		
Loss of fixation	1 (9.1)	1 (8.3)
Varus/Valgus > 5°	10 (90.9)	5 (41.7)
Recurvatum > 10°	1 (9.1)	0 (0)
Procurvatum >10°	1 (9.1)	0 (0)
Shortening >1cm	2 (18.2)	0 (0)

Post-operative complications: n (%)	Nail	Plate
Wound infection	0 (0)	0 (0)
Venous thromboembolism	0 (0)	0 (0)
Pneumonia	1 (9.1)	0 (0)
Urinary tract infection	1 (9.1)	0 (0)
Cerebrovascular accident	0 (0)	0 (0)
Myocardial infarction	1 (9.1)	0 (0)
Blood transfusion	2 (18.2)	2 (16.7)
Mal-union	0 (0)	0 (0)
Failure of fixation	0 (0)	1 (8.3)

For peer review only

For peer review only - http://bmjopen.bmj.com/site/about/guidelines.xhtml

Process evaluation

A summary of the complete findings of the process evaluation is presented in the supplemental Table S2.

Implementation

Reach of screening

During the 10-month recruitment period, 91 patients were screened and recorded on screening logs, usually by a research associate (RA). Of these, 54 were eligible to participate in the study. Retrospective review of screening by orthopaedic surgeons at each participating centre found a further 82 unscreened patients with distal femur fractures including 31 eligible patients who were not included on the screening logs. The number of patients added after rescreening varied by centre with between 0 and 34 patients added. At interview, staff emphasised that there were few patients eligible for the study, suggesting that they were unaware that potentially eligible patients were being missed.

> *"With the TRAFFIX study, well we just never had the numbers coming through and I think the only two that we actually missed were during weekends and I think one was when I was on annual leave"*

Staff (RA) 19

The differing levels of experience of the research teams and research culture, evident in the approaches to screening, recruitment and data collection, within the centres may explain differences in the number of patients missed from screening logs. In some centres, not all surgeons within the team screened and identified eligible patients, while in other centres patients deemed ineligible might not have been recorded in the screening logs.

Fidelity of screening

Interviews with research staff revealed that several centres relied on research teams to screen patients as the clinical team tended not to notify the research team of eligible patients. Screening was facilitated by the presence of the research team in the daily trauma meeting, as they were able to prompt the clinical teams to consider whether patients were eligible. The attendance of the research team in this meeting varied between the centres due to staffing and other commitments. Additionally, some centres found it difficult for the clinical team to keep the study in mind as potentially eligible patients were infrequent.

Acceptability of the interventions

At interview, some staff described an unwillingness from surgeons to randomise patients. They felt that some surgeons lacked equipoise and believed that only one method of fixation was appropriate for any given fracture.

> *"you would never do the other thing for this fracture"*

Staff (Surgeon) 8

However, some staff reported that surgeons' preferences for one of the two interventions were based on their surgical skill and experience

> *"Some surgeons, they only know how to do a plate and what you will see is plate plate plate plate so it comes down to also what you are good at sometimes and you don't want something else."*

Staff (Surgeon) 16

For peer review only - http://bmjopen.bmj.com/site/about/guidelines.xhtml
For peer review only

In contrast some surgeons were able to accept community equipoise (uncertainty within the expert medical community)[25], accepting their less preferred intervention if required.

Acceptability of the study procedures

Consent

Participants and carers were rarely able to describe the study in their own words but they tended to recall or recognise the interventions.

> *"Yes, because one is on the outside and one is a rod through the middle isn't it"*

Participant 4

Several participants described struggling to engage with information about the study around the time of their surgery explaining that they were not "in a fit state" (participant 5) to ask questions or were "trying to so hard to be normal (participant 3)."

Interviews with staff showed that they described the study to patients in simple terms. Randomisation, for example was explained using phrases such as '50/50' or 'computer decides.' When explaining the study, staff also emphasised that both treatments are routinely used and that their surgeon was happy for them to receive either intervention, as they believed these were important to patients.

> *"They're going to have surgery anyway and then like I said if you say we don't know which the better one is and the surgeon is happy, I think is the key point is that the surgeon is happy for them to be part of the study"*

Staff (RA) 1

The majority of staff found involving relatives in the discussion about the study to be helpful as it enabled patients to be supported in their decision-making. In contrast, a minority of staff felt, that it was easier to consent patients to studies when their relatives were not involved as they could be protective and involving them could lead to more potential participants declining.

> *"Sometimes you can find it easier if you approach them to try and time it around their visiting hours and so they've got a relative with them and so you can kind of talk to them as a family and a lot more patients feel more comfortable with that"*

Staff (RA) 7

Two of seven patients who were entered into the study under nominated consultee agreement withdrew when they were approached to consent to continue in the study after surgery. One of these seven patients participated in an interview. She acknowledged that she "*wasn't sufficiently with it to give an opinion*" (participant 1) prior to surgery.

Randomisation

The majority of staff found randomisation was acceptable to patients, explaining that it was considered acceptable because of the explanation that the surgeon thought both treatments were appropriate. Two members of staff, in contrast, found patients disliked randomisation and wanted their surgeons to choose the treatment that was most appropriate for them.

When randomisation was described to participants during their interview, they accepted it or seemed indifferent towards it. Several participants, however, demonstrated therapeutic misconception: "*when a research subject fails to appreciate the distinction between the imperatives of clinical research and of ordinary treatment, and therefore inaccurately attributes therapeutic intent to research procedures*".[26] They believed that they or their relative would receive the most appropriate intervention for them.

For peer review only - http://bmjopen.bmj.com/site/about/guidelines.xhtml

> *"I think I prefer the one they done. I mean I didn't have a say in it, they decided what they thought was best, you know but I preferred the one they done definitely."*

Participant 6

Blinding

A minority of participants were told of their treatment allocation following surgery or when they returned for follow-up appointments. Some staff felt that blinding participants to their treatment allocation would be difficult as patients could see their allocation in the letter sent to their GP or X-rays, or they may be told by another member of staff. Some staff wanted to tell patients who asked which intervention they were allocated as one explained, "*I wouldn't be particularly comfortable, on a personal level... I would want to tell them what they have had*" (surgeon 8).

Case Reporting Forms (CRFs)

Research staff found that completing CRFs with the patient group could be challenging, as most patients were frail and often had long-term medical problems. They felt the questionnaires were lengthy and tiring for patients who often needed support completing them. They tended to ask the questions as part of a conversation to pull out the information for the questionnaire as they found some patients were unable to express their experience using the responses in the scales. Several RAs felt that where patients were wary of participating in studies this was usually due to the burden of follow-up rather than a dislike of randomisation or preference for one of the interventions.

> *"Some of the questions you have to try to put it into different words for them to understand, some of the questions don't make any sense to them"*

Staff (RA) 2

Mechanisms of impact

We identified intermediate outcomes that might be associated with the effect of the interventions on the primary outcomes of interest. Associations between the factors identified here, the interventions and the outcome of interest will assessed in a definitive trial. Factors that might influence patients' recovery after a distal femur fracture were identified by patients and public representatives and from data collected from interviews with participants and staff. Factors fell into three groups: existing factors relating to the characteristics of patients including their age, cognition and psychological factors such as self-efficacy; injury and treatment factors including the injury itself and factors relating to the surgeon and centre in which the treatment was delivered; post-discharge factors including rehabilitation provided, living arrangements and availability of support at home both from social care and from family. These factors are presented in supplemental Table S3.

Context

Contextual differences between the participating centres may have led to differences in how the study was implemented. Surgeons' working in departments where several trials were running may have been more engaged in research and more inclined to participate.

> *"However, that's changing and we've got a lot more of the consultants on board now and a lot more involved in the trial… there's consultants that are running trials in hand orthopaedics, the more elective side of things but also do the trauma lists and so are getting more involved with what's going on in research and therefore they're more happy to be involved in it and identifying the patients and things like that."*

Staff (RA) 4

Some staff felt that differences in the size of the research team, their workload and working hours could influence how the study was implemented. For example, the presence of RAs in the trauma

For peer review only - http://bmjopen.bmj.com/site/about/guidelines.xhtml

meeting could facilitate screening but required RAs to start work before eight, which was not possible in every centre.

> *I'm keen to see what is going to happen when we do get the other research nurses going to the trauma meeting because I feel that that's going to make a big difference and I think our recruitment will increase quite a lot but I might be completely wrong in thinking that.*
>
> **Staff (RA) 4**

> *I think if we didn't have enough studies running to have a fully funded research team then it would be tricky.*
>
> **Staff (surgeon) 20**

Meanwhile, some centres relied on one or part time RAs. This can result in patients being missed either when the RA was away or because they were struggling with their workload.

> *There was nobody in [to recruit] this Friday just gone and it can happen because I've got to be everywhere.*
>
> **Staff (RA) 16**

DISCUSSION

We have reported a randomised feasibility study, and to our knowledge the first with an embedded process evaluation, to plan a future trial of the treatment of patients with a fracture of the distal femur. We found the number of eligible patients screened closely matched the original estimate of 1.5 patients per centre-month. The recruitment rate into the trial, however, fell substantially short of this. The process evaluation identified very significant surgeon-related barriers to recruitment into the study. Research cultures were found to vary widely between centres; a positive culture was a strong predictor of improved recruitment performance.

This study has challenged many of the assumptions which underpinned the development of the trial protocol. The recruited population had a much lower prevalence of cognitive impairment, less frailty and was physiologically fitter than the previously characterised incident fracture population.[4] Surgeons were actively selecting fitter patients for operative treatment in a way that we had not anticipated. The eligible population is therefore somewhat different than we originally expected and hence the design for the definitive trial can be modified in important ways.

It is clear that research staff can and do miss potentially eligible patients if the working practices at hospitals do not facilitate full integration of the research staff into the clinical team. Although the specifics vary between hospitals, crucial decision points exist within the clinical process, such as daily trauma meetings, into which research staff must be integrated in order to be able to engage clinicians in research studies.

Surgeons reported a lack of their confidence in the management of this relatively uncommon fracture, the operative treatment of which is perceived to be technically demanding. The combination of this common anxiety and some individuals' lack of equipoise, posed a very significant barrier to recruitment into this study. Similar challenges have been previously identified in other surgical trials.[27 28] Interventions that have included training on community equipoise have been successful when targeted at multidisciplinary teams and surgeons, improving both clinicians' confidence and potentially recruitment rates,[29 30] though definitive evidence is lacking.[31] We propose that a modified protocol that includes an intervention to promote awareness and understanding of the existing community equipoise will be able to overcome these surgeon-related barriers to recruitment.

Our findings demonstrate that the trial design tested in this feasibility study is unlikely to be successful on a larger scale. However, the recruitment rate of 0.42 participants per centre-month is

For peer review only - http://bmjopen.bmj.com/site/about/guidelines.xhtml

in line with that reported by the FixDT trial, which tested similar interventions for treating fractures of the tibia and delivered to target and budget.[32]

We propose a modified definitive trial with a further internal pilot to confirm recruitment rates; this will be based upon the findings of this feasibility study, with an integrated recruitment intervention to support surgeons in their decision-making to recruit participants.

We conclude that a trial comparing functional outcome in cognitively intact patients aged 18 years or older who have sustained a fragility fracture of the distal femur, defined as a fall from standing height, treated with a nail or plate, is feasible with appropriate mitigation strategies incorporated to avoid failure.

SUPPLEMENTARY FILES

Study protocol

CONSORT checklist for pilot or feasibility studies

Statistical Analysis Plan

Supplementary tables 1-3

ACKNOWLEDGEMENTS

Contributions of authors

Xavier L Griffin (Associate Professor of Trauma Surgery) contributed to the conception, design, conduct and reporting of the study.

Matthew L Costa (Professor of Orthopaedic Trauma Surgery) contributed to the conception, design and reporting of the study.

Emma Phelps (Postdoctoral Research Associate in Mixed Methods Research) contributed to the design, conduct and analysis of the process evaluation and the reporting of the study.

Nicholas Parsons (Associate Professor of Medical Statistics, Statistics and Epidemiology) contributed to the design, conduct, analysis and reporting of the study.

Melina Dritsaki (Senior Health Economist) contributed to the design and conduct of the study.

Juul Achten (Scientific Officer Oxford Trauma) contributed to the conception, design, conduct and reporting of the study.

Elizabeth Tutton (Senior Research Fellow) contributed to the design, conduct and analysis of the process evaluation and the reporting of the study.

Robin Lerner (Clinical Trial Manager) contributed to the conduct and reporting of the study.

Alwin McGibbon (Patient & Public Representative) contributed to the design, conduct and reporting of the study.

Janis Baird (Professor of Public Health and Epidemiology) contributed to the conception, design, conduct, reporting and analysis of the study.

Principal collaborators

Mr David Noyes, John Radcliffe Hospital, Oxford University Hospitals NHS Foundation Trust

Prof Peter Giannoudis, Leeds General Infirmary, The Leeds Teaching Hospitals NHS Trust

Prof Ben Ollivere, Queen's Medical Centre, Nottingham University Hospitals NHS Trust

For peer review only - http://bmjopen.bmj.com/site/about/guidelines.xhtml

For peer review only

Ms Charlotte Lewis, Queen Alexandra Hospital, Portsmouth Hospitals NHS Trust

Mr Andrew McAndrew, Royal Berkshire Hospital, Royal Berkshire NHS Foundation Trust

Mr Haroon Majeed, Mr Damian McClelland, Royal Stoke Hospital, University Hospitals of North Midlands NHS Trust

Mr Ashwin Kulkarni, Leicester Royal Infirmary, University Hospitals of Leicester NHS Trust

Funding

This project was funded by the Health Technology Assessment programme (HTA) (project number 15/59/22) and will be published in full in the NIHR HTA Journal. Further information available at: https://www.journalslibrary.nihr.ac.uk/programmes/hta/155922/#/. This work was also supported by NIHR Oxford Biomedical Research Centre.

The funders had no role in the design and conduct of the study; collection, management, analysis, and interpretation of the data; preparation, review, or approval of the manuscript; and decision to submit the manuscript for publication.

Data sharing

All data requests should be submitted to the corresponding author for consideration. Please note exclusive use will be retained until the publication of major outputs. Access to anonymised data may be granted following review.

Competing interests

Matthew Costa is a member of the UK NIHR HTA General Board. Janis Baird received funding from Danone Nutrica Early Life Nutrition for a specific research study which aims to improve the nutrition and Vitamin D status of pregnant women and is collaborating with Iceland Foods Ltd to evaluate the impact fruit and vegetable availability on diet.

REFERENCES

1. Court-Brown CM, Caesar B. Epidemiology of adult fractures: A review. *Injury* 2006;37(8):691-7. doi: 10.1016/j.injury.2006.04.130
2. Martinet O, Cordey J, Harder Y, et al. The epidemiology of fractures of the distal femur. *Injury* 2000;31 Suppl 3:C62-3.
3. Griffin XL, Parsons N, Zbaeda MM, et al. Interventions for treating fractures of the distal femur in adults. *Cochrane Database Syst Rev* 2015;8:CD010606. doi: 10.1002/14651858.CD010606.pub2
4. Smith JRA HR MJ, Morrison R, Yip G, Aquilina A, Kelly M. Distal femoral fractures: The size of the problem in the UK. *Orthopaedic Trauma Society* 2013
5. Browner BD EC. The science and practice of intramedullary nailing. *Philadelphia* 1987;Lea & Febiger
6. Miller DL, Goswami T, Prayson MJ. Overview of the locking compression plate and its clinical applications in fracture healing. *J Surg Orthop Adv* 2008;17(4):271-81.
7. Tornetta P, Egol KA, Ertl JP, et al. Locked plating versus retrograde nailing for distal femur fractures: A multicentre randomized trial (Abstract 39). Orthopaedic Trauma Association Annual Meeting. Phoenix, Arizona, USA, 2014.
8. Hoskins W, Sheehy R, Edwards ER, et al. Nails or plates for fracture of the distal femur? *The Bone & Joint Journal* 2016;98-B(6):846-50. doi: 10.1302/0301-620x.98b6.36826
9. Griffin XL, Costa ML, Achten J, et al. Trial of Acute Femoral Fracture Fixation (TrAFFix): study protocol for a randomised controlled feasibility trial. *Trials* 2017;18(1):538. doi: 10.1186/s13063-017-2265-0
10. Brooks R. EuroQol: the current state of play. *Health Policy* 1996;37(1):53-72.

For peer review only - http://bmjopen.bmj.com/site/about/guidelines.xhtml

11. Lecky F. Twenty-five years of the trauma audit and research network: a continuing evolution to drive improvement. *Emerg Med J* 2015;32(12):906-8. doi: 10.1136/emermed-2015-205460
12. Roberts HC, Denison HJ, Martin HJ, et al. A review of the measurement of grip strength in clinical and epidemiological studies: towards a standardised approach. *Age Ageing* 2011;40(4):423-9. doi: 10.1093/ageing/afr051
13. Rockwood K, Song X, MacKnight C, et al. A global clinical measure of fitness and frailty in elderly people. *CMAJ* 2005;173(5):489-95. doi: 10.1503/cmaj.050051
14. Sherbourne CD, Stewart AL. The MOS social support survey. *Soc Sci Med* 1991;32(6):705-14.
15. Schwarzer R, Bäßler J, Kwiatek P, et al. The Assessment of Optimistic Self-beliefs: Comparison of the German, Spanish, and Chinese Versions of the General Self-efficacy Scale. *Applied Psychology* 1997;46(1):69-88. doi: 10.1111/j.1464-0597.1997.tb01096.x
16. Hounsome N, Orrell M, Edwards RT. EQ-5D as a Quality of Life Measure in People with Dementia and Their Carers: Evidence and Key Issues. *Value in Health* 2011;14(2):390-99. doi: http://dx.doi.org/10.1016/j.jval.2010.08.002
17. Salen BA, Spangfort EV, Nygren AL, et al. The Disability Rating Index: an instrument for the assessment of disability in clinical settings. *J Clin Epidemiol* 1994;47(12):1423-35.
18. National Institute for Health and Care Excellence. Guide to the methods of technology appraisal. In: Excellence NIfHaC, ed.: National Institute for Health and Care Excellence, 2013.
19. Department of Health and Social Care. NHS Reference Costs 2015 to 2016: Department of Health and Social Care, 2016.
20. National Health Service. NHS Supply Chain Catalogue [Available from: https://my.supplychain.nhs.uk/catalogue accessed Mar 2018.
21. Curtis L, Burns A, Personal Social Services Research Unit. Unit Costs of Health and Social Care. *Personal Social Services Research Unit* 2016
22. van Hout B, Janssen MF, Feng YS, et al. Interim scoring for the EQ-5D-5L: mapping the EQ-5D-5L to EQ-5D-3L value sets. *Value Health* 2012;15(5):708-15. doi: 10.1016/j.jval.2012.02.008
23. Moore GF, Audrey S, Barker M, et al. Process evaluation of complex interventions: Medical Research Council guidance. *BMJ* 2015;350:h1258. doi: 10.1136/bmj.h1258
24. Herdman M, Gudex C, Lloyd A, et al. Development and preliminary testing of the new five-level version of EQ-5D (EQ-5D-5L). *Qual Life Res* 2011;20(10):1727-36. doi: 10.1007/s11136-011-9903-x
25. Freedman B. Equipoise and the ethics of clinical research. *N Engl J Med* 1987;317(3):141-5. doi: 10.1056/NEJM198707163170304
26. Lidz CW, Appelbaum PS. The therapeutic misconception: problems and solutions. *Med Care* 2002;40(9 Suppl):V55-63. doi: 10.1097/01.MLR.0000023956.25813.18
27. Donovan JL, Paramasivan S, de Salis I, et al. Clear obstacles and hidden challenges: understanding recruiter perspectives in six pragmatic randomised controlled trials. *Trials* 2014;15(1):5. doi: 10.1186/1745-6215-15-5
28. Donovan JL, de Salis I, Toerien M, et al. The intellectual challenges and emotional consequences of equipoise contributed to the fragility of recruitment in six randomized controlled trials. *Journal of Clinical Epidemiology* 2014;67(8):912-20. doi: https://doi.org/10.1016/j.jclinepi.2014.03.010
29. Realpe A, Adams A, Wall P, et al. A new simple six-step model to promote recruitment to RCTs was developed and successfully implemented. *Journal of Clinical Epidemiology* 2016;76:166-74. doi: 10.1016/j.jclinepi.2016.02.002
30. Donovan JL, Rooshenas L, Jepson M, et al. Optimising recruitment and informed consent in randomised controlled trials: the development and implementation of the Quintet Recruitment Intervention (QRI). *Trials* 2016;17:283. doi: 10.1186/s13063-016-1391-4
31. Townsend D, Mills N, Savović J, et al. A systematic review of training programmes for recruiters to randomised controlled trials. *Trials* 2015;16(1):432. doi: 10.1186/s13063-015-0908-6
32. Costa ML, Achten J, Hennings S, et al. Intramedullary nail fixation versus locking plate fixation for adults with a fracture of the distal tibia: the UK FixDT RCT. *Health Technol Assess* 2018;22(25):1-148. doi: 10.3310/hta22250

For peer review only - http://bmjopen.bmj.com/site/about/guidelines.xhtml

FIGURE LEGENDS

Figure 1: Summary of recruitment and data collection. [$]The eligible age was changed from 50 to 18 during the study.

For peer review only

For peer review only - http://bmjopen.bmj.com/site/about/guidelines.xhtml

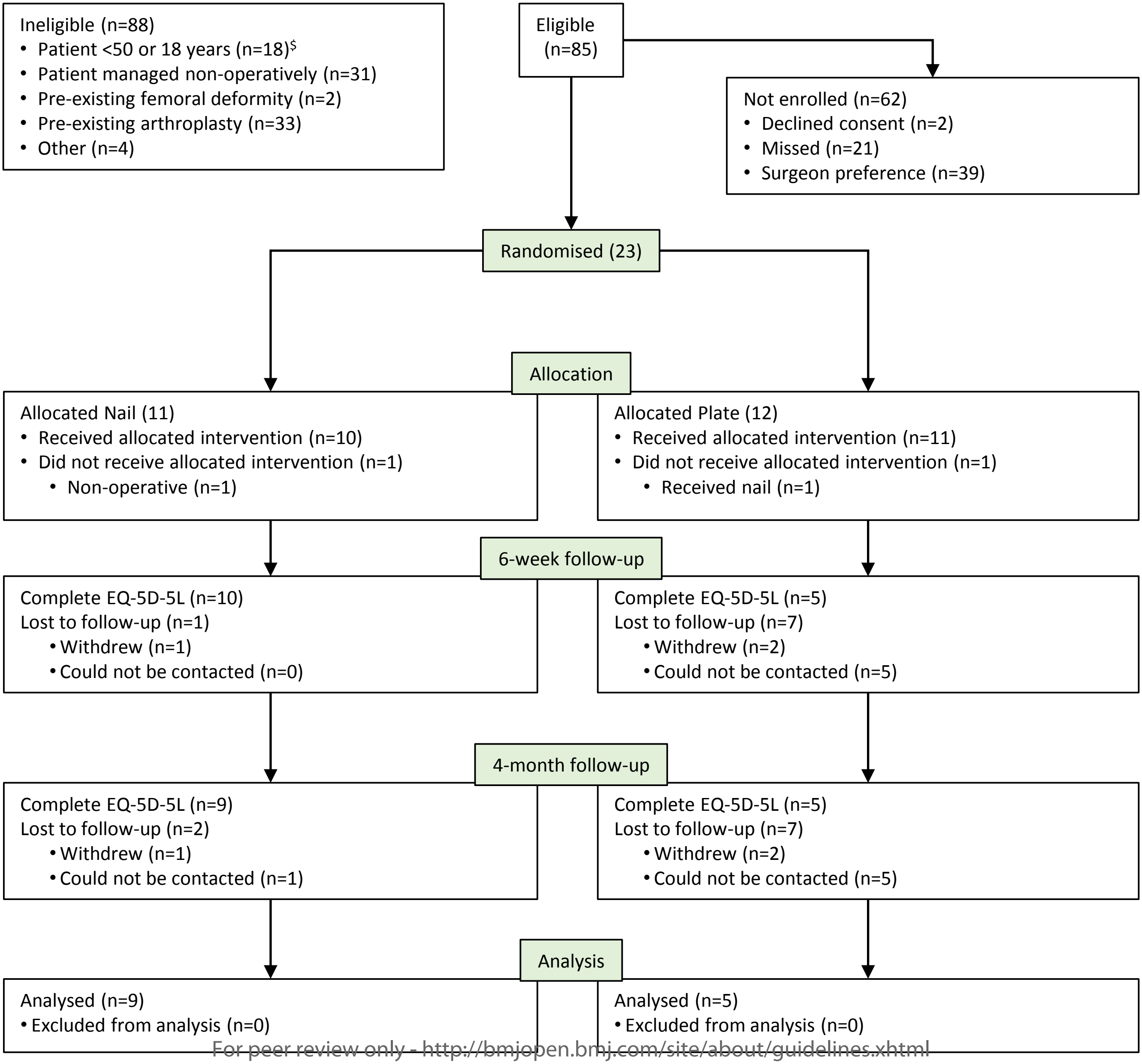

For peer review only - http://bmjopen.bmj.com/site/about/guidelines.xhtml

Supplemental table 1: Reasons for non-inclusion and mechanisms of injury returned by participating hospitals. $The eligible age was changed from 50 to 18 during the study. Site names have been removed, *denotes major trauma centre.

	Site ID							
**Reason ineligible**	**A***	**B**	**C**	**D***	**E**	**F***	**G***	**Total**
Patient 50 or 18 years old$	3	0	2	6	0	4	3	**18**
Patient managed non-operatively	2	18	2	1	3	0	5	**31**
Pre-existing femoral deformity	1	0	1	0	0	0	0	**2**
Pre-existing arthroplasty	4	2	3	5	6	6	7	**33**
Other:	1	0	0	0	0	3	0	**4**
**Reason missed**								
Staffing/weekend	0	0	1	1	0	3	0	**5**
Technical (RRAMP fault)	0	0	0	0	1	0	0	**1**
Unknown (added from monitoring)	1	0	0	0	4	0	10	**15**
Surgeon preference	10	15	5	3	1	5	0	**39**
**Reason declined**								
Patient doesn't want to be part of research	0	0	1	0	0	0	0	**1**
Consultee doesn't want to be part of research	0	1	0	0	0	0	0	**1**
**Mechanism of Injury (of eligible patients)**								
Fall from <2m	15	5	6	6	9	5	9	**55**
Fall from >2m	1	0	0	0	0	2	0	**3**
Other	4	0	1	1	0	4	1	**11**
**Mechanism of Injury (of ineligible patients)**								
Fall from <2m	7	0	6	5	9	7	11	**45**
Fall from >2m	0	0	0	0	0	1	0	**1**
Other	3	0	2	7	0	5	4	**21**
Not reported	1	20	0	0	0	0	0	**21**

For peer review only

Supplemental table 2: Overview of process evaluation findings

Item	Component	Data Sources	Quantitative Findings	Qualitative findings
Reach	• Number of eligible patients reached	• Screening logs<br>• Enhanced screening	• 23 of 85 eligible patients were included in the study<br>• 82 patients with distal femoral fractures were not recorded on the initial screening logs<br>• The number of distal femoral fractures missing from the screening logs varied from 0 to 34 between the centres	• Staff were confident that few patients were eligible for the study, suggesting that they were unaware that potentially eligible patients were being missed
Fidelity	• Procedure for screening patients<br>• Application of the study eligibility criteria	• Screening logs<br>• Enhanced screening<br>• Interviews with staff	• 82 patients who were admitted to the participating centres with a distal femoral fracture during the recruitment period were not included on the screening logs<br>• 39 eligible patients were excluded due to surgeon preference	• Clinical teams did not always notify the research teams of eligible patients<br>• In two centres, RAs did not attend the daily trauma meeting, where patient eligibility could be discussed<br>• Staff emphasised that there were fewer patients with distal femur fractures than they had expected and reported that many were ineligible<br>• Some staff felt that many surgeons had strong preferences for one of the two interventions and were unwilling to randomise patients<br>• These qualitative findings are linked to the themes i) making it work categories (category: research culture) and ii) knowing it's the right decision (category: making sense of the eligibility criteria)
	• Consent discussion	• Audio – recordings of patient staff interactions<br>• Interviews with RAs and	• N/A	• When discussing the study with patients, RAs emphasised that both treatments are routinely used and that their surgeon was happy for them to receive either intervention<br>• They also avoided jargon and described the study in simple terms, using phrases such as '50/50', 'computer decides' and 'heads or tails' to explain randomisation

For peer review only

		participants		• These findings are linked to the theme: navigating research with patients after orthopaedic trauma (categories: making sense of the study and enabling participation)
	• Deviations from protocol/ application of operative procedures	• CRFs<br>• Interviews with staff	• Two protocol deviations were recorded. One patient allocated a plate received a nail and one patient allocated a nail received a plate	• Some staff described strategies that may help avoid cross overs in surgical trials. If the treating surgeon was uncomfortable or unwilling to perform one of the interventions then another surgeon could take that patient onto their operating list instead.<br>• These findings are linked to the themes i) making it work (category: research culture) and ii) knowing it's the right decision (category: clinical uncertainty)
	• Delivery of post-operative rehabilitation components<br>• Patient compliance with postoperative	• CRFs<br>• Interviews with participants and surgeons	• Eight patients were instructed to weight bear as tolerated, three were instructed to weight bear as required to allow mobilisation, seven partial weight bear and five non-weight bear.<br>• At six week follow-up, six of 15 participants who completed the questionnaire were weight bearing and nine were not.<br>• At four month follow-up, nine of the 14 participants who completed the questionnaire were weight bearing and five were not.	• Surgeons held different views about the most appropriate rehabilitation instructions for patients with this fracture but the majority favoured early weight bearing<br>• For participants, rehabilitation was typically slow and for some it was a frustrating process.<br>• Loss of confidence and pain hindered rehabilitation for some participants
Acceptability	• Acceptability of Interventions	• Screening logs<br>• Interviews with participants	• 39 eligible patients were excluded due to surgeon preference	• Some staff felt that many surgeons had strong preferences for one of the two interventions and were unwilling to randomise patients<br>• Surgeons' unwillingness to randomise patients stemmed from their beliefs about whether both methods of fixation were appropriate for a patients'

For peer review only

		and staff		specific fracture and their own surgical skills<br>• Participants tended to be less concerned about the nature of the interventions as long as their fracture was treated appropriately.<br>• The majority of participants recalled that the study involved a plate vs nail<br>• Staff understood that it would be difficult for patients to appreciate the differences between these two interventions and that that the type of metalwork used to fix their fracture was unlikely to be a priority for patients<br>• These findings are linked to the themes i) knowing it's the right decision (categories: surgeon preference and clinical equipoise), and ii) navigating research after orthopaedic trauma (category: making sense of the study)
	• Acceptability of study procedures	• Screening logs<br>• Interviews with participants and staff	• Two patients declined to participate in the study<br>• Two of seven patients who were entered in the study under nominated consultee consent declined when approached for retrospective consent to continue in the study after surgery<br>• One participant withdrew from the study after surgery	• RAs found that in the majority of cases patients were accepting of the nominated consultee consent procedure<br>• The majority of staff found randomisation to be acceptable to most patients<br>• When randomisation was described to participants during their interview, they typically seemed to be accepting of this procedure or indifferent towards it<br>• Several participants demonstrated a misunderstanding of randomisation at interview<br>• These findings link to the theme: navigating research with patients after orthopaedic trauma (categories: making sense of the study and enabling participation)
How is delivery achieved?	• Facilitators and barriers in the delivery of the study	• Interviews with staff	• N/A	• The overarching concept of facilitating trial recruitment was identified from our analysis of the interview data. This concept aids our understanding of how delivery was achieved by highlighting facilitators and barriers to delivering complex trauma trails such as TrAFFix.<br>• Themes comprising this concept are navigating research with patients after orthopaedic trauma, ii) knowing it's the right decision, and iii) making it work

For peer review only

For peer review only

For peer review only - http://bmjopen.bmj.com/site/about/guidelines.xhtml

Supplemental table 3: Overview of process evaluation findings

Existing Factors	Injury and Treatment	Post-discharge factors
• Age<br>• Comorbidities<br>• Cognition<br>• Pre-injury mobility<br>• Pre-injury activity<br>• Nutrition<br>• Psychological factors (emotional resilience, self-efficacy, depression)	• Injury event<br>• Surgeon experience<br>• Surgeon preference<br>• Surgeon beliefs about rehabilitation<br>• Experience and care in hospital<br>• Communication – clear language<br>• Team work	• Transitions (e.g. from hospital to home)<br>• Appropriate discharge residence<br>• Follow-up<br>• Rehabilitation<br>• Pain<br>• Confidence<br>• Family (living arrangements, advocacy)<br>• Practical support<br>• Social support<br>• Changes to lifestyle after injury<br>• Loss of dignity<br>• Loss of independence<br>• Funding and access to social care<br>• Holistic approach to care<br>• Recovery pathways<br>• Balance of care between family and the system

For peer review only

TrAFFix

TrAFFix

Trial of Acute Femoral fracture Fixation

Statistical Analysis Plan

Version 2.0 – 2Nov2016

Based on Protocol version 2.0 – 27Jul2016

Name	Title	Role (Author/Reviewer/Approver)	Signature	Date
Nick Parsons	Trial Statistician	Author	N.R.Parsons	02Nov2016
Robin Lerner	Trial Manager	Reviewer		02Nov2016
Xavier Griffin	Chief Investigator	Reviewer	Xavier Griffin	02Nov2016

Oxford Clinical Trials Research Unit (OCTRU)
and

OCTRU
Oxford Clinical Trials Research Unit

WARWICK
THE UNIVERSITY OF WARWICK

Note: this document is version controlled and all draft versions and related communications should be stored in the Trial Master File

For peer review only
For peer review only - http://bmjopen.bmj.com/site/about/guidelines.xhtml

CONTENTS

1. INTRODUCTION ... 3
1.1 KEY PERSONNEL ... 3
2. BACKGROUND INFORMATION ... 3
2.1 RESEARCH HYPOTHESES AND STUDY OBJECTIVES ... 3
2.2 STUDY DESIGN AND TRIAL EXPECTED TIME-POINTS ... 3
2.3 ELIGIBILITY ... 4
2.4 TREATMENT INTERVENTIONS ... 4
2.5 SAMPLE SIZE ... 4
2.6 RANDOMISATION ... 5
2.7 HYPOTHESES AND DEFINITION OF PRIMARY AND SECONDARY OUTCOMES ... 5
2.8 OUTCOMES ASSESSMENT SCHEDULE ... 5
2.9 STATISTICAL ANALYSIS OUTLINE ... 5
3. QUALITY CONTROL AND DATA VALIDATION ... 7
3.1 DEFINITION OF DERIVED DATA ... 7
3.2 VALIDATION OF THE PRIMARY ANALYSIS ... 7
4. INTERIM ANALYSIS AND DATA REVIEW ... 7
5. SPECIFICATION OF STATISTICAL PACKAGES ... 7
6. DESCRIPTIVE ANALYSES ... 7
6.1 REPRESENTATIVENESS OF STUDY SAMPLE AND PATIENT THROUGHPUT ... 8
6.2 BASELINE COMPARABILITY OF RANDOMISED GROUPS ... 8
6.3 COMPARISON OF LOSSES TO FOLLOW-UP ... 8
6.4 DESCRIPTION OF AVAILABLE DATA ... 8
6.5 DESCRIPTION OF COMPLIANCE WITH INTERVENTION ... 9
6.6 UNBLINDING OF RANDOMISED TREATMENTS ... 9
6.7 RELIABILITY ... 9
7. DEFINITION OF POPULATIONS FOR ANALYSIS ... 9
8. ANALYSES TO ADDRESS PRIMARY AIMS ... 9
8.1 EVALUATION/DEFINITION OF PRIMARY OUTCOME (WHERE APPLICABLE) ... 9
8.2 STATISTICAL METHODS USED FOR ANALYSIS OF PRIMARY OUTCOME ... 9
8.3 ADJUSTMENT OF P VALUES FOR MULTIPLE TESTING ... 10
8.4 MISSING DATA ... 10
8.5 PRE-SPECIFIED SUBGROUP ANALYSIS ... 10
8.6 TREATMENT BY CENTRE INTERACTION ... 10
8.7 SENSITIVITY ANALYSIS ... 10
9. ANALYSIS TO ADDRESS SECONDARY AIMS ... 10
9.1 EVALUATION/DEFINITION OF SECONDARY OUTCOMES (WHERE APPLICABLE) ... 11
9.2 STATISTICAL METHODS USED FOR ANALYSIS OF SECONDARY OUTCOMES ... 11
9.1 PRE-SPECIFIED SUBGROUPS OF KEY SECONDARY OUTCOMES (WHERE APPLICABLE) ... 11
9.2 SENSITIVITY ANALYSIS OF KEY SECONDARY OUTCOMES (WHERE APPLICABLE) ... 11
9.3 HEALTH ECONOMICS AND COST EFFECTIVENESS ... 11
10. ADDITIONAL ANALYSES ... 11
10.1 EXPLORATORY ANALYSES ... 11
10.2 BLINDED ANALYSIS ... 11
10.3 META-ANALYSES (IF APPLICABLE) ... 12
11. SAFETY ANALYSIS ... 12

For peer review only - http://bmjopen.bmj.com/site/about/guidelines.xhtml

12. APPENDIX: GLOSSARY OF ABBREVIATIONS 12
13. DOCUMENT HISTORY 12
14. CHANGES FROM PREVIOUS VERSION OF SAP 12

1. INTRODUCTION

This document details the proposed presentation and analysis for the main paper(s) reporting results from the ***NIHR HTA funded Trial of Acute Femoral Fracture Fixation.*** The results reported in these papers should follow the strategy set out here. Subsequent analyses of a more exploratory nature will not be bound by this strategy, though they are expected to follow the broad principles laid down here. The principles are not intended to curtail exploratory analysis (for example, to decide cut-points for categorisation of continuous variables), nor to prohibit accepted practices (for example, data transformation prior to analysis), but they are intended to establish the rules that will be followed, as closely as possible, when analysing and reporting the trial.

The analysis strategy will be available on request when the principal papers are submitted for publication in a journal. Suggestions for subsequent analyses by journal editors or referees, will be considered carefully, and carried out as far as possible in line with the principles of this analysis strategy; if reported, the source of the suggestion will be acknowledged.

Any deviations from the statistical analysis plan will be described and justified in the final report of the trial. The analysis should be carried out by an identified, appropriately qualified and experienced statistician, who should ensure the integrity of the data during their processing. Examples of such procedures include quality control and evaluation procedures.

1.1 Key personnel

List of key people involved in the drafting and reviewing this SAP, together with their role in the trial and their contact details.

Author(s) (Trial statistician(s)):

Nick Parsons – Senior Statistician, Trial Statistician

Reviewers (Chief Investigator, Trial Manager, DSMC, TSC, Statistician as appropriate):

Xavier Griffin – Chief Investigator

Approver (Senior Statistician):

Nick Parsons – Senior Statistician, Trial Statistician

2. BACKGROUND INFORMATION

2.1 Research Hypotheses and Study Objectives

1. Assess the feasibility of a future definitive trial.
2. Perform a process evaluation to understand the generalisability and likely success of a future trial.
3. Explore the validity of self and proxy-reporting of the EQ-5D-5L in this specific population.

2.2 Study Design and trial expected time-points

The study design including the type of trial (e.g. parallel group, cluster, crossover, factorial, single arm), single/multi-centre, blinding, allocation ratio, trial framework (e.g. superiority, equivalence, non-inferiority, exploratory), primary outcome and crucial trial time-points.

For peer review only - http://bmjopen.bmj.com/site/about/guidelines.xhtml

TRAFFix is a randomized (1:1 allocation ratio) controlled multicentre trial with two (parallel) groups assessing fixation of the femur after fixation using either a nail or a plate.

Date of grant activation	01Jun2016
Date of start of recruitment:	XXAug2016
Date of expected end of recruitment:	31May2017
Date expected end follow-up:	30Sep2016
Date expected start of analysis:	01Oct2017
Date End of grant:	31Nov2017
Target number of subjects:	52
Participating Centres:	6

2.3 Eligibility

Inclusion criteria

Patients will be eligible for this study if they:

- are ≥50 years old as a surrogate for bone-density and therefore fragility fracture,
- have a fracture of the femur involving the distal two "Muller" squares,
- would, in the opinion of the attending surgeon, benefit from internal fixation of the fracture.

Exclusion criteria

Patients will be excluded from this trial if they have:

- a loose knee or hip arthroplasty requiring revision,
- pre-existing femoral deformity,
- an arthroplasty that precludes nail fixation.

2.4 Treatment Interventions

Intramedullary nailing: Fixation of the fracture will be achieved with a proximally and distally locked nail that spans the entire diaphysis of the femur. All nails will be introduced retrograde through the knee joint. In this pragmatic trial, the details of surgical incision and approach, fracture reduction and supplementary fixation with wires or screws will be at the surgeon's discretion as per their normal clinical practice.

Locking plate fixation: Fixation of the fracture will be achieved with anatomical distal femoral locking-plate and screws. Locking plates will be defined as those in which at least one fixed angle locking screw is placed distal to the fracture. The operating surgeon will determine the length, number and type of additional screws. Additional fixation with lag screws and cerclage wires will be at the surgeon's discretion. In this pragmatic trial, the details of surgical incision and approach, fracture reduction, number and type of other screws and supplementary fixation with wires or screws will be at the surgeon's discretion as per their normal clinical practice.

2.5 Sample Size

Details of the sample size for primary outcomes and any co-primary or important key secondary outcomes (if applicable), including treatment effect, power, levels of statistical significance (one-tailed or two-tailed), clinical relevance and justification.

Data from the TrAFFix feasibility study will be used to calculate estimates of the standard deviation of the primary outcome measure (EQ-5D-5L) to drive a formal power analysis and sample size calculation for the

For peer review only - http://bmjopen.bmj.com/site/about/guidelines.xhtml

future definitive trial; evidence from other relevant sources reporting the metrics properties of EQ-5D-5L in the study population will also be used to inform this process. We anticipate that each of 6 participating centres will treat approximately 1.5 eligible patients per month. Taking a conservative approach, we would expect, to ensure feasibility, to be able to recruit 1.0 patients per month per centre. Given our schedule for centre opening times and recruitment length, we will have 52 centre months available to recruit for this study. Assuming that the recruitment rate is 1.0 per month per centre and monthly centre counts of patient recruitment numbers are approximately Poisson distributed and independent of one another, then this will allow us to estimate the recruitment rate with a 95% confidence interval of 0.73 - 1.28.[35] Therefore recruiting 52 patients in total should provide sufficiently precise estimates of the monthly recruitment rate to decide if a definitive trial is feasible.

2.6 Randomisation

The method(s) of randomisation, including the randomisation allocation ratio and any stratification/minimisation factors.

The unit of randomisation will be the individual patient. Randomisation will have a 1:1 allocation to two intervention groups (Nail and Plate) and be stratified by recruiting centre (6 centres will be used) and chronic cognitive impairment (Yes or No). Within each strata a fixed block size of 4 will be used for all blocks.

Full details of the randomisation are available in TrAFFix_RBP_v1.0_16Aug2016, stored in the confidential statistical section of the TMF.

2.7 Hypotheses and Definition of Primary and Secondary Outcomes

Clear description of hypotheses and definitions of the primary and secondary outcome measures and, when applicable, any methods used to enhance the quality of measurements (e.g., multiple observations, training of assessors). It might be useful to specify types of data that will be encountered in this trial.

The principle aim of this feasibility study will be to determine the number of eligible and recruited patients in the trauma centres over the course of 10 months. The primary outcome measures for this study are the participant recruitment rate and the completion rate of the EQ-5D-5L at 4 months post-surgery.

2.8 Outcomes Assessment Schedule

**Outcomes**	Baseline	6-weeks	4-months
EQ-5D-5L	Pre + post injury	X	X
DEMQoL[A]	Pre + post injury	X	X
DRI[B]	Pre + post injury	X	X
Radiographs	X	X	
Complications	X	X	X
Health Economics			X
Qualitative interviews[C]	X	X	X

2.9 Statistical Analysis Outline (references refer to protocol)

Main statistical analysis

This feasibility study is not powered to formally assess the size of the treatment effect, rather to estimate the recruitment rate. The totality of the data collected will be used to assess the feasibility of a definitive large RCT; recruitment rate being the driver of the feasibility study design on the basis that unless a reasonable recruitment rate can be achieved no formal trial will be possible. The recruitment rate will be estimated based on data collected and a (Poisson distributed) 95% confidence interval determined for this measure.

If the estimated recruitment rate is such that a definitive trial is feasible then no formal analysis will be undertaken and data from the feasibility study will be locked and carried over into the main (definitive) trial. No formal analysis of treatment efficacy will be undertaken in this scenario. However, the study ISDMC will

For peer review only - http://bmjopen.bmj.com/site/about/guidelines.xhtml

see unblinded summary statistics, together with recruitment data, and will advise the TSC with relevant safety or ethical guidance as the study progresses. The reasons and patterns of any missing data, loss to follow-up and participant withdrawals will be carefully considered and reported, with particular emphasis on how these may impinge on the future trial.

If a definitive trial is not feasible, then outcome data will reported in the conventional manner. Baseline demographics (e.g. Age, Gender, cognitive status) will be compared between groups to ensure approximate balance has been achieved. This is a small study (n=52), so treatment group effects are unlikely to be estimated with much precision and consequently inferences will be tentative and reported as such. The main analysis will investigate differences in the primary outcome measure, EQ-5D-5L[21] score at 4 months, between the two treatment groups (Nail and Plate) on an intention-to-treat basis. In addition a per-protocol analysis will also be reported and early EQ-5D-5L status will also be assessed and reported at 6 weeks. Differences between groups will based on a normal approximation for EQ-5D-5L.[20 31] Tests will be two-sided and considered to provide evidence for a significant difference if p-values are less than 0.05 (5% significance level). The stratified randomisation procedure should ensure a balance in cognitive impairment and recruiting centre between test treatments. Although generally we have no reason to expect that clustering effects will be important for this study, in reality the data will be hierarchical in nature, with patients naturally clustered into groups by recruiting centre. Therefore we will account for this by generalising the conventional linear (fixed-effects) regression approach to a mixed-effects modelling approach; where participants are naturally grouped by recruiting centres (random-effects). This model will formally incorporate terms that allow for possible heterogeneity in responses for patients due to the recruiting centre, in addition to the fixed effects of the treatment groups, cognitive impairment and age and gender, as these latter participant characteristics may prove to be important moderators of the treatment effect. The mixed-effects model will be the primary analysis, and will be reported as such.

The main analyses will be conducted using specialist mixed-effects modelling functions available in the software package R (http://www.r-project.org/) where EQ-5D-5L[21] data will be assumed to be normally distributed; possibly after appropriate variance-stabilising transformation. The primary focus will be the comparison of the two treatment groups of patients, and this will be reflected in the analysis which will be reported together with appropriate diagnostic plots that check the underlying model assumptions. Results will be presented as mean differences between the trial groups, with 95% confidence intervals.

Secondary analyses will be undertaken using the above strategy for other approximately normally distributed outcome measures such as DRI[23]. For dichotomous outcome variables, such as complications related to the trial interventions, mixed effects logistic regression analysis will be undertaken with results presented as odds ratios (and 95% confidence intervals) between the trial groups. The temporal patterns of any complications will be presented graphically and if appropriate a time-to-event analysis (Kaplan-Meier survival analysis) will be used to assess the overall risk and risk within individual classes of complications. The reasons and patterns of any missing data, loss to follow-up and participant withdrawals will also be carefully considered and reported.

A proportion of study participants will be unable to self-report EQ-5D, often due to dementia, so an appropriate individual (e.g. carer, relative) will be asked to proxy-report. The Dementia Quality of Life Measure (DEMQoL) is a validated questionnaire specifically designed to assess quality of life in patients with dementia. DEMQoL can be self or proxy-reported (28 or 31 items respectively). In order to assess the relative performance and merits of EQ-5D and DEMQoL in the study population, these two measures will be compared in the subgroup of study participants who are unable to self-report EQ-5D. Given the relatively small size of this study, and minority of participants who will be unable to self-report, there will not be enough data to undertake formal statistical testing for differences between measures. However, graphical presentation of the distribution and relationship (correlation) between measures for individuals will provide will allow some

For peer review only - http://bmjopen.bmj.com/site/about/guidelines.xhtml
For peer review only

assessment to be made of the merits and metric properties (e.g. variance) of the two measures. Agreement or moderate to strong correlation between measures will indicate that they are measuring the same underlying (latent) trait (which we take to be the true QoL of the study participants). Utilities will also be calculated for the two measures and compared in a similar manner.

Economic Evaluation

The feasibility of a future definitive economic evaluation of treatment with modern intramedullary nails or anatomical locking plates for fragility fractures of the distal femur will be investigated in this study. The analysis plan for the health economics evaluation will be detailed in a separate analysis plan.

Process evaluation

Qualitative data collected to inform the process evaluation from interviews with patients, surgeons and other staff will be transcribed verbatim. The analysis plan for the process evaluation will be detailed in a separate analysis plan.

3. QUALITY CONTROL AND DATA VALIDATION

Quality control and data validation will be carried out in accordance with OCTRU SOPs and TrAFFix monitoring and data management plans.

TrAFFix Monitoring Plan V1.0 21Jul2016:

Z:\KC_TRAFFIX\TRAFFIX eTMF V3.0\Risk assessment and Monitoring\2. Monitoring Plan

TrAFFix Data Management Plan V1.0 21Jul2016

Z:\KC_TRAFFIX\TRAFFIX eTMF V3.0\Data Management\Data Management Plan

3.1 Definition of Derived Data

Definitions of any data derived for analysis outlined here with full details stored in the Data and Sharing Management Plan (see OCTRU SOP DM-008 Data and Sharing Management Plan).

3.2 Validation of the Primary analysis

The primary outcome and key secondary outcomes will be analysed, following the analyses detailed in this SAP, by a statistician independent of the trial using different statistical software (if possible). Any discrepancies will be reported in the Statistical report (See OCTRU SOP STATS-005 Statistical Report).

4. INTERIM ANALYSIS AND DATA REVIEW

A brief summary of the format, remit and time points for meetings of the Data and Safety Monitoring Committee including any planned formal interim analyses.

The Data and Safety Monitoring Committee is independent from sponsor and follows the TrAFFix_DSMC_Charter_V1.0_02Sep2016. No interim analyses are planned.

5. SPECIFICATION OF STATISTICAL PACKAGES

All analysis will be carried out using appropriate validated statistical software such as STATA, SAS, SPLUS or R statistical software. The relevant package and version number will be recorded in the Statistical report.

6. DESCRIPTIVE ANALYSES

Summary of flow of trial participants through the trial and baseline stratification, demographic and clinical characteristics of each group.

For peer review only - http://bmjopen.bmj.com/site/about/guidelines.xhtml

6.1 Representativeness of Study Sample and Patient Throughput

The flow of participants through each stage of the trial, including numbers of participants randomly assigned, receiving intended treatment, completing the study protocol, and analysed for the primary outcome is provided following the appropriate guideline (e.g. CONSORT). Protocol violations/deviations and information relating to the screening data including the number of ineligible patients randomised, together with reasons.

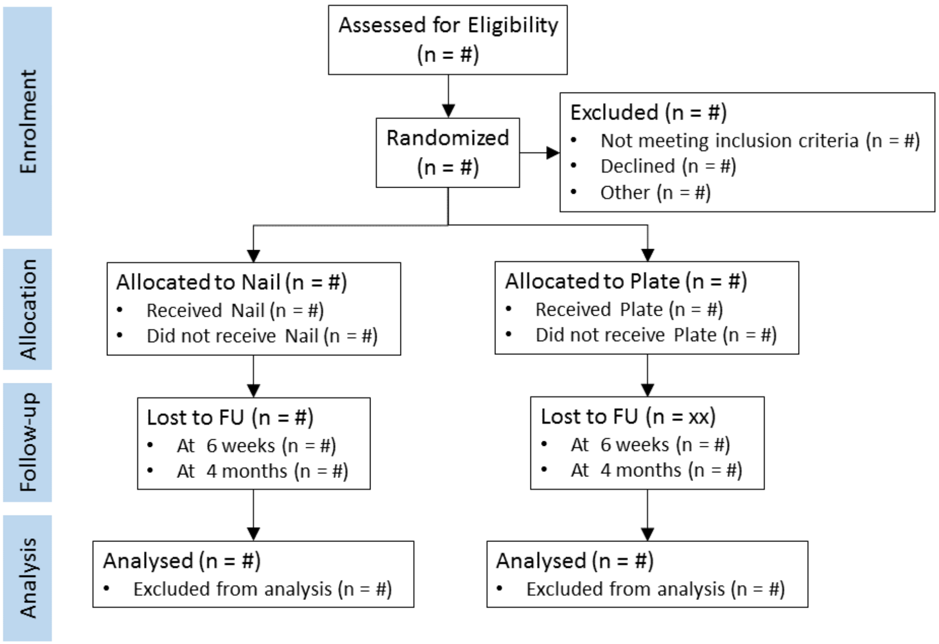

6.2 Baseline Comparability of Randomised Groups

Baseline characteristics are reported by treatment group, including the stratification/minimisation factors (if applicable) and important prognostic, demographic and clinical covariates).

Numbers (with percentages) for binary and categorical variables and means (and standard deviations), or medians (with lower and upper quartiles) for continuous variables will be presented; there will be no tests of statistical significance nor confidence intervals for differences between randomised groups on any baseline variable.

6.3 Comparison of Losses to Follow-up

Description of loss to follow-up together with reasons. Statistical tests comparing loss to follow-up to be undertaken and how these will be reported.

Loss to follow-up together with reasons will be reported by intervention arm. To assess differential losses between the groups this will be tested using absolute risk differences (95% confidence interval) and a chi-squared test. Any deaths (and their causes) will be reported separately.

6.4 Description of Available Data

The completeness of data required for primary and secondary outcomes is described, detailing methods to limit the possibility of missing data and methods for dealing with missing data when it occurs.

It seems likely that some data may not be available due to voluntary withdrawal of patients, lack of completion of individual data items or general loss to follow-up. Where possible the reasons for data missingness will be ascertained and reported. Although missing data are not expected to be a problem for this study, the nature

For peer review only - http://bmjopen.bmj.com/site/about/guidelines.xhtml

and pattern of the missingness will be carefully considered — including in particular whether data can be treated as missing completely at random (MCAR).

6.5 Description of Compliance with Intervention

Description of compliance with intervention – may include important covariates related to the interventional procedure or the standard treatment used in both arms.

A summary of the treatment received, as distinct from the treatment allocated, will be provided for each study participant. This is a pragmatic study, so detailed implementation of the study interventions will be left to the individual surgeons and will reflect their usual practice.

6.6 Unblinding of Randomised Treatments

Detail who is blinded in the study (participants, local site staff, outcome assessors). All cases of treatment unblinding will be listed, together with reasons and summarised (numbers, percentages)

As the surgical scars are clearly visible, the patients cannot be formally blinded to their treatment. Participants will only be informed of their treatment allocation at the end of the trial. In addition, the treating surgeons will also not be blind to the treatment, but will take no part in the post-operative assessment of the patients. The functional outcome data will be collected and entered onto the trial central database by a research assistant/data clerk in the trial central office.

6.7 Reliability

The radiographs collected will be reviewed by independent researchers at each hospital, A small sample of EQ-5D-5L utility scores will be checked by hand calculation to ensure that the computer algorithm has been implemented correctly.

7. DEFINITION OF POPULATIONS FOR ANALYSIS

Populations for analysis are defined as follows:

Intent to treat (ITT): all participants randomised in their randomised groups.

Per protocol (PP): all participants grouped by the intervention they received, rather than to which they were allocated. Participants receiving neither intervention will be excluded from the PP analysis

Safety: All participants who started the intervention or received a minimum amount of treatment as defined in the protocol.

8. ANALYSES TO ADDRESS PRIMARY AIMS

Statistical methods to be used to compare groups for primary outcome(s) and methods for point and interval estimation. Include methods for additional analyses, such as adjusted analyses and subgroup analyses, together with which populations will be analysed.

8.1 Evaluation/Definition of Primary Outcome (where applicable)

Criteria of evaluation and definition of outcomes and methods of calculation (rules for calculation of derived variables including definitions that can be programmed from the data).

Recruitment rate.

8.2 Statistical Methods Used for Analysis of Primary Outcome

Description of the primary and secondary/supplementary analyses and primary and secondary time-points (if applicable), including details of how results will be reported, both in tabular and graphical format.

For peer review only - http://bmjopen.bmj.com/site/about/guidelines.xhtml
For peer review only

The overall study recruitment rate will be estimated based on data collected and a 95% confidence interval determined for this measure. If the estimated recruitment rate is such that a definitive trial is feasible then no formal analysis will be undertaken and data from the feasibility study will be locked and carried over into the main (definitive) trial.

8.3 Adjustment of P values for Multiple Testing

Acknowledgement of the issue of multiple testing (if applicable), together with the rationale of the intention formally or informally adjust for multiplicity. If no adjustment planned this will be stated.

There is no multiple testing as only a single primary outcome is considered. Therefore significance levels used will be set at the conventional 5% level. Interim analyses of primary and secondary endpoints will not be carried out unless requested by the DSMC, who will formulate a plan for maintaining the overall study type I error rate if this is the case.

8.4 Missing Data

A description of methods utilised for dealing with missing, spurious (outliers) and unused data during statistical analysis including the type of missing data. If multiple imputation is to be used the methods will be specified. Methods for handling withdrawals and protocol deviations will be documented. State if no missing data adjustment will be used. Where multiple imputation is utilised the methods used to test the validity of the different assumptions will be described, either here or under the sensitivity analyses section.

A qualitative assessment will be made of the amount and pattern of any missing data and how this may affect the feasibility and or design of the main study.

8.5 Pre-specified Subgroup Analysis

Any pre-specified subgroup analyses will be described, together with the justification for relevance and importance and will include methods of analysis and presentation of the results. It is recommended that subgroup-treatment interaction methods are used with presentation using forest plots. This is usually included in the protocol, but additional subgroup analyses may be added before the final datalock following the blinded analysis or publication of other trials/research.

No subgroup analyses are planned

8.6 Treatment by Centre Interaction

Consistency of effect will be assessed across the 6 centres by informal examination of the within centre effects. There will be limited capacity to investigate these formally and it is noted that such centre effects are expected by chance.

8.7 Sensitivity Analysis

Describe any analysis utilised to check the robustness of the results. This may include repeating the primary analysis for different patient populations as defined in section 8.

No sensitivity analyses are planned.

Note: Sensitivity analyses for checking validity of multiple imputation assumptions should be detailed either here or in Section 9.4.

9. ANALYSIS TO ADDRESS SECONDARY AIMS

A list of the secondary outcomes will be provided with a separate section for each outcome with full definitions and analyses methods, together with details of the populations to be analysed and presentation of results will be provided. Sometimes the statistical method will be described first in full and then for each secondary outcome the method will be referred to in brief.

For peer review only - http://bmjopen.bmj.com/site/about/guidelines.xhtml

For peer review only

9.1 Evaluation/Definition of Secondary Outcomes (where applicable)

Not applicable.

9.2 Statistical Methods Used for Analysis of Secondary Outcomes

Statistical methods used to compare groups for secondary outcome(s); Methods for additional analyses, such as subgroup analyses and adjusted analyses.

The primary aim of this feasibility study is to assess the recruitment rate. Secondary outcomes will be collected;

Disability Rating Index (DRI), Dementia Quality of Life Measure (DEMQoL), EuroQol 5 Dimensions (5L) Score (EQ-5D-5L), self-efficacy, frailty, grip strength and complications. Methods for analysis of these variables are provided in Section 2.9. If a definitive trial is deemed feasible, then no analysis of these data will be undertaken and data will be carried over into the main study. However, if the main study is not deemed feasible, then outcome data will reported in the conventional manner.

9.1 Pre-specified Subgroups of Key Secondary Outcomes (where applicable)

Any pre-specified subgroup analyses of key secondary outcomes will be described, together with the justification for relevance and importance and will include methods of analysis, populations to be analysed and presentation of the results. This is usually included in the protocol, but additional subgroup analyses may be added before the final datalock following the blinded analysis or publication of other trials/research.

Not applicable.

9.2 Sensitivity Analysis of Key Secondary Outcomes (where applicable)

Describe any analysis utilised to check the robustness of the results for key secondary outcomes. See section 8.7.

Not applicable.

9.3 Health Economics and Cost Effectiveness

A summary from the protocol will be provided with reference to the relevant separate analysis plan (if applicable).

The statistician is not undertaking this analysis.

10. ADDITIONAL ANALYSES

10.1 Exploratory analyses

Pre-specified exploratory outcomes and analyses

Any outcomes and analyses pre-specified as exploratory in the protocol – detailed definition and analysis methodology will be provided or separate documents referred to together with who will perform these.

Additional Exploratory Analysis Not Specified Prior to Receiving Data

Any analyses not specified in the analysis protocol will be exploratory in nature and a significance level of 0.01 will be used to declare statistical significance. 99% confidence intervals will be presented.

10.2 Blinded analysis

Details of a blinded analysis will be undertaken prior to the final datalock will be provided, together with the rationale for undertaking this. If no blinded analysis to be undertaken, then state this.

No blinded analysis will be undertaken.

For peer review only - http://bmjopen.bmj.com/site/about/guidelines.xhtml

For peer review only

10.3 Meta-analyses (if applicable)

If meta-analysis is planned detail which trials will be included, which outcomes will be combined and whether random or fixed effects will be utilised. If this is under a separate protocol/analysis plan then reference this here.

Not applicable.

11. SAFETY ANALYSIS

Briefly outline the safety reporting from the protocol – this may be explicitly included as a secondary outcome – but include here. Describe how it will be summarised and compared (if applicable). Will adverse events/complications also be recorded, summarised and analysed – full details can be included here including dummy tables.

All complications and adverse events will be recorded. Complications will be classified as either: (a) unrelated to the trial protocol, (b) related systemic complications (including venous thromboembolic phenomena, death, pneumonia, urinary tract infection, blood transfusion, acute cerebrovascular incident, acute cardiac event, other) or (c) related local complications (superficial/deep infection, non/mal union, failure/removal/revision of metalwork, injury to adjacent structures such as nerves/tendons/blood vessels, other). The analysis will be conducted by intention to treat. Serious adverse events are defined as those that are fatal, life threatening, disabling or require hospitalisation or prolongation of hospitalisation.

For peer review only

12. APPENDIX: GLOSSARY OF ABBREVIATIONS

SAP Statistical Analysis Plan

DSMC Data and Safety Monitoring Committee

TSC Trial Steering Committee

CI Chief Investigator

13. DOCUMENT HISTORY

14. CHANGES FROM PREVIOUS VERSION OF SAP

A summary of key changes from earlier versions of SAP, with particular relevance to protocol changes that have an impact on the design, definition, sample size, data quality/collection and analysis of the outcomes will be provided. Include protocol version number and date.

Version number Issue date	Author of this issue	Protocol Version & Issue date	Significant changes from previous version together with reasons
V1.0_19Sep2016	NP	Protocol_V2.0_27Jul2106	Not applicable as this is the 1st issue
V2.0_2Nov2016	NP	Protocol_V2.0_27Jul2106	Paragraph added to describe comparison between EQ-5D and DEMQoL
			Add to or delete as required

For peer review only - http://bmjopen.bmj.com/site/about/guidelines.xhtml

CONSORT 2010 checklist of information to include when reporting a pilot or feasibility trial*

Section/Topic	Item No	Checklist item	Reported on page No
**Title and abstract**			
	1a	Identification as a pilot or feasibility randomised trial in the title	1
	1b	Structured summary of pilot trial design, methods, results, and conclusions (for specific guidance see CONSORT abstract extension for pilot trials)	2
**Introduction**			
Background and objectives	2a	Scientific background and explanation of rationale for future definitive trial, and reasons for randomised pilot trial	3
	2b	Specific objectives or research questions for pilot trial	2
**Methods**			
Trial design	3a	Description of pilot trial design (such as parallel, factorial) including allocation ratio	3-4
	3b	Important changes to methods after pilot trial commencement (such as eligibility criteria), with reasons	6
Participants	4a	Eligibility criteria for participants	3
	4b	Settings and locations where the data were collected	3
	4c	How participants were identified and consented	3
Interventions	5	The interventions for each group with sufficient details to allow replication, including how and when they were actually administered	4
Outcomes	6a	Completely defined prespecified assessments or measurements to address each pilot trial objective specified in 2b, including how and when they were assessed	4-5
	6b	Any changes to pilot trial assessments or measurements after the pilot trial commenced, with reasons	N/A
	6c	If applicable, prespecified criteria used to judge whether, or how, to proceed with future definitive trial	12
Sample size	7a	Rationale for numbers in the pilot trial	4
	7b	When applicable, explanation of any interim analyses and stopping guidelines	N/A
Randomisation:			
Sequence generation	8a	Method used to generate the random allocation sequence	4
	8b	Type of randomisation(s); details of any restriction (such as blocking and block size)	4
Allocation concealment mechanism	9	Mechanism used to implement the random allocation sequence (such as sequentially numbered containers), describing any steps taken to conceal the sequence until interventions were assigned	4

For peer review only

Implementation	10	Who generated the random allocation sequence, who enrolled participants, and who assigned participants to interventions	4
Blinding	11a	If done, who was blinded after assignment to interventions (for example, participants, care providers, those assessing outcomes) and how	4
	11b	If relevant, description of the similarity of interventions	4
Statistical methods	12	Methods used to address each pilot trial objective whether qualitative or quantitative	4-5
**Results**			
Participant flow (a diagram is strongly recommended)	13a	For each group, the numbers of participants who were approached and/or assessed for eligibility, randomly assigned, received intended treatment, and were assessed for each objective	6, fig 1
	13b	For each group, losses and exclusions after randomisation, together with reasons	6, fig 1
Recruitment	14a	Dates defining the periods of recruitment and follow-up	6
	14b	Why the pilot trial ended or was stopped	N/A
Baseline data	15	A table showing baseline demographic and clinical characteristics for each group	6-7
Numbers analysed	16	For each objective, number of participants (denominator) included in each analysis. If relevant, these numbers should be by randomised group	6
Outcomes and estimation	17	For each objective, results including expressions of uncertainty (such as 95% confidence interval) for any estimates. If relevant, these results should be by randomised group	6
Ancillary analyses	18	Results of any other analyses performed that could be used to inform the future definitive trial	6-12
Harms	19	All important harms or unintended effects in each group (for specific guidance see CONSORT for harms)	8
	19a	If relevant, other important unintended consequences	N/A
**Discussion**			
Limitations	20	Pilot trial limitations, addressing sources of potential bias and remaining uncertainty about feasibility	2, 12
Generalisability	21	Generalisability (applicability) of pilot trial methods and findings to future definitive trial and other studies	12
Interpretation	22	Interpretation consistent with pilot trial objectives and findings, balancing potential benefits and harms, and considering other relevant evidence	12
	22a	Implications for progression from pilot to future definitive trial, including any proposed amendments	12
**Other information**			
Registration	23	Registration number for pilot trial and name of trial registry	2
Protocol	24	Where the pilot trial protocol can be accessed, if available	9
Funding	25	Sources of funding and other support (such as supply of drugs), role of funders	2
	26	Ethical approval or approval by research review committee, confirmed with reference number	14

For peer review only

Citation: Eldridge SM, Chan CL, Campbell MJ, Bond CM, Hopewell S, Thabane L, et al. CONSORT 2010 statement: extension to randomised pilot and feasibility trials. BMJ. 2016;355.

*We strongly recommend reading this statement in conjunction with the CONSORT 2010, extension to randomised pilot and feasibility trials, Explanation and Elaboration for important clarifications on all the items. If relevant, we also recommend reading CONSORT extensions for cluster randomised trials, non-inferiority and equivalence trials, non-pharmacological treatments, herbal interventions, and pragmatic trials. Additional extensions are forthcoming: for those and for up to date references relevant to this checklist, see www.consort-statement.org.

For peer review only

BMJ Open

Intramedullary nails vs distal locking plates for fracture of the distal femur: results from the Trial of Acute Femoral Fracture Fixation (TrAFFix) randomised feasibility study and process evaluation

Journal:	*BMJ Open*
Manuscript ID	bmjopen-2018-026810.R1
Article Type:	Research
Date Submitted by the Author:	20-Feb-2019
Complete List of Authors:	Griffin, Xavier; University of Oxford Nuffield Department of Orthopaedics Rheumatology and Musculoskeletal Sciences, Oxford Trauma; Oxford University Hospitals NHS Foundation Trust<br>Costa, Matthew; University of Oxford Nuffield Department of Orthopaedics Rheumatology and Musculoskeletal Sciences, Oxford Trauma; Oxford University Hospitals NHS Foundation Trust<br>Phelps, Emma; University of Oxford Nuffield Department of Orthopaedics Rheumatology and Musculoskeletal Sciences, Oxford Trauma<br>Parsons, Nicholas; University of Warwick, Statistics and Epidemiology Unit<br>Dritsaki, Melina; University of Oxford Nuffield Department of Orthopaedics Rheumatology and Musculoskeletal Sciences, Oxford Clinical Trials Research Unit<br>Achten, Juul; University of Oxford Nuffield Department of Orthopaedics Rheumatology and Musculoskeletal Sciences, Oxford Trauma<br>Tutton, Elizabeth; University of Warwick, Warwick Medical School; Oxford University Hospitals NHS Foundation Trust<br>Lerner, Robin; University of Oxford Nuffield Department of Orthopaedics Rheumatology and Musculoskeletal Sciences, Kadoorie Centre<br>McGibbon, Alwin; Patient representative<br>Baird, Janis; University of Southampton, MRC Lifecourse Epidemiology Unit; University Hospital Southampton NHS Foundation Trust, NIHR Southampton Biomedical Research Centre
**Primary Subject Heading**:	Surgery
Secondary Subject Heading:	Qualitative research, Patient-centred medicine
Keywords:	Clinical trials < THERAPEUTICS, fractures, bone, fracture fixation, internal, randomized controlled trial, feasibility studies, femoral fractures

SCHOLARONE™
Manuscripts

For peer review only - http://bmjopen.bmj.com/site/about/guidelines.xhtml

TITLE

Intramedullary nails vs distal locking plates for fracture of the distal femur: results from the Trial of Acute Femoral Fracture Fixation (TrAFFix) randomised feasibility study and process evaluation

Authors

Xavier L Griffin[1,7*]

Matthew L Costa[1,7]

Emma Phelps[1]

Nicholas Parsons[5]

Melina Dritsaki[2]

Juul Achten[1]

Elizabeth Tutton[6,7]

Robin Lerner[1]

Alwin McGibbon[8]

Janis Baird[3,4]

on behalf of the TraFFix study collaborators

*corresponding author:

Email: xavier.griffin@ndorms.ox.ac.uk

Tel: +44 01865 223114

Address: Oxford Trauma, Kadoorie Centre, John Radcliffe Hospital, Oxford, OX3 9DU, UK

[1] Oxford Trauma, Nuffield Department of Orthopaedics, Rheumatology and Musculoskeletal Sciences (NDORMS), Kadoorie Centre, University of Oxford, Oxford, OX3 9DU, UK

[2] Oxford Clinical Trials Research Unit, NDORMS, University of Oxford, Windmill Road, Oxford, OX3 7LD, UK

[3] MRC Lifecourse Epidemiology Unit, Southampton General Hospital, University of Southampton, SO16 6YD, UK

[4] NIHR Southampton Biomedical Research Centre, University of Southampton and University Hospital Southampton NHS Foundation Trust, Southampton, UK

[5] Statistics and Epidemiology Unit, Warwick Medical School, University of Warwick, Coventry, CV4 7AL, UK

[6] University of Warwick, Warwick Research in Nursing, Warwick Medical School, Gibbet Hill Campus, Coventry CV4 7AL, UK

[7] Oxford University Hospitals NHS Foundation Trust, Trauma Research, Kadoorie Centre, John Radcliffe Hospital, Oxford OX3 9DU, UK

[8] Patient and public representative, Warwickshire, UK

Word count

4573/4000

For peer review only - http://bmjopen.bmj.com/site/about/guidelines.xhtml

ABSTRACT

Objectives: This feasibility study and process evaluation assessed the likely success of a definitive trial of intramedullary fixation with locked retrograde nails versus extramedullary fixation with fixed angle plates for fractures of the distal femur.

Design & setting: A multicentre, parallel, two-arm, randomised controlled feasibility study with an embedded process evaluation was conducted at seven NHS hospitals in England. Treatment was randomly allocated in 1:1 ratio, stratified by centre and chronic cognitive impairment. Participants, but not surgeons or research staff, were blinded to the allocation.

Participants: Patients 18 years and older with a fracture of the distal femur, who their surgeon believed would benefit from internal fixation, were eligible to take part.

Interventions: Patients were allocated to receive either a retrograde intramedullary nail or an anatomical locking plate.

Outcomes: The primary outcomes for this feasibility study were the recruitment rate and completion rate of the EQ-5D-5L at four months post-randomisation. Baseline characteristics, disability rating index, quality of life scores, measurements of social support and self-efficacy, resource use, and radiographic assessments were also collected. The views of patients and staff were collected during interviews.

Results: Recruitment and data completion were lower than expected. 23 of 82 eligible patients were recruited (nail; 11, plate; 12). The recruitment rate was estimated as 0.42 (95% CI; 0.27 to 0.62) participants per centre-month. Data completeness of the EQ-5D-5L at four-months was 61 percent (95% CI; 43 to 83%). The process evaluation demonstrated that the main barriers to recruitment were variation in treatment pathways across centres, lack of surgeon equipoise and confidence in using both interventions, and newly formed research cultures that lacked cohesion.

Conclusions: A modified trial design, with embedded recruitment support intervention, comparing functional outcome in cognitively intact adults who have sustained a fragility fracture of the distal femur is feasible.

Registration: ISRCTN92089567.

299/300 words

Strengths and limitations of this study:

- Mixed methods feasibility study including a randomised trial and process evaluation
- Conducted at seven hospitals, including several major trauma centres
- The study randomised only a small number of patients

Keywords: Femur; femoral fractures; fractures, bone; fracture fixation, internal; Randomized controlled trial; feasibility studies;

Disclaimer: This report presents independent research commissioned by the National Institute for Health Research (NIHR). The views and opinions expressed by the interviewees in this publication are those of the interviewees and do not necessarily reflect those of the authors, those of the NHS, the NIHR, MRC, CCF, NETSCC, the Health Technology Assessment programme or the Department of Health.

For peer review only

For peer review only - http://bmjopen.bmj.com/site/about/guidelines.xhtml

INTRODUCTION

Background

Distal femur fractures have an incidence of 10 per 100,000 and have a bimodal distribution with age, with approximately 85% sustained by older patients after a fall from a standing height and the remainder sustained by patients after major trauma. [1,2]

There is currently no consensus about the best way to treat these fractures. A recent Cochrane review identified few trials in this area, many using outdated implants or with important methodological limitations, and suggested that future patients' functional outcomes could be improved by a well-designed randomised controlled trial comparing modern treatments.[3]

In a multicentre retrospective study to review current practices, we found that the treatments commonly used in the UK are fixation with a locked retrograde nail (nail) and fixation with an anatomical, angular stable plate (plate).[4] Nails offer minimal disruption of the fracture site, a potential mechanical benefit from a device close to the axis of the femur, and stimulated blood supply from reaming of the intramedullary canal.[5] On the other hand fixed-angle plates are specifically designed for osteoporotic bone, and the bone-plate constructs have excellent biomechanical properties.[6] However plates require larger incisions and are more expensive than nails. Limited evidence suggests that there may be a benefit of using nails over plates in terms of patients' quality of life, with small studies identifying differences of 0.09 and 0.1 in EQ-5D-5L-derived utility scores at one year.[7,8]

This study of fracture fixation with nails compared with plates aimed to assess the feasibility of a future definitive trial, and perform a process evaluation to understand the generalisability and likely success of a future trial.

METHODS

Approval and oversight

The national research ethics service approved the study (ref: 16/WA/0225) and local approval was obtained from each of the recruiting sites. The trial protocol is available in the supplemental material and has been previously published.[9] The conduct of the study was overseen by independent data safety monitoring and trial steering committees.

Inclusion criteria

Patients aged 18 and over who were admitted to one of seven participating NHS hospitals in England with a fracture of the distal femur, which their treating surgeon believed would benefit from internal fixation were potentially eligible to take part. Patients with a loose knee or hip arthroplasty requiring revision, or an arthroplasty or pre-existing femoral deformity that precluded nail fixation, were excluded.

All potentially eligible participants for TrAFFix and their carers, as well as all staff involved with the study at participating centres, were eligible to be interviewed for the process evaluation.

Consent

Where patients had capacity to consent to be involved in the study prior to surgery, this was sought by hospital research teams. For patients lacking capacity to provide informed consent, the hospital research teams sought agreement from an appropriate consultee, either a nominated consultee (e.g. treating surgeon who was not part of the research team) or a personal consultee such as the next of kin. Where a consultee gave agreement prior to surgery and the patient regained capacity following surgery, consent for continued participation in the study was sought from the patient.

For peer review only - http://bmjopen.bmj.com/site/about/guidelines.xhtml

During the consent process patients and carers indicated their willingness to be approached for interviews for the process evaluation. Consent for interviews was sought at the start of each interview by a researcher from the University of Oxford.

Randomisation and blinding

Following consent from the patient or agreement from an appropriate consultee, participants were randomised prior to surgery to receive either nail or plate in a 1:1 ratio using an online randomisation system. The trial statistician generated the allocation sequences. Allocations used fixed blocks of size four and were stratified by centre to ensure any clustering effect related to centres was distributed across arms, and by the presence of chronic cognitive impairment to ensure this important effect modifier was distributed evenly across groups.

Participants were not informed of their allocation during the trial but were able to request to be informed of their allocation at the end of the study. Surgeons and research staff were not blinded. Radiographs were reviewed by independent assessors, however due to the presence of the implants they were also not blinded.

For peer review only

Sample size

There was no sample size calculation performed, instead it was estimated that recruiting for 52 centre-months with an average of one patient per centre-month would allow an estimation of the recruitment rate of a future definitive trial with a 95% confidence interval of 0.73-1.28.

Interventions

Potential participants were reviewed in daily trauma meetings and operated on at the next available theatre time. Patients were assessed and received anaesthesia, analgesia and prophylactic antibiotics as per local practices. Preparation, positioning and reduction of the fracture, details of incision and approach and supplementary fixation with wires or screws were left to the discretion of the treating surgeon.

For patients allocated a nail, fixation of the fracture was achieved with a proximally and distally locked nail that spanned the entire diaphysis of the femur. All nails were introduced retrograde through the knee joint. For patients allocated plates, fixation of the fracture was achieved with anatomical distal femoral locking-plate and screws, defined as those in which at least one fixed angle locking screw was placed distal to the fracture.

Outcome measures

The primary outcomes for this feasibility study were the recruitment rate and completion rate of the EQ-5D-5L[10] at four months post-surgery.

Screening

Participating hospitals were asked to record screening data for all patients admitted with a fracture of the distal femur during the study period. In addition, an orthopaedic surgeon at each participating hospital retrospectively reviewed admissions data and re-screened all admissions during the recruitment period. Similarly, we requested admissions data from the Trauma Audit Research Network (TARN)[11] in order to monitor the accuracy of screening data from research staff and orthopaedic trainees. These data included admission date, diagnosis and treatment received.

Clinical and patient-reported outcomes

Baseline recordings were made of patient characteristics, grip strength[12] and Rockwood frailty scores[13] as predictors of frailty, the level of social support available to patients using the medical outcomes survey (MOS) social support survey[14], and patient activation using a general self-efficacy questionnaire.[15]

For peer review only - http://bmjopen.bmj.com/site/about/guidelines.xhtml

EQ-5D-5L, a generic five-level health utility instrument used to measure health-related quality of life across five domains of mobility, self-care, usual activities, pain and depression was measured at baseline (pre-injury and contemporary) and 6-weeks and 4-months post-injury. Dementia quality of life scores (DEMQoL, and DEMQoL-proxy when completed by a carer) were recorded for patients with chronic cognitive impairment, along with proxy-reported EQ-5D-5L scores.[16] The disability rating index (DRI)[17], which measures disability from 0-100 across 12 items, was recorded at each time point for patients without cognitive impairment.

Radiographs taken at six-weeks post-injury were assessed independently for evidence of loss of fixation, varus or valgus deformity >5° (deformity in the coronal plane), recurvatum/procurvatum >10° (deformity in the sagittal plane), and shortening of the femur >1cm. Related medical complications occurring during the trial period were recorded.

In order to assess the feasibility of a health economic assessment in a larger trial, resource use data were measured using baseline CRFs (treatment costs) and a patient completed questionnaire at four months post-injury. Estimation of unit costs followed recent National Institute for Health and Care Excellence (NICE) guidelines on costing health and social care services[18] using the NHS reference costs[19] and NHS Supply Chain Catalogue[20] and inflated to 2017/18 prices using the NHS hospital & community health services (HCHS) index for health service resources.[21]

Analysis

Analyses were conducted in R (V3.3.0, R Foundation for Statistical Computing, Vienna, Austria. https://www.R-project.org/) and the statistical analysis plan is available as supplemental material. The recruitment rate was estimated using Poisson regression analysis, and the completeness of EQ-5D-5L was calculated as the percentage of randomised participants completing the EQ-5D-5L questionnaire. Given the small study group, treatment effects were not estimated.

Responses from the EQ-5D-5L health classifications were converted into an overall score using a published utility algorithm for the population of the United Kingdom.[22]

Process evaluation

A process evaluation was performed to assess implementation, mechanisms of impact and context of the interventions, in line with MRC guidelines.[23] In order to inform the development of a definitive trial, our process evaluation also examined the implementation of study processes. The evaluation of implementation included the reach and fidelity of screening and the acceptability of the interventions and study procedures. To understand how the interventions produced change in outcomes, we identified relevant intermediate outcomes that might be associated with the effect of the interventions on the primary outcomes of interest. The context in which the intervention is delivered will influence outcomes. A particular focus in our evaluation of context considered contextual similarities and differences between the participating centres, which might have influenced the delivery of study procedures.

We used a mixed methodology approach using a variety of data sources in the process evaluation including qualitative interviews with patients (n=9), carers (n=2) and staff (n=24), screening logs, national datasets and a one-day workshop with patients and public representatives. Interviews were based on a topic guide that included participants' experience of taking part in TrAFFix and the experiences of staff recruiting to TrAFFix. Interviews were audio recorded and transcribed verbatim; data were managed using NVivo 10 (QSR International, Warrington, UK) Data were analysed inductively using a thematic analysis. Quantitative data were summarised using standard descriptive statistical techniques. The analyses were integrated using the framework of a process evaluation and summarised in tables.

For peer review only - http://bmjopen.bmj.com/site/about/guidelines.xhtml

Patient & public involvement

A public representative was a member of the trial management committee, and was a co-applicant on the funding application. Patients and public representatives were consulted on the design of patient-facing documents via the UK Musculoskeletal Trauma PPI mailing list. Patient and public representatives were also invited to a meeting to develop a logic model.

Important changes to the study design

Patient's age (50 years and older) was initially used as an inclusion criterion as a surrogate for likely fragility fracture. However following review of the screening data, the independent steering committee requested that the minimum inclusion age be lowered to 18 and a mechanistic determination of fragility fracture, using mechanism of injury, be assessed as part of the feasibility study to explore the impact of this modification on recruitment rate.

Instead of utilising the algorithm by Herdman et al[24] to convert EQ-5D-5L to EQ-5D-3L utility as mentioned in the health economics analysis plan, the NICE-approved method of calculating utility values was performed using the algorithm provided by Van Hout et al.[22]

RESULTS

Recruitment & baseline

Recruitment opened in October 2016 and closed in August 2017, with a total of 54.8 centre-months of recruitment measured. Twenty three participants were randomised into the study, giving an estimated recruitment rate of 0.42 (95% CI: 0.27 to 0.62) participants per centre-month. The flow of participants through the trial is described in Figure 1.

Differences in surgical practices were identified across participating hospitals. For example the numbers of patients treated non-operatively varied between 0 and 18 patients (0% to 44% of patients screened at each centre), and the proportion of patients excluded due to surgeon preference varied between 40% and 70%. A breakdown of recruitment and reasons for exclusion for each site is given in supplemental Table S1.

Of the 173 of patients screened, the most common reason that patients were not recruited were surgeon preference for either a nail or plate (23%; 39/173), presence of a pre-existing arthroplasty (19%; 33/173) and a decision to treat the patient non-operatively (18%; 31/173). The number of patients treated non-operatively was higher than anticipated, but confirmed by data from retrospective screening of all admissions, and by data from TARN. The baseline characteristics of randomised participants in each group were well matched and are described in Table 1. Following the change of the minimum inclusion age to 18 years old one patient under 50 was randomised, this patient was aged 40-45 years, was injured by fall from over 2m, and had no reported bone health problems.

Table 1 Baseline variables and treatment details

		Nail (n=11)	Plate (n=12)
Baseline variables			
Age (years); mean (sd; n)		70.1 (13.6; 10)	78.7 (14.9; 11)
Grip strength (kg); mean (sd; n)		16.8 (4.6; 4)	18.7 (1.2; 3)
Rockwood frailty score (1-9); mean (sd; n)		3.7 (2.2; 10)	4.5 (2.1; 12)
Pre-Op AMTS	< 7; n (%)	3 (27.3)	1 (8.3)
	7+; n (%)	4 (36.4)	7 (58.3)
Post-Op AMTS	< 7; n (%)	0 (0.0)	1 (8.3)

For peer review only - http://bmjopen.bmj.com/site/about/guidelines.xhtml

	7+; n (%)	2 (18.2)	3 (25.0)
Gender	Female; n (%)	7 (63.6)	9 (75)
	Male; n (%)	4 (36.4)	3 (25)
Ethnicity	Indian; n (%)	1 (9.1)	0 (0)
	White; n (%)	9 (81.8)	11 (91.7)
Diabetes	No; n (%)	5 (45.5)	9 (75)
	Yes; n (%)	5 (45.5)	2 (16.7)
Regular Smoker	No; n (%)	9 (81.8)	10 (83.3)
	Yes; n (%)	1 (9.1)	1 (8.3)
Living Arrangement	Care home; n (%)	1 (9.1)	1 (8.3)
	Live alone; n (%)	3 (27.3)	4 (33.3)
	Live with relatives; n (%)	1 (9.1)	1 (8.3)
	Live with wife/husband/ partner; n (%)	5 (45.5)	5 (41.7)
Treatment details			
Time from admission to surgery (days); median (IQR)		2 (1 to 3)	2 (1 to 3)
Mechanism of injury	Fall from <2m (%)	11 (100%)	12 (100%)
Fracture Classification*	A1; n (%)	7 (63.6)	5 (41.7)
	A2; n (%)	3 (27.3)	2 (16.7)
	A3; n (%)	0 (0)	1 (8.3)
	B1; n (%)	0 (0)	1 (8.3)
	C2; n (%)	1 (9.1)	1 (8.3)
	C3; n (%)	0 (0)	2 (16.7)
Periprosthetic Fracture	No; n (%)	8 (72.7)	9 (75)
	Yes; n (%)	3 (27.3)	3 (25)
ASA Grade	1 or 2; n (%)	3 (27.3)	4 (33.3)
	3 or 4; n (%)	8 (72.8)	6 (50)
Method Fixation	Nail; n (%)	10 (90.9)	1 (8.3)
	Plate; n (%)	0 (0)	11 (91.7)
	Other; n (%)	1 (9.1)	0 (0)
Grade Operating Surgeon	Consultant; n (%)	7 (63.6)	8 (66.7)
	SAS; n (%)	1 (9.1)	1 (8.3)
	ST3+; n (%)	3 (27.3)	3 (25)

Notes:
Where totals do not sum to column totals then it indicates that there were missing data
Reported percentages are based on the full population
* AO/OTA classification of peri-articular fractures

Clinical outcomes

Of the 23 participants randomised the EQ-5D-5L questionnaire was completed by 20 (87%, 95% CI: 65 to 97%), 15 (65%, 95% CI: 43 to 83%) and 14 (61%, 95% CI: 39 to 80%) patients at baseline, six weeks and four months respectively. There were insufficient numbers of patients to draw any meaningful conclusions about treatment effects between groups.

The mean scores across each time point for EQ-5D-5L, DRI and DEMQoL are reported in Table 2. Independent assessors at participating sites were asked to review any radiographs taken at 6-week follow-up appointments, the results of assessments are described in Table 3, along with a summary of the post-operative complications reported during the four-month follow-up period.

For peer review only - http://bmjopen.bmj.com/site/about/guidelines.xhtml
For peer review only

Power analysis

We estimated the sample size necessary to power a definitive study using a patient reported outcome such as DRI. Using a minimum important difference of 8 points and standard deviation of 21 points defined in previous studies and in line with values reported here,[25] and an attrition rate of 30%, a sample of 210 participants per group (420 in total) would be needed to reject the null hypothesis with probability (power) 0.9 and type I error rate of 5%.

Table 2 Patient reported outcome measures

	Nail (n=11)	Plate (n=12)
Pre-injury: mean (sd; n)		
EQ-5D-5L	0.59 (0.29; 10)	0.56 (0.22; 10)
DRI	45.4 (36.7; 9)	67.5 (10.1; 10)
DEMQoL Proxy	87 (0; 1)	-
DEMQoL Self	-	78.2 (0; 1)
Post-injury: mean (sd; n)		
EQ-5D-5L	-0.05 (0.28; 10)	-0.04 (0.16; 10)
DRI	86.5 (15.5; 9)	92.5 (5.3; 10)
DEMQoL Proxy	97 (0; 1)	-
DEMQoL Self	-	84 (0, 1)
6-weeks: mean (sd; n)		
EQ-5D-5L	0.35 (0.30; 10)	0.05 (0.16; 5)
DRI	78.0 (16.9; 9)	87.3 (3.1; 5)
DEMQoL Proxy	87 (0; 1)	-
DEMQoL Self	-	-
4-months: mean (sd; n)		
EQ-5D-5L	0.38 (0.36; 9)	0.37 (0.41; 5)
DRI	60.9 (23.1; 8)	82.8 (2.9; 4)
DEMQoL Proxy	89 (0; 1)	-
DEMQoL Self	-	-

Note
- indicates no data were available

Table 3 Summary of 6-week post-operative radiograph review and reported complications reported up to 4-months

	Nail (n=11)	Plate (n=12)
6-week radiographic assessment: n (%)		
Loss of fixation	1 (9.1)	1 (8.3)
Varus/Valgus > 5°	10 (90.9)	5 (41.7)
Recurvatum > 10°	1 (9.1)	0 (0)
Procurvatum >10°	1 (9.1)	0 (0)
Shortening >1cm	2 (18.2)	0 (0)
Post-operative complications: n (%)	**Nail**	**Plate**
Wound infection	0 (0)	0 (0)
Venous thromboembolism	0 (0)	0 (0)
Pneumonia	1 (9.1)	0 (0)

For peer review only - http://bmjopen.bmj.com/site/about/guidelines.xhtml

Urinary tract infection	1 (9.1)	0 (0)
Cerebrovascular accident	0 (0)	0 (0)
Myocardial infarction	1 (9.1)	0 (0)
Blood transfusion	2 (18.2)	2 (16.7)
Mal-union	0 (0)	0 (0)
Failure of fixation	0 (0)	1 (8.3)

Process evaluation

A summary of the complete findings of the process evaluation is presented in the supplemental Table S2.

Implementation

Reach of screening

During the 10-month recruitment period, 91 patients were screened and recorded on screening logs, usually by a research associate (RA). Of these, 54 were eligible to participate in the study. Retrospective review of screening by orthopaedic surgeons at each participating centre found a further 82 unscreened patients with distal femur fractures including 31 eligible patients who were not included on the screening logs. The number of patients added after rescreening varied by centre with between 0 and 34 patients added. At interview, staff emphasised that there were few patients eligible for the study, suggesting that they were unaware that potentially eligible patients were being missed.

> *"With the TRAFFIX study, well we just never had the numbers coming through and I think the only two that we actually missed were during weekends and I think one was when I was on annual leave"*

Staff (RA) 19

The differing levels of experience of the research teams and research culture, evident in the approaches to screening, recruitment and data collection, within the centres may explain differences in the number of patients missed from screening logs. In some centres, not all surgeons within the team screened and identified eligible patients, while in other centres patients deemed ineligible might not have been recorded in the screening logs.

Fidelity of screening

Interviews with research staff revealed that several centres relied on research teams to screen patients as the clinical team tended not to notify the research team of eligible patients. Screening was facilitated by the presence of the research team in the daily trauma meeting, as they were able to prompt the clinical teams to consider whether patients were eligible. The attendance of the research team in this meeting varied between the centres due to staffing and other commitments. Additionally, some centres found it difficult for the clinical team to keep the study in mind as potentially eligible patients were infrequent.

Acceptability of the interventions

At interview, some staff described an unwillingness from surgeons to randomise patients. They felt that some surgeons lacked equipoise and believed that only one method of fixation was appropriate for any given fracture.

> *"you would never do the other thing for this fracture"*

Staff (Surgeon) 8

However, some staff reported that surgeons' preferences for one of the two interventions were based on their surgical skill and experience

For peer review only - http://bmjopen.bmj.com/site/about/guidelines.xhtml

"Some surgeons, they only know how to do a plate and what you will see is plate plate plate plate so it comes down to also what you are good at sometimes and you don't want something else."

Staff (Surgeon) 16

In contrast some surgeons were able to accept community equipoise (uncertainty within the expert medical community)[26], accepting their less preferred intervention if required.

Acceptability of the study procedures

Consent

Participants and carers were rarely able to describe the study in their own words but they tended to recall or recognise the interventions.

"Yes, because one is on the outside and one is a rod through the middle isn't it"

Participant 4

Several participants described struggling to engage with information about the study around the time of their surgery explaining that they were not "in a fit state" (participant 5) to ask questions or were "trying to so hard to be normal (participant 3)."

Interviews with staff showed that they described the study to patients in simple terms. Randomisation, for example was explained using phrases such as '50/50' or 'computer decides.' When explaining the study, staff also emphasised that both treatments are routinely used and that their surgeon was happy for them to receive either intervention, as they believed these were important to patients.

"They're going to have surgery anyway and then like I said if you say we don't know which the better one is and the surgeon is happy, I think is the key point is that the surgeon is happy for them to be part of the study"

Staff (RA) 1

The majority of staff found involving relatives in the discussion about the study to be helpful as it enabled patients to be supported in their decision-making. In contrast, a minority of staff felt, that it was easier to consent patients to studies when their relatives were not involved as they could be protective and involving them could lead to more potential participants declining.

"Sometimes you can find it easier if you approach them to try and time it around their visiting hours and so they've got a relative with them and so you can kind of talk to them as a family and a lot more patients feel more comfortable with that"

Staff (RA) 7

Two of seven patients who were entered into the study under nominated consultee agreement withdrew when they were approached to consent to continue in the study after surgery. One of these seven patients participated in an interview. She acknowledged that she "*wasn't sufficiently with it to give an opinion*" (participant 1) prior to surgery.

Randomisation

The majority of staff found randomisation was acceptable to patients, explaining that it was considered acceptable because of the explanation that the surgeon thought both treatments were appropriate. Two members of staff, in contrast, found patients disliked randomisation and wanted their surgeons to choose the treatment that was most appropriate for them.

For peer review only - http://bmjopen.bmj.com/site/about/guidelines.xhtml
For peer review only

When randomisation was described to participants during their interview, they accepted it or seemed indifferent towards it. Several participants, however, demonstrated therapeutic misconception: "*when a research subject fails to appreciate the distinction between the imperatives of clinical research and of ordinary treatment, and therefore inaccurately attributes therapeutic intent to research procedures*".[27] They believed that they or their relative would receive the most appropriate intervention for them.

> *"I think I prefer the one they done. I mean I didn't have a say in it, they decided what they thought was best, you know but I preferred the one they done definitely."*

Participant 6

Blinding

A minority of participants were told of their treatment allocation following surgery or when they returned for follow-up appointments. Some staff felt that blinding participants to their treatment allocation would be difficult as patients could see their allocation in the letter sent to their GP or X-rays, or they may be told by another member of staff. Some staff wanted to tell patients who asked which intervention they were allocated as one explained, "*I wouldn't be particularly comfortable, on a personal level... I would want to tell them what they have had*" (surgeon 8).

Case Reporting Forms (CRFs)

Research staff found that completing CRFs with the patient group could be challenging, as most patients were frail and often had long-term medical problems. They felt the questionnaires were lengthy and tiring for patients who often needed support completing them. They tended to ask the questions as part of a conversation to pull out the information for the questionnaire as they found some patients were unable to express their experience using the responses in the scales. Several RAs felt that where patients were wary of participating in studies this was usually due to the burden of follow-up rather than a dislike of randomisation or preference for one of the interventions.

> *"Some of the questions you have to try to put it into different words for them to understand, some of the questions don't make any sense to them"*

Staff (RA) 2

Mechanisms of impact

We identified intermediate outcomes that might be associated with the effect of the interventions on the primary outcomes of interest. Associations between the factors identified here, the interventions and the outcome of interest will assessed in a definitive trial. Factors that might influence patients' recovery after a distal femur fracture were identified by patients and public representatives and from data collected from interviews with participants and staff. Factors fell into three groups: existing factors relating to the characteristics of patients including their age, cognition and psychological factors such as self-efficacy; injury and treatment factors including the injury itself and factors relating to the surgeon and centre in which the treatment was delivered; post-discharge factors including rehabilitation provided, living arrangements and availability of support at home both from social care and from family. These factors are presented in supplemental Table S3.

Context

Contextual differences between the participating centres may have led to differences in how the study was implemented. Surgeons' working in departments where several trials were running may have been more engaged in research and more inclined to participate.

> *"However, that's changing and we've got a lot more of the consultants on board now and a lot more involved in the trial… there's consultants that are running trials in hand orthopaedics, the more elective side of things but also do the trauma lists*

For peer review only - http://bmjopen.bmj.com/site/about/guidelines.xhtml

and so are getting more involved with what's going on in research and therefore they're more happy to be involved in it and identifying the patients and things like that."

Staff (RA) 4

Some staff felt that differences in the size of the research team, their workload and working hours could influence how the study was implemented. For example, the presence of RAs in the trauma meeting could facilitate screening but required RAs to start work before eight, which was not possible in every centre.

I'm keen to see what is going to happen when we do get the other research nurses going to the trauma meeting because I feel that that's going to make a big difference and I think our recruitment will increase quite a lot but I might be completely wrong in thinking that.

Staff (RA) 4

I think if we didn't have enough studies running to have a fully funded research team then it would be tricky.

Staff (surgeon) 20

Meanwhile, some centres relied on one or part time RAs. This can result in patients being missed either when the RA was away or because they were struggling with their workload.

There was nobody in [to recruit] this Friday just gone and it can happen because I've got to be everywhere.

Staff (RA) 16

DISCUSSION

We have reported a randomised feasibility study, and to our knowledge the first with an embedded process evaluation, to plan a future trial of the treatment of patients with a fracture of the distal femur. We found the number of eligible patients screened closely matched the original estimate of 1.5 patients per centre-month. The recruitment rate into the trial, however, fell substantially short of this. The process evaluation identified very significant surgeon-related barriers to recruitment into the study. Research cultures were found to vary widely between centres; a positive culture was a strong predictor of improved recruitment performance.

This study has challenged many of the assumptions which underpinned the development of the trial protocol. The recruited population had a much lower prevalence of cognitive impairment, less frailty and was physiologically fitter than the previously characterised incident fracture population.[4] Surgeons were actively selecting fitter patients for operative treatment in a way that we had not anticipated. The eligible population is therefore somewhat different than we originally expected and hence the design for the definitive trial can be modified in important ways.

It is clear that research staff can and do miss potentially eligible patients if the working practices at hospitals do not facilitate full integration of the research staff into the clinical team. Although the specifics vary between hospitals, crucial decision points exist within the clinical process, such as daily trauma meetings, into which research staff must be integrated in order to be able to engage clinicians in research studies.

Surgeons reported a lack of their confidence in the management of this relatively uncommon fracture, the operative treatment of which is perceived to be technically demanding. The combination of this common anxiety and some individuals' lack of equipoise, posed a very significant barrier to recruitment into this study. Similar challenges have been previously identified in other surgical trials.[28, 29] Interventions that have included training on community equipoise have been successful when targeted at multidisciplinary teams and surgeons, improving both clinicians' confidence and

For peer review only - http://bmjopen.bmj.com/site/about/guidelines.xhtml

potentially recruitment rates,[30 31] though definitive evidence is lacking.[32] We propose that a modified protocol that includes an intervention to promote awareness and understanding of the existing community equipoise will be able to overcome these surgeon-related barriers to recruitment.

Our findings demonstrate that the trial design tested in this feasibility study is unlikely to be successful on a larger scale. However, the recruitment rate of 0.42 participants per centre-month is in line with that reported by the FixDT trial, which tested similar interventions for treating fractures of the tibia and delivered to target and budget.[25]

We propose a modified definitive trial with a further internal pilot to confirm recruitment rates; this will be based upon the findings of this feasibility study, with an integrated recruitment intervention to support surgeons in their decision-making to recruit participants.

We conclude that a trial comparing functional outcome in cognitively intact patients aged 18 years or older who have sustained a fragility fracture of the distal femur, defined as a fall from standing height, treated with a nail or plate, is feasible with appropriate mitigation strategies incorporated to avoid failure.

SUPPLEMENTARY FILES

Study protocol

Statistical Analysis Plan

Supplementary tables 1-3

ACKNOWLEDGEMENTS

Contributions of authors

Xavier L Griffin (Associate Professor of Trauma Surgery) contributed to the conception, design, conduct and reporting of the study.

Matthew L Costa (Professor of Orthopaedic Trauma Surgery) contributed to the conception, design and reporting of the study.

Emma Phelps (Postdoctoral Research Associate in Mixed Methods Research) contributed to the design, conduct and analysis of the process evaluation and the reporting of the study.

Nicholas Parsons (Associate Professor of Medical Statistics, Statistics and Epidemiology) contributed to the design, conduct, analysis and reporting of the study.

Melina Dritsaki (Senior Health Economist) contributed to the design and conduct of the study.

Juul Achten (Scientific Officer Oxford Trauma) contributed to the conception, design, conduct and reporting of the study.

Elizabeth Tutton (Senior Research Fellow) contributed to the design, conduct and analysis of the process evaluation and the reporting of the study.

Robin Lerner (Clinical Trial Manager) contributed to the conduct and reporting of the study.

Alwin McGibbon (Patient & Public Representative) contributed to the design, conduct and reporting of the study.

Janis Baird (Professor of Public Health and Epidemiology) contributed to the conception, design, conduct, reporting and analysis of the study.

For peer review only

For peer review only - http://bmjopen.bmj.com/site/about/guidelines.xhtml

Principal collaborators

Mr David Noyes, John Radcliffe Hospital, Oxford University Hospitals NHS Foundation Trust

Prof Peter Giannoudis, Leeds General Infirmary, The Leeds Teaching Hospitals NHS Trust

Prof Ben Ollivere, Queen's Medical Centre, Nottingham University Hospitals NHS Trust

Ms Charlotte Lewis, Queen Alexandra Hospital, Portsmouth Hospitals NHS Trust

Mr Andrew McAndrew, Royal Berkshire Hospital, Royal Berkshire NHS Foundation Trust

Mr Haroon Majeed, Mr Damian McClelland, Royal Stoke Hospital, University Hospitals of North Midlands NHS Trust

Mr Ashwin Kulkarni, Leicester Royal Infirmary, University Hospitals of Leicester NHS Trust

Funding

This project was funded by the Health Technology Assessment programme (HTA) (project number 15/59/22) and will be published in full in the NIHR HTA Journal. Further information available at: https://www.journalslibrary.nihr.ac.uk/programmes/hta/155922/#/. This work was also supported by NIHR Oxford Biomedical Research Centre.

The funders had no role in the design and conduct of the study; collection, management, analysis, and interpretation of the data; preparation, review, or approval of the manuscript; and decision to submit the manuscript for publication.

Data sharing

All data requests should be submitted to the corresponding author for consideration. Please note exclusive use will be retained until the publication of major outputs. Access to anonymised data may be granted following review.

Competing interests

Matthew Costa is a member of the UK NIHR HTA General Board. Janis Baird received funding from Danone Nutrica Early Life Nutrition for a specific research study which aims to improve the nutrition and Vitamin D status of pregnant women and is collaborating with Iceland Foods Ltd to evaluate the impact fruit and vegetable availability on diet.

For peer review only

REFERENCES

1. Court-Brown CM, Caesar B. Epidemiology of adult fractures: A review. *Injury* 2006;37(8):691-7. doi: 10.1016/j.injury.2006.04.130
2. Martinet O, Cordey J, Harder Y, et al. The epidemiology of fractures of the distal femur. *Injury* 2000;31 Suppl 3:C62-3.
3. Griffin XL, Parsons N, Zbaeda MM, et al. Interventions for treating fractures of the distal femur in adults. *Cochrane Database Syst Rev* 2015;8:CD010606. doi: 10.1002/14651858.CD010606.pub2
4. Smith JRA HR MJ, Morrison R, Yip G, Aquilina A, Kelly M. Distal femoral fractures: The size of the problem in the UK. *Orthopaedic Trauma Society* 2013
5. Browner BD EC. The science and practice of intramedullary nailing. *Philadelphia* 1987;Lea & Febiger
6. Miller DL, Goswami T, Prayson MJ. Overview of the locking compression plate and its clinical applications in fracture healing. *J Surg Orthop Adv* 2008;17(4):271-81.
7. Tornetta P, Egol KA, Ertl JP, et al. Locked plating versus retrograde nailing for distal femur fractures: A multicentre randomized trial (Abstract 39). Orthopaedic Trauma Association Annual Meeting. Phoenix, Arizona, USA, 2014.
8. Hoskins W, Sheehy R, Edwards ER, et al. Nails or plates for fracture of the distal femur? *The Bone & Joint Journal* 2016;98-B(6):846-50. doi: 10.1302/0301-620x.98b6.36826

For peer review only - http://bmjopen.bmj.com/site/about/guidelines.xhtml

9. Griffin XL, Costa ML, Achten J, et al. Trial of Acute Femoral Fracture Fixation (TrAFFix): study protocol for a randomised controlled feasibility trial. *Trials* 2017;18(1):538. doi: 10.1186/s13063-017-2265-0
10. Brooks R. EuroQol: the current state of play. *Health Policy* 1996;37(1):53-72.
11. Lecky F. Twenty-five years of the trauma audit and research network: a continuing evolution to drive improvement. *Emerg Med J* 2015;32(12):906-8. doi: 10.1136/emermed-2015-205460
12. Roberts HC, Denison HJ, Martin HJ, et al. A review of the measurement of grip strength in clinical and epidemiological studies: towards a standardised approach. *Age Ageing* 2011;40(4):423-9. doi: 10.1093/ageing/afr051
13. Rockwood K, Song X, MacKnight C, et al. A global clinical measure of fitness and frailty in elderly people. *CMAJ* 2005;173(5):489-95. doi: 10.1503/cmaj.050051
14. Sherbourne CD, Stewart AL. The MOS social support survey. *Soc Sci Med* 1991;32(6):705-14.
15. Schwarzer R, Bäßler J, Kwiatek P, et al. The Assessment of Optimistic Self-beliefs: Comparison of the German, Spanish, and Chinese Versions of the General Self-efficacy Scale. *Applied Psychology* 1997;46(1):69-88. doi: 10.1111/j.1464-0597.1997.tb01096.x
16. Hounsome N, Orrell M, Edwards RT. EQ-5D as a Quality of Life Measure in People with Dementia and Their Carers: Evidence and Key Issues. *Value in Health* 2011;14(2):390-99. doi: http://dx.doi.org/10.1016/j.jval.2010.08.002
17. Salen BA, Spangfort EV, Nygren AL, et al. The Disability Rating Index: an instrument for the assessment of disability in clinical settings. *J Clin Epidemiol* 1994;47(12):1423-35.
18. National Institute for Health and Care Excellence. Guide to the methods of technology appraisal. In: Excellence NIfHaC, ed.: National Institute for Health and Care Excellence, 2013.
19. Department of Health and Social Care. NHS Reference Costs 2015 to 2016: Department of Health and Social Care, 2016.
20. National Health Service. NHS Supply Chain Catalogue [Available from: https://my.supplychain.nhs.uk/catalogue accessed Mar 2018.
21. Curtis L, Burns A, Personal Social Services Research Unit. Unit Costs of Health and Social Care. *Personal Social Services Research Unit* 2016
22. van Hout B, Janssen MF, Feng YS, et al. Interim scoring for the EQ-5D-5L: mapping the EQ-5D-5L to EQ-5D-3L value sets. *Value Health* 2012;15(5):708-15. doi: 10.1016/j.jval.2012.02.008
23. Moore GF, Audrey S, Barker M, et al. Process evaluation of complex interventions: Medical Research Council guidance. *BMJ* 2015;350:h1258. doi: 10.1136/bmj.h1258
24. Herdman M, Gudex C, Lloyd A, et al. Development and preliminary testing of the new five-level version of EQ-5D (EQ-5D-5L). *Qual Life Res* 2011;20(10):1727-36. doi: 10.1007/s11136-011-9903-x
25. Costa ML, Achten J, Hennings S, et al. Intramedullary nail fixation versus locking plate fixation for adults with a fracture of the distal tibia: the UK FixDT RCT. *Health Technol Assess* 2018;22(25):1-148. doi: 10.3310/hta22250
26. Freedman B. Equipoise and the ethics of clinical research. *N Engl J Med* 1987;317(3):141-5. doi: 10.1056/NEJM198707163170304
27. Lidz CW, Appelbaum PS. The therapeutic misconception: problems and solutions. *Med Care* 2002;40(9 Suppl):V55-63. doi: 10.1097/01.MLR.0000023956.25813.18
28. Donovan JL, Paramasivan S, de Salis I, et al. Clear obstacles and hidden challenges: understanding recruiter perspectives in six pragmatic randomised controlled trials. *Trials* 2014;15(1):5. doi: 10.1186/1745-6215-15-5
29. Donovan JL, de Salis I, Toerien M, et al. The intellectual challenges and emotional consequences of equipoise contributed to the fragility of recruitment in six randomized controlled trials. *Journal of Clinical Epidemiology* 2014;67(8):912-20. doi: https://doi.org/10.1016/j.jclinepi.2014.03.010
30. Realpe A, Adams A, Wall P, et al. A new simple six-step model to promote recruitment to RCTs was developed and successfully implemented. *Journal of Clinical Epidemiology* 2016;76:166-74. doi: 10.1016/j.jclinepi.2016.02.002

For peer review only - http://bmjopen.bmj.com/site/about/guidelines.xhtml

31. Donovan JL, Rooshenas L, Jepson M, et al. Optimising recruitment and informed consent in randomised controlled trials: the development and implementation of the Quintet Recruitment Intervention (QRI). *Trials* 2016;17:283. doi: 10.1186/s13063-016-1391-4
32. Townsend D, Mills N, Savović J, et al. A systematic review of training programmes for recruiters to randomised controlled trials. *Trials* 2015;16(1):432. doi: 10.1186/s13063-015-0908-6

FIGURE LEGENDS

Figure 1: Summary of recruitment and data collection. [$]The eligible age was changed from 50 to 18 during the study.

For peer review only

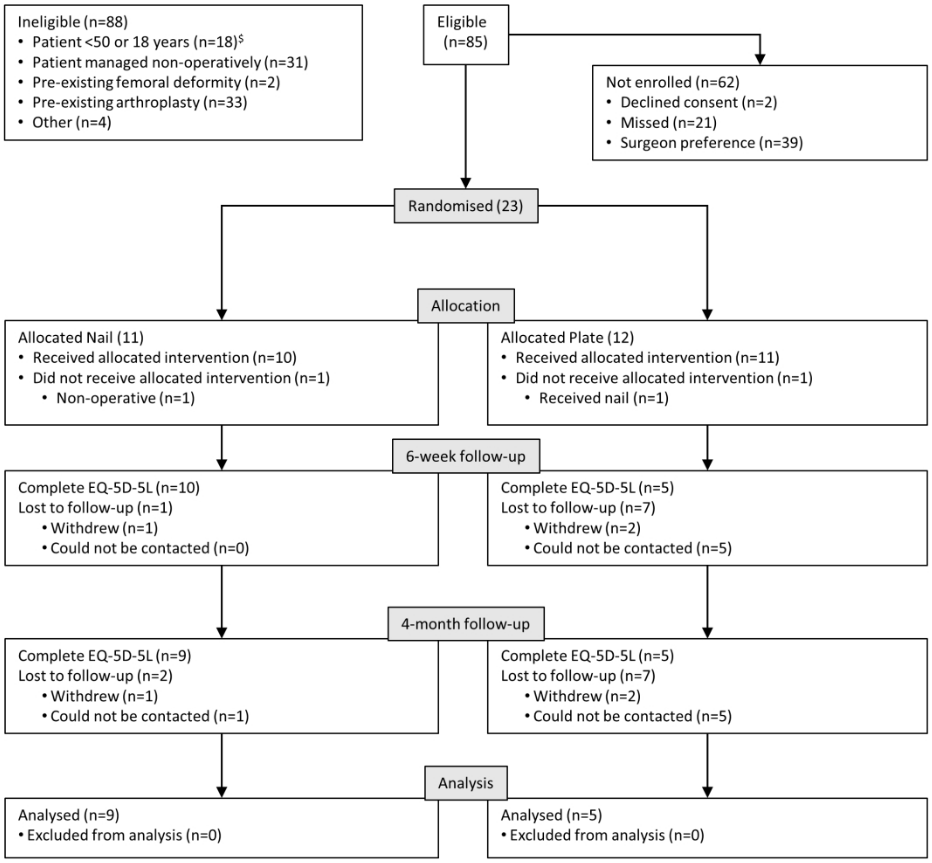

Figure 1: Summary of recruitment and data collection. $The eligible age was changed from 50 to 18 during the study.

190x188mm (300 x 300 DPI)

Supplemental table 1: Reasons for non-inclusion and mechanisms of injury returned by participating hospitals. $The eligible age was changed from 50 to 18 during the study. Site names have been removed, *denotes major trauma centre.

	Site ID							
**Reason ineligible**	**A***	**B**	**C**	**D***	**E**	**F***	**G***	**Total**
Patient 50 or 18 years old$	3	0	2	6	0	4	3	**18**
Patient managed non-operatively	2	18	2	1	3	0	5	**31**
Pre-existing femoral deformity	1	0	1	0	0	0	0	**2**
Pre-existing arthroplasty	4	2	3	5	6	6	7	**33**
Other:	1	0	0	0	0	3	0	**4**
**Reason missed**								
Staffing/weekend	0	0	1	1	0	3	0	**5**
Technical (RRAMP fault)	0	0	0	0	1	0	0	**1**
Unknown (added from monitoring)	1	0	0	0	4	0	10	**15**
Surgeon preference	10	15	5	3	1	5	0	**39**
**Reason declined**								
Patient doesn't want to be part of research	0	0	1	0	0	0	0	**1**
Consultee doesn't want to be part of research	0	1	0	0	0	0	0	**1**
**Mechanism of Injury (of eligible patients)**								
Fall from <2m	15	5	6	6	9	5	9	**55**
Fall from >2m	1	0	0	0	0	2	0	**3**
Other	4	0	1	1	0	4	1	**11**
**Mechanism of Injury (of ineligible patients)**								
Fall from <2m	7	0	6	5	9	7	11	**45**
Fall from >2m	0	0	0	0	0	1	0	**1**
Other	3	0	2	7	0	5	4	**21**
Not reported	1	20	0	0	0	0	0	**21**

For peer review only

Supplemental table 2: Overview of process evaluation findings

Item	Component	Data Sources	Quantitative Findings	Qualitative findings
Reach	• Number of eligible patients reached	• Screening logs<br>• Enhanced screening	• 23 of 85 eligible patients were included in the study<br>• 82 patients with distal femoral fractures were not recorded on the initial screening logs<br>• The number of distal femoral fractures missing from the screening logs varied from 0 to 34 between the centres	• Staff were confident that few patients were eligible for the study, suggesting that they were unaware that potentially eligible patients were being missed
Fidelity	• Procedure for screening patients<br>• Application of the study eligibility criteria	• Screening logs<br>• Enhanced screening<br>• Interviews with staff	• 82 patients who were admitted to the participating centres with a distal femoral fracture during the recruitment period were not included on the screening logs<br>• 39 eligible patients were excluded due to surgeon preference	• Clinical teams did not always notify the research teams of eligible patients<br>• In two centres, RAs did not attend the daily trauma meeting, where patient eligibility could be discussed<br>• Staff emphasised that there were fewer patients with distal femur fractures than they had expected and reported that many were ineligible<br>• Some staff felt that many surgeons had strong preferences for one of the two interventions and were unwilling to randomise patients<br>• These qualitative findings are linked to the themes i) making it work categories (category: research culture) and ii) knowing it's the right decision (category: making sense of the eligibility criteria)
	• Consent discussion	• Audio – recordings of patient staff interactions	• N/A	• When discussing the study with patients, RAs emphasised that both treatments are routinely used and that their surgeon was happy for them to receive either intervention<br>• They also avoided jargon and described the study in simple terms, using phrases such as '50/50', 'computer decides' and 'heads or tails' to explain randomisation

For peer review only - http://bmjopen.bmj.com/site/about/guidelines.xhtml

		• Interviews with RAs and participants		• These findings are linked to the theme: navigating research with patients after orthopaedic trauma (categories: making sense of the study and enabling participation)
	• Deviations from protocol/ application of operative procedures	• CRFs<br>• Interviews with staff	• Two protocol deviations were recorded. One patient allocated a plate received a nail and one patient allocated a nail received a plate	• Some staff described strategies that may help avoid cross overs in surgical trials. If the treating surgeon was uncomfortable or unwilling to perform one of the interventions then another surgeon could take that patient onto their operating list instead.<br>• These findings are linked to the themes i) making it work (category: research culture) and ii) knowing it's the right decision (category: clinical uncertainty)
	• Delivery of post-operative rehabilitation components<br>• Patient compliance with postoperative	• CRFs<br>• Interviews with participants and surgeons	• Eight patients were instructed to weight bear as tolerated, three were instructed to weight bear as required to allow mobilisation, seven partial weight bear and five non-weight bear.<br>• At six week follow-up, six of 15 participants who completed the questionnaire were weight bearing and nine were not.<br>• At four month follow-up, nine of the 14 participants who completed the questionnaire were weight bearing and five were not.	• Surgeons held different views about the most appropriate rehabilitation instructions for patients with this fracture but the majority favoured early weight bearing<br>• For participants, rehabilitation was typically slow and for some it was a frustrating process.<br>• Loss of confidence and pain hindered rehabilitation for some participants
Acceptability	• Acceptability of Interventions	• Screening logs<br>• Interviews with participants and staff	• 39 eligible patients were excluded due to surgeon preference	• Some staff felt that many surgeons had strong preferences for one of the two interventions and were unwilling to randomise patients<br>• Surgeons' unwillingness to randomise patients stemmed from their beliefs about whether both methods of fixation were appropriate for a patients' specific fracture and their own surgical skills

For peer review only

				• Participants tended to be less concerned about the nature of the interventions as long as their fracture was treated appropriately.<br>• The majority of participants recalled that the study involved a plate vs nail<br>• Staff understood that it would be difficult for patients to appreciate the differences between these two interventions and that that the type of metalwork used to fix their fracture was unlikely to be a priority for patients<br>• These findings are linked to the themes i) knowing it's the right decision (categories: surgeon preference and clinical equipoise), and ii) navigating research after orthopaedic trauma (category: making sense of the study)
	• Acceptability of study procedures	• Screening logs<br>• Interviews with participants and staff	• Two patients declined to participate in the study<br>• Two of seven patients who were entered in the study under nominated consultee consent declined when approached for retrospective consent to continue in the study after surgery<br>• One participant withdrew from the study after surgery	• RAs found that in the majority of cases patients were accepting of the nominated consultee consent procedure<br>• The majority of staff found randomisation to be acceptable to most patients<br>• When randomisation was described to participants during their interview, they typically seemed to be accepting of this procedure or indifferent towards it<br>• Several participants demonstrated a misunderstanding of randomisation at interview<br>• These findings link to the theme: navigating research with patients after orthopaedic trauma (categories: making sense of the study and enabling participation)
How is delivery achieved?	• Facilitators and barriers in the delivery of the study	• Interviews with staff	• N/A	• The overarching concept of facilitating trial recruitment was identified from our analysis of the interview data. This concept aids our understanding of how delivery was achieved by highlighting facilitators and barriers to delivering complex trauma trails such as TrAFFix.<br>• Themes comprising this concept are navigating research with patients after orthopaedic trauma, ii) knowing it's the right decision, and iii) making it work

For peer review only

Supplemental table 3: Overview of process evaluation findings

Existing Factors	Injury and Treatment	Post-discharge factors
• Age<br>• Comorbidities<br>• Cognition<br>• Pre-injury mobility<br>• Pre-injury activity<br>• Nutrition<br>• Psychological factors (emotional resilience, self-efficacy, depression)	• Injury event<br>• Surgeon experience<br>• Surgeon preference<br>• Surgeon beliefs about rehabilitation<br>• Experience and care in hospital<br>• Communication – clear language<br>• Team work	• Transitions (e.g. from hospital to home)<br>• Appropriate discharge residence<br>• Follow-up<br>• Rehabilitation<br>• Pain<br>• Confidence<br>• Family (living arrangements, advocacy)<br>• Practical support<br>• Social support<br>• Changes to lifestyle after injury<br>• Loss of dignity<br>• Loss of independence<br>• Funding and access to social care<br>• Holistic approach to care<br>• Recovery pathways<br>• Balance of care between family and the system

For peer review only

TrAFFix

TrAFFix

Trial of Acute Femoral fracture Fixation

Statistical Analysis Plan

Version 2.0 – 2Nov2016

Based on Protocol version 2.0 – 27Jul2016

Name	Title	Role (Author/Reviewer/Approver)	Signature	Date
Nick Parsons	Trial Statistician	Author		02Nov2016
Robin Lerner	Trial Manager	Reviewer		02Nov2016
Xavier Griffin	Chief Investigator	Reviewer		02Nov2016

For peer review only

Oxford Clinical Trials Research Unit (OCTRU)
and

OCTRU
Oxford Clinical Trials Research Unit

WARWICK
THE UNIVERSITY OF WARWICK

Note: this document is version controlled and all draft versions and related communications should be stored in the Trial Master File

For peer review only - http://bmjopen.bmj.com/site/about/guidelines.xhtml

CONTENTS

1. INTRODUCTION 3
   1.1 Key personnel 3
2. BACKGROUND INFORMATION 3
   2.1 Research Hypotheses and Study Objectives 3
   2.2 Study Design and trial expected time-points 3
   2.3 Eligibility 4
   2.4 Treatment Interventions 4
   2.5 Sample Size 4
   2.6 Randomisation 5
   2.7 Hypotheses and Definition of Primary and Secondary Outcomes 5
   2.8 Outcomes Assessment Schedule 5
   2.9 Statistical Analysis Outline 5
3. QUALITY CONTROL AND DATA VALIDATION 7
   3.1 Definition of Derived Data 7
   3.2 Validation of the Primary analysis 7
4. INTERIM ANALYSIS AND DATA REVIEW 7
5. SPECIFICATION OF STATISTICAL PACKAGES 7
6. DESCRIPTIVE ANALYSES 7
   6.1 Representativeness of Study Sample and Patient Throughput 8
   6.2 Baseline Comparability of Randomised Groups 8
   6.3 Comparison of Losses to Follow-up 8
   6.4 Description of Available Data 8
   6.5 Description of Compliance with Intervention 9
   6.6 Unblinding of Randomised Treatments 9
   6.7 Reliability 9
7. DEFINITION OF POPULATIONS FOR ANALYSIS 9
8. ANALYSES TO ADDRESS PRIMARY AIMS 9
   8.1 Evaluation/Definition of Primary Outcome (where applicable) 9
   8.2 Statistical Methods Used for Analysis of Primary Outcome 9
   8.3 Adjustment of P values for Multiple Testing 10
   8.4 Missing Data 10
   8.5 Pre-specified Subgroup Analysis 10
   8.6 Treatment by Centre Interaction 10
   8.7 Sensitivity Analysis 10
9. ANALYSIS TO ADDRESS SECONDARY AIMS 10
   9.1 Evaluation/Definition of Secondary Outcomes (where applicable) 11
   9.2 Statistical Methods Used for Analysis of Secondary Outcomes 11
   9.1 Pre-specified Subgroups of Key Secondary Outcomes (where applicable) 11
   9.2 Sensitivity Analysis of Key Secondary Outcomes (where applicable) 11
   9.3 Health Economics and Cost Effectiveness 11
10. ADDITIONAL ANALYSES 11
   10.1 Exploratory analyses 11
   10.2 Blinded analysis 11
   10.3 Meta-analyses (if applicable) 12
11. SAFETY ANALYSIS 12

For peer review only - http://bmjopen.bmj.com/site/about/guidelines.xhtml

12. APPENDIX: GLOSSARY OF ABBREVIATIONS 12
13. DOCUMENT HISTORY 12
14. CHANGES FROM PREVIOUS VERSION OF SAP 12

1. INTRODUCTION

This document details the proposed presentation and analysis for the main paper(s) reporting results from the ***NIHR HTA funded Trial of Acute Femoral Fracture Fixation.*** The results reported in these papers should follow the strategy set out here. Subsequent analyses of a more exploratory nature will not be bound by this strategy, though they are expected to follow the broad principles laid down here. The principles are not intended to curtail exploratory analysis (for example, to decide cut-points for categorisation of continuous variables), nor to prohibit accepted practices (for example, data transformation prior to analysis), but they are intended to establish the rules that will be followed, as closely as possible, when analysing and reporting the trial.

The analysis strategy will be available on request when the principal papers are submitted for publication in a journal. Suggestions for subsequent analyses by journal editors or referees, will be considered carefully, and carried out as far as possible in line with the principles of this analysis strategy; if reported, the source of the suggestion will be acknowledged.

Any deviations from the statistical analysis plan will be described and justified in the final report of the trial. The analysis should be carried out by an identified, appropriately qualified and experienced statistician, who should ensure the integrity of the data during their processing. Examples of such procedures include quality control and evaluation procedures.

1.1 Key personnel

List of key people involved in the drafting and reviewing this SAP, together with their role in the trial and their contact details.

Author(s) (Trial statistician(s)):

Nick Parsons – Senior Statistician, Trial Statistician

Reviewers (Chief Investigator, Trial Manager, DSMC, TSC, Statistician as appropriate):

Xavier Griffin – Chief Investigator

Approver (Senior Statistician):

Nick Parsons – Senior Statistician, Trial Statistician

2. BACKGROUND INFORMATION

2.1 Research Hypotheses and Study Objectives

1. Assess the feasibility of a future definitive trial.
2. Perform a process evaluation to understand the generalisability and likely success of a future trial.
3. Explore the validity of self and proxy-reporting of the EQ-5D-5L in this specific population.

2.2 Study Design and trial expected time-points

The study design including the type of trial (e.g. parallel group, cluster, crossover, factorial, single arm), single/multi-centre, blinding, allocation ratio, trial framework (e.g. superiority, equivalence, non-inferiority, exploratory), primary outcome and crucial trial time-points.

For peer review only - http://bmjopen.bmj.com/site/about/guidelines.xhtml

TRAFFix is a randomized (1:1 allocation ratio) controlled multicentre trial with two (parallel) groups assessing fixation of the femur after fixation using either a nail or a plate.

Date of grant activation	01Jun2016
Date of start of recruitment:	XXAug2016
Date of expected end of recruitment:	31May2017
Date expected end follow-up:	30Sep2016
Date expected start of analysis:	01Oct2017
Date End of grant:	31Nov2017
Target number of subjects:	52
Participating Centres:	6

2.3 Eligibility

Inclusion criteria

Patients will be eligible for this study if they:

- are ≥50 years old as a surrogate for bone-density and therefore fragility fracture,
- have a fracture of the femur involving the distal two "Muller" squares,
- would, in the opinion of the attending surgeon, benefit from internal fixation of the fracture.

Exclusion criteria

Patients will be excluded from this trial if they have:

- a loose knee or hip arthroplasty requiring revision,
- pre-existing femoral deformity,
- an arthroplasty that precludes nail fixation.

2.4 Treatment Interventions

Intramedullary nailing: Fixation of the fracture will be achieved with a proximally and distally locked nail that spans the entire diaphysis of the femur. All nails will be introduced retrograde through the knee joint. In this pragmatic trial, the details of surgical incision and approach, fracture reduction and supplementary fixation with wires or screws will be at the surgeon's discretion as per their normal clinical practice.

Locking plate fixation: Fixation of the fracture will be achieved with anatomical distal femoral locking-plate and screws. Locking plates will be defined as those in which at least one fixed angle locking screw is placed distal to the fracture. The operating surgeon will determine the length, number and type of additional screws. Additional fixation with lag screws and cerclage wires will be at the surgeon's discretion. In this pragmatic trial, the details of surgical incision and approach, fracture reduction, number and type of other screws and supplementary fixation with wires or screws will be at the surgeon's discretion as per their normal clinical practice.

2.5 Sample Size

Details of the sample size for primary outcomes and any co-primary or important key secondary outcomes (if applicable), including treatment effect, power, levels of statistical significance (one-tailed or two-tailed), clinical relevance and justification.

Data from the TrAFFix feasibility study will be used to calculate estimates of the standard deviation of the primary outcome measure (EQ-5D-5L) to drive a formal power analysis and sample size calculation for the

For peer review only - http://bmjopen.bmj.com/site/about/guidelines.xhtml

future definitive trial; evidence from other relevant sources reporting the metrics properties of EQ-5D-5L in the study population will also be used to inform this process. We anticipate that each of 6 participating centres will treat approximately 1.5 eligible patients per month. Taking a conservative approach, we would expect, to ensure feasibility, to be able to recruit 1.0 patients per month per centre. Given our schedule for centre opening times and recruitment length, we will have 52 centre months available to recruit for this study. Assuming that the recruitment rate is 1.0 per month per centre and monthly centre counts of patient recruitment numbers are approximately Poisson distributed and independent of one another, then this will allow us to estimate the recruitment rate with a 95% confidence interval of 0.73 - 1.28.[35] Therefore recruiting 52 patients in total should provide sufficiently precise estimates of the monthly recruitment rate to decide if a definitive trial is feasible.

2.6 Randomisation

The method(s) of randomisation, including the randomisation allocation ratio and any stratification/minimisation factors.

The unit of randomisation will be the individual patient. Randomisation will have a 1:1 allocation to two intervention groups (Nail and Plate) and be stratified by recruiting centre (6 centres will be used) and chronic cognitive impairment (Yes or No). Within each strata a fixed block size of 4 will be used for all blocks.

Full details of the randomisation are available in TrAFFix_RBP_v1.0_16Aug2016, stored in the confidential statistical section of the TMF.

2.7 Hypotheses and Definition of Primary and Secondary Outcomes

Clear description of hypotheses and definitions of the primary and secondary outcome measures and, when applicable, any methods used to enhance the quality of measurements (e.g., multiple observations, training of assessors). It might be useful to specify types of data that will be encountered in this trial.

The principle aim of this feasibility study will be to determine the number of eligible and recruited patients in the trauma centres over the course of 10 months. The primary outcome measures for this study are the participant recruitment rate and the completion rate of the EQ-5D-5L at 4 months post-surgery.

2.8 Outcomes Assessment Schedule

**Outcomes**	Baseline	6-weeks	4-months
EQ-5D-5L	Pre + post injury	X	X
DEMQoL[A]	Pre + post injury	X	X
DRI[B]	Pre + post injury	X	X
Radiographs	X	X	
Complications	X	X	X
Health Economics			X
Qualitative interviews[C]	X	X	X

2.9 Statistical Analysis Outline (references refer to protocol)

Main statistical analysis

This feasibility study is not powered to formally assess the size of the treatment effect, rather to estimate the recruitment rate. The totality of the data collected will be used to assess the feasibility of a definitive large RCT; recruitment rate being the driver of the feasibility study design on the basis that unless a reasonable recruitment rate can be achieved no formal trial will be possible. The recruitment rate will be estimated based on data collected and a (Poisson distributed) 95% confidence interval determined for this measure.

If the estimated recruitment rate is such that a definitive trial is feasible then no formal analysis will be undertaken and data from the feasibility study will be locked and carried over into the main (definitive) trial. No formal analysis of treatment efficacy will be undertaken in this scenario. However, the study ISDMC will

For peer review only - http://bmjopen.bmj.com/site/about/guidelines.xhtml
For peer review only

see unblinded summary statistics, together with recruitment data, and will advise the TSC with relevant safety or ethical guidance as the study progresses. The reasons and patterns of any missing data, loss to follow-up and participant withdrawals will be carefully considered and reported, with particular emphasis on how these may impinge on the future trial.

If a definitive trial is not feasible, then outcome data will reported in the conventional manner. Baseline demographics (e.g. Age, Gender, cognitive status) will be compared between groups to ensure approximate balance has been achieved. This is a small study (n=52), so treatment group effects are unlikely to be estimated with much precision and consequently inferences will be tentative and reported as such. The main analysis will investigate differences in the primary outcome measure, EQ-5D-5L[21] score at 4 months, between the two treatment groups (Nail and Plate) on an intention-to-treat basis. In addition a per-protocol analysis will also be reported and early EQ-5D-5L status will also be assessed and reported at 6 weeks. Differences between groups will based on a normal approximation for EQ-5D-5L.[20 31] Tests will be two-sided and considered to provide evidence for a significant difference if p-values are less than 0.05 (5% significance level). The stratified randomisation procedure should ensure a balance in cognitive impairment and recruiting centre between test treatments. Although generally we have no reason to expect that clustering effects will be important for this study, in reality the data will be hierarchical in nature, with patients naturally clustered into groups by recruiting centre. Therefore we will account for this by generalising the conventional linear (fixed-effects) regression approach to a mixed-effects modelling approach; where participants are naturally grouped by recruiting centres (random-effects). This model will formally incorporate terms that allow for possible heterogeneity in responses for patients due to the recruiting centre, in addition to the fixed effects of the treatment groups, cognitive impairment and age and gender, as these latter participant characteristics may prove to be important moderators of the treatment effect. The mixed-effects model will be the primary analysis, and will be reported as such.

The main analyses will be conducted using specialist mixed-effects modelling functions available in the software package R (http://www.r-project.org/) where EQ-5D-5L[21] data will be assumed to be normally distributed; possibly after appropriate variance-stabilising transformation. The primary focus will be the comparison of the two treatment groups of patients, and this will be reflected in the analysis which will be reported together with appropriate diagnostic plots that check the underlying model assumptions. Results will be presented as mean differences between the trial groups, with 95% confidence intervals.

Secondary analyses will be undertaken using the above strategy for other approximately normally distributed outcome measures such as DRI[23]. For dichotomous outcome variables, such as complications related to the trial interventions, mixed effects logistic regression analysis will be undertaken with results presented as odds ratios (and 95% confidence intervals) between the trial groups. The temporal patterns of any complications will be presented graphically and if appropriate a time-to-event analysis (Kaplan-Meier survival analysis) will be used to assess the overall risk and risk within individual classes of complications. The reasons and patterns of any missing data, loss to follow-up and participant withdrawals will also be carefully considered and reported.

A proportion of study participants will be unable to self-report EQ-5D, often due to dementia, so an appropriate individual (e.g. carer, relative) will be asked to proxy-report. The Dementia Quality of Life Measure (DEMQoL) is a validated questionnaire specifically designed to assess quality of life in patients with dementia. DEMQoL can be self or proxy-reported (28 or 31 items respectively). In order to assess the relative performance and merits of EQ-5D and DEMQoL in the study population, these two measures will be compared in the subgroup of study participants who are unable to self-report EQ-5D. Given the relatively small size of this study, and minority of participants who will be unable to self-report, there will not be enough data to undertake formal statistical testing for differences between measures. However, graphical presentation of the distribution and relationship (correlation) between measures for individuals will provide will allow some

For peer review only - http://bmjopen.bmj.com/site/about/guidelines.xhtml

assessment to be made of the merits and metric properties (e.g. variance) of the two measures. Agreement or moderate to strong correlation between measures will indicate that they are measuring the same underlying (latent) trait (which we take to be the true QoL of the study participants). Utilities will also be calculated for the two measures and compared in a similar manner.

Economic Evaluation

The feasibility of a future definitive economic evaluation of treatment with modern intramedullary nails or anatomical locking plates for fragility fractures of the distal femur will be investigated in this study. The analysis plan for the health economics evaluation will be detailed in a separate analysis plan.

Process evaluation

Qualitative data collected to inform the process evaluation from interviews with patients, surgeons and other staff will be transcribed verbatim. The analysis plan for the process evaluation will be detailed in a separate analysis plan.

3. QUALITY CONTROL AND DATA VALIDATION

Quality control and data validation will be carried out in accordance with OCTRU SOPs and TrAFFix monitoring and data management plans.

TrAFFix Monitoring Plan V1.0 21Jul2016:

Z:\KC_TRAFFIX\TRAFFIX eTMF V3.0\Risk assessment and Monitoring\2. Monitoring Plan

TrAFFix Data Management Plan V1.0 21Jul2016

Z:\KC_TRAFFIX\TRAFFIX eTMF V3.0\Data Management\Data Management Plan

3.1 Definition of Derived Data

Definitions of any data derived for analysis outlined here with full details stored in the Data and Sharing Management Plan (see OCTRU SOP DM-008 Data and Sharing Management Plan).

3.2 Validation of the Primary analysis

The primary outcome and key secondary outcomes will be analysed, following the analyses detailed in this SAP, by a statistician independent of the trial using different statistical software (if possible). Any discrepancies will be reported in the Statistical report (See OCTRU SOP STATS-005 Statistical Report).

4. INTERIM ANALYSIS AND DATA REVIEW

A brief summary of the format, remit and time points for meetings of the Data and Safety Monitoring Committee including any planned formal interim analyses.

The Data and Safety Monitoring Committee is independent from sponsor and follows the TrAFFix_DSMC_Charter_V1.0_02Sep2016. No interim analyses are planned.

5. SPECIFICATION OF STATISTICAL PACKAGES

All analysis will be carried out using appropriate validated statistical software such as STATA, SAS, SPLUS or R statistical software. The relevant package and version number will be recorded in the Statistical report.

6. DESCRIPTIVE ANALYSES

Summary of flow of trial participants through the trial and baseline stratification, demographic and clinical characteristics of each group.

For peer review only - http://bmjopen.bmj.com/site/about/guidelines.xhtml

6.1 Representativeness of Study Sample and Patient Throughput

The flow of participants through each stage of the trial, including numbers of participants randomly assigned, receiving intended treatment, completing the study protocol, and analysed for the primary outcome is provided following the appropriate guideline (e.g. CONSORT). Protocol violations/deviations and information relating to the screening data including the number of ineligible patients randomised, together with reasons.

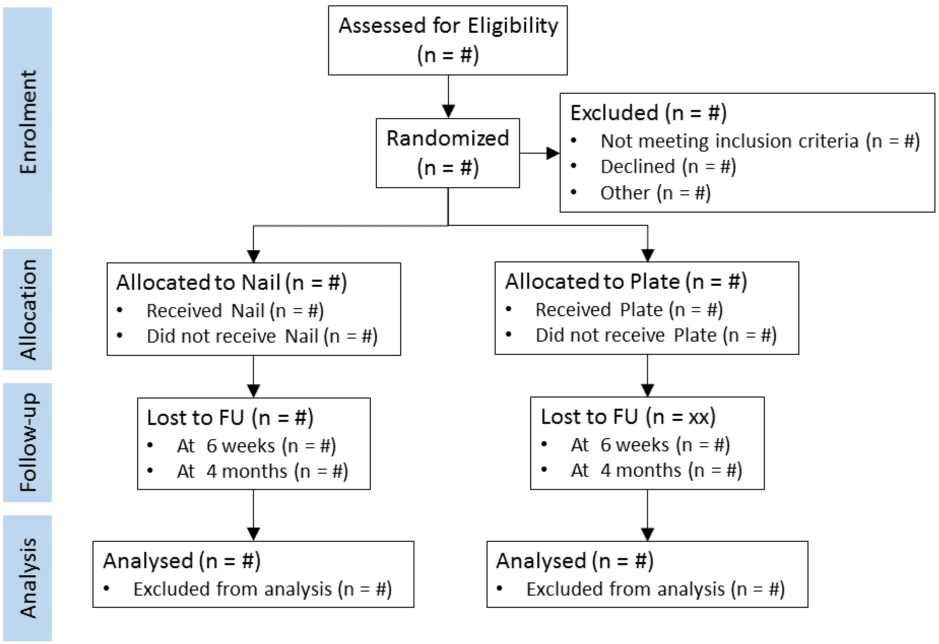

6.2 Baseline Comparability of Randomised Groups

Baseline characteristics are reported by treatment group, including the stratification/minimisation factors (if applicable) and important prognostic, demographic and clinical covariates).

Numbers (with percentages) for binary and categorical variables and means (and standard deviations), or medians (with lower and upper quartiles) for continuous variables will be presented; there will be no tests of statistical significance nor confidence intervals for differences between randomised groups on any baseline variable.

6.3 Comparison of Losses to Follow-up

Description of loss to follow-up together with reasons. Statistical tests comparing loss to follow-up to be undertaken and how these will be reported.

Loss to follow-up together with reasons will be reported by intervention arm. To assess differential losses between the groups this will be tested using absolute risk differences (95% confidence interval) and a chi-squared test. Any deaths (and their causes) will be reported separately.

6.4 Description of Available Data

The completeness of data required for primary and secondary outcomes is described, detailing methods to limit the possibility of missing data and methods for dealing with missing data when it occurs.

It seems likely that some data may not be available due to voluntary withdrawal of patients, lack of completion of individual data items or general loss to follow-up. Where possible the reasons for data missingness will be ascertained and reported. Although missing data are not expected to be a problem for this study, the nature

For peer review only - http://bmjopen.bmj.com/site/about/guidelines.xhtml

and pattern of the missingness will be carefully considered — including in particular whether data can be treated as missing completely at random (MCAR).

6.5 Description of Compliance with Intervention

Description of compliance with intervention – may include important covariates related to the interventional procedure or the standard treatment used in both arms.

A summary of the treatment received, as distinct from the treatment allocated, will be provided for each study participant. This is a pragmatic study, so detailed implementation of the study interventions will be left to the individual surgeons and will reflect their usual practice.

6.6 Unblinding of Randomised Treatments

Detail who is blinded in the study (participants, local site staff, outcome assessors). All cases of treatment unblinding will be listed, together with reasons and summarised (numbers, percentages)

As the surgical scars are clearly visible, the patients cannot be formally blinded to their treatment. Participants will only be informed of their treatment allocation at the end of the trial. In addition, the treating surgeons will also not be blind to the treatment, but will take no part in the post-operative assessment of the patients. The functional outcome data will be collected and entered onto the trial central database by a research assistant/data clerk in the trial central office.

6.7 Reliability

The radiographs collected will be reviewed by independent researchers at each hospital, A small sample of EQ-5D-5L utility scores will be checked by hand calculation to ensure that the computer algorithm has been implemented correctly.

7. DEFINITION OF POPULATIONS FOR ANALYSIS

Populations for analysis are defined as follows:

Intent to treat (ITT): all participants randomised in their randomised groups.

Per protocol (PP): all participants grouped by the intervention they received, rather than to which they were allocated. Participants receiving neither intervention will be excluded from the PP analysis

Safety: All participants who started the intervention or received a minimum amount of treatment as defined in the protocol.

8. ANALYSES TO ADDRESS PRIMARY AIMS

Statistical methods to be used to compare groups for primary outcome(s) and methods for point and interval estimation. Include methods for additional analyses, such as adjusted analyses and subgroup analyses, together with which populations will be analysed.

8.1 Evaluation/Definition of Primary Outcome (where applicable)

Criteria of evaluation and definition of outcomes and methods of calculation (rules for calculation of derived variables including definitions that can be programmed from the data).

Recruitment rate.

8.2 Statistical Methods Used for Analysis of Primary Outcome

Description of the primary and secondary/supplementary analyses and primary and secondary time-points (if applicable), including details of how results will be reported, both in tabular and graphical format.

For peer review only - http://bmjopen.bmj.com/site/about/guidelines.xhtml
For peer review only

The overall study recruitment rate will be estimated based on data collected and a 95% confidence interval determined for this measure. If the estimated recruitment rate is such that a definitive trial is feasible then no formal analysis will be undertaken and data from the feasibility study will be locked and carried over into the main (definitive) trial.

8.3 Adjustment of P values for Multiple Testing

Acknowledgement of the issue of multiple testing (if applicable), together with the rationale of the intention formally or informally adjust for multiplicity. If no adjustment planned this will be stated.

There is no multiple testing as only a single primary outcome is considered. Therefore significance levels used will be set at the conventional 5% level. Interim analyses of primary and secondary endpoints will not be carried out unless requested by the DSMC, who will formulate a plan for maintaining the overall study type I error rate if this is the case.

8.4 Missing Data

A description of methods utilised for dealing with missing, spurious (outliers) and unused data during statistical analysis including the type of missing data. If multiple imputation is to be used the methods will be specified. Methods for handling withdrawals and protocol deviations will be documented. State if no missing data adjustment will be used. Where multiple imputation is utilised the methods used to test the validity of the different assumptions will be described, either here or under the sensitivity analyses section.

A qualitative assessment will be made of the amount and pattern of any missing data and how this may affect the feasibility and or design of the main study.

8.5 Pre-specified Subgroup Analysis

Any pre-specified subgroup analyses will be described, together with the justification for relevance and importance and will include methods of analysis and presentation of the results. It is recommended that subgroup-treatment interaction methods are used with presentation using forest plots. This is usually included in the protocol, but additional subgroup analyses may be added before the final datalock following the blinded analysis or publication of other trials/research.

No subgroup analyses are planned

8.6 Treatment by Centre Interaction

Consistency of effect will be assessed across the 6 centres by informal examination of the within centre effects. There will be limited capacity to investigate these formally and it is noted that such centre effects are expected by chance.

8.7 Sensitivity Analysis

Describe any analysis utilised to check the robustness of the results. This may include repeating the primary analysis for different patient populations as defined in section 8.

No sensitivity analyses are planned.

Note: Sensitivity analyses for checking validity of multiple imputation assumptions should be detailed either here or in Section 9.4.

9. ANALYSIS TO ADDRESS SECONDARY AIMS

A list of the secondary outcomes will be provided with a separate section for each outcome with full definitions and analyses methods, together with details of the populations to be analysed and presentation of results will be provided. Sometimes the statistical method will be described first in full and then for each secondary outcome the method will be referred to in brief.

For peer review only - http://bmjopen.bmj.com/site/about/guidelines.xhtml

9.1 Evaluation/Definition of Secondary Outcomes (where applicable)

Not applicable.

9.2 Statistical Methods Used for Analysis of Secondary Outcomes

Statistical methods used to compare groups for secondary outcome(s); Methods for additional analyses, such as subgroup analyses and adjusted analyses.

The primary aim of this feasibility study is to assess the recruitment rate. Secondary outcomes will be collected;

Disability Rating Index (DRI), Dementia Quality of Life Measure (DEMQoL), EuroQol 5 Dimensions (5L) Score (EQ-5D-5L), self-efficacy, frailty, grip strength and complications. Methods for analysis of these variables are provided in Section 2.9. If a definitive trial is deemed feasible, then no analysis of these data will be undertaken and data will be carried over into the main study. However, if the main study is not deemed feasible, then outcome data will reported in the conventional manner.

9.1 Pre-specified Subgroups of Key Secondary Outcomes (where applicable)

Any pre-specified subgroup analyses of key secondary outcomes will be described, together with the justification for relevance and importance and will include methods of analysis, populations to be analysed and presentation of the results. This is usually included in the protocol, but additional subgroup analyses may be added before the final datalock following the blinded analysis or publication of other trials/research.

Not applicable.

9.2 Sensitivity Analysis of Key Secondary Outcomes (where applicable)

Describe any analysis utilised to check the robustness of the results for key secondary outcomes. See section 8.7.

Not applicable.

9.3 Health Economics and Cost Effectiveness

A summary from the protocol will be provided with reference to the relevant separate analysis plan (if applicable).

The statistician is not undertaking this analysis.

10. ADDITIONAL ANALYSES

10.1 Exploratory analyses

Pre-specified exploratory outcomes and analyses

Any outcomes and analyses pre-specified as exploratory in the protocol – detailed definition and analysis methodology will be provided or separate documents referred to together with who will perform these.

Additional Exploratory Analysis Not Specified Prior to Receiving Data

Any analyses not specified in the analysis protocol will be exploratory in nature and a significance level of 0.01 will be used to declare statistical significance. 99% confidence intervals will be presented.

10.2 Blinded analysis

Details of a blinded analysis will be undertaken prior to the final datalock will be provided, together with the rationale for undertaking this. If no blinded analysis to be undertaken, then state this.

No blinded analysis will be undertaken.

For peer review only - http://bmjopen.bmj.com/site/about/guidelines.xhtml
For peer review only

10.3 Meta-analyses (if applicable)

If meta-analysis is planned detail which trials will be included, which outcomes will be combined and whether random or fixed effects will be utilised. If this is under a separate protocol/analysis plan then reference this here.

Not applicable.

11. SAFETY ANALYSIS

Briefly outline the safety reporting from the protocol – this may be explicitly included as a secondary outcome – but include here. Describe how it will be summarised and compared (if applicable). Will adverse events/complications also be recorded, summarised and analysed – full details can be included here including dummy tables.

All complications and adverse events will be recorded. Complications will be classified as either: (a) unrelated to the trial protocol, (b) related systemic complications (including venous thromboembolic phenomena, death, pneumonia, urinary tract infection, blood transfusion, acute cerebrovascular incident, acute cardiac event, other) or (c) related local complications (superficial/deep infection, non/mal union, failure/removal/revision of metalwork, injury to adjacent structures such as nerves/tendons/blood vessels, other). The analysis will be conducted by intention to treat. Serious adverse events are defined as those that are fatal, life threatening, disabling or require hospitalisation or prolongation of hospitalisation.

12. APPENDIX: GLOSSARY OF ABBREVIATIONS

SAP Statistical Analysis Plan

DSMC Data and Safety Monitoring Committee

TSC Trial Steering Committee

CI Chief Investigator

13. DOCUMENT HISTORY

14. CHANGES FROM PREVIOUS VERSION OF SAP

A summary of key changes from earlier versions of SAP, with particular relevance to protocol changes that have an impact on the design, definition, sample size, data quality/collection and analysis of the outcomes will be provided. Include protocol version number and date.

Version number Issue date	Author of this issue	Protocol Version & Issue date	Significant changes from previous version together with reasons
V1.0_19Sep2016	NP	Protocol_V2.0_27Jul2106	Not applicable as this is the 1st issue
V2.0_2Nov2016	NP	Protocol_V2.0_27Jul2106	Paragraph added to describe comparison between EQ-5D and DEMQoL
			Add to or delete as required

For peer review only - http://bmjopen.bmj.com/site/about/guidelines.xhtml

CONSORT 2010 checklist of information to include when reporting a pilot or feasibility trial*

Section/Topic	Item No	Checklist item	Reported on page No
**Title and abstract**			
	1a	Identification as a pilot or feasibility randomised trial in the title	1
	1b	Structured summary of pilot trial design, methods, results, and conclusions (for specific guidance see CONSORT abstract extension for pilot trials)	2
**Introduction**			
Background and objectives	2a	Scientific background and explanation of rationale for future definitive trial, and reasons for randomised pilot trial	3
	2b	Specific objectives or research questions for pilot trial	2
**Methods**			
Trial design	3a	Description of pilot trial design (such as parallel, factorial) including allocation ratio	3-4
	3b	Important changes to methods after pilot trial commencement (such as eligibility criteria), with reasons	6
Participants	4a	Eligibility criteria for participants	3
	4b	Settings and locations where the data were collected	3
	4c	How participants were identified and consented	3
Interventions	5	The interventions for each group with sufficient details to allow replication, including how and when they were actually administered	4
Outcomes	6a	Completely defined prespecified assessments or measurements to address each pilot trial objective specified in 2b, including how and when they were assessed	4-5
	6b	Any changes to pilot trial assessments or measurements after the pilot trial commenced, with reasons	N/A
	6c	If applicable, prespecified criteria used to judge whether, or how, to proceed with future definitive trial	12
Sample size	7a	Rationale for numbers in the pilot trial	4
	7b	When applicable, explanation of any interim analyses and stopping guidelines	N/A
Randomisation:			
Sequence generation	8a	Method used to generate the random allocation sequence	4
	8b	Type of randomisation(s); details of any restriction (such as blocking and block size)	4
Allocation concealment mechanism	9	Mechanism used to implement the random allocation sequence (such as sequentially numbered containers), describing any steps taken to conceal the sequence until interventions were assigned	4

For peer review only

Implementation	10	Who generated the random allocation sequence, who enrolled participants, and who assigned participants to interventions	4
Blinding	11a	If done, who was blinded after assignment to interventions (for example, participants, care providers, those assessing outcomes) and how	4
	11b	If relevant, description of the similarity of interventions	4
Statistical methods	12	Methods used to address each pilot trial objective whether qualitative or quantitative	4-5
**Results**			
Participant flow (a diagram is strongly recommended)	13a	For each group, the numbers of participants who were approached and/or assessed for eligibility, randomly assigned, received intended treatment, and were assessed for each objective	6, fig 1
	13b	For each group, losses and exclusions after randomisation, together with reasons	6, fig 1
Recruitment	14a	Dates defining the periods of recruitment and follow-up	6
	14b	Why the pilot trial ended or was stopped	N/A
Baseline data	15	A table showing baseline demographic and clinical characteristics for each group	6-7
Numbers analysed	16	For each objective, number of participants (denominator) included in each analysis. If relevant, these numbers should be by randomised group	6
Outcomes and estimation	17	For each objective, results including expressions of uncertainty (such as 95% confidence interval) for any estimates. If relevant, these results should be by randomised group	6
Ancillary analyses	18	Results of any other analyses performed that could be used to inform the future definitive trial	6-12
Harms	19	All important harms or unintended effects in each group (for specific guidance see CONSORT for harms)	8
	19a	If relevant, other important unintended consequences	N/A
**Discussion**			
Limitations	20	Pilot trial limitations, addressing sources of potential bias and remaining uncertainty about feasibility	2, 12
Generalisability	21	Generalisability (applicability) of pilot trial methods and findings to future definitive trial and other studies	12
Interpretation	22	Interpretation consistent with pilot trial objectives and findings, balancing potential benefits and harms, and considering other relevant evidence	12
	22a	Implications for progression from pilot to future definitive trial, including any proposed amendments	12
**Other information**			
Registration	23	Registration number for pilot trial and name of trial registry	2
Protocol	24	Where the pilot trial protocol can be accessed, if available	9
Funding	25	Sources of funding and other support (such as supply of drugs), role of funders	2
	26	Ethical approval or approval by research review committee, confirmed with reference number	14

For peer review only

Citation: Eldridge SM, Chan CL, Campbell MJ, Bond CM, Hopewell S, Thabane L, et al. CONSORT 2010 statement: extension to randomised pilot and feasibility trials. BMJ. 2016;355.
*We strongly recommend reading this statement in conjunction with the CONSORT 2010, extension to randomised pilot and feasibility trials, Explanation and Elaboration for important clarifications on all the items. If relevant, we also recommend reading CONSORT extensions for cluster randomised trials, non-inferiority and equivalence trials, non-pharmacological treatments, herbal interventions, and pragmatic trials. Additional extensions are forthcoming: for those and for up to date references relevant to this checklist, see www.consort-statement.org.

For peer review only

BMJ Open

Intramedullary nails vs distal locking plates for fracture of the distal femur: results from the Trial of Acute Femoral Fracture Fixation (TrAFFix) randomised feasibility study and process evaluation

Journal:	*BMJ Open*
Manuscript ID	bmjopen-2018-026810.R2
Article Type:	Research
Date Submitted by the Author:	12-Mar-2019

Complete List of Authors:	Griffin, Xavier; University of Oxford Nuffield Department of Orthopaedics Rheumatology and Musculoskeletal Sciences, Oxford Trauma; Oxford University Hospitals NHS Foundation Trust<br>Costa, Matthew; University of Oxford Nuffield Department of Orthopaedics Rheumatology and Musculoskeletal Sciences, Oxford Trauma; Oxford University Hospitals NHS Foundation Trust<br>Phelps, Emma; University of Oxford Nuffield Department of Orthopaedics Rheumatology and Musculoskeletal Sciences, Oxford Trauma<br>Parsons, Nicholas; University of Warwick, Statistics and Epidemiology Unit<br>Dritsaki, Melina; University of Oxford Nuffield Department of Orthopaedics Rheumatology and Musculoskeletal Sciences, Oxford Clinical Trials Research Unit<br>Achten, Juul; University of Oxford Nuffield Department of Orthopaedics Rheumatology and Musculoskeletal Sciences, Oxford Trauma<br>Tutton, Elizabeth; University of Warwick, Warwick Medical School; Oxford University Hospitals NHS Foundation Trust<br>Lerner, Robin; University of Oxford Nuffield Department of Orthopaedics Rheumatology and Musculoskeletal Sciences, Kadoorie Centre<br>McGibbon, Alwin; Patient representative<br>Baird, Janis; University of Southampton, MRC Lifecourse Epidemiology Unit; University Hospital Southampton NHS Foundation Trust, NIHR Southampton Biomedical Research Centre

**Primary Subject Heading**:	Surgery
Secondary Subject Heading:	Qualitative research, Patient-centred medicine
Keywords:	Clinical trials < THERAPEUTICS, fractures, bone, fracture fixation, internal, randomized controlled trial, feasibility studies, femoral fractures

SCHOLARONE™ Manuscripts

TITLE

Intramedullary nails vs distal locking plates for fracture of the distal femur: results from the Trial of Acute Femoral Fracture Fixation (TrAFFix) randomised feasibility study and process evaluation

Authors

Xavier L Griffin[1,7*]

Matthew L Costa[1,7]

Emma Phelps[1]

Nicholas Parsons[5]

Melina Dritsaki[2]

Juul Achten[1]

Elizabeth Tutton[6,7]

Robin Lerner[1]

Alwin McGibbon[8]

Janis Baird[3,4]

on behalf of the TraFFix study collaborators

*corresponding author:

Email: xavier.griffin@ndorms.ox.ac.uk

Tel: +44 01865 223114

Address: Oxford Trauma, Kadoorie Centre, John Radcliffe Hospital, Oxford, OX3 9DU, UK

[1] Oxford Trauma, Nuffield Department of Orthopaedics, Rheumatology and Musculoskeletal Sciences (NDORMS), Kadoorie Centre, University of Oxford, Oxford, OX3 9DU, UK

[2] Oxford Clinical Trials Research Unit, NDORMS, University of Oxford, Windmill Road, Oxford, OX3 7LD, UK

[3] MRC Lifecourse Epidemiology Unit, Southampton General Hospital, University of Southampton, SO16 6YD, UK

[4] NIHR Southampton Biomedical Research Centre, University of Southampton and University Hospital Southampton NHS Foundation Trust, Southampton, UK

[5] Statistics and Epidemiology Unit, Warwick Medical School, University of Warwick, Coventry, CV4 7AL, UK

[6] University of Warwick, Warwick Research in Nursing, Warwick Medical School, Gibbet Hill Campus, Coventry CV4 7AL, UK

[7] Oxford University Hospitals NHS Foundation Trust, Trauma Research, Kadoorie Centre, John Radcliffe Hospital, Oxford OX3 9DU, UK

[8] Patient and public representative, Warwickshire, UK

Word count

4632/4000

For peer review only - http://bmjopen.bmj.com/site/about/guidelines.xhtml

ABSTRACT

Objectives: This feasibility study and process evaluation assessed the likely success of a definitive trial of intramedullary fixation with locked retrograde nails versus extramedullary fixation with fixed angle plates for fractures of the distal femur.

Design & setting: A multicentre, parallel, two-arm, randomised controlled feasibility study with an embedded process evaluation was conducted at seven NHS hospitals in England. Treatment was randomly allocated in 1:1 ratio, stratified by centre and chronic cognitive impairment. Participants, but not surgeons or research staff, were blinded to the allocation.

Participants: Patients 18 years and older with a fracture of the distal femur, who their surgeon believed would benefit from internal fixation, were eligible to take part.

Interventions: Patients were allocated to receive either a retrograde intramedullary nail or an anatomical locking plate.

Outcomes: The primary outcomes for this feasibility study were the recruitment rate and completion rate of the EQ-5D-5L at four months post-randomisation. Baseline characteristics, disability rating index, quality of life scores, measurements of social support and self-efficacy, resource use, and radiographic assessments were also collected. The views of patients and staff were collected during interviews.

Results: Recruitment and data completion were lower than expected. 23 of 82 eligible patients were recruited (nail; 11, plate; 12). The recruitment rate was estimated as 0.42 (95% CI; 0.27 to 0.62) participants per centre-month. Data completeness of the EQ-5D-5L at four-months was 61 percent (95% CI; 43 to 83%). The process evaluation demonstrated that the main barriers to recruitment were variation in treatment pathways across centres, lack of surgeon equipoise and confidence in using both interventions, and newly formed research cultures that lacked cohesion.

Conclusions: A modified trial design, with embedded recruitment support intervention, comparing functional outcome in cognitively intact adults who have sustained a fragility fracture of the distal femur is feasible.

Registration: ISRCTN92089567.

299/300 words

Strengths and limitations of this study:

- Mixed methods feasibility study including a randomised trial and process evaluation
- Conducted at seven hospitals, including several major trauma centres
- Included qualitative interviews with participants, researchers and surgeons
- The study randomised only a small number of patients

Keywords: Femur; femoral fractures; fractures, bone; fracture fixation, internal; Randomized controlled trial; feasibility studies;

Disclaimer: This report presents independent research commissioned by the National Institute for Health Research (NIHR). The views and opinions expressed by the interviewees in this publication are those of the interviewees and do not necessarily reflect those of the authors, those of the NHS, the NIHR, MRC, CCF, NETSCC, the Health Technology Assessment programme or the Department of Health.

For peer review only - http://bmjopen.bmj.com/site/about/guidelines.xhtml

INTRODUCTION

Background

Distal femur fractures have an incidence of 10 per 100,000 and have a bimodal distribution with age, with approximately 85% sustained by older patients after a fall from a standing height and the remainder sustained by patients after major trauma. [1 2]

There is currently no consensus about the best way to treat these fractures. A recent Cochrane review identified few trials in this area, many using outdated implants or with important methodological limitations, and suggested that future patients' functional outcomes could be improved by a well-designed randomised controlled trial comparing modern treatments.[3]

In a multicentre retrospective study to review current practices, we found that the treatments commonly used in the UK are fixation with a locked retrograde nail (nail) and fixation with an anatomical, angular stable plate (plate).[4] Nails offer minimal disruption of the fracture site, a potential mechanical benefit from a device close to the axis of the femur, and stimulated blood supply from reaming of the intramedullary canal.[5] On the other hand fixed-angle plates are specifically designed for osteoporotic bone, and the bone-plate constructs have excellent biomechanical properties.[6] However plates require larger incisions and are more expensive than nails. Limited evidence suggests that there may be a benefit of using nails over plates in terms of patients' quality of life, with small studies identifying differences of 0.09 and 0.1 in EQ-5D-5L-derived utility scores at one year.[7 8]

This study of fracture fixation with nails compared with plates aimed to assess the feasibility of a future definitive trial, and perform a process evaluation to understand the generalisability and likely success of a future trial.

METHODS

Approval and oversight

The Wales Research Ethics Committee 5 approved the study (ref: 16/WA/0225). The trial protocol is available in the supplemental material and has been previously published.[9] The conduct of the study was overseen by independent data safety monitoring and trial steering committees.

Inclusion criteria

Patients aged 18 and over who were admitted to one of seven participating NHS hospitals in England with a fracture of the distal femur, which their treating surgeon believed would benefit from internal fixation were potentially eligible to take part. Patients with a loose knee or hip arthroplasty requiring revision, or an arthroplasty or pre-existing femoral deformity that precluded nail fixation, were excluded.

All potentially eligible participants for TrAFFix and their carers, as well as all staff involved with the study at participating centres, were eligible to be interviewed for the process evaluation.

Consent

Where patients had capacity to consent to be involved in the study prior to surgery, this was sought by hospital research teams. For patients lacking capacity to provide informed consent, the hospital research teams sought agreement from an appropriate consultee, either a nominated consultee (e.g. treating surgeon who was not part of the research team) or a personal consultee such as the next of kin. Where a consultee gave agreement prior to surgery and the patient regained capacity following surgery, consent for continued participation in the study was sought from the patient.

For peer review only - http://bmjopen.bmj.com/site/about/guidelines.xhtml

During the consent process patients and carers indicated their willingness to be approached for interviews for the process evaluation. Consent for interviews was sought at the start of each interview by a researcher from the University of Oxford.

Randomisation and blinding

Following consent from the patient or agreement from an appropriate consultee, participants were randomised prior to surgery to receive either nail or plate in a 1:1 ratio using an online randomisation system. The trial statistician generated the allocation sequences. Allocations used fixed blocks of size four and were stratified by centre to ensure any clustering effect related to centres was distributed across arms, and by the presence of chronic cognitive impairment to ensure this important effect modifier was distributed evenly across groups.

Participants were not informed of their allocation during the trial but were able to request to be informed of their allocation at the end of the study. Surgeons and research staff were not blinded. Radiographs were reviewed by independent assessors, however due to the presence of the implants they were also not blinded.

Sample size

There was no sample size calculation performed, instead it was estimated that recruiting for 52 centre-months with an average of one patient per centre-month would allow an estimation of the recruitment rate of a future definitive trial with a 95% confidence interval of 0.73-1.28.

Interventions

Potential participants were reviewed in daily trauma meetings and operated on at the next available theatre time. Patients were assessed and received anaesthesia, analgesia and prophylactic antibiotics as per local practices. Preparation, positioning and reduction of the fracture, details of incision and approach and supplementary fixation with wires or screws were left to the discretion of the treating surgeon.

For patients allocated a nail, fixation of the fracture was achieved with a proximally and distally locked nail that spanned the entire diaphysis of the femur. All nails were introduced retrograde through the knee joint. For patients allocated plates, fixation of the fracture was achieved with anatomical distal femoral locking-plate and screws, defined as those in which at least one fixed angle locking screw was placed distal to the fracture.

Outcome measures

The primary outcomes for this feasibility study were the recruitment rate and completion rate of the EQ-5D-5L[10] at four months post-surgery.

Screening

Participating hospitals were asked to record screening data for all patients admitted with a fracture of the distal femur during the study period. In addition, an orthopaedic surgeon at each participating hospital retrospectively reviewed admissions data and re-screened all admissions during the recruitment period. Similarly, we requested admissions data from the Trauma Audit Research Network (TARN)[11] in order to monitor the accuracy of screening data from research staff and orthopaedic trainees. These data included admission date, diagnosis and treatment received.

Clinical and patient-reported outcomes

Baseline recordings were made of patient characteristics, grip strength[12] and Rockwood frailty scores[13] as predictors of frailty, the level of social support available to patients using the medical outcomes survey (MOS) social support survey[14], and patient activation using a general self-efficacy questionnaire.[15]

For peer review only - http://bmjopen.bmj.com/site/about/guidelines.xhtml

EQ-5D-5L, a generic five-level health utility instrument used to measure health-related quality of life across five domains of mobility, self-care, usual activities, pain and depression was measured at baseline (pre-injury and contemporary) and 6-weeks and 4-months post-injury. Dementia quality of life scores (DEMQoL, and DEMQoL-proxy when completed by a carer) were recorded for patients with chronic cognitive impairment, along with proxy-reported EQ-5D-5L scores.[16] The disability rating index (DRI)[17], which measures disability from 0-100 across 12 items, was recorded at each time point for patients without cognitive impairment.

Radiographs taken at six-weeks post-injury were assessed independently for evidence of loss of fixation, varus or valgus deformity >5° (deformity in the coronal plane), recurvatum/procurvatum >10° (deformity in the sagittal plane), and shortening of the femur >1cm. Related medical complications occurring during the trial period were recorded.

In order to assess the feasibility of a health economic assessment in a larger trial, resource use data were measured using baseline CRFs (treatment costs) and a patient completed questionnaire at four months post-injury. Estimation of unit costs followed recent National Institute for Health and Care Excellence (NICE) guidelines on costing health and social care services[18] using the NHS reference costs[19] and NHS Supply Chain Catalogue[20] and inflated to 2017/18 prices using the NHS hospital & community health services (HCHS) index for health service resources.[21]

Analysis

Analyses were conducted in R (V3.3.0, R Foundation for Statistical Computing, Vienna, Austria. https://www.R-project.org/) and the statistical analysis plan is available as supplemental material. The recruitment rate was estimated using Poisson regression analysis, and the completeness of EQ-5D-5L was calculated as the percentage of randomised participants completing the EQ-5D-5L questionnaire. Given the small study group, treatment effects were not estimated.

Responses from the EQ-5D-5L health classifications were converted into an overall score using a published utility algorithm for the population of the United Kingdom.[22]

Process evaluation

A process evaluation was performed to assess implementation, mechanisms of impact and context of the interventions, in line with MRC guidelines.[23] In order to inform the development of a definitive trial, our process evaluation also examined the implementation of study processes. The evaluation of implementation included the reach and fidelity of screening and the acceptability of the interventions and study procedures. To understand how the interventions produced change in outcomes, we identified relevant intermediate outcomes that might be associated with the effect of the interventions on the primary outcomes of interest. The context in which the intervention is delivered will influence outcomes. A particular focus in our evaluation of context considered contextual similarities and differences between the participating centres, which might have influenced the delivery of study procedures.

We used a mixed methodology approach using a variety of data sources in the process evaluation including qualitative interviews with patients (n=9), carers (n=2) and staff (n=24), screening logs, national datasets and a one-day workshop with patients and public representatives. Interviews were based on a topic guide that included participants' experience of taking part in TrAFFix and the experiences of staff recruiting to TrAFFix. Interviews were audio recorded and transcribed verbatim; data were managed using NVivo 10 (QSR International, Warrington, UK) Data were analysed inductively using a thematic analysis. Quantitative data were summarised using standard descriptive statistical techniques. The analyses were integrated using the framework of a process evaluation and summarised in tables.

For peer review only - http://bmjopen.bmj.com/site/about/guidelines.xhtml

Patient & public involvement

A public representative was a member of the trial management committee, and was a co-applicant on the funding application. Patients and public representatives were consulted on the design of patient-facing documents via the UK Musculoskeletal Trauma PPI mailing list. Patient and public representatives were also invited to a meeting to develop a logic model.

Important changes to the study design

Patient's age (50 years and older) was initially used as an inclusion criterion as a surrogate for likely fragility fracture. However following review of the screening data, the independent steering committee requested that the minimum inclusion age be lowered to 18 and a mechanistic determination of fragility fracture, using mechanism of injury, be assessed as part of the feasibility study to explore the impact of this modification on recruitment rate.

Instead of utilising the algorithm by Herdman et al[24] to convert EQ-5D-5L to EQ-5D-3L utility as mentioned in the health economics analysis plan, the NICE-approved method of calculating utility values was performed using the algorithm provided by Van Hout et al.[22]

RESULTS

Recruitment & baseline

Recruitment opened in October 2016 and closed in August 2017, with a total of 54.8 centre-months of recruitment measured. Twenty three participants were randomised into the study, giving an estimated recruitment rate of 0.42 (95% CI: 0.27 to 0.62) participants per centre-month. The flow of participants through the trial is described in Figure 1.

Differences in surgical practices were identified across participating hospitals. For example the numbers of patients treated non-operatively varied between 0 and 18 patients (0% to 44% of patients screened at each centre), and the proportion of patients excluded due to surgeon preference varied between 40% and 70%. A breakdown of recruitment and reasons for exclusion for each site is given in supplemental Table S1.

Of the 173 of patients screened, the most common reason that patients were not recruited were surgeon preference for either a nail or plate (23%; 39/173), presence of a pre-existing arthroplasty (19%; 33/173) and a decision to treat the patient non-operatively (18%; 31/173). The number of patients treated non-operatively was higher than anticipated, but confirmed by data from retrospective screening of all admissions, and by data from TARN. The baseline characteristics of randomised participants in each group were well matched and are described in Table 1. Following the change of the minimum inclusion age to 18 years old one patient under 50 was randomised, this patient was aged 40-45 years, was injured by fall from over 2m, and had no reported bone health problems.

Table 1 Baseline variables and treatment details

		Nail (n=11)	Plate (n=12)
Baseline variables			
Age (years); mean (sd; n)		70.1 (13.6; 10)	78.7 (14.9; 11)
Grip strength (kg); mean (sd; n)		16.8 (4.6; 4)	18.7 (1.2; 3)
Rockwood frailty score (1-9); mean (sd; n)		3.7 (2.2; 10)	4.5 (2.1; 12)
Pre-Op AMTS	< 7; n (%)	3 (27.3)	1 (8.3)
	7+; n (%)	4 (36.4)	7 (58.3)
Post-Op AMTS	< 7; n (%)	0 (0.0)	1 (8.3)

For peer review only - http://bmjopen.bmj.com/site/about/guidelines.xhtml

	7+; n (%)	2 (18.2)	3 (25.0)
Gender	Female; n (%)	7 (63.6)	9 (75)
	Male; n (%)	4 (36.4)	3 (25)
Ethnicity	Indian; n (%)	1 (9.1)	0 (0)
	White; n (%)	9 (81.8)	11 (91.7)
Diabetes	No; n (%)	5 (45.5)	9 (75)
	Yes; n (%)	5 (45.5)	2 (16.7)
Regular Smoker	No; n (%)	9 (81.8)	10 (83.3)
	Yes; n (%)	1 (9.1)	1 (8.3)
Living Arrangement	Care home; n (%)	1 (9.1)	1 (8.3)
	Live alone; n (%)	3 (27.3)	4 (33.3)
	Live with relatives; n (%)	1 (9.1)	1 (8.3)
	Live with wife/husband/ partner; n (%)	5 (45.5)	5 (41.7)
Treatment details			
Time from admission to surgery (days); median (IQR)		2 (1 to 3)	2 (1 to 3)
Mechanism of injury	Fall from <2m (%)	11 (100%)	12 (100%)
Fracture Classification*	A1; n (%)	7 (63.6)	5 (41.7)
	A2; n (%)	3 (27.3)	2 (16.7)
	A3; n (%)	0 (0)	1 (8.3)
	B1; n (%)	0 (0)	1 (8.3)
	C2; n (%)	1 (9.1)	1 (8.3)
	C3; n (%)	0 (0)	2 (16.7)
Periprosthetic Fracture	No; n (%)	8 (72.7)	9 (75)
	Yes; n (%)	3 (27.3)	3 (25)
ASA Grade	1 or 2; n (%)	3 (27.3)	4 (33.3)
	3 or 4; n (%)	8 (72.8)	6 (50)
Method Fixation	Nail; n (%)	10 (90.9)	1 (8.3)
	Plate; n (%)	0 (0)	11 (91.7)
	Other; n (%)	1 (9.1)	0 (0)
Grade Operating Surgeon	Consultant; n (%)	7 (63.6)	8 (66.7)
	SAS; n (%)	1 (9.1)	1 (8.3)
	ST3+; n (%)	3 (27.3)	3 (25)

Notes:
Where totals do not sum to column totals then it indicates that there were missing data
Reported percentages are based on the full population
* AO/OTA classification of peri-articular fractures

Clinical outcomes

Of the 23 participants randomised the EQ-5D-5L questionnaire was completed by 20 (87%, 95% CI: 65 to 97%), 15 (65%, 95% CI: 43 to 83%) and 14 (61%, 95% CI: 39 to 80%) patients at baseline, six weeks and four months respectively. There were insufficient numbers of patients to draw any meaningful conclusions about treatment effects between groups.

The mean scores across each time point for EQ-5D-5L, DRI and DEMQoL are reported in Table 2. Independent assessors at participating sites were asked to review any radiographs taken at 6-week follow-up appointments, the results of assessments are described in Table 3, along with a summary of the post-operative complications reported during the four-month follow-up period.

For peer review only - http://bmjopen.bmj.com/site/about/guidelines.xhtml

Power analysis

We estimated the sample size necessary to power a definitive study using a patient reported outcome such as DRI. Using a minimum important difference of 8 points and standard deviation of 21 points defined in previous studies and in line with values reported here,[25] and an attrition rate of 30%, a sample of 210 participants per group (420 in total) would be needed to reject the null hypothesis with probability (power) 0.9 and type I error rate of 5%.

Table 2 Patient reported outcome measures

	Nail (n=11)	Plate (n=12)
Pre-injury: mean (sd; n)		
EQ-5D-5L	0.59 (0.29; 10)	0.56 (0.22; 10)
DRI	45.4 (36.7; 9)	67.5 (10.1; 10)
DEMQoL Proxy	87 (0; 1)	-
DEMQoL Self	-	78.2 (0; 1)
Post-injury: mean (sd; n)		
EQ-5D-5L	-0.05 (0.28; 10)	-0.04 (0.16; 10)
DRI	86.5 (15.5; 9)	92.5 (5.3; 10)
DEMQoL Proxy	97 (0; 1)	-
DEMQoL Self	-	84 (0, 1)
6-weeks: mean (sd; n)		
EQ-5D-5L	0.35 (0.30; 10)	0.05 (0.16; 5)
DRI	78.0 (16.9; 9)	87.3 (3.1; 5)
DEMQoL Proxy	87 (0; 1)	-
DEMQoL Self	-	-
4-months: mean (sd; n)		
EQ-5D-5L	0.38 (0.36; 9)	0.37 (0.41; 5)
DRI	60.9 (23.1; 8)	82.8 (2.9; 4)
DEMQoL Proxy	89 (0; 1)	-
DEMQoL Self	-	-

Note
- indicates no data were available

For peer review only

Table 3 Summary of 6-week post-operative radiograph review and reported complications reported up to 4-months

	Nail (n=11)	Plate (n=12)
6-week radiographic assessment: n (%)		
Loss of fixation	1 (9.1)	1 (8.3)
Varus/Valgus > 5º	10 (90.9)	5 (41.7)
Recurvatum > 10º	1 (9.1)	0 (0)
Procurvatum >10º	1 (9.1)	0 (0)
Shortening >1cm	2 (18.2)	0 (0)
Post-operative complications: n (%)	**Nail**	**Plate**
Wound infection	0 (0)	0 (0)
Venous thromboembolism	0 (0)	0 (0)
Pneumonia	1 (9.1)	0 (0)

For peer review only - http://bmjopen.bmj.com/site/about/guidelines.xhtml

Urinary tract infection	1 (9.1)	0 (0)
Cerebrovascular accident	0 (0)	0 (0)
Myocardial infarction	1 (9.1)	0 (0)
Blood transfusion	2 (18.2)	2 (16.7)
Mal-union	0 (0)	0 (0)
Failure of fixation	0 (0)	1 (8.3)

Process evaluation

A summary of the complete findings of the process evaluation is presented in the supplemental Table S2.

Implementation

Reach of screening

During the 10-month recruitment period, 91 patients were screened and recorded on screening logs, usually by a research associate (RA). Of these, 54 were eligible to participate in the study. Retrospective review of screening by orthopaedic surgeons at each participating centre found a further 82 unscreened patients with distal femur fractures including 31 eligible patients who were not included on the screening logs. The number of patients added after rescreening varied by centre with between 0 and 34 patients added. At interview, staff emphasised that there were few patients eligible for the study, suggesting that they were unaware that potentially eligible patients were being missed.

> *"With the TRAFFIX study, well we just never had the numbers coming through and I think the only two that we actually missed were during weekends and I think one was when I was on annual leave"*

Staff (RA) 19

The differing levels of experience of the research teams and research culture, evident in the approaches to screening, recruitment and data collection, within the centres may explain differences in the number of patients missed from screening logs. In some centres, not all surgeons within the team screened and identified eligible patients, while in other centres patients deemed ineligible might not have been recorded in the screening logs.

Fidelity of screening

Interviews with research staff revealed that several centres relied on research teams to screen patients as the clinical team tended not to notify the research team of eligible patients. Screening was facilitated by the presence of the research team in the daily trauma meeting, as they were able to prompt the clinical teams to consider whether patients were eligible. The attendance of the research team in this meeting varied between the centres due to staffing and other commitments. Additionally, some centres found it difficult for the clinical team to keep the study in mind as potentially eligible patients were infrequent.

Acceptability of the interventions

At interview, some staff described an unwillingness from surgeons to randomise patients. They felt that some surgeons lacked equipoise and believed that only one method of fixation was appropriate for any given fracture.

> *"you would never do the other thing for this fracture"*

Staff (Surgeon) 8

For peer review only - http://bmjopen.bmj.com/site/about/guidelines.xhtml

However, some staff reported that surgeons' preferences for one of the two interventions were based on their surgical skill and experience

> *"Some surgeons, they only know how to do a plate and what you will see is plate plate plate plate so it comes down to also what you are good at sometimes and you don't want something else."*

Staff (Surgeon) 16

In contrast some surgeons were able to accept community equipoise (uncertainty within the expert medical community)[26], accepting their less preferred intervention if required.

Acceptability of the study procedures

Consent

Participants and carers were rarely able to describe the study in their own words but they tended to recall or recognise the interventions.

> *"Yes, because one is on the outside and one is a rod through the middle isn't it"*

Participant 4

Several participants described struggling to engage with information about the study around the time of their surgery explaining that they were not "in a fit state" (participant 5) to ask questions or were "trying to so hard to be normal (participant 3)."

Interviews with staff showed that they described the study to patients in simple terms. Randomisation, for example was explained using phrases such as '50/50' or 'computer decides.' When explaining the study, staff also emphasised that both treatments are routinely used and that their surgeon was happy for them to receive either intervention, as they believed these were important to patients.

> *"They're going to have surgery anyway and then like I said if you say we don't know which the better one is and the surgeon is happy, I think is the key point is that the surgeon is happy for them to be part of the study"*

Staff (RA) 1

The majority of staff found involving relatives in the discussion about the study to be helpful as it enabled patients to be supported in their decision-making. In contrast, a minority of staff felt, that it was easier to consent patients to studies when their relatives were not involved as they could be protective and involving them could lead to more potential participants declining.

> *"Sometimes you can find it easier if you approach them to try and time it around their visiting hours and so they've got a relative with them and so you can kind of talk to them as a family and a lot more patients feel more comfortable with that"*

Staff (RA) 7

Two of seven patients who were entered into the study under nominated consultee agreement withdrew when they were approached to consent to continue in the study after surgery. One of these seven patients participated in an interview. She acknowledged that she "*wasn't sufficiently with it to give an opinion*" (participant 1) prior to surgery.

Randomisation

The majority of staff found randomisation was acceptable to patients, explaining that it was considered acceptable because of the explanation that the surgeon thought both treatments were

For peer review only - http://bmjopen.bmj.com/site/about/guidelines.xhtml

appropriate. Two members of staff, in contrast, found patients disliked randomisation and wanted their surgeons to choose the treatment that was most appropriate for them.

When randomisation was described to participants during their interview, they accepted it or seemed indifferent towards it. Several participants, however, demonstrated therapeutic misconception: "*when a research subject fails to appreciate the distinction between the imperatives of clinical research and of ordinary treatment, and therefore inaccurately attributes therapeutic intent to research procedures*".[27] They believed that they or their relative would receive the most appropriate intervention for them.

> *"I think I prefer the one they done. I mean I didn't have a say in it, they decided what they thought was best, you know but I preferred the one they done definitely."*

Participant 6

Blinding

A minority of participants were told of their treatment allocation following surgery or when they returned for follow-up appointments. Some staff felt that blinding participants to their treatment allocation would be difficult as patients could see their allocation in the letter sent to their GP or X-rays, or they may be told by another member of staff. Some staff wanted to tell patients who asked which intervention they were allocated as one explained, "*I wouldn't be particularly comfortable, on a personal level... I would want to tell them what they have had*" (surgeon 8).

Case Reporting Forms (CRFs)

Research staff found that completing CRFs with the patient group could be challenging, as most patients were frail and often had long-term medical problems. They felt the questionnaires were lengthy and tiring for patients who often needed support completing them. They tended to ask the questions as part of a conversation to pull out the information for the questionnaire as they found some patients were unable to express their experience using the responses in the scales. Several RAs felt that where patients were wary of participating in studies this was usually due to the burden of follow-up rather than a dislike of randomisation or preference for one of the interventions.

> *"Some of the questions you have to try to put it into different words for them to understand, some of the questions don't make any sense to them"*

Staff (RA) 2

Mechanisms of impact

We identified intermediate outcomes that might be associated with the effect of the interventions on the primary outcomes of interest. Associations between the factors identified here, the interventions and the outcome of interest will assessed in a definitive trial. Factors that might influence patients' recovery after a distal femur fracture were identified by patients and public representatives and from data collected from interviews with participants and staff. Factors fell into three groups: existing factors relating to the characteristics of patients including their age, cognition and psychological factors such as self-efficacy; injury and treatment factors including the injury itself and factors relating to the surgeon and centre in which the treatment was delivered; post-discharge factors including rehabilitation provided, living arrangements and availability of support at home both from social care and from family. These factors are presented in supplemental Table S3.

Context

Contextual differences between the participating centres may have led to differences in how the study was implemented. Surgeons' working in departments where several trials were running may have been more engaged in research and more inclined to participate.

For peer review only - http://bmjopen.bmj.com/site/about/guidelines.xhtml

"However, that's changing and we've got a lot more of the consultants on board now and a lot more involved in the trial… there's consultants that are running trials in hand orthopaedics, the more elective side of things but also do the trauma lists and so are getting more involved with what's going on in research and therefore they're more happy to be involved in it and identifying the patients and things like that."

Staff (RA) 4

Some staff felt that differences in the size of the research team, their workload and working hours could influence how the study was implemented. For example, the presence of RAs in the trauma meeting could facilitate screening but required RAs to start work before eight, which was not possible in every centre.

I'm keen to see what is going to happen when we do get the other research nurses going to the trauma meeting because I feel that that's going to make a big difference and I think our recruitment will increase quite a lot but I might be completely wrong in thinking that.

Staff (RA) 4

I think if we didn't have enough studies running to have a fully funded research team then it would be tricky.

Staff (surgeon) 20

Meanwhile, some centres relied on one or part time RAs. This can result in patients being missed either when the RA was away or because they were struggling with their workload.

There was nobody in [to recruit] this Friday just gone and it can happen because I've got to be everywhere.

Staff (RA) 16

DISCUSSION

We have reported a randomised feasibility study, and to our knowledge the first with an embedded process evaluation, to plan a future trial of the treatment of patients with a fracture of the distal femur. We found the number of eligible patients screened closely matched the original estimate of 1.5 patients per centre-month. The recruitment rate into the trial, however, fell substantially short of this. The process evaluation identified very significant surgeon-related barriers to recruitment into the study. Research cultures were found to vary widely between centres; a positive culture was a strong predictor of improved recruitment performance.

This study has challenged many of the assumptions which underpinned the development of the trial protocol. The recruited population had a much lower prevalence of cognitive impairment, less frailty and was physiologically fitter than the previously characterised incident fracture population.[4] Surgeons were actively selecting fitter patients for operative treatment in a way that we had not anticipated. The eligible population is therefore somewhat different than we originally expected and hence the design for the definitive trial can be modified in important ways.

It is clear that research staff can and do miss potentially eligible patients if the working practices at hospitals do not facilitate full integration of the research staff into the clinical team. Although the specifics vary between hospitals, crucial decision points exist within the clinical process, such as daily trauma meetings, into which research staff must be integrated in order to be able to engage clinicians in research studies.

Surgeons reported a lack of their confidence in the management of this relatively uncommon fracture, the operative treatment of which is perceived to be technically demanding. The combination of this common anxiety and some individuals' lack of equipoise, posed a very significant

For peer review only - http://bmjopen.bmj.com/site/about/guidelines.xhtml

barrier to recruitment into this study. Similar challenges have been previously identified in other surgical trials.[28 29] Interventions that have included training on community equipoise have been successful when targeted at multidisciplinary teams and surgeons, improving both clinicians' confidence and potentially recruitment rates,[30 31] though definitive evidence is lacking.[32] We propose that a modified protocol that includes an intervention to promote awareness and understanding of the existing community equipoise will be able to overcome these surgeon-related barriers to recruitment.

Our findings demonstrate that the trial design tested in this feasibility study is unlikely to be successful on a larger scale. However, the recruitment rate of 0.42 participants per centre-month is in line with that reported by the FixDT trial, which tested similar interventions for treating fractures of the tibia and delivered to target and budget.[25]

We propose a modified definitive trial with a further internal pilot to confirm recruitment rates; this will be based upon the findings of this feasibility study, with an integrated recruitment intervention to support surgeons in their decision-making to recruit participants.

A future study may choose to exclude the small number of patients with chronic cognitive impairment to simplify trial procedures for research staff or reduce the number and length of questionnaires to reduce the burden on participants and improve retention rates. Similarly a future study may consider a trial design that accounts for surgeons' treatment preferences during the allocation of treatment to improve surgeon participation and willingness to randomise.

We conclude that a trial comparing functional outcome in cognitively intact patients aged 18 years or older who have sustained a fragility fracture of the distal femur, defined as a fall from standing height, treated with a nail or plate, is feasible with appropriate mitigation strategies incorporated to avoid failure.

SUPPLEMENTARY FILES

Study protocol

Statistical Analysis Plan

Supplementary tables 1-3

ACKNOWLEDGEMENTS

Contributions of authors

Xavier L Griffin (Associate Professor of Trauma Surgery) contributed to the conception, design, conduct and reporting of the study.

Matthew L Costa (Professor of Orthopaedic Trauma Surgery) contributed to the conception, design and reporting of the study.

Emma Phelps (Postdoctoral Research Associate in Mixed Methods Research) contributed to the design, conduct and analysis of the process evaluation and the reporting of the study.

Nicholas Parsons (Associate Professor of Medical Statistics, Statistics and Epidemiology) contributed to the design, conduct, analysis and reporting of the study.

Melina Dritsaki (Senior Health Economist) contributed to the design and conduct of the study.

Juul Achten (Scientific Officer Oxford Trauma) contributed to the conception, design, conduct and reporting of the study.

For peer review only - http://bmjopen.bmj.com/site/about/guidelines.xhtml

Elizabeth Tutton (Senior Research Fellow) contributed to the design, conduct and analysis of the process evaluation and the reporting of the study.

Robin Lerner (Clinical Trial Manager) contributed to the conduct and reporting of the study.

Alwin McGibbon (Patient & Public Representative) contributed to the design, conduct and reporting of the study.

Janis Baird (Professor of Public Health and Epidemiology) contributed to the conception, design, conduct, reporting and analysis of the study.

Principal collaborators

Mr David Noyes, John Radcliffe Hospital, Oxford University Hospitals NHS Foundation Trust

Prof Peter Giannoudis, Leeds General Infirmary, The Leeds Teaching Hospitals NHS Trust

Prof Ben Ollivere, Queen's Medical Centre, Nottingham University Hospitals NHS Trust

Ms Charlotte Lewis, Queen Alexandra Hospital, Portsmouth Hospitals NHS Trust

Mr Andrew McAndrew, Royal Berkshire Hospital, Royal Berkshire NHS Foundation Trust

Mr Haroon Majeed, Mr Damian McClelland, Royal Stoke Hospital, University Hospitals of North Midlands NHS Trust

Mr Ashwin Kulkarni, Leicester Royal Infirmary, University Hospitals of Leicester NHS Trust

Funding

This project was funded by the Health Technology Assessment programme (HTA) (project number 15/59/22) and will be published in full in the NIHR HTA Journal. Further information available at: https://www.journalslibrary.nihr.ac.uk/programmes/hta/155922/#/. This work was also supported by NIHR Oxford Biomedical Research Centre.

The funders had no role in the design and conduct of the study; collection, management, analysis, and interpretation of the data; preparation, review, or approval of the manuscript; and decision to submit the manuscript for publication.

Data sharing

All data requests should be submitted to the corresponding author for consideration. Please note exclusive use will be retained until the publication of major outputs. Access to anonymised data may be granted following review.

Competing interests

Matthew Costa is a member of the UK NIHR HTA General Board. Janis Baird received funding from Danone Nutrica Early Life Nutrition for a specific research study which aims to improve the nutrition and Vitamin D status of pregnant women and is collaborating with Iceland Foods Ltd to evaluate the impact fruit and vegetable availability on diet.

REFERENCES

1. Court-Brown CM, Caesar B. Epidemiology of adult fractures: A review. *Injury* 2006;37(8):691-7. doi: 10.1016/j.injury.2006.04.130
2. Martinet O, Cordey J, Harder Y, et al. The epidemiology of fractures of the distal femur. *Injury* 2000;31 Suppl 3:C62-3.
3. Griffin XL, Parsons N, Zbaeda MM, et al. Interventions for treating fractures of the distal femur in adults. *Cochrane Database Syst Rev* 2015;8:CD010606. doi: 10.1002/14651858.CD010606.pub2

For peer review only - http://bmjopen.bmj.com/site/about/guidelines.xhtml

4. Smith JRA HR MJ, Morrison R, Yip G, Aquilina A, Kelly M. Distal femoral fractures: The size of the problem in the UK. *Orthopaedic Trauma Society* 2013
5. Browner BD EC. The science and practice of intramedullary nailing. *Philadelphia* 1987;Lea & Febiger
6. Miller DL, Goswami T, Prayson MJ. Overview of the locking compression plate and its clinical applications in fracture healing. *J Surg Orthop Adv* 2008;17(4):271-81.
7. Tornetta P, Egol KA, Ertl JP, et al. Locked plating versus retrograde nailing for distal femur fractures: A multicentre randomized trial (Abstract 39). Orthopaedic Trauma Association Annual Meeting. Phoenix, Arizona, USA, 2014.
8. Hoskins W, Sheehy R, Edwards ER, et al. Nails or plates for fracture of the distal femur? *The Bone & Joint Journal* 2016;98-B(6):846-50. doi: 10.1302/0301-620x.98b6.36826
9. Griffin XL, Costa ML, Achten J, et al. Trial of Acute Femoral Fracture Fixation (TrAFFix): study protocol for a randomised controlled feasibility trial. *Trials* 2017;18(1):538. doi: 10.1186/s13063-017-2265-0
10. Brooks R. EuroQol: the current state of play. *Health Policy* 1996;37(1):53-72.
11. Lecky F. Twenty-five years of the trauma audit and research network: a continuing evolution to drive improvement. *Emerg Med J* 2015;32(12):906-8. doi: 10.1136/emermed-2015-205460
12. Roberts HC, Denison HJ, Martin HJ, et al. A review of the measurement of grip strength in clinical and epidemiological studies: towards a standardised approach. *Age Ageing* 2011;40(4):423-9. doi: 10.1093/ageing/afr051
13. Rockwood K, Song X, MacKnight C, et al. A global clinical measure of fitness and frailty in elderly people. *CMAJ* 2005;173(5):489-95. doi: 10.1503/cmaj.050051
14. Sherbourne CD, Stewart AL. The MOS social support survey. *Soc Sci Med* 1991;32(6):705-14.
15. Schwarzer R, Bäßler J, Kwiatek P, et al. The Assessment of Optimistic Self-beliefs: Comparison of the German, Spanish, and Chinese Versions of the General Self-efficacy Scale. *Applied Psychology* 1997;46(1):69-88. doi: 10.1111/j.1464-0597.1997.tb01096.x
16. Hounsome N, Orrell M, Edwards RT. EQ-5D as a Quality of Life Measure in People with Dementia and Their Carers: Evidence and Key Issues. *Value in Health* 2011;14(2):390-99. doi: http://dx.doi.org/10.1016/j.jval.2010.08.002
17. Salen BA, Spangfort EV, Nygren AL, et al. The Disability Rating Index: an instrument for the assessment of disability in clinical settings. *J Clin Epidemiol* 1994;47(12):1423-35.
18. National Institute for Health and Care Excellence. Guide to the methods of technology appraisal. In: Excellence NIfHaC, ed.: National Institute for Health and Care Excellence, 2013.
19. Department of Health and Social Care. NHS Reference Costs 2015 to 2016: Department of Health and Social Care, 2016.
20. National Health Service. NHS Supply Chain Catalogue [Available from: https://my.supplychain.nhs.uk/catalogue accessed Mar 2018.
21. Curtis L, Burns A, Personal Social Services Research Unit. Unit Costs of Health and Social Care. *Personal Social Services Research Unit* 2016
22. van Hout B, Janssen MF, Feng YS, et al. Interim scoring for the EQ-5D-5L: mapping the EQ-5D-5L to EQ-5D-3L value sets. *Value Health* 2012;15(5):708-15. doi: 10.1016/j.jval.2012.02.008
23. Moore GF, Audrey S, Barker M, et al. Process evaluation of complex interventions: Medical Research Council guidance. *BMJ* 2015;350:h1258. doi: 10.1136/bmj.h1258
24. Herdman M, Gudex C, Lloyd A, et al. Development and preliminary testing of the new five-level version of EQ-5D (EQ-5D-5L). *Qual Life Res* 2011;20(10):1727-36. doi: 10.1007/s11136-011-9903-x
25. Costa ML, Achten J, Hennings S, et al. Intramedullary nail fixation versus locking plate fixation for adults with a fracture of the distal tibia: the UK FixDT RCT. *Health Technol Assess* 2018;22(25):1-148. doi: 10.3310/hta22250
26. Freedman B. Equipoise and the ethics of clinical research. *N Engl J Med* 1987;317(3):141-5. doi: 10.1056/NEJM198707163170304
27. Lidz CW, Appelbaum PS. The therapeutic misconception: problems and solutions. *Med Care* 2002;40(9 Suppl):V55-63. doi: 10.1097/01.MLR.0000023956.25813.18

For peer review only - http://bmjopen.bmj.com/site/about/guidelines.xhtml

28. Donovan JL, Paramasivan S, de Salis I, et al. Clear obstacles and hidden challenges: understanding recruiter perspectives in six pragmatic randomised controlled trials. *Trials* 2014;15(1):5. doi: 10.1186/1745-6215-15-5
29. Donovan JL, de Salis I, Toerien M, et al. The intellectual challenges and emotional consequences of equipoise contributed to the fragility of recruitment in six randomized controlled trials. *Journal of Clinical Epidemiology* 2014;67(8):912-20. doi: https://doi.org/10.1016/j.jclinepi.2014.03.010
30. Realpe A, Adams A, Wall P, et al. A new simple six-step model to promote recruitment to RCTs was developed and successfully implemented. *Journal of Clinical Epidemiology* 2016;76:166-74. doi: 10.1016/j.jclinepi.2016.02.002
31. Donovan JL, Rooshenas L, Jepson M, et al. Optimising recruitment and informed consent in randomised controlled trials: the development and implementation of the Quintet Recruitment Intervention (QRI). *Trials* 2016;17:283. doi: 10.1186/s13063-016-1391-4
32. Townsend D, Mills N, Savović J, et al. A systematic review of training programmes for recruiters to randomised controlled trials. *Trials* 2015;16(1):432. doi: 10.1186/s13063-015-0908-6

For peer review only

FIGURE LEGENDS

Figure 1: Summary of recruitment and data collection. [$]The eligible age was changed from 50 to 18 during the study.

For peer review only - http://bmjopen.bmj.com/site/about/guidelines.xhtml

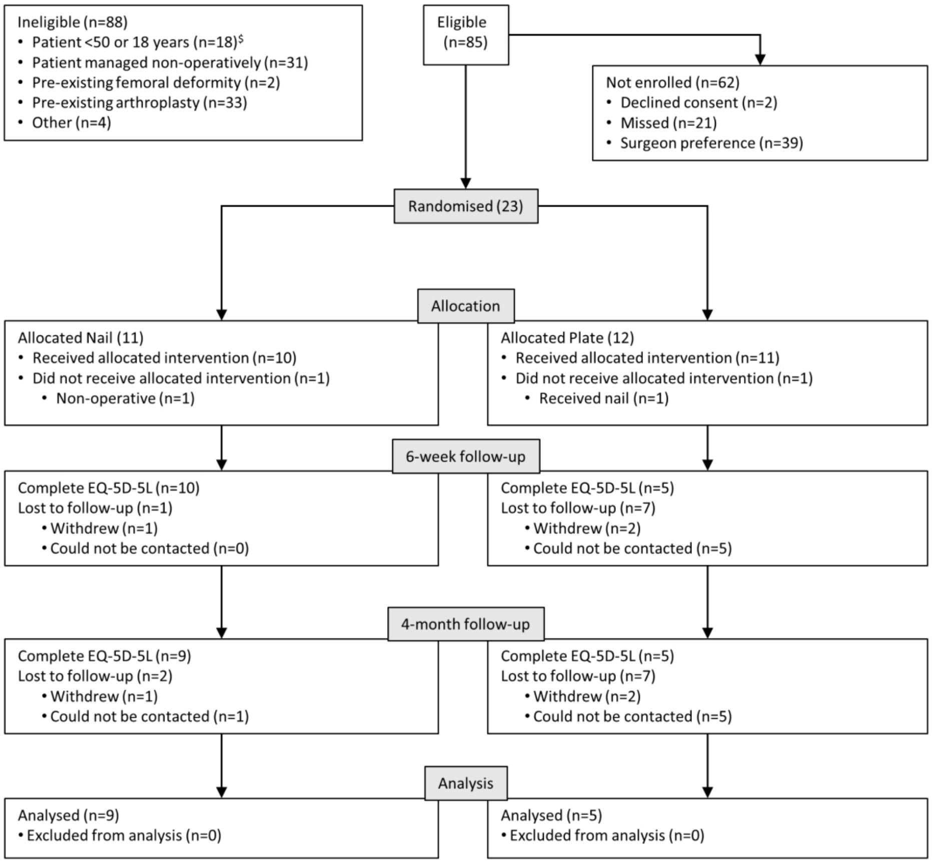

Figure 1: Summary of recruitment and data collection. $The eligible age was changed from 50 to 18 during the study.

190x188mm (300 x 300 DPI)

Trial of Acute Femoral Fracture Fixation (TrAFFix), a feasibility study

For patients with an acute, fragility fracture of the distal femur, is there a clinical and cost-effectiveness difference between locking plate fixation and retrograde intramedullary nail fixation?

Protocol version 3.0
05 May 2017

Ethical approval

NRES approval was obtained on the 01/08/2016 with reference number 16/WA/0225

Funding

This study is funded by the National Institute of Health Research Health Technology Assessment (15/59/22)

Sponsorship

The University of Oxford is the sponsor of this study.

Registration

The study has been registered with the ISRCTN registry under reference number ISRCTN92089567, and on the NIHR Portfolio (CPMS ID: 32536).

Dates

Study start date: 01/06/2016
Study end date: 31/01/2018

For peer review only - http://bmjopen.bmj.com/site/about/guidelines.xhtml

Table of contents

TABLE OF CONTENTS **2**
ABBREVIATIONS **3**
1. CONTACT DETAILS **4**
2. SYNOPSIS **5**
3. RATIONALE **6**
3.1 Background 6
3.2 Good Clinical Practice 7
3.3 Consort 7
4. TRIAL DESIGN **8**
4.1 Trial summary 8
4.2 Objectives 8
4.3 Measurements 9
4.4 Sample size 12
4.5 Methodology 12
4.5.1 Eligibility *12*
4.5.3 Recruitment and consent – process evaluation *15*
4.5.4 Randomisation *15*
4.5.5 Post randomisation withdrawals/exclusions *16*
4.5.6 Blinding *16*
4.6 Technologies assessed 16
4.6.1 Treatment options *16*
4.6.2 Rehabilitation *17*
4.6.3 Follow-up *17*
4.7 Process evaluation 17
4.7.1 Implementation *18*
4.7.2 Mechanisms of impact *18*
4.7.3 Context *18*
4.8 Adverse event management 19
4.8.2 Risks and benefits *19*
4.9 End of trial 19
5. DATA MANAGEMENT **21**
5.1 Statistical Analysis 21
5.2 Economic evaluation 22
5.3 Process evaluation 22
6. TRIAL OVERSIGHT **23**
6.1 Trial Supervision 23
6.2 Quality control 23
6.3 Funding 24
6.4 Insurance and Indemnity Arrangements 24
6.5 Dissemination 24
6.6 Project Timetable and Milestones 25
7. PROTOCOL AMENDMENTS: **26**
8. REFERENCES **27**

For peer review only

For peer review only - http://bmjopen.bmj.com/site/about/guidelines.xhtml

Abbreviations

AE – Adverse Event
CI – Chief Investigator
CRF – Clinical Reporting Form
DEMQoL – Dementia Quality of Life
DSMC – Data Safety Monitoring Committee
DRI – Disability Rating Index
EQ-5D – EuroQol
GCP – Good Clinical Practice
HE – Health Economy/Economist
HTA- Health Technology Assessment
MCA – Mental Capacity Act
MOS – Medical Outcomes Survey
MRC – Medical Research Council
OCTRU – Oxford Clinical Trials Research Unit
PACS - Picture Archiving and Communications System
PI – Principal Investigator
QA – Quality Assurance
QALY – Quality Adjusted Life Year
RCT- Randomised Controlled Trial
REC – Research Ethics Committee
RF – Research Fellow
SAE – Serious Adverse Event
SOP – Standard Operating Procedure
TMG – Trial Management Group
TrAFFix – Trial of Acute Femoral Fracture Fixation
TSC – Trial Steering Committee
VAS – Visual Analogue Scale

For peer review only

For peer review only - http://bmjopen.bmj.com/site/about/guidelines.xhtml

1. Contact details

Chief Investigator
Xavier Griffin
Oxford Trauma, Kadoorie Centre
NDORMS, University of Oxford
John Radcliffe Hospital, Headley Way
Oxford, OX3 9DU
xavier.griffin@ndorms.ox.ac.uk

Research Manager
Dr Juul Achten
Oxford Trauma, Kadoorie Centre
NDORMS, University of Oxford
John Radcliffe Hospital, Headley Way
Oxford, OX3 9DU
juul.achten@ndorms.ox.ac.uk

Trial Co-ordinator
Dr Robin Lerner
Oxford Trauma, Kadoorie Centre
NDORMS, University of Oxford
John Radcliffe Hospital, Headley Way
Oxford, OX3 9DU
robin.lerner@ndorms.ox.ac.uk

Trial Management Group
Mr Xavier Griffin
Prof Matt Costa
Dr Juul Achten
Dr Nick Parsons
Dr Robin Lerner
Mr Damian Haywood
Dr Melina Dritsaki
Dr Janis Baird

Trial Steering Committee

Mr Mike Kelly	Chair
Mr Graham Smith	Independent clinician
Dr Ada Keding	Independent statistician
Dr Philip Bell	Independent lay member
Mr Xavier Griffin	Chief Investigator

Data monitoring committee

Mr Dan Perry	Chair
Mr Tim White	Independent clinician
Dr Nikki Totton	Independent statistician

For peer review only - http://bmjopen.bmj.com/site/about/guidelines.xhtml
For peer review only

2. Synopsis

Study Title	Trial of Acute Femoral Fracture Fixation.<br>For patients with an acute fragility fracture of the distal femur, is there a clinical and cost-effectiveness difference between locking plate fixation and retrograde intramedullary nail fixation?	
Internal ref. no. / short title	TrAFFix	
Study Design	Feasibility trial for a multi-centre, multi-surgeon, parallel, two-arm, randomised controlled trial	
Study Participants	Participants of 18 years and older who have sustained an fracture of the distal femur and who would benefit from internal fixation of the fracture	
Planned Sample Size	52	
Planned Study Period	01/6/16 - 31/01/18	
	Objectives	Outcome Measures
Primary	• Assess the feasibility of a future definitive trial	• Recruitment rate<br>• Completion rate of EQ-5D-5L at 4 months
Secondary	• Perform a process evaluation to understand the generalisability and likely success of a future trial<br><br>• Explore the validity of self and proxy reporting of the EQ-5D-5L in this population.	• Compliance with peri-operative and post-operative components of protocol<br>• Intervention acceptability<br>• Patient and staff experiences<br><br>• Dementia quality of life measure (DEMQoL)<br>• EQ-5D-5L (proxy and self report)

For peer review only

For peer review only - http://bmjopen.bmj.com/site/about/guidelines.xhtml

3. Rationale

3.1 Background

Fractures of the distal femur are increasingly common injuries. They account for 5% of all fractures of the femur with an estimated incidence of 10 per 100,000.[1] The optimal management of these fractures remains controversial. There is a bimodal distribution of the incidence of these fractures with age.[2] The majority, approximately 85%, are fragility fractures sustained by elderly patients after a fall from a standing height, the remainder are typically sustained by multiply injured patients after massive trauma.[2] This study will investigate the commonest fracture subtype – fragility fractures sustained in the elderly population often as a consequence of osteoporosis.

Fragility fractures of the distal femur occur in the same population as hip fractures.[3] Similar to hip fractures, these are significant injuries in a vulnerable group of patients; causing considerable morbidity and mortality as well as placing a major financial burden on the NHS.[4] Hip fracture is approximately ten times more common than fracture of the distal femur.[1] However, the overall incidence of fragility fractures associated with osteoporosis is rising steadily, promising an increasing challenge to future healthcare provision.[5]

There has been very little research exploring treatment options for distal femoral fractures in this population. A recent Cochrane review[6] found few trials in this area, most of which compared outdated implants, such as non-anatomic, non-locking plating systems or earlier generation nails. Furthermore, important limitations in the methodology of each of the trials were identified leading to substantial risks of bias. It was suggested that in order to optimise patient functional recovery following this debilitating injury “a well-designed, adequately powered, randomised controlled trial comparing modern treatments is required”.[7]

It is current practice to manage displaced fractures with operative fixation if the patient is medically fit enough to undergo surgery.[3 8] Surgery reduces the substantial complications associated with non-operative treatment, such as prolonged immobilisation and bed rest, as well as the problems of non-union and mal-union.[9]

Since the operative treatment of these fractures was popularised, there has been a wide variety of implants employed to achieve fixation. Despite the significant advancements in implant design and manufacture, operative fixation is still associated with substantial complications similar to those seen with hip fractures.[8 10] This reflects the common pathology underlying these injuries, that the bone is weakened by osteoporosis, undermining the stability of the bone-implant construct.

The two interventions most commonly used in UK practice are intramedullary fixation with a locked retrograde nail (nail), and extramedullary fixation with an anatomical angular stable plate (locking plate).[3] Nails offer twin theoretical advantages; the mechanical impact of a long, intramedullary device that is close to the axis of the femur[11] and the biological advantages of minimum disruption of the fracture site and stimulation of blood supply through reaming.[12] However, nails provide only limited options for distal locking screws, as all screws must pass through the centre of the nail, so the stability of the bone-implant construct may be sub-optimal. Locking-plate fixation has been facilitated by recent advances in implant technology that allow the screws to be screwed into the bone as well as the plate itself (‘locked’). This produces a 'fixed-angle' bone-plate

For peer review only - http://bmjopen.bmj.com/site/about/guidelines.xhtml

construct. These plates were designed specifically for use in osteoporotic bone, and have been shown to exhibit excellent biomechanical properties.[13] However, they are more expensive than nails and require larger surgical wounds to apply. There are few clinical data available to guide clinicians,[7] and it is clear that there is no current consensus concerning the best management of these injuries.[3]

We performed a multicentre retrospective study to review the current management of distal femoral fractures at four UK major trauma centres.[3] We found that only two devices are now used for fixation – retrograde nails and locking plates. Also, over 80% of these injuries were fragility fractures in a population which matched those sustaining hip fracture. Furthermore, mortality in this frail elderly group of patients was 20% at one year and the prevalence of cognitive impairment 30%. The overall annual incidence in each centre was 20 fractures per year. This retrospective study illustrates the patient demographics, the variability in treatment of these fractures in the UK and the considerable morbidity associated with the injury.

Crucially, we have found that there may be an important difference in outcomes following the choice of surgical management of these patients. The mean benefit of a nail over locking plates may be as great as 0.12 in EQ-5D-derived utility (p=0.019).[14] The minimum clinically important difference for EQ-5D is estimated to be 0.08.[15] Similar effect sizes are demonstrated in other measures of function and quality-of-life, such as the Glasgow Outcome Scale (extended) and SF-12.[14 16 17] These findings have also been reported by other groups. A small pilot study in the US comparing these technologies found some evidence of a similar benefit in quality of life in favour of nails (mean difference EQ-5D 0.1, p=0.07).[18]

We propose, therefore, to conduct a feasibility study for a later definitive randomised controlled trial comparing functional outcome after treatment with modern intramedullary nails or anatomical locking plates for fragility fractures of the distal femur.

3.2 Good Clinical Practice

The trial will be conducted in accordance with the Medical Research Council's Good Clinical Practice (MRC GCP) principles and guidelines, the Declaration of Helsinki, Oxford Clinical Trials Unit SOPs, relevant UK legislation and this Protocol. GCP-trained personnel will conduct the trial.

3.3 Consort

The trial will be reported in line with the CONSORT statement.[19]

For peer review only - http://bmjopen.bmj.com/site/about/guidelines.xhtml

4. Trial design

4.1 Trial summary

The research plan is broken into two phases. Phase 1 is a feasibility study and will determine the expected rate of recruitment in a large scale multicentre randomised controlled trial. Phase 2 will be a definitive multi-centre randomised controlled trial in the UK. This protocol concerns Phase 1 only.

The feasibility study will take place in 6 centres over a period of 20 months. The aim will be to determine the number of eligible and recruited patients in the trauma centres over the course of 10 months. Screening logs will be kept at each site to determine the number of patients assessed for eligibility and the reasons for any exclusions. In addition, the number of eligible and recruited patients, and the number of patients who decline consent/withdraw, will be recorded. Follow-up will be limited to 4 months to determine early completion rates for the outcome measures.

All adult patients (≥18 years old) presenting at the trial centres with an isolated, acute, fracture of the distal femur are potentially eligible to take part in the trial. The study will assess feasibility in patients with fragility fractures and non-fragility fractures. Fragility fractures will be defined using a combination of the mechanism of injury (fall from standing) and age (fractures sustained by adults ≥50 years old). [20] We hope the broad eligibility criteria will ensure that the results of the study can readily be generalised to the wider patient population. A computer generated randomisation sequence, stratified by centre and chronic cognitive impairment will be produced and administered independently by a secure web-based service. Randomisation will be on a 1:1 basis to either nail or locking plate fixation. Both of these operations are widely used within the NHS[3] and all of the surgeons in the chosen centres will be familiar with both techniques.

Demographic data, pre-injury and contemporary functional data will be collected at baseline. Radiographs will be collected at baseline and 6 weeks post-surgery. Follow-up questionnaires will be completed at 6 weeks and 4 months post-surgery. The primary outcome (EQ-5D-5L[21]) will be determined at 4 months, when the majority of recovery has been attained by this population of patients.[3 22]

Patients without cognitive impairment will be asked to complete the EuroQol 5 Dimensions (EQ-5D-5L) health-related quality of life questionnaire[21] and the Disability Rating Index (DRI).[23] Patients with cognitive impairment, or an appropriate proxy, will complete the EQ-5D-5L[21] and Dementia Quality of Life (DEMQoL)[24] questionnaires.

A process evaluation will be performed as part of the feasibility study. The main aim of this evaluation will be to identify barriers and facilitators in the delivery of the interventions and the trial, through a mixed methods approach. This will include qualitative interviews with staff and participants as well as a quantitative assessment of the characteristics of the sample, the fidelity of the interventions and the acceptability of the follow-up schedule. Results from the process evaluation will inform the development of any future definitive trial.

4.2 Objectives

1. Assess the feasibility of a future definitive trial.
2. Perform a process evaluation to understand the generalisability and likely success of a future trial.
3. Explore the validity of self and proxy-reporting of the EQ-5D-5L in this specific population.

For peer review only - http://bmjopen.bmj.com/site/about/guidelines.xhtml

4.3 Measurements

The primary outcome measures for this study are the participant recruitment rate and the completion rate of the EQ-5D-5L at 4 months post-surgery. Measurements that will be made during the trial are summarised in Table 1 (overleaf).

Baseline characteristics: Routine baseline characteristics will be recorded for all participants to describe the nature of the participants. Additional measurements will be made to more fully describe the groups:

Grip strength: Grip strength is a measure of muscle strength and gives an indication of sarcopaenia, a predictor of frailty, and will be measured as previously described by Roberts et al.[25]

Frailty: The degree of frailty can provide useful predictive information[26], and will be measured using the Rockwood frailty scale score. The Rockwood scale score is a quick 9-point index used to measure frailty.[26]

Social support: The Medical Outcomes Survey (MOS) social support survey is a brief multi-dimensional, self-administered social support survey.[27]

Self-efficacy: Self-efficacy is a measure of an individual's confidence in their ability to accomplish tasks and overcome problems. Low levels of self-efficacy are associated with less optimal health behaviours.[28]

EuroQol 5 Dimensions (5L) Score (EQ-5D-5L)[21]: EQ-5D-5L is a validated, generalised and standardised instrument comprising a visual analogue scale (VAS) measuring self-rated health and a health status instrument, consisting of a five-level response (no problems, some problems, moderate problems, severe problems and unable) for five domains related to daily activities;[21] (i) mobility, (ii) self-care, (iii) usual activities, (iv) pain and discomfort and (v) anxiety and depression. Responses to the health status classification system are converted into an overall score using a published utility algorithm for the UK population. [29] A respondent's EQ-VAS gives self-rated health on a scale where the endpoints are labelled 'best imaginable health state' (100) and 'worst imaginable health state' (0). EQ-5D-5L has some important advantages in this study. It has been validated for use in patients with cognitive impairment where an appropriate proxy may respond to the questions.[30] It can be administered by mail or by telephone. Our recent work has demonstrated it to have excellent measurement properties in comparison with other commonly used disease and region-specific outcome tools in the similar cohort of patients with fragility hip fracture.[20 31] EQ-5D-5L scores will be collected at baseline (for pre and post-surgery), 6-weeks post-surgery, and 4-months post-surgery.

The secondary outcome measures in this study are:

Disability Rating Index (DRI):[23] The DRI score is a validated self-reported questionnaire. It consists of 12 items specifically related to function of the lower limb. These data will be collected at baseline, 6 weeks, and 4 months post-surgery in participants who do not have cognitive impairment. The DRI[23] has been proven to be a robust, practical clinical and research instrument with good responsiveness and acceptability for assessment of disability caused by impairment in the lower limb.[32]

Dementia Quality of Life Measure (DEMQoL):[24] The DEMQoL[24] score is a validated questionnaire specifically designed to assess quality of life in patients with dementia. A large minority of the participants in this study are expected to have co-existing dementia. The score can be self or proxy-reported and consists of 28 or 31 items respectively. These data will be collected at baseline, 6 weeks, and 4 months post-surgery in participants who have cognitive impairment. Recently preference based utility scores for a UK population have also been published.[33]

For peer review only - http://bmjopen.bmj.com/site/about/guidelines.xhtml

Complications: All complications will be recorded. Complications will be classified as either:

- unrelated to the trial protocol,
- related systemic complications (including venous thromboembolic phenomena, death, pneumonia, urinary tract infection, blood transfusion, acute cerebrovascular incident, acute cardiac event, other),
- related local complications (superficial/deep infection, non/mal union, failure/removal/revision of metalwork, injury to adjacent structures such as nerves/tendons/blood vessels, other).

Radiographic evaluation: Standard anterior-posterior and lateral radiographs of the femur will be assessed for mal-union at 6 weeks post-injury. Radiographs are those routinely used for the investigation of patients with a suspected fracture of the distal femur and for the follow-up of such patients following any intervention, so there will be no need to request any additional or special investigations. Radiographs will be assessed by an independent researcher at each site for:

- Evidence of early loss of fixation
- Varus/valgus more than 5°
- Re/procurvatum more than 10°
- Shortening more than 1cm

Semi-structured interviews: Patients, carers and staff will be asked to participate in qualitative interviews to discuss their experience of participating in the trial and the intervention. Interviews will be semi-structured, based on the semi-structured interview guide.

For peer review only - http://bmjopen.bmj.com/site/about/guidelines.xhtml

	Pre-surgery	Baseline	6 Weeks	4 Months
**Pre-Randomisation**				
Fracture classification[34]	X			
Cognitive impairment	X	X		
Age	X			
**Personal Details**				
Contact details		X		
**Baseline characteristics**				
Date of birth		X		
Sex		X		
Current medications		X		
Co-morbidities		X		
Current/previous occupation		X		
Educational attainment		X		
Grip strength		X	X	X
Self-efficacy report		X		
Rockwood frailty score		X		
MOS social support		X		
Government benefits		X		
Residential status		X		
Discharge destination		X		
Mobility		X		
**Treatment**				
Additional fixation		X		
Anaesthesia		X		
Grade of surgeon		X		
Prescribed medications		X		
Rehab Assessment		X		
**Outcomes**				
EQ-5D-5L		Pre+post injury	X	X
DEMQoL[A]		Pre+post injury	X	X
DRI[B]		Pre+post injury	X	X
Radiographs		X	X	
Complications		X	X	X
Health Economics				X
Qualitative interviews[C]		X	X	X

Table 1 Follow-up measures. [A]Patients with chronic cognitive impairment, [B]Patients without chronic cognitive impairment, [C]Selection of patients & staff

For peer review only

For peer review only - http://bmjopen.bmj.com/site/about/guidelines.xhtml

4.4 Sample size

Data from the feasibility study will be used calculate estimates of the standard deviation of the primary outcome measure (EQ-5D-5L) to drive a formal power analysis and sample size calculation for the definitive trial; evidence from other relevant sources to EQ-5D-5L [20 31] will also be used to inform this process. We anticipate that each of 6 participating centres will treat approximately 1.5 eligible patients per month.[3] Taking a conservative approach, we would expect, to ensure feasibility, to be able to recruit 1.0 patients per month per centre. Given our schedule for centre opening times and recruitment length, we will have 52 centre months available to recruit for this study. Assuming that the recruitment rate is 1.0 per month per centre and monthly centre counts of patient recruitment numbers are approximately Poisson distributed and independent of one another, then this will allow us to estimate the recruitment rate with a 95% confidence interval of 0.73 - 1.28.[35] Therefore recruiting 52 patients in total should provide sufficiently precise estimates of the monthly recruitment rate to decide if a definitive trial is feasible.

For the qualitative interviews, convenience sampling will be used to identify potential participants at different time points. The sampling of patients and staff will be reviewed on an ongoing basis throughout the process evaluation, and interviews will continue until data saturation is achieved.

4.5 Methodology

4.5.1 Eligibility

Patients will be screened against the following criteria:

<u>Inclusion criteria</u>

Patients will be eligible for this study if they:

- are ≥18 years old,
- have a fracture of the femur involving the distal two "Muller" squares,[34]
- would, in the opinion of the attending surgeon, benefit from internal fixation of the fracture.

<u>Exclusion criteria</u>

Patients will be excluded from this trial if they have:

- a loose knee or hip arthroplasty requiring revision,
- pre-existing femoral deformity,
- an arthroplasty that precludes nail fixation.

Patients who sustain injuries to areas of the body other than the lower limbs, which may affect the primary outcome measure, will still be included in the analysis. For patients with bilateral fractures of the distal femur, a rare event, both fractures will be recorded but only one fracture will be included in the trial. The treating surgeon will decide which injury to include in the study prior to randomisation.

All patients who are eligible for inclusion in TrAFFix and their personal consultees, as well as all staff members involved in the research and intervention delivery, are eligible to be approached about participating in qualitative interviews as part of the process evaluation.

Many of the patients who sustain this injury will have some degree of cognitive impairment, similar to patients sustaining hip fracture. Some will have chronic cognitive impairment[3] and some will likely be suffering with acute delirium.[36] Cognitive status is an important, independent predictor of patients' baseline functional, quality-of-life status and likely outcome in this population.[22 36] This is

For peer review only - http://bmjopen.bmj.com/site/about/guidelines.xhtml

possibly related to patients' pre-injury independence, post-injury expectations and ability to participate fully in their rehabilitation. Inclusion of these patients is essential to determine specific effect sizes in this subgroup and so that the sample properly reflects the population sustaining this injury. Recent studies investigating hip fracture have demonstrated that contemporary trials including patients with cognitive impairment have good acceptability and are feasible.[37-39]

4.5.2 Recruitment and consenting – for randomised control trial

The clinical care team will notify the research team of any potentially eligible patients. Non-identifiable patient details will then be used for screening in the Emergency Department and Trauma Wards at the trial centres. Reasons for patients' exclusion or ineligibility will be recorded.

The nature of these injuries means that the great majority of patients will be operated on immediately or on the next available trauma operating list, depending on access to an appropriate operating theatre. Some patients may be unconscious, all will be distracted by the injury to their lower limb and its subsequent treatment and all will have had large doses of opiates for pain relief, potentially affecting their ability to process information. Similarly, patients' next-of-kin, carers and friends are often anxious at this time and may have difficulty in weighing the large amounts of information that they are given about the injury and plan for treatment. In this emergency situation the focus is on obtaining consent for surgery (where possible) and informing the patient and any next-of-kin about immediate clinical care. The consent procedure for this trial will reflect that of the surgery, with the clinical team assessing capacity before taking consent for the surgical procedure and this capacity assessment then being used to decide on the proper approach to consenting to the research. The appropriate method, as described below, will then be used to gain either prospective or retrospective consent from the patient or appropriate consultee by a GCP-trained, appropriately delegated member of the research team.

Conducting research in this 'emergency setting' is regulated by the *Mental Capacity Act 2005* (MCA). As patients may lack capacity as described above, and because of the urgent nature of the treatment limits access to and appropriate discussion with personal consultees, we propose to act in accordance with section 32, subsection 9b of the MCA following a process approved by the relevant research ethics committee; the clinical team will make an assessment of capacity as per usual procedures for obtaining consent for a surgical procedure. The clinical team will then provide guidance to the research team as to whether the patient has capacity to consent prospectively or if consultee agreement should be sought.

Where the clinical team advise that prospective patient consent is appropriate, this will be sought by the research team. If the clinical team advise that prospective patient consent is not appropriate, the research team will approach an appropriate Consultee. Where a Personal Consultee is available, they will be provided with the study information. The Personal Consultee will be given the opportunity to ask questions and discuss the study after which their written agreement will be recorded. Where a Personal Consultee is not available then a Nominated Consultee will be identified to advise the research team. The Nominated consultee will be the patient's treating surgeon. If that surgeon is a member of the research team, another independent surgeon will be identified. The Nominated Consultee will be asked to agree for the patient to be randomised, this will be prospectively recorded during the electronic randomisation process. Hereafter, at the first appropriate opportunity and when the clinical situation allows, the Nominated Consultee will provide a wet-ink signature on a copy of the electronic recorded agreement. Consent or agreement for further participation into the study after surgery will then be sought by the patient themselves or a Personal/Nominated consultee.

Those patients who are able to consent before their operation will always be approached for consent before surgery. For those patients that did not consent prior to surgery, the research associate will

For peer review only - http://bmjopen.bmj.com/site/about/guidelines.xhtml

provide the patients with all of the study information at the first appropriate time when the patient has regained capacity. The patients will be given the opportunity to ask questions and discuss the study with their family and friends. They will then be asked to provide written consent for continuation in the study. Patients will be asked to consent to long-term follow-up and data linkage to routine NHS datasets.

For those patients who did not prospectively consent or who had a nominated consultee give prospective agreement and still lack capacity after their surgery, a personal Consultee will be contacted to advise the research team about the patients continued participation in the study. The Personal Consultee will be provided with all the study information and be given the opportunity to ask questions and discuss the study with other relatives and friends. If the consultee is present during the consultee agreement discussion, they will be asked to sign a consultee agreement form. In those circumstances where the consultee is not present (for example when they are being contacted via telephone), verbal agreement will be recorded by the research associate on an informed agreement checklist. For all patients, a consultee decision with regards agreement for the patient to continue to be involved in the study, will be recorded in the patient's notes.

On rare occasions, participants may be discharged prior to consent or consultee agreement. If this happens the trial team will make every effort to discuss the trial with the patient at their next clinical follow-up appointment. If the patient lacks capacity at this appointment, the trial will be discussed with the patient's personal consultee.

Patients or Personal Consultees who prefer not to be actively involved in the study follow-up, will be asked if they are willing to consent to the research team using their routinely collected NHS data for the study. All original signed consent forms will be kept in the investigator site file. Three copies of the consent forms will be made; one held in the patient's medical notes, one for the patient and one copy for the study team.

Throughout the study, best efforts will be made to involve participants who, temporarily or permanently, lack capacity in the decision to be involved in the study. The clinical team will make a judgement about the amount and complexity of the information that the participant is able to understand and retain on an individual basis. Appropriate information will be communicated to the participant and updated as their understanding changes. At all times the study team will act in accordance with the participants' best interests. Any new information that arises during the trial that may affect participants' willingness to take part will be reviewed by the Trial Steering Committee; if necessary this will be communicated to all participants. A revised consent form will be completed if necessary.

Responsibility for recording and dating both oral and written informed consent or agreement will be with the investigator, or persons delegated by the investigator, who conducted the informed consent discussion. Delegated responsibility should be recorded on the site delegation log. Permission will be sought to inform the patients GP of their participation in the study.

For peer review only - http://bmjopen.bmj.com/site/about/guidelines.xhtml

4.5.3 Recruitment and consent – process evaluation

As part of the initial consent process patients and their personal consultees will be asked whether they may be approached about participating in interviews with regards their views on participating in this trial. Written agreement from patients or their personal consultees to be approached for an interview will be sent to the TrAFFix research office. Patients who do not consent to the main trial may agree to be approached by a member of the research team about taking part in an interview. A researcher from the TrAFFix team will identify which patients will be approached for interview, from those who agree to be approached. A researcher from the TrAFFix team will also identify which NHS staff could be approached for interview.

Participants who agree to be approached will have a separate informed consent discussion for the interviews with a member of the research team either in person, by phone, or by post. NHS staff will initially be invited to interview by phone or email, and if they agree to be approached an informed consent discussion will take place with a researcher from the TrAFFix office by phone, post or in person.

As part of the process evaluation, conversations between researchers and patients or personal consultees may be audio recorded. This may include the initial discussion about TrAFFix, before informed consent for the main study has been give. Where audio recording may be acceptable and appropriate, local research staff will explain the rationale for the process evaluation and recording of the conversation and ask patients whether the conversation can be audio recorded. Where patients agree, their verbal consent will be recorded at the start of the recording. Where patients do not agree, the informed consent conversation will continue as usual without being recorded.

4.5.4 Randomisation

Eligible participants will usually be enrolled by a trial research associate or member of the clinical team using a secure online registration and randomisation system provided by the Oxford Clinical Trials Research Unit (OCTRU). Basic information including the patient initials, age and eligibility checks will be entered. The patient will then receive a trial ID that will be used on all non-public facing trial documentation.

The treatment allocation will be generated prior to surgery, allowing randomisation outside of working hours. The allocation sequence will be stratified by recruitment centre and cognitive status.

Stratification by centre will ensure that any clustering effect related to the centre itself will be equally distributed across the trial arms. This will not eliminate centre or surgeon –specific effects, but as between 10 and 30 surgeons will be involved in the management of the patient group, it is likely that any one surgeon will treat only 2-3 patients enrolled in the trial. Patients will also be stratified by chronic cognitive impairment, which will be judged by the clinical team upon randomisation, to ensure that participants with cognitive impairment will be allocated evenly across the treatment groups.

For peer review only - http://bmjopen.bmj.com/site/about/guidelines.xhtml

4.5.5 Post randomisation withdrawals/exclusions

Participants may decline to continue to take part in the trial at any time without prejudice. A decision to decline consent or withdraw will not affect the standard of care the patient receives. Once withdrawn, the patient will be advised to discuss their further care plan with their surgeon. Data collected up to the point of withdrawal will be included in the final analysis. Post-randomisation withdrawals will not be replaced.

4.5.6 Blinding

As the surgical scars are clearly visible, the patients cannot be formally blinded to their treatment. Participants will only be informed of their treatment allocation at the end of the trial. In addition, the treating surgeons will also not be blind to the treatment, but will take no part in the post-operative assessment of the patients. The functional outcome data will be collected and entered onto the trial central database by a research assistant/data clerk in the trial central office. The radiographs collected will be reviewed by independent assessors.

4.6 Technologies assessed

Participants will usually be assessed in the Emergency Department. Diagnosis of a fracture of the distal femur will be confirmed from plain radiographs of the femur. Where there is doubt over the radiological pattern of the fracture, for example whether it extends into the knee or not, participants will be reviewed by the on-call orthopaedic surgeon and where clinically indicated a CT may be performed – this constitutes standard of care practice.

All participants will undergo the following investigations as a minimum: electrocardiogram, full blood count, group and save, coagulation screen, urea, creatinine and electrolytes. Routine thromboprophylaxis will be started in all participants not already receiving anticoagulant therapy. Pharmaceutical and mechanical prophylaxis measures will be used in accordance with current practice agreed at each centre. A regional or general anaesthesia technique will be used and routine analgesia provided according to local practice.

All participants will receive perioperative prophylactic antibiotics in accordance with current practice agreed at each centre. Appropriate preparation, positioning and fracture reduction will be left to the discretion of the operating surgeon, as per their normal clinical practice. Participants will be randomly allocated to fixation using either intramedullary nailing or locking plate fixation.

4.6.1 Treatment options

All the hospitals involved in this trial currently use both of the methods of fixation[3] and all of the consulting surgeons involved will be familiar with both techniques.

Intramedullary nailing: Fixation of the fracture will be achieved with a proximally and distally locked nail that spans the entire diaphysis of the femur. All nails will be introduced retrograde through the knee joint. In this pragmatic trial, the details of surgical incision and approach, fracture reduction and supplementary fixation with wires or screws will be at the surgeon's discretion as per their normal clinical practice.

Locking plate fixation: Fixation of the fracture will be achieved with anatomical distal femoral locking-plate and screws. Locking plates will be defined as those in which at least one fixed angle locking screw is placed distal to the fracture. The operating surgeon will determine the length, number and type of additional screws. Additional fixation with lag screws and cerclage wires will be

For peer review only - http://bmjopen.bmj.com/site/about/guidelines.xhtml

at the surgeon's discretion. In this pragmatic trial, the details of surgical incision and approach, fracture reduction, number and type of other screws and supplementary fixation with wires or screws will be at the surgeon's discretion as per their normal clinical practice.

4.6.2 Rehabilitation

Patients allocated to either of the two groups will receive the same standardised, written physiotherapy advice detailing the exercises they need to perform for rehabilitation following their injury. All of the patients in both groups will be advised to move their toes, ankle and knee joints fully within the limits of their comfort. Weight bearing status will be decided by the treating surgeon, with a preference for early weight bearing mobilisation immediately or as soon as the surgeon feels appropriate. In this pragmatic trial, any other rehabilitation input beyond the written physiotherapy advice (including a formal referral to physiotherapy) will be left to the discretion of the treating clinicians. However, a record of any additional rehabilitation input (type of input and number of additional appointments) together with a record of any other investigations/interventions will be requested as part of the 6 week and 4 month postal follow-ups and this will also form part of the trial dataset.

4.6.3 Follow-up

Baseline, 6-week and 4-month clinical report forms (CRFs) will be sent to the trial coordinating centre. The Research Associate will perform a clinical assessment and make a record of any early complications at 6 weeks. Standardised radiographs from 6-weeks post injury will be reviewed by a researcher at each site.

The majority of pre-injury function is recovered in this cohort of patients by 4 months.[20 31] The outcome measures will therefore be collected at this time point. The functional and health-related quality of life outcome data will be collected using the EQ-5D-5L[21], DEMQoL[24] and DRI[23] at baseline (post injury and retrospectively pre-injury), and 6 weeks and 4 months post-surgery.

Where possible patients will complete baseline, 6 week, and 4 month questionnaires while in hospital during their initial stay or during routine follow-up appointments, with the help of an appropriate proxy where necessary. If patients do not complete a questionnaire during a follow up visit, a copy will be sent to their address. If patients have not returned questionnaires, a copy of the outstanding questionnaires will be sent again by post and a follow-up phone call will be made by the trial central office. (The primary outcome questionnaire can be completed over the phone, even if postal copies are not returned). In the unlikely event that the follow-up data can still not be secured, then the patient's GP will be contacted to ensure correct contact details are available for the patient.

As part of routine clinical practice patients will be seen in clinic on a regular basis after this injury. Any further clinical follow-up in the first year after the injury will be at the discretion of the surgeon but will not influence the collection of the standard outcome data.

We will use techniques common in long-term cohort studies to ensure minimum loss to follow-up, such as collection of multiple contact addresses and telephone numbers, mobile telephone numbers and email addresses.

4.7 Process evaluation

We will evaluate intervention implementation, context and mechanisms of impact. This approach is consistent with recent Medical Research Guidance on process evaluation of complex interventions.[40] Process evaluation will enable us to understand how the interventions work and under what circumstances effectiveness is achieved. In this feasibility study we will be developing and testing

For peer review only - http://bmjopen.bmj.com/site/about/guidelines.xhtml

methods for process evaluation which might then be modified in the subsequent definitive trial. The findings of the process evaluation might also lead to modifications in the plan for outcomes evaluation and so we will not separate research team members conducting process and outcomes evaluation in the way that might be appropriate in the definitive trial. The process evaluation will be led by a post-doctoral fellow with mixed methods research experience, who will work closely with research nurses and surgeons in the participating centres.

4.7.1 Implementation

We will assess the implementation of study procedures in each of the six participating centres. Specifically, we will assess the fidelity of procedures for screening patients and applying study eligibility criteria, rates of screening and recruitment in each centre over the ten-month recruitment period, application of operative procedures for the two interventions and any deviations from the study protocol, fidelity of delivery and patient compliance with the peri-operative (thromboprophylaxis, analgesia and antibiotics) and post-operative components (rehabilitation and standardised physiotherapy advice) of the study. These data will be collected through standardised recording of the screening and eligibility assessments using an enhanced screening log, in which patients' reasons for refusal to participate can also be recorded, audio recording of a sample of the participant-researcher interactions, interviews with research nurses, surgeons, and physiotherapists and occupational therapists in each participating centre, and transcription of data from patient notes describing peri-operative, operative and post-operative care. Acceptability of the interventions will be assessed through interviews with participants during the post-operative period and will be carried out by the post-doctoral fellow or a member of the evaluation team with appropriate qualitative experience. Those patients who decline to take part in the full study to be invited for an interview to explore the reasons for declining to participate, in order to inform the design of any subsequent definitive trial.

4.7.2 *Mechanisms of impact*

We will work with stakeholders to develop a logic model for the feasibility study. Based on our previous research, the logic model will work from a hypothesis that nail fixation is associated with better outcomes than locking plate fixation. Together with stakeholders, we will identify relevant intermediate outcomes that might be associated with the effect of the interventions on the primary outcomes of interest. Such intermediate outcomes will relate to patient and surgical factors. Likely patient factors include the health of participants, as indicated by the presence of co-morbidities, their physical frailty and psychosocial factors including levels of self-efficacy and social support. Patients will also be interviewed to find out their experiences of treatment. Surgical factors include the skills and treatment preferences of the surgeons participating in the study. These will be assessed through assessment of surgical case-load and expertise collected from operative logs, and questionnaires and interviews completed by staff. .The content of interviews and questionnaires will be developed and reviewed on an ongoing basis during the trial.

4.7.3 Context

Contextual factors have the potential to influence the effectiveness of interventions and can lead to differential effects. The multi-centre nature of the feasibility study, and proposed definitive trial, means that factors within the centres need close consideration. Within the feasibility study we will work with stakeholders to identify important contextual factors and will develop methods for their assessment in the feasibility study in order that they can be refined for the definitive trial. Evaluation of context will focus on three areas: the demographic features of populations served by participating centres; care pathways and configuration of services within each centre; and centre specific issues, related to staffing for example. The approaches used to assess these components will include

For peer review only - http://bmjopen.bmj.com/site/about/guidelines.xhtml

interviews with research nurses, surgeons, physiotherapists and occupational therapists, documentary analysis and observation within the centres.

4.8 Adverse event management

Adverse events (AE) are defined as any untoward medical occurrence in a clinical trial subject and which do not necessarily have a causal relationship with the treatment. All AEs will be listed on the appropriate case report form for routine return to the TrAFFix central office. Serious AE (SAE) are defined as any untoward and unexpected medical occurrence that:

- Results in death,
- Is life-threatening,
- Requires hospitalisation or prolongation of existing inpatients´ hospitalisation,
- Results in persistent or significant disability or incapacity,
- Is a congenital anomaly or birth defect,
- Any other important medical condition which, although not included in the above, may require medical or surgical intervention to prevent one of the outcomes listed.

Some SAEs may be expected as part of the surgical interventions, and that do not need to be reported to the trial coordinating centre, provided that they are recorded on the 'complications' section of the CRF. These include:

- Wound infection
- Venous thromboembolic phenomena
- Blood transfusion
- Death
- Pneumonia
- Urinary tract infection
- Cerebrovascular accident
- Myocardial infarction/acute coronary syndrome
- Damage to a nerve, tendon or blood vessel

All other SAEs will be entered onto the Serious Adverse Event reporting form and sent to the TrAFFix office by fax or email within 24 hours of the PI (or delegated clinician) becoming aware of them. Once received, causality and expectedness will be confirmed by the Chief Investigator. SAEs that are deemed to be unexpected and related to the trial will be notified to the Research Ethics Committee (REC) within 15 days of being reported to the TrAFFix office. All such events will be reported to the Trial Steering Committee and Data Monitoring Committee at their next meetings.

All participants experiencing SAEs will be followed up as per protocol until the end of the trial.

4.8.2 Risks and benefits

The risks associated with this study are predominantly the risks associated with the surgery: infection, bleeding and damage to the adjacent structures such as nerves, blood vessels and tendons. Participants in both groups will undergo surgery and will potentially be at risk from any/all of these complications. There are no data to suggest that the risk is greater in one group or another. We believe that the overall risk profile is similar for the two interventions but assessment of the number of complications in each group is an objective of this trial.

4.9 End of trial

The end of the trial will be defined as the collection/receipt of the last follow-up questionnaire from the last participant.

For peer review only - http://bmjopen.bmj.com/site/about/guidelines.xhtml

For peer review only

For peer review only - http://bmjopen.bmj.com/site/about/guidelines.xhtml

5. Data Management

The Case Report Forms will be designed by the trial coordinator in conjunction with the trial management team. All electronic patient-identifiable information will be held on a secure, password-protected database at the University of Oxford, accessible only to the research team. Paper forms with patient-identifiable information will be held in secure, locked filing cabinets within a restricted area. On all other documentation, participants will be identified by a trial ID and the participant's initials only. Audio recordings of interview and participant-researcher interactions will be transcribed, and the anonymised transcriptions stored on secure servers at the University of Oxford, identified by a trial ID and initials only.

Direct access to source data/documents will be required for trial-related monitoring and/or audit by the Sponsor, NHS Trust or regulatory authorities as required. All paper and electronic data will be retained for at least five years after completion of the trial.

5.1 Statistical Analysis

This feasibility study is not powered to formally assess the size of the treatment effect, rather to estimate the recruitment rate. However, the totality of the data collected will be used to assess the feasibility of a definitive large RCT; recruitment rate being the driver of the feasibility study design on the basis that unless a reasonable recruitment rate can be achieved no formal trial will be possible. The recruitment rate will be estimated based on data collected and a 95% confidence interval determined for this measure.

If the estimated recruitment rate is such that a definitive trial is feasible then no formal analysis will be undertaken and data from the feasibility study will be locked and carried over into the main (definitive) trial. No formal analysis of treatment efficacy will be undertaken in this scenario. However, the study DSMC will see unblinded summary statistics, together with recruitment data, and will advise the TSC with relevant safety or ethical guidance as the study progresses. The reasons and patterns of any missing data, loss to follow-up and participant withdrawals will be carefully considered and reported, with particular emphasis on how these may impinge on the future trial.

If a definitive trial is not feasible, then outcome data will reported in the conventional manner. Baseline demographics (e.g. Age, Gender, cognitive status) will be compared between groups to ensure approximate balance has been achieved. This is a small study (n=52), so group treatment effects are unlikely to be estimated with much precision and consequently inferences will be tentative and reported as such. The main analysis will investigate differences in the primary outcome measure, EQ-5D-5L[21] score at 4 months, between the two treatment groups (nail and plate) on an intention-to-treat basis. In addition a per-protocol analysis will also be reported and early EQ-5D-5L status will also be assessed and reported at 6 weeks. Differences between groups will based on a normal approximation for EQ-5D-5L.[20 31] Tests will be two-sided and considered to provide evidence for a significant difference if p-values are less than 0.05 (5% significance level). The stratified randomisation procedure should ensure a balance in cognitive impairment and recruiting centre between test treatments. Although generally we have no reason to expect that clustering effects will be important for this study, in reality the data will be hierarchical in nature, with patients naturally clustered into groups by recruiting centre. Therefore we will account for this by generalising the conventional linear (fixed-effects) regression approach to a mixed-effects modelling approach; where participants are naturally grouped by recruiting centres (random-effects). This model will formally incorporate terms that allow for possible heterogeneity in responses for patients due to the recruiting centre, in addition to the fixed effects of the treatment groups, cognitive impairment and

For peer review only - http://bmjopen.bmj.com/site/about/guidelines.xhtml

other participant characteristics that may prove to be important moderators of the treatment effect (e.g. age and gender).

The main analyses will be conducted using specialist mixed-effects modelling functions available in the software package R (http://www.r-project.org/) where EQ-5D-5L[21] data will be assumed to be normally distributed; possibly after appropriate variance-stabilising transformation. The primary focus will be the comparison of the two treatment groups of patients, and this will be reflected in the analysis which will be reported together with appropriate diagnostic plots that check the underlying model assumptions. Results will be presented as mean differences between the trial groups, with 95% confidence intervals.

Secondary analyses will be undertaken using the above strategy for approximately normally distributed outcome measures such as DRI[23]. For dichotomous outcome variables, such as complications related to the trial interventions, mixed effects logistic regression analysis will be undertaken with results presented as odds ratios (and 95% confidence intervals) between the trial groups. The temporal patterns of any complications will be presented graphically and if appropriate a time-to-event analysis (Kaplan-Meier survival analysis) will be used to assess the overall risk and risk within individual classes of complications.

The reasons and patterns of any missing data, loss to follow-up and participant withdrawals will also be carefully considered and reported. A more detailed statistical analysis plan will be agreed with the Trial Steering Committee at the start of the study.

5.2 Economic evaluation

The feasibility of a future definitive economic evaluation of treatment with modern intramedullary nails or anatomical locking plates for fragility fractures of the distal femur will be investigated in this study. Unit costs for health and social care resources will be collected at 4 months via self-reported patient questionnaires and appropriate proxies (when necessary)[21 29] with the view to inform any future trial. The data collected in the participant questionnaires at each time point will also record indirect costs borne by participants and carers as a result of attending hospital visits; as well as direct non-medical costs (including travel expenses) attributable to their health state. Unit cost data will be obtained from national databases such as the BNF and PSSRU Costs of Health and Social Care.[41] EQ-5D-5L[21] and DEMQoL-U[24 33] will be collected at baseline (pre-injury status and current injury status) and 4 months following treatment allocation in order to estimate quality-adjusted life years (QALYs) in any future trial-based economic evaluation.

5.3 Process evaluation

Qualitative data collected to inform the process evaluation from interviews with patients, surgeons and other staff will be transcribed verbatim. The material will be organised into themes, using inductive coding. Using a constant comparative approach, these themes and their sub-themes will be used to produce a coding framework. The relationship between themes and sub-themes will be illustrated in a thematic map.

For peer review only - http://bmjopen.bmj.com/site/about/guidelines.xhtml

6. Trial Oversight

The day-to-day management of the trial will be the responsibility of the Trial Manager, supported by the CTU administrative staff. This will be overseen by the Trial Management Group, who will meet monthly to assess progress. It will also be the responsibility of the Trial Manager to undertake training of the research staff at each of the trial centres. The trial statistician and health economist will be closely involved in setting up data capture systems, design of databases and clinical reporting forms. A Trial Steering Committee (TSC) and an Independent Safety & Data Monitoring Committee (DSMC) will be set up.

6.1 Trial Supervision

Day-to-day management of the trial will be overseen by a Trial Management Group which is made up of the Investigators listed in Section 1 and staff working on the project within OCTRU. A TSC -with an independent Chairman - and DSMC will be set up.

The TSC, which includes independent members, provides overall supervision of the trial on behalf of the funder. Its terms of reference will be agreed with the HTA and will be drawn up in a TSC charter which will outline its roles and responsibilities. Meetings of the TSC will take place at least once a year during the recruitment period.

An outline of the remit of the TSC is to:

- monitor and supervise the progress of the trial towards its interim and overall objectives,
- review at regular intervals relevant information from other sources,
- consider the recommendations of the DSMC,
- inform the funding body on the progress of the trial.

The DSMC is a group of independent experts external to the trial who assess the progress, conduct, participant safety and, if required critical endpoints of a clinical trial.

The study DSMC will agree and adopt an appropriate charter which defines its terms of reference and operation in relation to oversight of the trial. They will not be asked to perform any formal interim analyses of effectiveness. They will, however, review accruing data, summaries of the data presented by treatment group, and will assess the screening algorithm against the eligibility criteria. They will also consider emerging evidence from other related trials or research and review related SAEs that have been reported. They may advise the chair of the Trial Steering Committee at any time if, in their view, the trial should be stopped for ethical reasons, including concerns about participant safety. DSMC meetings will be held at least annually during the recruitment phase of the study. Full details including names will be included in the DSMC charter.

6.2 Quality control

We will institute a rigorous programme of quality control. The trial coordinator will be responsible for ensuring adherence to the trial protocols at the trial sites. Quality assurance checks will be undertaken by the CTU to ensure integrity of randomisation, study entry procedures and data collection. The CTU has a quality assurance manager who will monitor this trial by conducting regular (at least once in the lifetime of the study, more if deemed necessary) inspections of the Trial Master File. Furthermore the processes of consenting, randomisation, registration, provision of information

For peer review only - http://bmjopen.bmj.com/site/about/guidelines.xhtml

and provision of treatment will be monitored. Written reports will be produced for the TSC, informing them if any corrective action is required.

6.3 Funding

This study is funded by the National Institute of Health Research Health Technology Assessment (15/59/22)

6.4 Insurance and Indemnity Arrangements

The Sponsor has a specialist insurance policy in place - Newline Underwriting Management Ltd, at Lloyd's of London - which would operate in the event of any participant suffering harm as a result of their involvement in the research. Standard NHS cover for negligent harm is in place for NHS procedures. There will be no cover for non-negligent harm.

6.5 Dissemination

The study monograph will be prepared by the trial management team at the completion of the trial. No patient identifiable information will be contained in any form of dissemination of study results.

For peer review only

For peer review only - http://bmjopen.bmj.com/site/about/guidelines.xhtml

6.6 Project Timetable and Milestones

We propose a 20 month study starting in June 2016. The trial timetable is shown below, with key milestones indicated and responsible parties identified:

Month	By date	Activity	Milestone	Responsibility
-4-0		Ethics submission	REC approval	CI/RF
0-2	June 2016	Start study	1st TSC/DSMC meeting	CI/TC
		Finalise trial protocol	Protocol final version	TMG
	July 2016	Complete CRF's	CRF final version	CI/Stat/TC
	Aug 2016	Start recruitment lead centre & feasibility centre 2	1st trial site open for recruitment	TC/CI
3-9	Oct 2016	Start recruitment at feasibility centres 3,4, 5& 6	All feasibility sites open for recruitment	TC/CI
	July 2017	End recruitment	~60 patients enrolled	
6-16	Nov 2017	Complete follow-up all sites	All patients completed follow-up	
17-18	Dec 2017	Statistical analysis		Stat
	Jan 2018	HE analysis		HE
		Reporting and full protocol development		TMG/HE
post	Feb 2018	Study close down	Final TSC/DSMC meeting	CI/TC
			HTA report	TMG

CI Chief Investigator, RF Research Fellow, TMG Trial management group, TM Trial Manager, TSC trial steering committee, DMEC Data monitoring and Ethics Committee, Stat statistician, HE Health Economist, DC Data Clerk

For peer review only

For peer review only - http://bmjopen.bmj.com/site/about/guidelines.xhtml

7. Protocol Amendments:

Amendment No.	Date of Amendment	Date of Approval
AM05	Modification of inclusion criteria, extension of recruitment period.	[TBC]

For peer review only

For peer review only - http://bmjopen.bmj.com/site/about/guidelines.xhtml

8. References

1. Court-Brown CM, Caesar B. Epidemiology of adult fractures: A review. Injury 2006;**37**(8):691-7.
2. Martinet O, Cordey J, Harder Y, et al. The epidemiology of fractures of the distal femur. Injury 2000;**31 Suppl 3**:C62-3.
3. Smith JRA HR, McArthur J, Morrison R, Yip G, Aquilina A, Kelly M. Distal femoral fractures: The size of the problem in the UK. Orthopaedic Trauma Society. Edinburgh, UK, 2013.
4. Johnell O, Kanis JA. An estimate of the worldwide prevalence, mortality and disability associated with hip fracture. Osteoporos Int 2004;**15**(11):897-902.
5. Bleibler F, Konnopka A, Benzinger P, et al. The health burden and costs of incident fractures attributable to osteoporosis from 2010 to 2050 in Germany--a demographic simulation model. Osteoporos Int 2013;**24**(3):835-47.
6. Griffin XL, Parsons N, Zbaeda MM, et al. Interventions for treating fractures of the distal femur in adults. Cochrane Database Syst Rev 2015;**8**:CD010606.
7. Griffin XL PN, Zbaeda M, McArthur J. Interventions for treating fractures of the distal femur in adults. Cochrane Database Systematic Reviews 2015;***in press***.
8. Zlowodzki M, Bhandari M, Marek DJ, et al. Operative treatment of acute distal femur fractures: systematic review of 2 comparative studies and 45 case series (1989 to 2005). J Orthop Trauma 2006;**20**(5):366-71.
9. Butt MS, Krikler SJ, Ali MS. Displaced fractures of the distal femur in elderly patients. Operative versus non-operative treatment. J Bone Joint Surg Br 1996;**78**(1):110-4.
10. Lu-Yao GL, Keller RB, Littenberg B, et al. Outcomes after displaced fractures of the femoral neck. A meta-analysis of one hundred and six published reports. J Bone Joint Surg Am 1994;**76**(1):15-25.
11. Harma A, Germen B, Karakas H, et al. The comparison of femoral curves and curves of contemporary intramedullary nails. Surgical and Radiologic Anatomy 2005;**27**(6):502-6.
12. Browner BD EC. The science and practice of intramedullary nailing. Philadelphia 1987;**Lea & Febiger**.
13. Miller DL, Goswami T, Prayson MJ. Overview of the locking compression plate and its clinical applications in fracture healing. J Surg Orthop Adv 2008;**17**(4):271-81.
14. Hoskins W SR, Bucknill A. Nails or plates for fracture of the distal femur? Data from the Victoria Orthopaedic Trauma Outcomes Registry. Bone Joint Journal 2016;**in press**.
15. Walters SJ, Brazier JE. Comparison of the minimally important difference for two health state utility measures: EQ-5D and SF-6D. Quality of life research : an international journal of quality of life aspects of treatment, care and rehabilitation 2005;**14**(6):1523-32.
16. Ware J, Jr., Kosinski M, Keller SD. A 12-Item Short-Form Health Survey: construction of scales and preliminary tests of reliability and validity. Med Care 1996;**34**(3):220-33.
17. Jennett B, Snoek J, Bond MR, et al. Disability after severe head injury: observations on the use of the Glasgow Outcome Scale. Journal of Neurology, Neurosurgery, and Psychiatry 1981;**44**(4):285-93.
18. Tornetta P, Egol KA, Ertl JP, et al. Locked plating versus retrograde nailing for distal femur fractures: A multicentre randomized trial. Orthopaedic Trauma Association 2013;**Phoenix**(Arizona).
19. Schulz KF, Altman DG, Moher D, et al. CONSORT 2010 statement: updated guidelines for reporting parallel group randomised trials. BMJ 2010;**340**:c332.

For peer review only - http://bmjopen.bmj.com/site/about/guidelines.xhtml

20. Griffin XL, Parsons N, Achten J, et al. Recovery of health-related quality of life in a United Kingdom hip fracture population. The Warwick Hip Trauma Evaluation--a prospective cohort study. Bone Joint J 2015;**97-B**(3):372-82.
21. Brooks R. EuroQol: the current state of play. Health Policy 1996;**37**(1):53-72.
22. Griffin XL, Achten J, Parsons N, et al. Platelet-rich therapy in the treatment of patients with hip fractures: a single centre, parallel group, participant-blinded, randomised controlled trial. BMJ Open 2013;**3**(6).
23. Salen BA, Spangfort EV, Nygren AL, et al. The Disability Rating Index: an instrument for the assessment of disability in clinical settings. J Clin Epidemiol 1994;**47**(12):1423-35.
24. Smith SC, Lamping DL, Banerjee S, et al. Development of a new measure of health-related quality of life for people with dementia: DEMQOL. Psychol Med 2007;**37**(5):737-46.
25. Roberts HC, Denison HJ, Martin HJ, et al. A review of the measurement of grip strength in clinical and epidemiological studies: towards a standardised approach. Age Ageing 2011;**40**(4):423-9.
26. Rockwood K, Song X, MacKnight C, et al. A global clinical measure of fitness and frailty in elderly people. CMAJ 2005;**173**(5):489-95.
27. Sherbourne CD, Stewart AL. The MOS social support survey. Soc Sci Med 1991;**32**(6):705-14.
28. Schwarzer R, Bäßler J, Kwiatek P, et al. The Assessment of Optimistic Self-beliefs: Comparison of the German, Spanish, and Chinese Versions of the General Self-efficacy Scale. Applied Psychology 1997;**46**(1):69-88.
29. Dolan P. Modeling valuations for EuroQol health states. Med Care 1997;**35**(11):1095-108.
30. Hounsome N, Orrell M, Edwards RT. EQ-5D as a quality of life measure in people with dementia and their carers: evidence and key issues. Value in health : the journal of the International Society for Pharmacoeconomics and Outcomes Research 2011;**14**(2):390-9.
31. Parsons N, Griffin XL, Achten J, et al. Outcome assessment after hip fracture: is EQ-5D the answer? Bone & joint research 2014;**3**(3):69-75.
32. Parsons H, Bruce J, Achten J, et al. Measurement properties of the Disability Rating Index in patients undergoing hip replacement. Rheumatology (Oxford) 2015;**54**(1):64-71.
33. Mulhern B, Rowen D, Brazier J, et al. Development of DEMQOL-U and DEMQOL-PROXY-U: generation of preference-based indices from DEMQOL and DEMQOL-PROXY for use in economic evaluation. Health Technol Assess 2013;**17**(5):v-xv, 1-140.
34. Marsh JL, Slongo TF, Agel J, et al. Fracture and dislocation classification compendium - 2007: Orthopaedic Trauma Association classification, database and outcomes committee. J Orthop Trauma 2007;**21**(10 Suppl):S1-133.
35. Cocks K, Torgerson DJ. Sample size calculations for pilot randomized trials: a confidence interval approach. J Clin Epidemiol 2013;**66**(2):197-201.
36. Dolan MM, Hawkes WG, Zimmerman SI, et al. Delirium on hospital admission in aged hip fracture patients: prediction of mortality and 2-year functional outcomes. J Gerontol A Biol Sci Med Sci 2000;**55**(9):M527-34.
37. Griffin XL, McArthur J, Achten J, et al. The Warwick Hip Trauma Evaluation Two -an abridged protocol for the WHiTE Two Study: An embedded randomised trial comparing the Dual-Mobility withpolyethylene cups in hip arthroplasty for fracture. Bone Joint Res 2013;**2**(10):210-3.
38. Griffin XL, McArthur J, Achten J, et al. The Warwick Hip Trauma Evaluation One -an abridged protocol for the WHiTE One Study: An embedded randomised trial

For peer review only - http://bmjopen.bmj.com/site/about/guidelines.xhtml

comparing the X-bolt with slidinghip screw fixation in extracapsular hip fractures. Bone Joint Res 2013;**2**(10):206-9.
39. Sims AL, Parsons N, Achten J, et al. The World Hip Trauma Evaluation Study 3: Hemiarthroplasty Evaluation by Multicentre Investigation - WHITE 3: HEMI - An Abridged Protocol. Bone Joint Res 2016;**5**(1):18-25.
40. Moore GF, Audrey S, Barker M, et al. Process evaluation of complex interventions: Medical Research Council guidance. BMJ 2015;**350**:h1258.
41. PSSR Unit., Curtis L. Unit Costs of Health and Social Care. Personal Social Services Research Unit, University of Kent & National Schedule of Reference Costs Year 2012-2013 DoH 2013.

For peer review only

For peer review only - http://bmjopen.bmj.com/site/about/guidelines.xhtml

TrAFFix

Trial of Acute Femoral fracture Fixation

Statistical Analysis Plan

Version 2.0 – 2Nov2016

Based on Protocol version 2.0 – 27Jul2016

Name	Title	Role (Author/Reviewer/Approver)	Signature	Date
Nick Parsons	Trial Statistician	**Author**	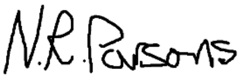	02Nov2016
Robin Lerner	Trial Manager	**Reviewer**	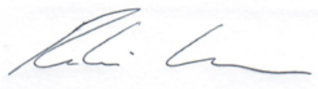	02Nov2016
Xavier Griffin	Chief Investigator	**Reviewer**	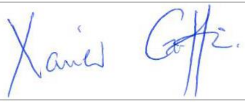	02Nov2016

Oxford Clinical Trials Research Unit (OCTRU)
and

Note: this document is version controlled and all draft versions and related communications should be stored in the Trial Master File

For peer review only - http://bmjopen.bmj.com/site/about/guidelines.xhtml

For peer review only

CONTENTS

1. INTRODUCTION ... 3
1.1 KEY PERSONNEL ... 3
2. BACKGROUND INFORMATION ... 3
2.1 RESEARCH HYPOTHESES AND STUDY OBJECTIVES ... 3
2.2 STUDY DESIGN AND TRIAL EXPECTED TIME-POINTS ... 3
2.3 ELIGIBILITY ... 4
2.4 TREATMENT INTERVENTIONS ... 4
2.5 SAMPLE SIZE ... 4
2.6 RANDOMISATION ... 5
2.7 HYPOTHESES AND DEFINITION OF PRIMARY AND SECONDARY OUTCOMES ... 5
2.8 OUTCOMES ASSESSMENT SCHEDULE ... 5
2.9 STATISTICAL ANALYSIS OUTLINE ... 5
3. QUALITY CONTROL AND DATA VALIDATION ... 7
3.1 DEFINITION OF DERIVED DATA ... 7
3.2 VALIDATION OF THE PRIMARY ANALYSIS ... 7
4. INTERIM ANALYSIS AND DATA REVIEW ... 7
5. SPECIFICATION OF STATISTICAL PACKAGES ... 7
6. DESCRIPTIVE ANALYSES ... 7
6.1 REPRESENTATIVENESS OF STUDY SAMPLE AND PATIENT THROUGHPUT ... 8
6.2 BASELINE COMPARABILITY OF RANDOMISED GROUPS ... 8
6.3 COMPARISON OF LOSSES TO FOLLOW-UP ... 8
6.4 DESCRIPTION OF AVAILABLE DATA ... 8
6.5 DESCRIPTION OF COMPLIANCE WITH INTERVENTION ... 9
6.6 UNBLINDING OF RANDOMISED TREATMENTS ... 9
6.7 RELIABILITY ... 9
7. DEFINITION OF POPULATIONS FOR ANALYSIS ... 9
8. ANALYSES TO ADDRESS PRIMARY AIMS ... 9
8.1 EVALUATION/DEFINITION OF PRIMARY OUTCOME (WHERE APPLICABLE) ... 9
8.2 STATISTICAL METHODS USED FOR ANALYSIS OF PRIMARY OUTCOME ... 9
8.3 ADJUSTMENT OF P VALUES FOR MULTIPLE TESTING ... 10
8.4 MISSING DATA ... 10
8.5 PRE-SPECIFIED SUBGROUP ANALYSIS ... 10
8.6 TREATMENT BY CENTRE INTERACTION ... 10
8.7 SENSITIVITY ANALYSIS ... 10
9. ANALYSIS TO ADDRESS SECONDARY AIMS ... 10
9.1 EVALUATION/DEFINITION OF SECONDARY OUTCOMES (WHERE APPLICABLE) ... 11
9.2 STATISTICAL METHODS USED FOR ANALYSIS OF SECONDARY OUTCOMES ... 11
9.1 PRE-SPECIFIED SUBGROUPS OF KEY SECONDARY OUTCOMES (WHERE APPLICABLE) ... 11
9.2 SENSITIVITY ANALYSIS OF KEY SECONDARY OUTCOMES (WHERE APPLICABLE) ... 11
9.3 HEALTH ECONOMICS AND COST EFFECTIVENESS ... 11
10. ADDITIONAL ANALYSES ... 11
10.1 EXPLORATORY ANALYSES ... 11
10.2 BLINDED ANALYSIS ... 11
10.3 META-ANALYSES (IF APPLICABLE) ... 12
11. SAFETY ANALYSIS ... 12

For peer review only - http://bmjopen.bmj.com/site/about/guidelines.xhtml

12. APPENDIX: GLOSSARY OF ABBREVIATIONS 12
13. DOCUMENT HISTORY 12
14. CHANGES FROM PREVIOUS VERSION OF SAP 12

1. INTRODUCTION

This document details the proposed presentation and analysis for the main paper(s) reporting results from the ***NIHR HTA funded Trial of Acute Femoral Fracture Fixation.*** The results reported in these papers should follow the strategy set out here. Subsequent analyses of a more exploratory nature will not be bound by this strategy, though they are expected to follow the broad principles laid down here. The principles are not intended to curtail exploratory analysis (for example, to decide cut-points for categorisation of continuous variables), nor to prohibit accepted practices (for example, data transformation prior to analysis), but they are intended to establish the rules that will be followed, as closely as possible, when analysing and reporting the trial.

The analysis strategy will be available on request when the principal papers are submitted for publication in a journal. Suggestions for subsequent analyses by journal editors or referees, will be considered carefully, and carried out as far as possible in line with the principles of this analysis strategy; if reported, the source of the suggestion will be acknowledged.

Any deviations from the statistical analysis plan will be described and justified in the final report of the trial. The analysis should be carried out by an identified, appropriately qualified and experienced statistician, who should ensure the integrity of the data during their processing. Examples of such procedures include quality control and evaluation procedures.

1.1 Key personnel

List of key people involved in the drafting and reviewing this SAP, together with their role in the trial and their contact details.

Author(s) (Trial statistician(s)):

Nick Parsons – Senior Statistician, Trial Statistician

Reviewers (Chief Investigator, Trial Manager, DSMC, TSC, Statistician as appropriate):

Xavier Griffin – Chief Investigator

Approver (Senior Statistician):

Nick Parsons – Senior Statistician, Trial Statistician

2. BACKGROUND INFORMATION

2.1 Research Hypotheses and Study Objectives

1. Assess the feasibility of a future definitive trial.
2. Perform a process evaluation to understand the generalisability and likely success of a future trial.
3. Explore the validity of self and proxy-reporting of the EQ-5D-5L in this specific population.

2.2 Study Design and trial expected time-points

The study design including the type of trial (e.g. parallel group, cluster, crossover, factorial, single arm), single/multi-centre, blinding, allocation ratio, trial framework (e.g. superiority, equivalence, non-inferiority, exploratory), primary outcome and crucial trial time-points.

For peer review only - http://bmjopen.bmj.com/site/about/guidelines.xhtml

TRAFFix is a randomized (1:1 allocation ratio) controlled multicentre trial with two (parallel) groups assessing fixation of the femur after fixation using either a nail or a plate.

Date of grant activation	01Jun2016
Date of start of recruitment:	XXAug2016
Date of expected end of recruitment:	31May2017
Date expected end follow-up:	30Sep2016
Date expected start of analysis:	01Oct2017
Date End of grant:	31Nov2017
Target number of subjects:	52
Participating Centres:	6

2.3 Eligibility

Inclusion criteria

Patients will be eligible for this study if they:

- are ≥50 years old as a surrogate for bone-density and therefore fragility fracture,
- have a fracture of the femur involving the distal two "Muller" squares,
- would, in the opinion of the attending surgeon, benefit from internal fixation of the fracture.

Exclusion criteria

Patients will be excluded from this trial if they have:

- a loose knee or hip arthroplasty requiring revision,
- pre-existing femoral deformity,
- an arthroplasty that precludes nail fixation.

2.4 Treatment Interventions

Intramedullary nailing: Fixation of the fracture will be achieved with a proximally and distally locked nail that spans the entire diaphysis of the femur. All nails will be introduced retrograde through the knee joint. In this pragmatic trial, the details of surgical incision and approach, fracture reduction and supplementary fixation with wires or screws will be at the surgeon's discretion as per their normal clinical practice.

Locking plate fixation: Fixation of the fracture will be achieved with anatomical distal femoral locking-plate and screws. Locking plates will be defined as those in which at least one fixed angle locking screw is placed distal to the fracture. The operating surgeon will determine the length, number and type of additional screws. Additional fixation with lag screws and cerclage wires will be at the surgeon's discretion. In this pragmatic trial, the details of surgical incision and approach, fracture reduction, number and type of other screws and supplementary fixation with wires or screws will be at the surgeon's discretion as per their normal clinical practice.

2.5 Sample Size

Details of the sample size for primary outcomes and any co-primary or important key secondary outcomes (if applicable), including treatment effect, power, levels of statistical significance (one-tailed or two-tailed), clinical relevance and justification.

Data from the TrAFFix feasibility study will be used to calculate estimates of the standard deviation of the primary outcome measure (EQ-5D-5L) to drive a formal power analysis and sample size calculation for the

For peer review only - http://bmjopen.bmj.com/site/about/guidelines.xhtml

future definitive trial; evidence from other relevant sources reporting the metrics properties of EQ-5D-5L in the study population will also be used to inform this process. We anticipate that each of 6 participating centres will treat approximately 1.5 eligible patients per month. Taking a conservative approach, we would expect, to ensure feasibility, to be able to recruit 1.0 patients per month per centre. Given our schedule for centre opening times and recruitment length, we will have 52 centre months available to recruit for this study. Assuming that the recruitment rate is 1.0 per month per centre and monthly centre counts of patient recruitment numbers are approximately Poisson distributed and independent of one another, then this will allow us to estimate the recruitment rate with a 95% confidence interval of 0.73 - 1.28.[35] Therefore recruiting 52 patients in total should provide sufficiently precise estimates of the monthly recruitment rate to decide if a definitive trial is feasible.

2.6 Randomisation

The method(s) of randomisation, including the randomisation allocation ratio and any stratification/minimisation factors.

The unit of randomisation will be the individual patient. Randomisation will have a 1:1 allocation to two intervention groups (Nail and Plate) and be stratified by recruiting centre (6 centres will be used) and chronic cognitive impairment (Yes or No). Within each strata a fixed block size of 4 will be used for all blocks.

Full details of the randomisation are available in TrAFFix_RBP_v1.0_16Aug2016, stored in the confidential statistical section of the TMF.

2.7 Hypotheses and Definition of Primary and Secondary Outcomes

Clear description of hypotheses and definitions of the primary and secondary outcome measures and, when applicable, any methods used to enhance the quality of measurements (e.g., multiple observations, training of assessors). It might be useful to specify types of data that will be encountered in this trial.

The principle aim of this feasibility study will be to determine the number of eligible and recruited patients in the trauma centres over the course of 10 months. The primary outcome measures for this study are the participant recruitment rate and the completion rate of the EQ-5D-5L at 4 months post-surgery.

2.8 Outcomes Assessment Schedule

**Outcomes**	Baseline	6-weeks	4-months
EQ-5D-5L	Pre + post injury	X	X
DEMQoL[A]	Pre + post injury	X	X
DRI[B]	Pre + post injury	X	X
Radiographs	X	X	
Complications	X	X	X
Health Economics			X
Qualitative interviews[C]	X	X	X

2.9 Statistical Analysis Outline (references refer to protocol)

Main statistical analysis

This feasibility study is not powered to formally assess the size of the treatment effect, rather to estimate the recruitment rate. The totality of the data collected will be used to assess the feasibility of a definitive large RCT; recruitment rate being the driver of the feasibility study design on the basis that unless a reasonable recruitment rate can be achieved no formal trial will be possible. The recruitment rate will be estimated based on data collected and a (Poisson distributed) 95% confidence interval determined for this measure.

If the estimated recruitment rate is such that a definitive trial is feasible then no formal analysis will be undertaken and data from the feasibility study will be locked and carried over into the main (definitive) trial. No formal analysis of treatment efficacy will be undertaken in this scenario. However, the study ISDMC will

For peer review only - http://bmjopen.bmj.com/site/about/guidelines.xhtml

see unblinded summary statistics, together with recruitment data, and will advise the TSC with relevant safety or ethical guidance as the study progresses. The reasons and patterns of any missing data, loss to follow-up and participant withdrawals will be carefully considered and reported, with particular emphasis on how these may impinge on the future trial.

If a definitive trial is not feasible, then outcome data will reported in the conventional manner. Baseline demographics (e.g. Age, Gender, cognitive status) will be compared between groups to ensure approximate balance has been achieved. This is a small study (n=52), so treatment group effects are unlikely to be estimated with much precision and consequently inferences will be tentative and reported as such. The main analysis will investigate differences in the primary outcome measure, EQ-5D-5L[21] score at 4 months, between the two treatment groups (Nail and Plate) on an intention-to-treat basis. In addition a per-protocol analysis will also be reported and early EQ-5D-5L status will also be assessed and reported at 6 weeks. Differences between groups will based on a normal approximation for EQ-5D-5L.[20 31] Tests will be two-sided and considered to provide evidence for a significant difference if p-values are less than 0.05 (5% significance level). The stratified randomisation procedure should ensure a balance in cognitive impairment and recruiting centre between test treatments. Although generally we have no reason to expect that clustering effects will be important for this study, in reality the data will be hierarchical in nature, with patients naturally clustered into groups by recruiting centre. Therefore we will account for this by generalising the conventional linear (fixed-effects) regression approach to a mixed-effects modelling approach; where participants are naturally grouped by recruiting centres (random-effects). This model will formally incorporate terms that allow for possible heterogeneity in responses for patients due to the recruiting centre, in addition to the fixed effects of the treatment groups, cognitive impairment and age and gender, as these latter participant characteristics may prove to be important moderators of the treatment effect. The mixed-effects model will be the primary analysis, and will be reported as such.

The main analyses will be conducted using specialist mixed-effects modelling functions available in the software package R (http://www.r-project.org/) where EQ-5D-5L[21] data will be assumed to be normally distributed; possibly after appropriate variance-stabilising transformation. The primary focus will be the comparison of the two treatment groups of patients, and this will be reflected in the analysis which will be reported together with appropriate diagnostic plots that check the underlying model assumptions. Results will be presented as mean differences between the trial groups, with 95% confidence intervals.

Secondary analyses will be undertaken using the above strategy for other approximately normally distributed outcome measures such as DRI[23]. For dichotomous outcome variables, such as complications related to the trial interventions, mixed effects logistic regression analysis will be undertaken with results presented as odds ratios (and 95% confidence intervals) between the trial groups. The temporal patterns of any complications will be presented graphically and if appropriate a time-to-event analysis (Kaplan-Meier survival analysis) will be used to assess the overall risk and risk within individual classes of complications. The reasons and patterns of any missing data, loss to follow-up and participant withdrawals will also be carefully considered and reported.

A proportion of study participants will be unable to self-report EQ-5D, often due to dementia, so an appropriate individual (e.g. carer, relative) will be asked to proxy-report. The Dementia Quality of Life Measure (DEMQoL) is a validated questionnaire specifically designed to assess quality of life in patients with dementia. DEMQoL can be self or proxy-reported (28 or 31 items respectively). In order to assess the relative performance and merits of EQ-5D and DEMQoL in the study population, these two measures will be compared in the subgroup of study participants who are unable to self-report EQ-5D. Given the relatively small size of this study, and minority of participants who will be unable to self-report, there will not be enough data to undertake formal statistical testing for differences between measures. However, graphical presentation of the distribution and relationship (correlation) between measures for individuals will provide will allow some

For peer review only - http://bmjopen.bmj.com/site/about/guidelines.xhtml

assessment to be made of the merits and metric properties (e.g. variance) of the two measures. Agreement or moderate to strong correlation between measures will indicate that they are measuring the same underlying (latent) trait (which we take to be the true QoL of the study participants). Utilities will also be calculated for the two measures and compared in a similar manner.

Economic Evaluation

The feasibility of a future definitive economic evaluation of treatment with modern intramedullary nails or anatomical locking plates for fragility fractures of the distal femur will be investigated in this study. The analysis plan for the health economics evaluation will be detailed in a separate analysis plan.

Process evaluation

Qualitative data collected to inform the process evaluation from interviews with patients, surgeons and other staff will be transcribed verbatim. The analysis plan for the process evaluation will be detailed in a separate analysis plan.

3. QUALITY CONTROL AND DATA VALIDATION

Quality control and data validation will be carried out in accordance with OCTRU SOPs and TrAFFix monitoring and data management plans.

TrAFFix Monitoring Plan V1.0 21Jul2016:

Z:\KC_TRAFFIX\TRAFFIX eTMF V3.0\Risk assessment and Monitoring\2. Monitoring Plan

TrAFFix Data Management Plan V1.0 21Jul2016

Z:\KC_TRAFFIX\TRAFFIX eTMF V3.0\Data Management\Data Management Plan

3.1 Definition of Derived Data

Definitions of any data derived for analysis outlined here with full details stored in the Data and Sharing Management Plan (see OCTRU SOP DM-008 Data and Sharing Management Plan).

3.2 Validation of the Primary analysis

The primary outcome and key secondary outcomes will be analysed, following the analyses detailed in this SAP, by a statistician independent of the trial using different statistical software (if possible). Any discrepancies will be reported in the Statistical report (See OCTRU SOP STATS-005 Statistical Report).

4. INTERIM ANALYSIS AND DATA REVIEW

A brief summary of the format, remit and time points for meetings of the Data and Safety Monitoring Committee including any planned formal interim analyses.

The Data and Safety Monitoring Committee is independent from sponsor and follows the TrAFFix_DSMC_Charter_V1.0_02Sep2016. No interim analyses are planned.

5. SPECIFICATION OF STATISTICAL PACKAGES

All analysis will be carried out using appropriate validated statistical software such as STATA, SAS, SPLUS or R statistical software. The relevant package and version number will be recorded in the Statistical report.

6. DESCRIPTIVE ANALYSES

Summary of flow of trial participants through the trial and baseline stratification, demographic and clinical characteristics of each group.

For peer review only - http://bmjopen.bmj.com/site/about/guidelines.xhtml
For peer review only

6.1 Representativeness of Study Sample and Patient Throughput

The flow of participants through each stage of the trial, including numbers of participants randomly assigned, receiving intended treatment, completing the study protocol, and analysed for the primary outcome is provided following the appropriate guideline (e.g. CONSORT). Protocol violations/deviations and information relating to the screening data including the number of ineligible patients randomised, together with reasons.

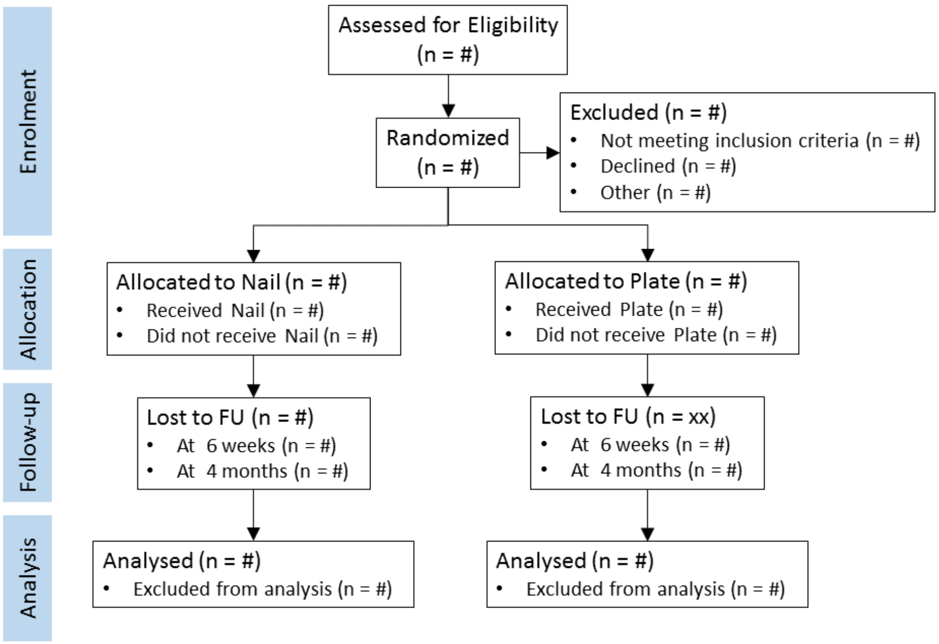

6.2 Baseline Comparability of Randomised Groups

Baseline characteristics are reported by treatment group, including the stratification/minimisation factors (if applicable) and important prognostic, demographic and clinical covariates).

Numbers (with percentages) for binary and categorical variables and means (and standard deviations), or medians (with lower and upper quartiles) for continuous variables will be presented; there will be no tests of statistical significance nor confidence intervals for differences between randomised groups on any baseline variable.

6.3 Comparison of Losses to Follow-up

Description of loss to follow-up together with reasons. Statistical tests comparing loss to follow-up to be undertaken and how these will be reported.

Loss to follow-up together with reasons will be reported by intervention arm. To assess differential losses between the groups this will be tested using absolute risk differences (95% confidence interval) and a chi-squared test. Any deaths (and their causes) will be reported separately.

6.4 Description of Available Data

The completeness of data required for primary and secondary outcomes is described, detailing methods to limit the possibility of missing data and methods for dealing with missing data when it occurs.

It seems likely that some data may not be available due to voluntary withdrawal of patients, lack of completion of individual data items or general loss to follow-up. Where possible the reasons for data missingness will be ascertained and reported. Although missing data are not expected to be a problem for this study, the nature

For peer review only - http://bmjopen.bmj.com/site/about/guidelines.xhtml

and pattern of the missingness will be carefully considered — including in particular whether data can be treated as missing completely at random (MCAR).

6.5 Description of Compliance with Intervention

Description of compliance with intervention – may include important covariates related to the interventional procedure or the standard treatment used in both arms.

A summary of the treatment received, as distinct from the treatment allocated, will be provided for each study participant. This is a pragmatic study, so detailed implementation of the study interventions will be left to the individual surgeons and will reflect their usual practice.

6.6 Unblinding of Randomised Treatments

Detail who is blinded in the study (participants, local site staff, outcome assessors). All cases of treatment unblinding will be listed, together with reasons and summarised (numbers, percentages)

As the surgical scars are clearly visible, the patients cannot be formally blinded to their treatment. Participants will only be informed of their treatment allocation at the end of the trial. In addition, the treating surgeons will also not be blind to the treatment, but will take no part in the post-operative assessment of the patients. The functional outcome data will be collected and entered onto the trial central database by a research assistant/data clerk in the trial central office.

6.7 Reliability

The radiographs collected will be reviewed by independent researchers at each hospital, A small sample of EQ-5D-5L utility scores will be checked by hand calculation to ensure that the computer algorithm has been implemented correctly.

7. DEFINITION OF POPULATIONS FOR ANALYSIS

Populations for analysis are defined as follows:

Intent to treat (ITT): all participants randomised in their randomised groups.

Per protocol (PP): all participants grouped by the intervention they received, rather than to which they were allocated. Participants receiving neither intervention will be excluded from the PP analysis

Safety: All participants who started the intervention or received a minimum amount of treatment as defined in the protocol.

8. ANALYSES TO ADDRESS PRIMARY AIMS

Statistical methods to be used to compare groups for primary outcome(s) and methods for point and interval estimation. Include methods for additional analyses, such as adjusted analyses and subgroup analyses, together with which populations will be analysed.

8.1 Evaluation/Definition of Primary Outcome (where applicable)

Criteria of evaluation and definition of outcomes and methods of calculation (rules for calculation of derived variables including definitions that can be programmed from the data).

Recruitment rate.

8.2 Statistical Methods Used for Analysis of Primary Outcome

Description of the primary and secondary/supplementary analyses and primary and secondary time-points (if applicable), including details of how results will be reported, both in tabular and graphical format.

For peer review only - http://bmjopen.bmj.com/site/about/guidelines.xhtml
For peer review only

The overall study recruitment rate will be estimated based on data collected and a 95% confidence interval determined for this measure. If the estimated recruitment rate is such that a definitive trial is feasible then no formal analysis will be undertaken and data from the feasibility study will be locked and carried over into the main (definitive) trial.

8.3 Adjustment of P values for Multiple Testing

Acknowledgement of the issue of multiple testing (if applicable), together with the rationale of the intention formally or informally adjust for multiplicity. If no adjustment planned this will be stated.

There is no multiple testing as only a single primary outcome is considered. Therefore significance levels used will be set at the conventional 5% level. Interim analyses of primary and secondary endpoints will not be carried out unless requested by the DSMC, who will formulate a plan for maintaining the overall study type I error rate if this is the case.

8.4 Missing Data

A description of methods utilised for dealing with missing, spurious (outliers) and unused data during statistical analysis including the type of missing data. If multiple imputation is to be used the methods will be specified. Methods for handling withdrawals and protocol deviations will be documented. State if no missing data adjustment will be used. Where multiple imputation is utilised the methods used to test the validity of the different assumptions will be described, either here or under the sensitivity analyses section.

A qualitative assessment will be made of the amount and pattern of any missing data and how this may affect the feasibility and or design of the main study.

8.5 Pre-specified Subgroup Analysis

Any pre-specified subgroup analyses will be described, together with the justification for relevance and importance and will include methods of analysis and presentation of the results. It is recommended that subgroup-treatment interaction methods are used with presentation using forest plots. This is usually included in the protocol, but additional subgroup analyses may be added before the final datalock following the blinded analysis or publication of other trials/research.

No subgroup analyses are planned

8.6 Treatment by Centre Interaction

Consistency of effect will be assessed across the 6 centres by informal examination of the within centre effects. There will be limited capacity to investigate these formally and it is noted that such centre effects are expected by chance.

8.7 Sensitivity Analysis

Describe any analysis utilised to check the robustness of the results. This may include repeating the primary analysis for different patient populations as defined in section 8.

No sensitivity analyses are planned.

Note: Sensitivity analyses for checking validity of multiple imputation assumptions should be detailed either here or in Section 9.4.

9. ANALYSIS TO ADDRESS SECONDARY AIMS

A list of the secondary outcomes will be provided with a separate section for each outcome with full definitions and analyses methods, together with details of the populations to be analysed and presentation of results will be provided. Sometimes the statistical method will be described first in full and then for each secondary outcome the method will be referred to in brief.

For peer review only - http://bmjopen.bmj.com/site/about/guidelines.xhtml

9.1 Evaluation/Definition of Secondary Outcomes (where applicable)

Not applicable.

9.2 Statistical Methods Used for Analysis of Secondary Outcomes

Statistical methods used to compare groups for secondary outcome(s); Methods for additional analyses, such as subgroup analyses and adjusted analyses.

The primary aim of this feasibility study is to assess the recruitment rate. Secondary outcomes will be collected;

Disability Rating Index (DRI), Dementia Quality of Life Measure (DEMQoL), EuroQol 5 Dimensions (5L) Score (EQ-5D-5L), self-efficacy, frailty, grip strength and complications. Methods for analysis of these variables are provided in Section 2.9. If a definitive trial is deemed feasible, then no analysis of these data will be undertaken and data will be carried over into the main study. However, if the main study is not deemed feasible, then outcome data will reported in the conventional manner.

9.1 Pre-specified Subgroups of Key Secondary Outcomes (where applicable)

Any pre-specified subgroup analyses of key secondary outcomes will be described, together with the justification for relevance and importance and will include methods of analysis, populations to be analysed and presentation of the results. This is usually included in the protocol, but additional subgroup analyses may be added before the final datalock following the blinded analysis or publication of other trials/research.

Not applicable.

9.2 Sensitivity Analysis of Key Secondary Outcomes (where applicable)

Describe any analysis utilised to check the robustness of the results for key secondary outcomes. See section 8.7.

Not applicable.

9.3 Health Economics and Cost Effectiveness

A summary from the protocol will be provided with reference to the relevant separate analysis plan (if applicable).

The statistician is not undertaking this analysis.

10. ADDITIONAL ANALYSES

10.1 Exploratory analyses

Pre-specified exploratory outcomes and analyses

Any outcomes and analyses pre-specified as exploratory in the protocol – detailed definition and analysis methodology will be provided or separate documents referred to together with who will perform these.

Additional Exploratory Analysis Not Specified Prior to Receiving Data

Any analyses not specified in the analysis protocol will be exploratory in nature and a significance level of 0.01 will be used to declare statistical significance. 99% confidence intervals will be presented.

10.2 Blinded analysis

Details of a blinded analysis will be undertaken prior to the final datalock will be provided, together with the rationale for undertaking this. If no blinded analysis to be undertaken, then state this.

No blinded analysis will be undertaken.

For peer review only - http://bmjopen.bmj.com/site/about/guidelines.xhtml
For peer review only

10.3 Meta-analyses (if applicable)

If meta-analysis is planned detail which trials will be included, which outcomes will be combined and whether random or fixed effects will be utilised. If this is under a separate protocol/analysis plan then reference this here.

Not applicable.

11. SAFETY ANALYSIS

Briefly outline the safety reporting from the protocol – this may be explicitly included as a secondary outcome – but include here. Describe how it will be summarised and compared (if applicable). Will adverse events/complications also be recorded, summarised and analysed – full details can be included here including dummy tables.

All complications and adverse events will be recorded. Complications will be classified as either: (a) unrelated to the trial protocol, (b) related systemic complications (including venous thromboembolic phenomena, death, pneumonia, urinary tract infection, blood transfusion, acute cerebrovascular incident, acute cardiac event, other) or (c) related local complications (superficial/deep infection, non/mal union, failure/removal/revision of metalwork, injury to adjacent structures such as nerves/tendons/blood vessels, other). The analysis will be conducted by intention to treat. Serious adverse events are defined as those that are fatal, life threatening, disabling or require hospitalisation or prolongation of hospitalisation.

12. APPENDIX: GLOSSARY OF ABBREVIATIONS

SAP Statistical Analysis Plan

DSMC Data and Safety Monitoring Committee

TSC Trial Steering Committee

CI Chief Investigator

13. DOCUMENT HISTORY

14. CHANGES FROM PREVIOUS VERSION OF SAP

A summary of key changes from earlier versions of SAP, with particular relevance to protocol changes that have an impact on the design, definition, sample size, data quality/collection and analysis of the outcomes will be provided. Include protocol version number and date.

Version number Issue date	Author of this issue	Protocol Version & Issue date	Significant changes from previous version together with reasons
V1.0_19Sep2016	NP	Protocol_V2.0_27Jul2106	Not applicable as this is the 1st issue
V2.0_2Nov2016	NP	Protocol_V2.0_27Jul2106	Paragraph added to describe comparison between EQ-5D and DEMQoL
			Add to or delete as required

For peer review only - http://bmjopen.bmj.com/site/about/guidelines.xhtml

Supplemental table 1: Reasons for non-inclusion and mechanisms of injury returned by participating hospitals. $The eligible age was changed from 50 to 18 during the study. Site names have been removed, *denotes major trauma centre.

	Site ID							
**Reason ineligible**	**A***	**B**	**C**	**D***	**E**	**F***	**G***	**Total**
Patient 50 or 18 years old$	3	0	2	6	0	4	3	**18**
Patient managed non-operatively	2	18	2	1	3	0	5	**31**
Pre-existing femoral deformity	1	0	1	0	0	0	0	**2**
Pre-existing arthroplasty	4	2	3	5	6	6	7	**33**
Other:	1	0	0	0	0	3	0	**4**
**Reason missed**								
Staffing/weekend	0	0	1	1	0	3	0	**5**
Technical (RRAMP fault)	0	0	0	0	1	0	0	**1**
Unknown (added from monitoring)	1	0	0	0	4	0	10	**15**
Surgeon preference	10	15	5	3	1	5	0	**39**
**Reason declined**								
Patient doesn't want to be part of research	0	0	1	0	0	0	0	**1**
Consultee doesn't want to be part of research	0	1	0	0	0	0	0	**1**
**Mechanism of Injury (of eligible patients)**								
Fall from <2m	15	5	6	6	9	5	9	**55**
Fall from >2m	1	0	0	0	0	2	0	**3**
Other	4	0	1	1	0	4	1	**11**
**Mechanism of Injury (of ineligible patients)**								
Fall from <2m	7	0	6	5	9	7	11	**45**
Fall from >2m	0	0	0	0	0	1	0	**1**
Other	3	0	2	7	0	5	4	**21**
Not reported	1	20	0	0	0	0	0	**21**

For peer review only

Supplemental table 2: Overview of process evaluation findings

Item	Component	Data Sources	Quantitative Findings	Qualitative findings
Reach	• Number of eligible patients reached	• Screening logs<br>• Enhanced screening	• 23 of 85 eligible patients were included in the study<br>• 82 patients with distal femoral fractures were not recorded on the initial screening logs<br>• The number of distal femoral fractures missing from the screening logs varied from 0 to 34 between the centres	• Staff were confident that few patients were eligible for the study, suggesting that they were unaware that potentially eligible patients were being missed
Fidelity	• Procedure for screening patients<br>• Application of the study eligibility criteria	• Screening logs<br>• Enhanced screening<br>• Interviews with staff	• 82 patients who were admitted to the participating centres with a distal femoral fracture during the recruitment period were not included on the screening logs<br>• 39 eligible patients were excluded due to surgeon preference	• Clinical teams did not always notify the research teams of eligible patients<br>• In two centres, RAs did not attend the daily trauma meeting, where patient eligibility could be discussed<br>• Staff emphasised that there were fewer patients with distal femur fractures than they had expected and reported that many were ineligible<br>• Some staff felt that many surgeons had strong preferences for one of the two interventions and were unwilling to randomise patients<br>• These qualitative findings are linked to the themes i) making it work categories (category: research culture) and ii) knowing it's the right decision (category: making sense of the eligibility criteria)
	• Consent discussion	• Audio – recordings of patient staff interactions	• N/A	• When discussing the study with patients, RAs emphasised that both treatments are routinely used and that their surgeon was happy for them to receive either intervention<br>• They also avoided jargon and described the study in simple terms, using phrases such as '50/50', 'computer decides' and 'heads or tails' to explain randomisation

		• Interviews with RAs and participants		• These findings are linked to the theme: navigating research with patients after orthopaedic trauma (categories: making sense of the study and enabling participation)
	• Deviations from protocol/ application of operative procedures	• CRFs<br>• Interviews with staff	• Two protocol deviations were recorded. One patient allocated a plate received a nail and one patient allocated a nail received a plate	• Some staff described strategies that may help avoid cross overs in surgical trials. If the treating surgeon was uncomfortable or unwilling to perform one of the interventions then another surgeon could take that patient onto their operating list instead.<br>• These findings are linked to the themes i) making it work (category: research culture) and ii) knowing it's the right decision (category: clinical uncertainty)
	• Delivery of post-operative rehabilitation components<br>• Patient compliance with postoperative	• CRFs<br>• Interviews with participants and surgeons	• Eight patients were instructed to weight bear as tolerated, three were instructed to weight bear as required to allow mobilisation, seven partial weight bear and five non-weight bear.<br>• At six week follow-up, six of 15 participants who completed the questionnaire were weight bearing and nine were not.<br>• At four month follow-up, nine of the 14 participants who completed the questionnaire were weight bearing and five were not.	• Surgeons held different views about the most appropriate rehabilitation instructions for patients with this fracture but the majority favoured early weight bearing<br>• For participants, rehabilitation was typically slow and for some it was a frustrating process.<br>• Loss of confidence and pain hindered rehabilitation for some participants
Acceptability	• Acceptability of Interventions	• Screening logs<br>• Interviews with participants and staff	• 39 eligible patients were excluded due to surgeon preference	• Some staff felt that many surgeons had strong preferences for one of the two interventions and were unwilling to randomise patients<br>• Surgeons' unwillingness to randomise patients stemmed from their beliefs about whether both methods of fixation were appropriate for a patients' specific fracture and their own surgical skills

For peer review only

				• Participants tended to be less concerned about the nature of the interventions as long as their fracture was treated appropriately.<br>• The majority of participants recalled that the study involved a plate vs nail<br>• Staff understood that it would be difficult for patients to appreciate the differences between these two interventions and that that the type of metalwork used to fix their fracture was unlikely to be a priority for patients<br>• These findings are linked to the themes i) knowing it's the right decision (categories: surgeon preference and clinical equipoise), and ii) navigating research after orthopaedic trauma (category: making sense of the study)
	• Acceptability of study procedures	• Screening logs<br>• Interviews with participants and staff	• Two patients declined to participate in the study<br>• Two of seven patients who were entered in the study under nominated consultee consent declined when approached for retrospective consent to continue in the study after surgery<br>• One participant withdrew from the study after surgery	• RAs found that in the majority of cases patients were accepting of the nominated consultee consent procedure<br>• The majority of staff found randomisation to be acceptable to most patients<br>• When randomisation was described to participants during their interview, they typically seemed to be accepting of this procedure or indifferent towards it<br>• Several participants demonstrated a misunderstanding of randomisation at interview<br>• These findings link to the theme: navigating research with patients after orthopaedic trauma (categories: making sense of the study and enabling participation)
How is delivery achieved?	• Facilitators and barriers in the delivery of the study	• Interviews with staff	• N/A	• The overarching concept of facilitating trial recruitment was identified from our analysis of the interview data. This concept aids our understanding of how delivery was achieved by highlighting facilitators and barriers to delivering complex trauma trails such as TrAFFix.<br>• Themes comprising this concept are navigating research with patients after orthopaedic trauma, ii) knowing it's the right decision, and iii) making it work

For peer review only

Supplemental table 3: Overview of process evaluation findings

Existing Factors	Injury and Treatment	Post-discharge factors
• Age<br>• Comorbidities<br>• Cognition<br>• Pre-injury mobility<br>• Pre-injury activity<br>• Nutrition<br>• Psychological factors (emotional resilience, self-efficacy, depression)	• Injury event<br>• Surgeon experience<br>• Surgeon preference<br>• Surgeon beliefs about rehabilitation<br>• Experience and care in hospital<br>• Communication – clear language<br>• Team work	• Transitions (e.g. from hospital to home)<br>• Appropriate discharge residence<br>• Follow-up<br>• Rehabilitation<br>• Pain<br>• Confidence<br>• Family (living arrangements, advocacy)<br>• Practical support<br>• Social support<br>• Changes to lifestyle after injury<br>• Loss of dignity<br>• Loss of independence<br>• Funding and access to social care<br>• Holistic approach to care<br>• Recovery pathways<br>• Balance of care between family and the system

For peer review only

CONSORT 2010 checklist of information to include when reporting a pilot or feasibility trial*

Section/Topic	Item No	Checklist item	Reported on page No
**Title and abstract**			
	1a	Identification as a pilot or feasibility randomised trial in the title	1
	1b	Structured summary of pilot trial design, methods, results, and conclusions (for specific guidance see CONSORT abstract extension for pilot trials)	2
**Introduction**			
Background and objectives	2a	Scientific background and explanation of rationale for future definitive trial, and reasons for randomised pilot trial	3
	2b	Specific objectives or research questions for pilot trial	2
**Methods**			
Trial design	3a	Description of pilot trial design (such as parallel, factorial) including allocation ratio	3-4
	3b	Important changes to methods after pilot trial commencement (such as eligibility criteria), with reasons	6
Participants	4a	Eligibility criteria for participants	3
	4b	Settings and locations where the data were collected	3
	4c	How participants were identified and consented	3
Interventions	5	The interventions for each group with sufficient details to allow replication, including how and when they were actually administered	4
Outcomes	6a	Completely defined prespecified assessments or measurements to address each pilot trial objective specified in 2b, including how and when they were assessed	4-5
	6b	Any changes to pilot trial assessments or measurements after the pilot trial commenced, with reasons	N/A
	6c	If applicable, prespecified criteria used to judge whether, or how, to proceed with future definitive trial	12
Sample size	7a	Rationale for numbers in the pilot trial	4
	7b	When applicable, explanation of any interim analyses and stopping guidelines	N/A
Randomisation:			
Sequence generation	8a	Method used to generate the random allocation sequence	4
	8b	Type of randomisation(s); details of any restriction (such as blocking and block size)	4
Allocation concealment mechanism	9	Mechanism used to implement the random allocation sequence (such as sequentially numbered containers), describing any steps taken to conceal the sequence until interventions were assigned	4

For peer review only

Implementation	10	Who generated the random allocation sequence, who enrolled participants, and who assigned participants to interventions	4
Blinding	11a	If done, who was blinded after assignment to interventions (for example, participants, care providers, those assessing outcomes) and how	4
	11b	If relevant, description of the similarity of interventions	4
Statistical methods	12	Methods used to address each pilot trial objective whether qualitative or quantitative	4-5
**Results**			
Participant flow (a diagram is strongly recommended)	13a	For each group, the numbers of participants who were approached and/or assessed for eligibility, randomly assigned, received intended treatment, and were assessed for each objective	6, fig 1
	13b	For each group, losses and exclusions after randomisation, together with reasons	6, fig 1
Recruitment	14a	Dates defining the periods of recruitment and follow-up	6
	14b	Why the pilot trial ended or was stopped	N/A
Baseline data	15	A table showing baseline demographic and clinical characteristics for each group	6-7
Numbers analysed	16	For each objective, number of participants (denominator) included in each analysis. If relevant, these numbers should be by randomised group	6
Outcomes and estimation	17	For each objective, results including expressions of uncertainty (such as 95% confidence interval) for any estimates. If relevant, these results should be by randomised group	6
Ancillary analyses	18	Results of any other analyses performed that could be used to inform the future definitive trial	6-12
Harms	19	All important harms or unintended effects in each group (for specific guidance see CONSORT for harms)	8
	19a	If relevant, other important unintended consequences	N/A
**Discussion**			
Limitations	20	Pilot trial limitations, addressing sources of potential bias and remaining uncertainty about feasibility	2, 12
Generalisability	21	Generalisability (applicability) of pilot trial methods and findings to future definitive trial and other studies	12
Interpretation	22	Interpretation consistent with pilot trial objectives and findings, balancing potential benefits and harms, and considering other relevant evidence	12
	22a	Implications for progression from pilot to future definitive trial, including any proposed amendments	12
**Other information**			
Registration	23	Registration number for pilot trial and name of trial registry	2
Protocol	24	Where the pilot trial protocol can be accessed, if available	9
Funding	25	Sources of funding and other support (such as supply of drugs), role of funders	2
	26	Ethical approval or approval by research review committee, confirmed with reference number	14

For peer review only

Citation: Eldridge SM, Chan CL, Campbell MJ, Bond CM, Hopewell S, Thabane L, et al. CONSORT 2010 statement: extension to randomised pilot and feasibility trials. BMJ. 2016;355.
*We strongly recommend reading this statement in conjunction with the CONSORT 2010, extension to randomised pilot and feasibility trials, Explanation and Elaboration for important clarifications on all the items. If relevant, we also recommend reading CONSORT extensions for cluster randomised trials, non-inferiority and equivalence trials, non-pharmacological treatments, herbal interventions, and pragmatic trials. Additional extensions are forthcoming: for those and for up to date references relevant to this checklist, see www.consort-statement.org.

For peer review only
